# Data for in-depth characterisation of the lamb meat proteome from *longissimus lumborum*

**DOI:** 10.1016/j.dib.2015.02.006

**Published:** 2015-02-20

**Authors:** Tzer-Yang Yu, James D. Morton, Stefan Clerens, Jolon M. Dyer

**Affiliations:** a Food & Bio-Based Products, AgResearch Lincoln Research Centre, New Zealand; b Wine, Food & Molecular Biosciences, Lincoln University, New Zealand; c Riddet Institute, based at Massey University, New Zealand; d Biomolecular Interaction Centre, University of Canterbury, New Zealand

## Abstract

This Data article provides Supplementary data related to the research article titled “In-depth characterisation of the lamb meat proteome from *longissimus lumborum*” by Yu et al. [1]. This research article reports the proteome catalogue of the 48 h post-mortem lamb *longissimus lumborum.* A list of 388 ovine-specific proteins were identified and characterised after separating the samples into sarcoplasmic, myofibrillar and insoluble fractions, followed by an in-depth shotgun proteomic evaluation and bioinformatic analysis. The detailed list of identified proteins, the annotated MS/MS spectra corresponding to the proteins identified by a single peptide-spectrum match, the raw Gene Ontology annotation data and other miscellaneous files, as will be described below, were contained in this Data article. We hope the data presented here will contribute to the current knowledge of the global protein composition of lamb skeletal muscle/meat.

**Table:** 

**Specifications table**	

Subject area	*Biology*

More specific subject area	*Skeletal muscle/meat proteomics*
Type of data	*Tables and figures*
How data was acquired	*SDS-PAGE and image acquisition:* photos of the gels were taken using a Nikon D100 digital camera over a light box. Gel images were labelled and marked using Corel Paint Shop Pro XI (Corel, Ottawa, Canada).
	*Mass spectrometry*: using an amaZon Speed ETD ion trap mass spectrometer (Bruker Daltonics, Bremen, Germany)
Data format	*Processed*
Experimental factors	*Skeletal muscle samples underwent post-mortem aging in a chiller*
Experimental features	*Sarcoplasmic and myofibrillar fractions: separated by SDS-PAGE, followed by in-gel trypsin digestion and LC–MS/MS*
	*Insoluble fraction: in-solution trypsin digestion and LC–MS/MS*
Data source location	*Canterbury, New Zealand*
Data accessibility	*The data is available with this article*

**Value of the data**

• The results of a list of 388 ovine-specific proteins identified are sorted into Excel worksheets corresponding to sarcoplasmic, myofibrillar and insoluble fractions. The peptide identification details (e.g., sequence, retention time, score) associated with the identified proteins were also presented. The data could serve as a reference for future studies on ovine skeletal muscle/meat.
• The protein identifications were accepted when they were mapped to: (1) at least two unique peptides at a posterior error probability (PEP) below 0.05, resulting in the false discovery rates (FDR) of the peptide-spectrum matches (PSMs) all below 2%; or (2) at least one unique peptide at a PEP below 0.01, resulting in the FDR of the PSMs all below 0.2%. The ProteinExtractor algorithm (Bruker Daltonics) was employed to minimise the protein identification redundancy.
• The Gene Ontology (GO) annotation(s) were associated with the identified proteins when applicable via the representative sequences which were retrieved from the public databases. The raw GO annotation files presented would allow an interested reader to look into the GO annotation relating to an identified protein by matching the UniProt ID of its corresponding representative sequence (Supplementary data 4) to the raw annotation files (Supplementary data 5–8, the last worksheet counting from the left).

## 1 Data, experimental design, materials and methods

### 1.1 Experimental design [1]

*Longissimus lumborum* samples were taken from five animals. The samples were pooled and separated in the sarcoplasmic, myofibrillar and insoluble fractions. The sarcoplasmic and myofibrillar fractions (in duplicate lanes for each fraction) were separated on SDS-PAGE gels as detailed in Table 1. The number of gel slices obtained from each gel lane, i.e., sub-fractions, is presented in Table 1. Each gel slice sample was analysed by a single LC–MS/MS run. The insoluble sample was analysed by LC–MS/MS without prior separation using two different gradients with triplicate runs for each gradient (Table 1). The MS/MS spectra files acquired were merged as specified in Table 1, resulting in four datasets for the Mascot searches post-processed with the Mascot Percolator.

### 1.2 SDS-PAGE for 4–20% T gels (retrieved from Ref. [1, Sections 2.4 and 2.5] with slight editing)

**Fig. 1:**
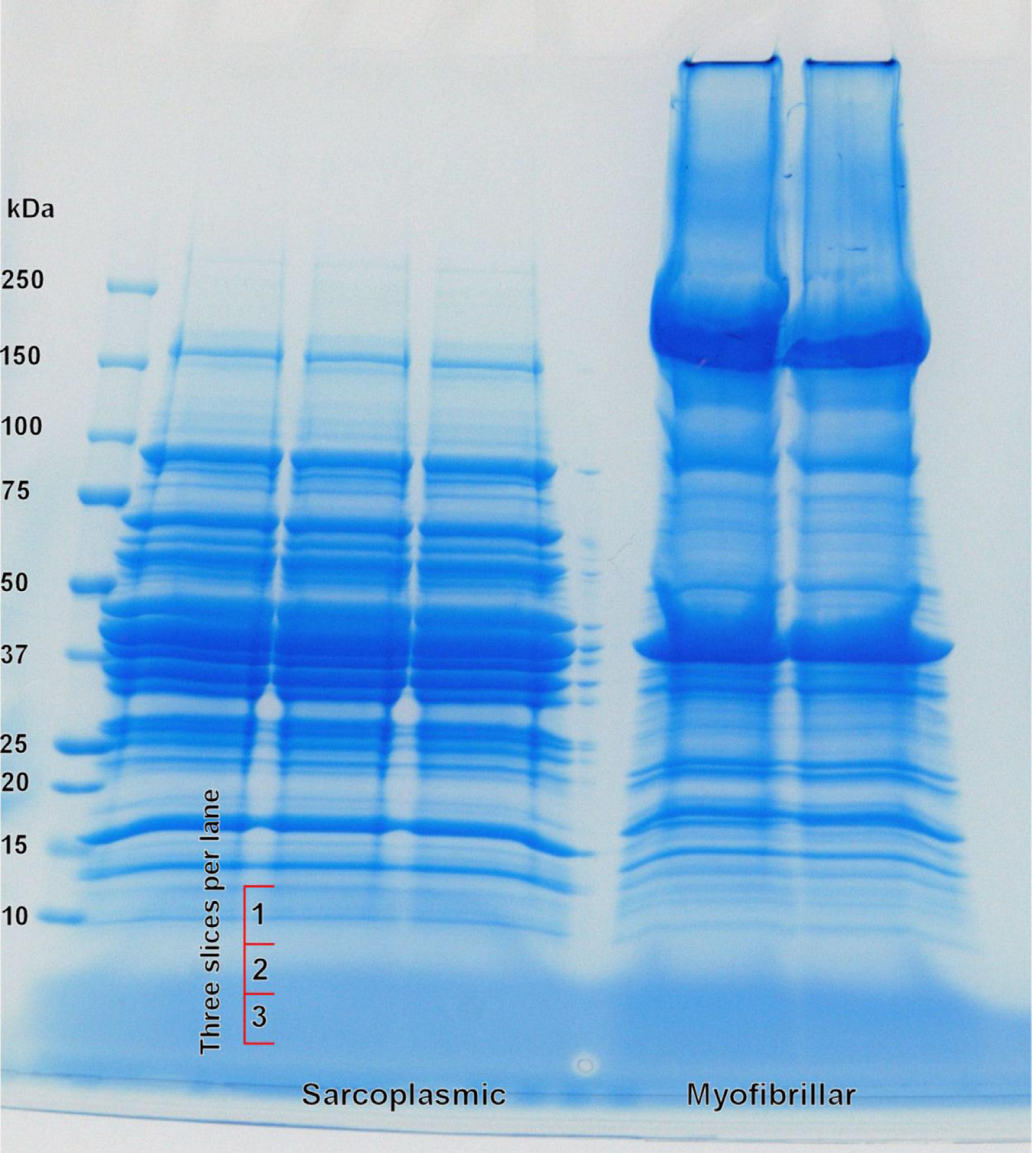
The 4–20% T gel that was run for LC–MS/MS analysis of low M_r_ region of the gel. The marks on the left hand side of the photograph indicate the approximate position of the gel lanes sliced for proteomic analysis.

The sarcoplasmic fraction was mixed with the SDS sample buffer at a ratio of 1:1 (v/v) and heated for 5 min at 95 °C with mild shaking. The myofibrillar fraction was heated directly in the same way. Protein fractions were separated on two 4–20% T Criterion Tris–HCl precast gels (Bio-Rad) at a constant voltage of 200 V, 80 mA and 15 W until the bromophenol blue dye front was about to reach the bottom of the gel. For Gel 1 [1, Fig. 1], 90 µg of sarcoplasmic or 147 µg myofibrillar protein fraction was loaded on a lane of a gel. For Gel 2 (Fig. 1): 88 µg of sarcoplasmic or 135 µg myofibrillar protein fraction was loaded on a lane of a gel. After electrophoresis, fixation was carried out in 50% ethanol (v/v), 10% acetic acid (v/v) for 30 min followed by colloidal Coomassie staining [2]. Gels were destained with Kimwipes (Kimberly–Clark) in Milli-Q water under gentle shaking.

For Gel 1, 15 gel sections of approximately equal length (about 5 mm) were excised from each of four gel lanes (duplicate for both sarcoplasmic and myofibrillar fractions) [1, Fig. 1]. For Gel 2, three gel sections of approximately equal length (about 4 mm) were excised from the low M_r_ region of each sarcoplasmic and myofibrillar fraction in duplicate (Fig. 1).

### 1.3 Tryptic protein digestions and LC–MS/MS

The methods for collecting proteomic data from the samples listed in Table 1 were referred to Ref. [1, Sections 2.5–2.7]. The number of MS/MS acquired from each experiment is summarised in Table 1.

### 1.4 The sequences augmented to the in-house NCBI ovine protein sequence database (some parts were retrieved from Ref. [1, Section 2.8.1] with slight editing)

The candidate sequences from the BGI Shenzen-predicted gene models (see Ref. [1, Section 2.8.1] for details) were retained for sequence annotation and updating the in-house sequence database. All entries with an identifier/name corresponding to keratin, hornerin, trypsin or macroglobulin were excluded from further analysis. These Oar v3 protein sequences were searched against the public NCBInr using NCBI BLAST to find similar sequences (required ≥60% query coverage, >70% max identity). One of the similar sequences, preferably from UniProtKB [3] or RefSeq protein sequence entries [4] that contain an accession # beginning with “NP”, i.e., “known protein” (http://asia.ensembl.org/info/docs/genebuild/genome_annotation.html) if applicable as well as with higher sequence coverage and per cent max identity, was chosen as a “representative sequence” to each protein sequence of interest for naming and retrieving Gene Ontology (GO) annotations if applicable. Multiple sequence alignment was conducted using ClustalW [5] to assess sequence completeness. For the alignment setting, Gap Open Penalty was set to 10 whereas Gap Extension Penalty, 0.2.

A meaningful name was then assigned to individual gene model identification based on the query coverage and max identity values of the “representative” sequences mentioned earlier in this section and the multiple sequence alignment results. The naming convention is described in Tables 2.1 and 2.2.

Annotated (candidate) protein sequences were curated using CD-HIT-2D [6,7] against the NCBI *Ovis aries* protein sequence database (August 27, 2013; 30,406 sequences). A local BLAST command line was used for curation, as shown below:

cd-hit-2d -i NR_9940_27082013.fasta -i2 in_house_Oar_v3_082013.txt -o NR_vs_ih_local_70%id51%cov_S2_30000 -G 0 -c 0.7 -aS 0.51 -n 5 -S2 30000

where the file name after -i was the NCBI ovine database and the file name after -i2 was the annotated candidate sequences described above. The file name after -o was the output sequence file which only retained the candidate sequences that exhibited less than 70% sequence identity with at least 51% alignment coverage for the shorter sequence. This choice was made to avoid taking potentially redundant sequences already exiting in the NCBI sequence database, which contained the protein sequences predicted from Oar v3 by the NCBI׳s own pipeline (http://www.ncbi.nlm.nih.gov/genome/annotation_euk/process/). Description of the commands is referred to the CD-Hit User׳s Guide (http://weizhong-lab.ucsd.edu/cd-hit/wiki/doku.php?id=cd-hit_user_guide).

Sequences that remained after curation were merged with the NCBI *Ovis aries* protein sequences (*NCBI Taxonomy: 9940*; Aug 27, 2013) and the in-house sheep protein sequences [8] to create a combined database for the final Mascot search. These Oar v3 sequences along with their corresponding representative sequences and their proposed names are listed in Supplementary data 1a.

### 1.5 The protein identification (part of this section was retrieved from Ref. [1, Section 2.8.1])

The protein identification approach is described in Ref. [1, Section 2.8.1]. The final protein identification results based on the Mascot Percolator validated PSMs using the following criteria: (1) at least two unique peptides at a posterior error probability (PEP) below 0.05; or (2) at least one unique peptide at a PEP below 0.01 [1] is reported in Supplementary data 2. The associated peptide identification results are also included in the Supplementary data file. For the proteins identified by a single PSM, their associated ProteinScape (v3.1.0, Bruker Daltonics) annotated spectra are reported in Supplementary data 3a–3c (3a, sarcoplasmic fraction; 3b, myofibrillar fraction; 3c, SDS-insoluble pellet). The FDR of these final database searches post-processed with the Mascot Percolator are provided in Supplementary data 4. The Mascot Percolator validated results formed the basis for the 48 h lamb *longissimus lumborum* proteome characterisation reported in this study.

Protein identifications of individual gel slices were based on non-Percolator Mascot searches because the Percolator works best if there are several thousand spectra, which was not applicable to the data sets of these individual slices. Protein identification results of individual gel slices and the search parameter are shown in Supplementary data 1b, 1c, 1d and 1e. These results were used for discussing the gel profile but not for proteome characterisation that involved only the Percolator-post-processed results.

### 1.6 Function prediction

The process of function prediction for the list of validated protein identifications is described in Ref. [1, Section 2.8.2]. The validated protein identifications along with their UniProtKB-derived representative sequences and the BLAST results are given in Supplementary data 4. Details of GO annotations for the representative sequences are provided in Supplementary data 5 (molecular function; the total protein list), Supplementary data 6 (molecular function; the sarcoplasmic fraction), Supplementary data 7 (cellular component; the total protein list) and Supplementary data 8 (cellular component; the sarcoplasmic fraction). The InterProScan results for the protein identifications that did not have a UniProtKB representative sequence or did not map to any GO annotations of molecular function or cellular component aspect are given in Supplementary data 4.

## Figures and Tables

**Table 1:** Summary of the MS/MS datasets used for protein identification.

Dataset/experiment	Sub-fractions	Replicates	LC-analytical gradients (% mobile phase B)	# LCMS/MS runs	# MS/MS spectra

Sarcoplasmic fraction, 4–20% T gel	15	2	0–45% in 45 min; 800 nL/min	30	18617
Sarcoplasmic fraction, 4–20% T gel, low molecular mass region	3	2		6	
Myofribrillar fraction, 4–20% T gel	15	2		30	26687
Myofribrillar fraction, 4–20% T gel, low molecular mass region	3	2		6	
Myofibrillar fraction, 5% T gel	11	2		22	9146
SDS-insoluble pellet	1	3	0–45% in 60 min; 500 nL/min	3	12447
		3	0–6% to 31–61% in 60 min; 500 nL/min	3	
			Total #	100	66897

**Table 2.1:** Naming convention used to name the identified gene models.

**Criterion**	**Qualifier**
100%>per cent identity≥90%	*homologue to* (*the name of the* “*representative sequence*”)
90%>per cent identity≥70%	*similar to* (*the name of the* “*representative sequence*”)
per cent identity<70%	*weakly similar to* (*the name of the* “*representative sequence*”)

**Table 2.2:** Naming convention used to indicate (predicted) sequence completeness of the identified gene models.

**Criterion**	**Qualifier**
Neither truncation nor missing aa region(s) within a sequence	(*nothing*)
Hard to resolve based on multiple sequence alignment. For example, not-so-good overall alignment, good matching only on certain part(s) of the sequence	*tentatively partial*
Obvious truncation and/or missing aa region(s) within a sequence	*partial*
