# Supplementary material for: Data for in-depth characterisation of the lamb meat proteome from longissimus lumborum
Source: Data Brief. 2015 Feb 20;3:143–8. doi: 10.1016/j.dib.2015.02.006 (PMC4510072; doi:10.1016/j.dib.2015.02.006)

agresearch

Spectrum Report

Source: M:/Documents/Lamb meat protein project/1. Characterisation of lamb skeletal proteome/Real run - 5 lambs from LCF/mgf_Obj_1/Sarc_4-20pc_sarc_15B-17B_concat_all_the_line_dele.mgf

Protein: PREDICTED: heat shock 70 kDa protein 1-like [Ovis aries]

Accession: gi|426250526|ref|XP_004018986.1|

Parent m/z: 733.406, 2+

Sequence: K.AKIHDIVLVGGSTR.I

Score: 47.281096251637024

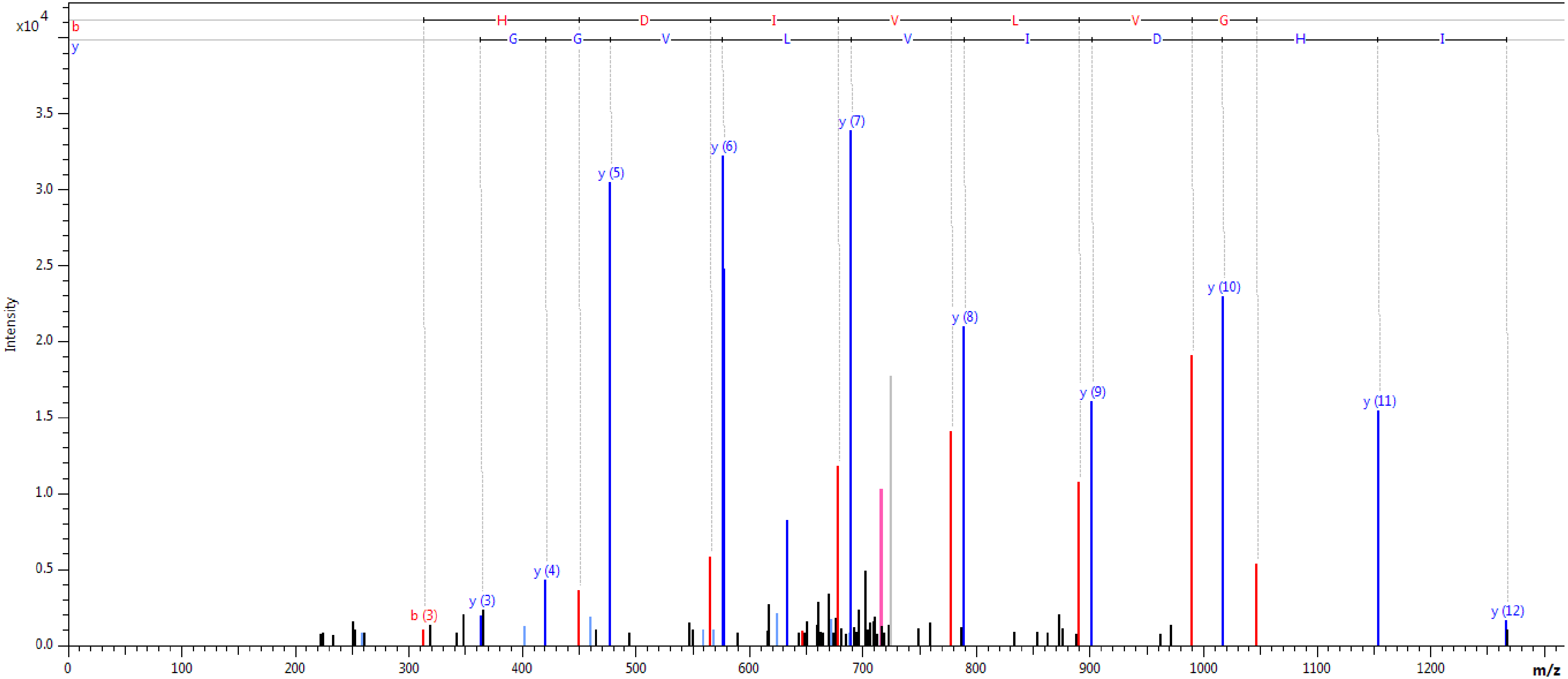

Cmpd 455, +MSn(733.41), 1023.9 s [Cmpd 455, +MSn(733.41), 1023.9 s]

Spectrum Report

Source: M:/Documents/Lamb meat protein project/1. Characterisation of lamb skeletal proteome/Real run - 5 lambs from LCF/mgf_Obj_1/Sarc_4-20pc_sarc_15B-17B_concat_all_the_line_dele.mgf

Protein: PREDICTED: glutathione S-transferase Mu 1-like isoform 2 [Ovis aries]

Accession: gi|426216182|ref|XP_004002345.1|

Parent m/z: 808.912, 2+

Sequence: R.LLLEYTDSNYEEK.K

Score: 118.84529283759429

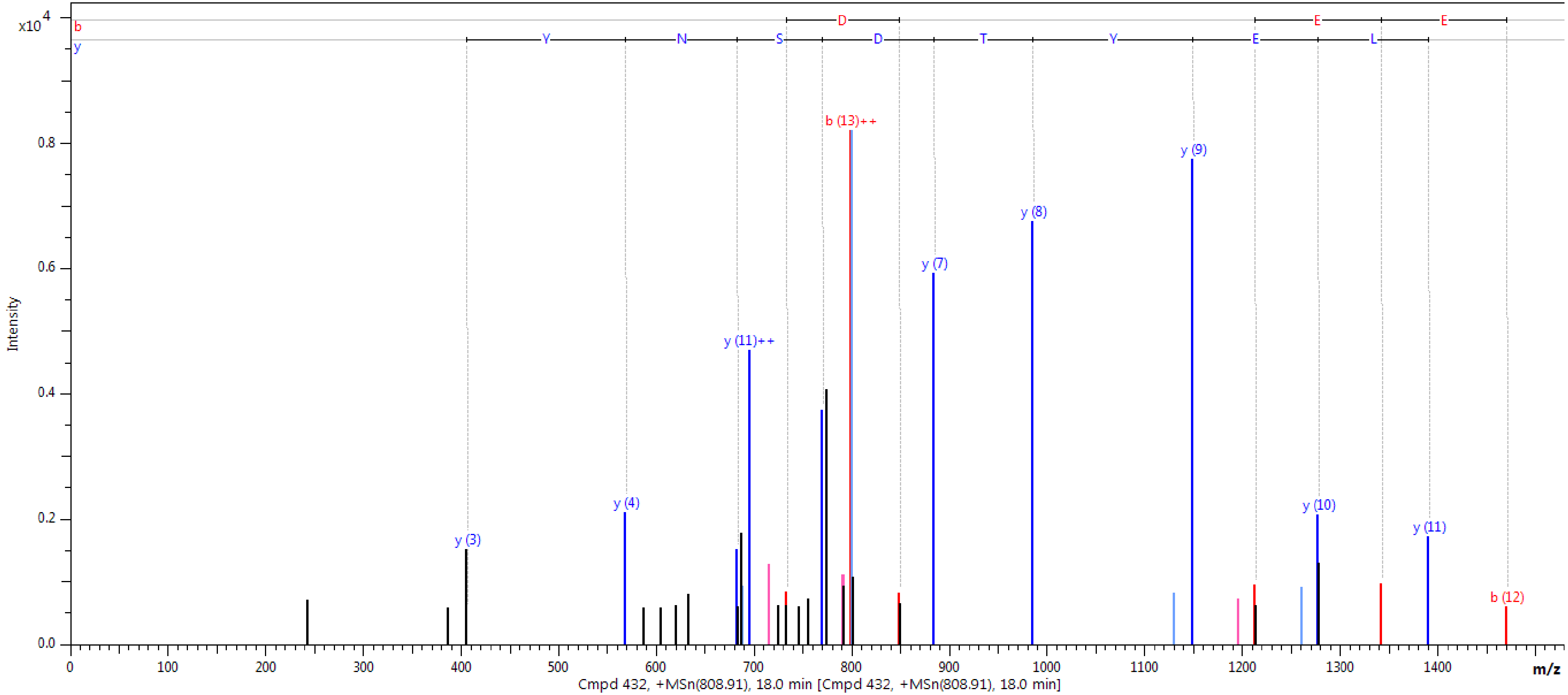

agresearch

Spectrum Report

Source: M:/Documents/Lamb meat protein project/1. Characterisation of lamb skeletal proteome/Real run - 5 lambs from LCF/mgf_Obj_1/Sarc_4-20pc_sarc_15B-17B_concat_all_the_line_dele.mgf
Protein: PREDICTED: glyceraldehyde-3-phosphate dehydrogenase, testis-specific [Ovis aries]
Accession: gi|426243707|ref|XP_004015692.1|
Parent m/z: 786.452, 2+
Sequence: R.VPTPDVSVVDLTCR.L
Score: 80.44689475342835

Modification: Propionamide: 13

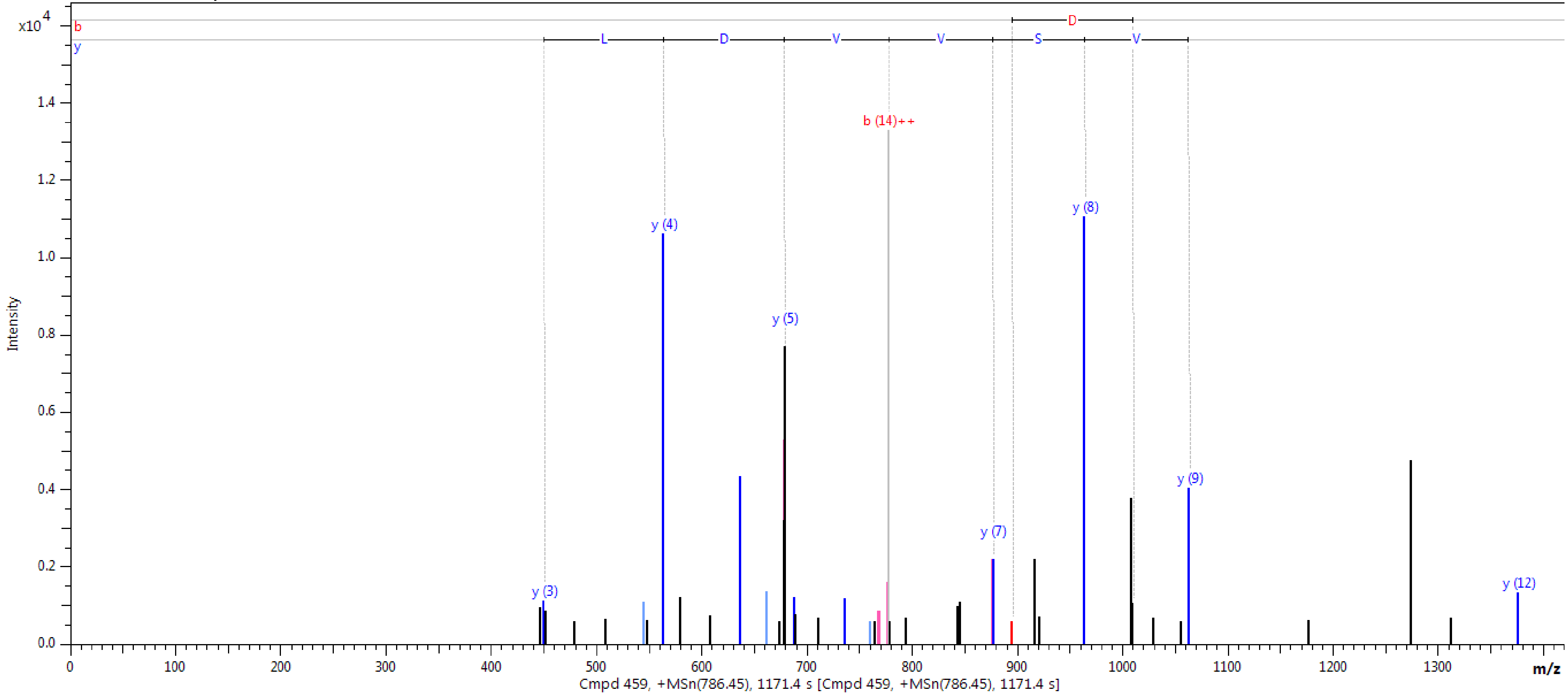

Spectrum Report

Source: M:/Documents/Lamb meat protein project/1. Characterisation of lamb skeletal proteome/Real run - 5 lambs from LCF/ mgf_Obj_1/Sarc_4-20pc_sarc_15B-17B_concat_all_the_line_dele.mgf

Protein: PREDICTED: heat shock protein HSP 90-beta [Ovis aries]

Accession: gi|426250357|ref|XP_004018903.1|

Parent m/z: 614.882, 2+

Sequence: K.ADLVNNLGTIAK.S

Score: 26.247663217402817

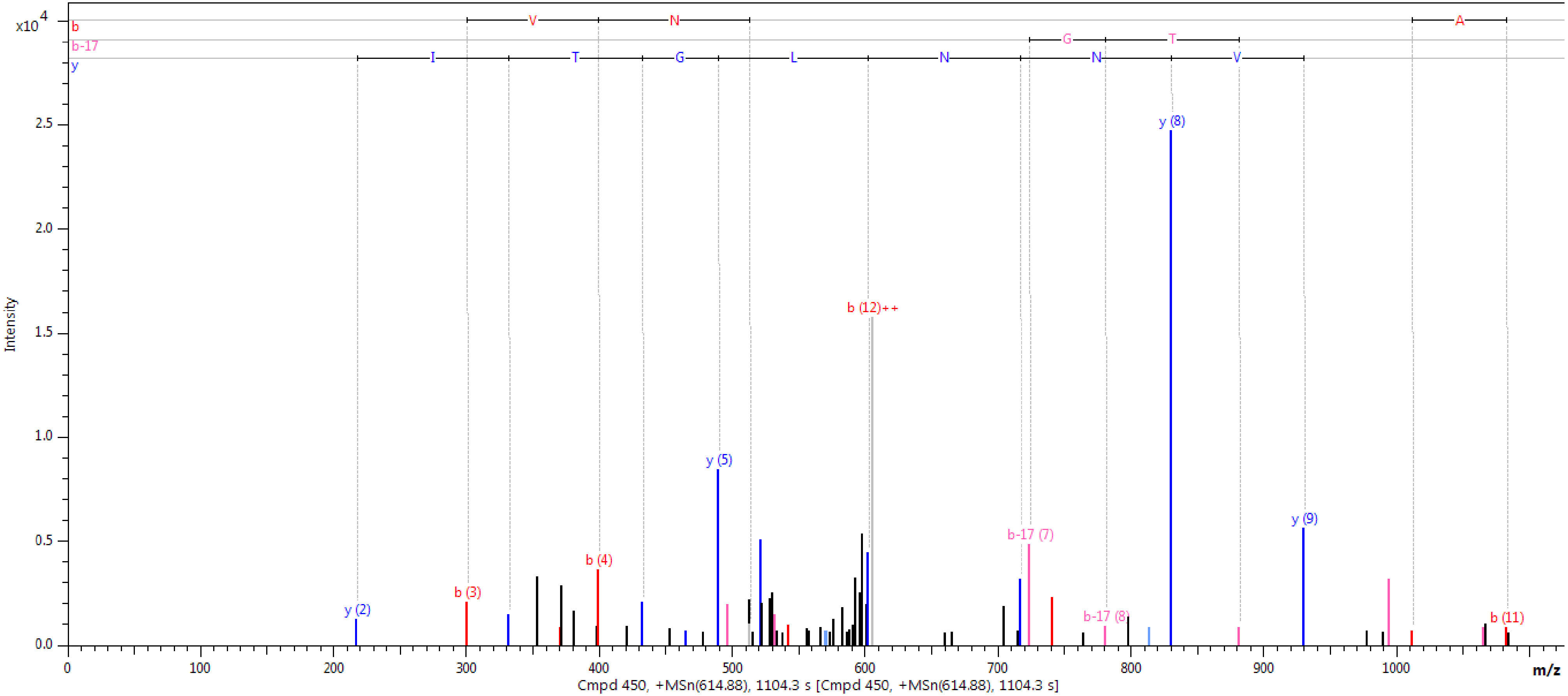

agresearch

Spectrum Report

Source: M:/Documents/Lamb meat protein project/1. Characterisation of lamb skeletal proteome/Real run - 5 lambs from LCF/mgf_Obj_1/Sarc_4-20pc_sarc_15B-17B_concat_all_the_line_dele.mgf
Protein: PREDICTED: profilin-1-like [Ovis aries]
Accession: gi|426231105|ref|XP_004009583.1| **Parent m/z:** 607.352, 2+
Sequence: I.DSPSVWAAVPGK.T **Score:** 26.559065293596163

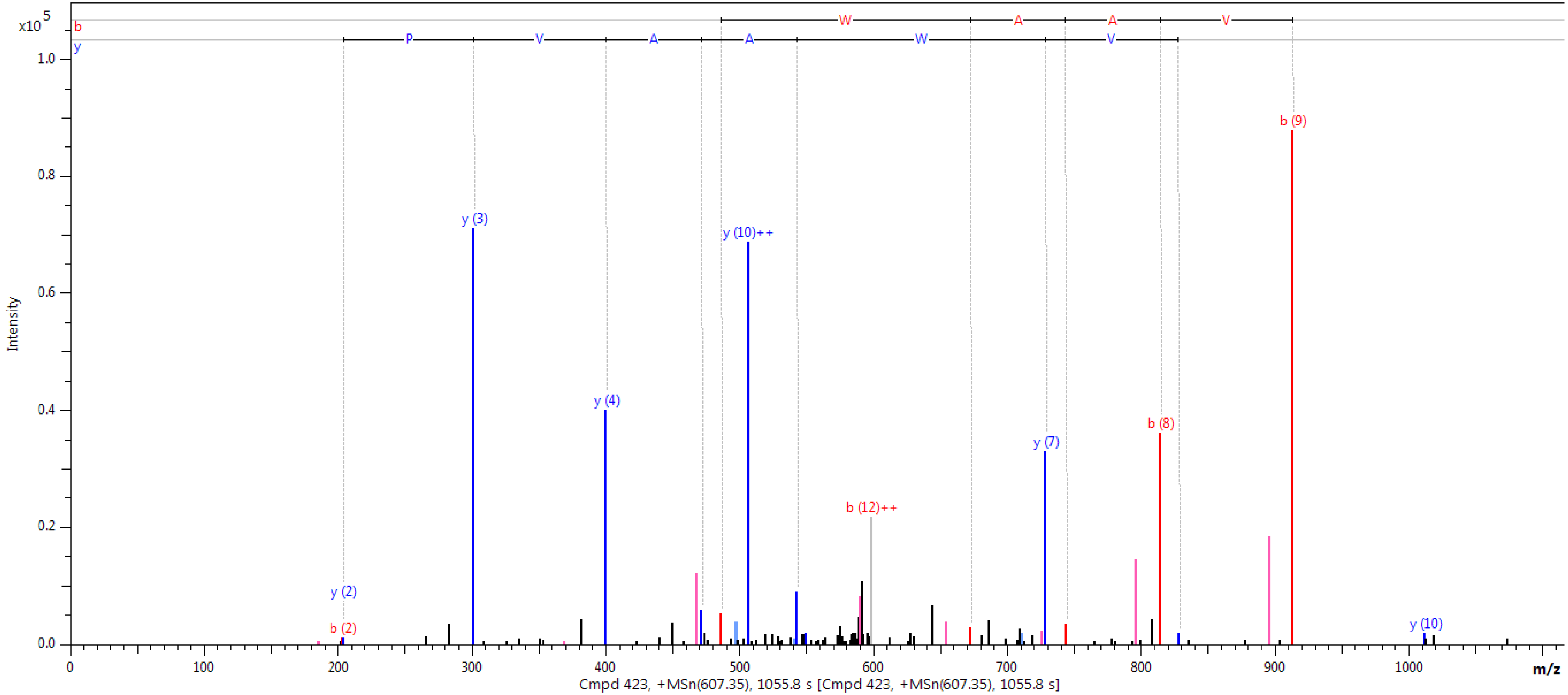

Spectrum Report

Source: M:/Documents/Lamb meat protein project/1. Characterisation of lamb skeletal proteome/Real run - 5 lambs from LCF/ mgf_Obj_1/Sarc_4-20pc_sarc_15B-17B_concat_all_the_line_dele.mgf
Protein: PREDICTED: succinate dehydrogenase [ubiquinone] flavoprotein subunit, mitochondrial [Ovis aries]
Accession: gi|426246735|ref|XP_004017146.1| **Parent m/z:** 737.464, 2+
Sequence: R.LGANSLLDLVVFGR.A **Score:** 153.49422462689344

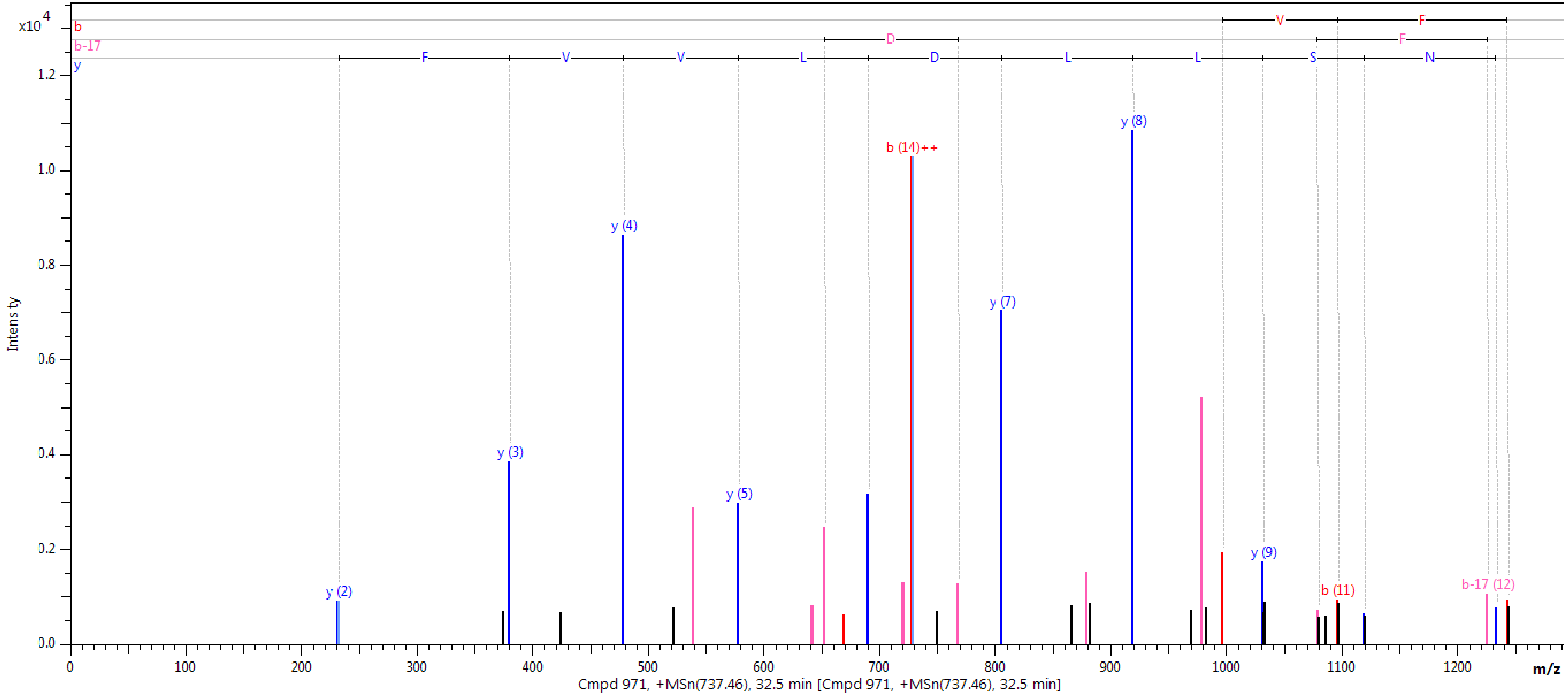

agresearch

Spectrum Report

Source: M:/Documents/Lamb meat protein project/1. Characterisation of lamb skeletal proteome/Real run - 5 lambs from LCF/ mgf_Obj_1/Sarc_4-20pc_sarc_15B-17B_concat_all_the_line_dele.mgf
Protein: similar to Serine/threonine-protein kinase SIK3, partial [Ovis aries: Oar v3]
Accession: gi|1999014709|gb|1999014709.1|
Parent m/z: 608.914, 2+
Sequence: K.VSILAAIDEASK.K
Score: 126.40502222801734

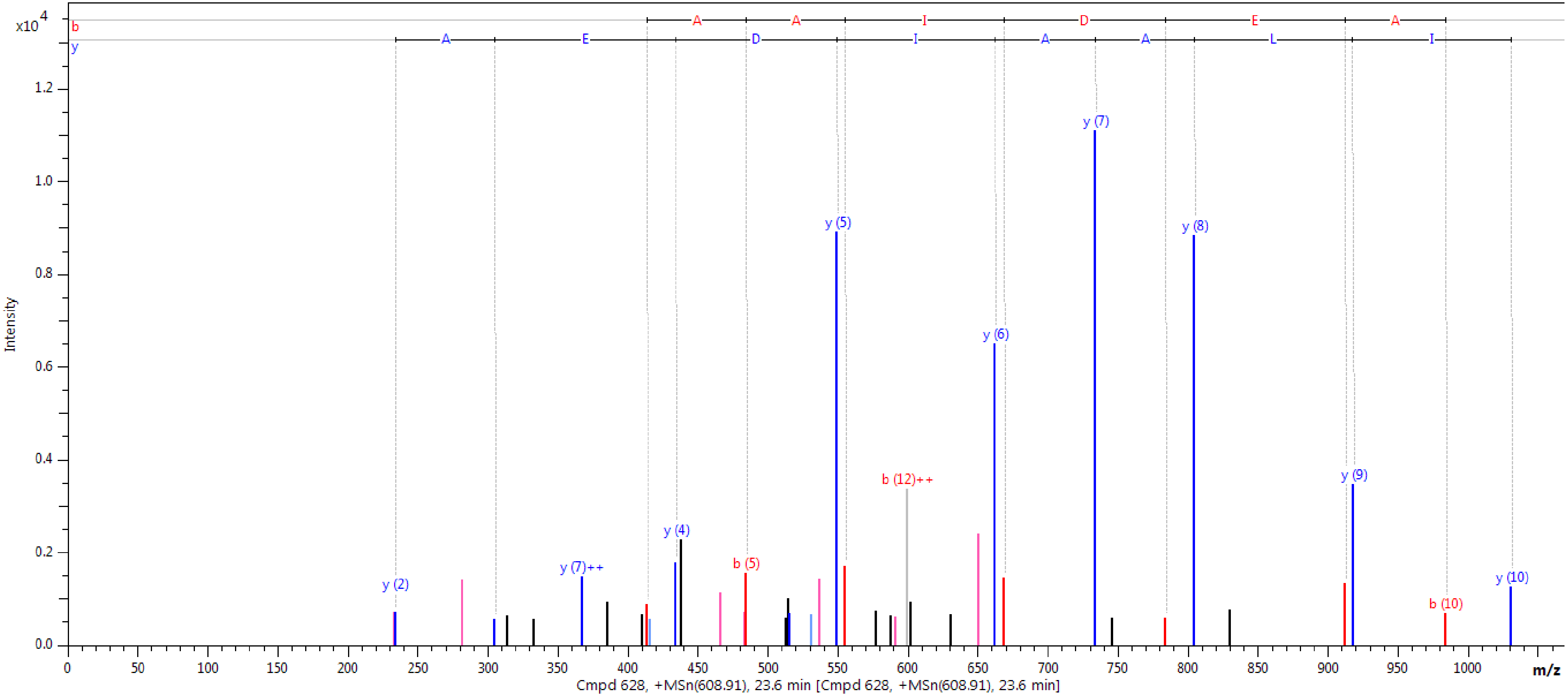

Spectrum Report

Source: M:/Documents/Lamb meat protein project/1. Characterisation of lamb skeletal proteome/Real run - 5 lambs from LCF/mgf_Obj_1/Sarc_4-20pc_sarc_15B-17B_concat_all_the_line_dele.mgf
Protein: PREDICTED: peptidyl-prolyl cis-trans isomerase FKBP1A [Ovis aries]
Accession: gi|426241909|ref|XP_004014822.1| **Parent m/z:** 767.362, 2+
Sequence: R.GWEEGVAQMSVGQR.A **Score:** 153.62638376231394

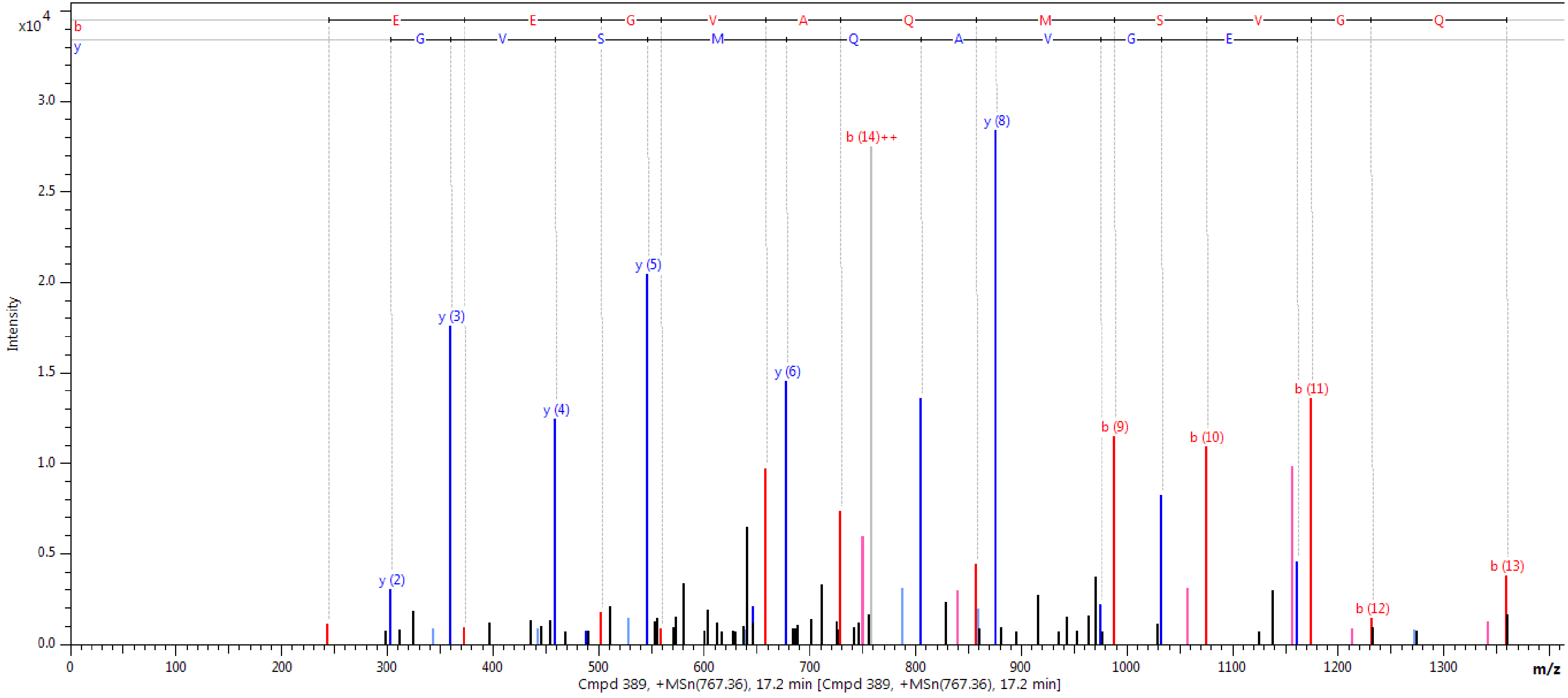

Spectrum Report

Source: M:/Documents/Lamb meat protein project/1. Characterisation of lamb skeletal proteome/Real run - 5 lambs from LCF/mgf_Obj_1/Sarc_4-20pc_sarc_15B-17B_concat_all_the_line_dele.mgf

Protein: PREDICTED: SH3 domain-binding glutamic acid-rich protein [Ovis aries]

Accession: gi|426218329|ref|XP_004003401.1| **Parent m/z:** 654.451, 2+

Sequence: K.VFVATSSGSIAIR.K **Score:** 153.62638376231394

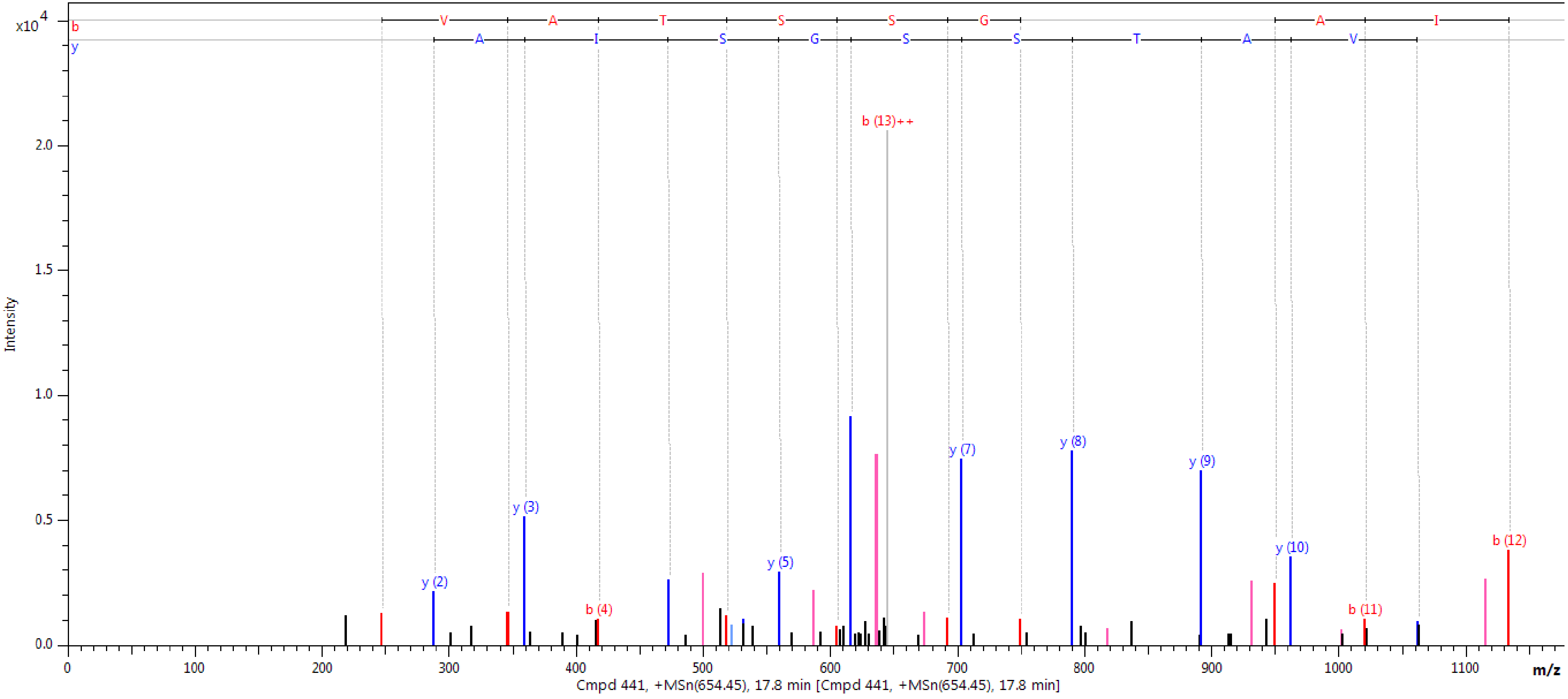

Spectrum Report

Source: M:/Documents/Lamb meat protein project/1. Characterisation of lamb skeletal proteome/Real run - 5 lambs from LCF/mgf_Obj_1/Sarc_4-20pc_sarc_15B-17B_concat_all_the_line_dele.mgf
Protein: PREDICTED: L-lactate dehydrogenase A-like 6B-like [Ovis aries]
Accession: gi|426234994|ref|XP_004011476.1| **Parent m/z:** 965.583, 2+
Sequence: K.LIVVSNPVDILTYVAWK.L **Score:** 153.62638376231394

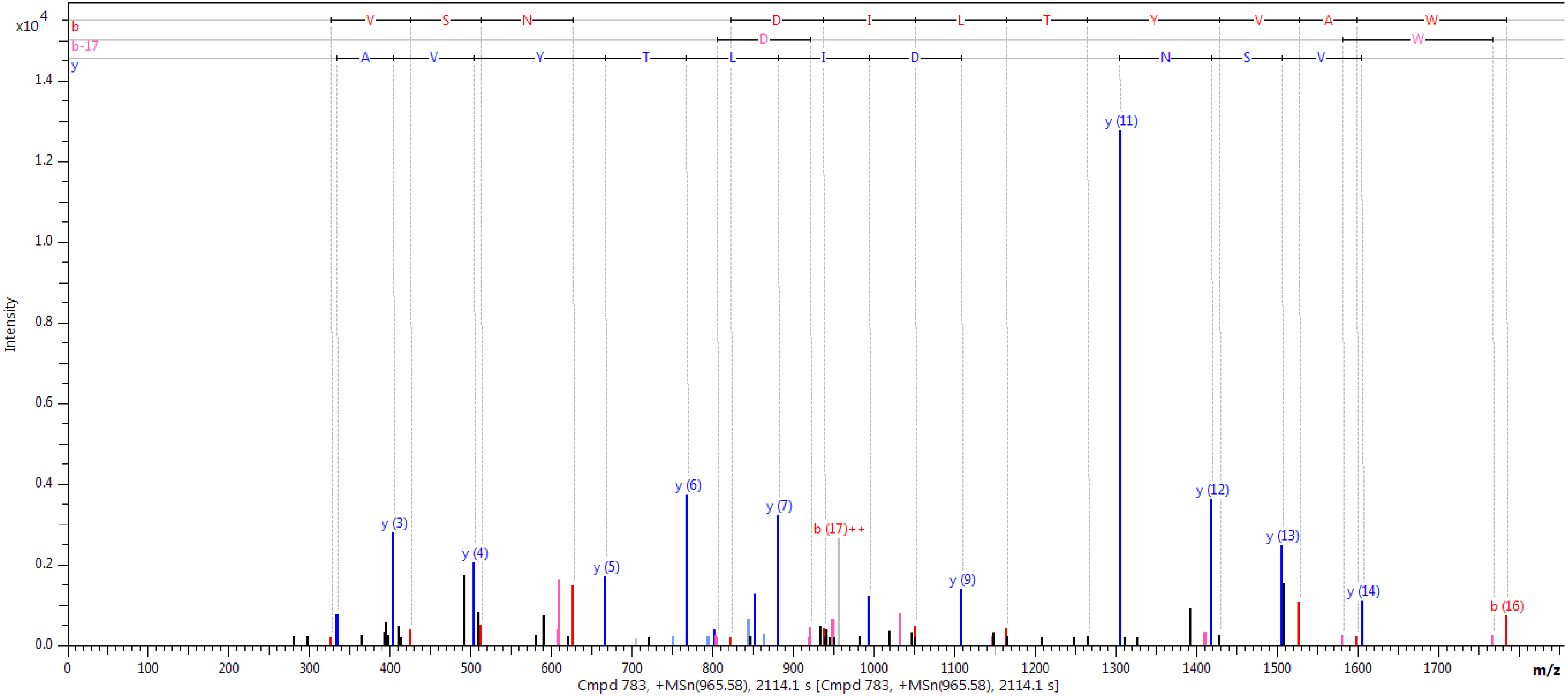

agresearch

Spectrum Report

Source: M:/Documents/Lamb meat protein project/1. Characterisation of lamb skeletal proteome/Real run - 5 lambs from LCF/mgf_Obj_1/Sarc_4-20pc_sarc_15B-17B_concat_all_the_line_dele.mgf
Protein: PREDICTED: elongation factor 1-gamma [Ovis aries]
Accession: gi|426252412|ref|XP_004019908.1|
Sequence: K.ALIAAQYSGAQVR.V
Parent m/z: 674.376, 2+
Score: 136.2885267834785

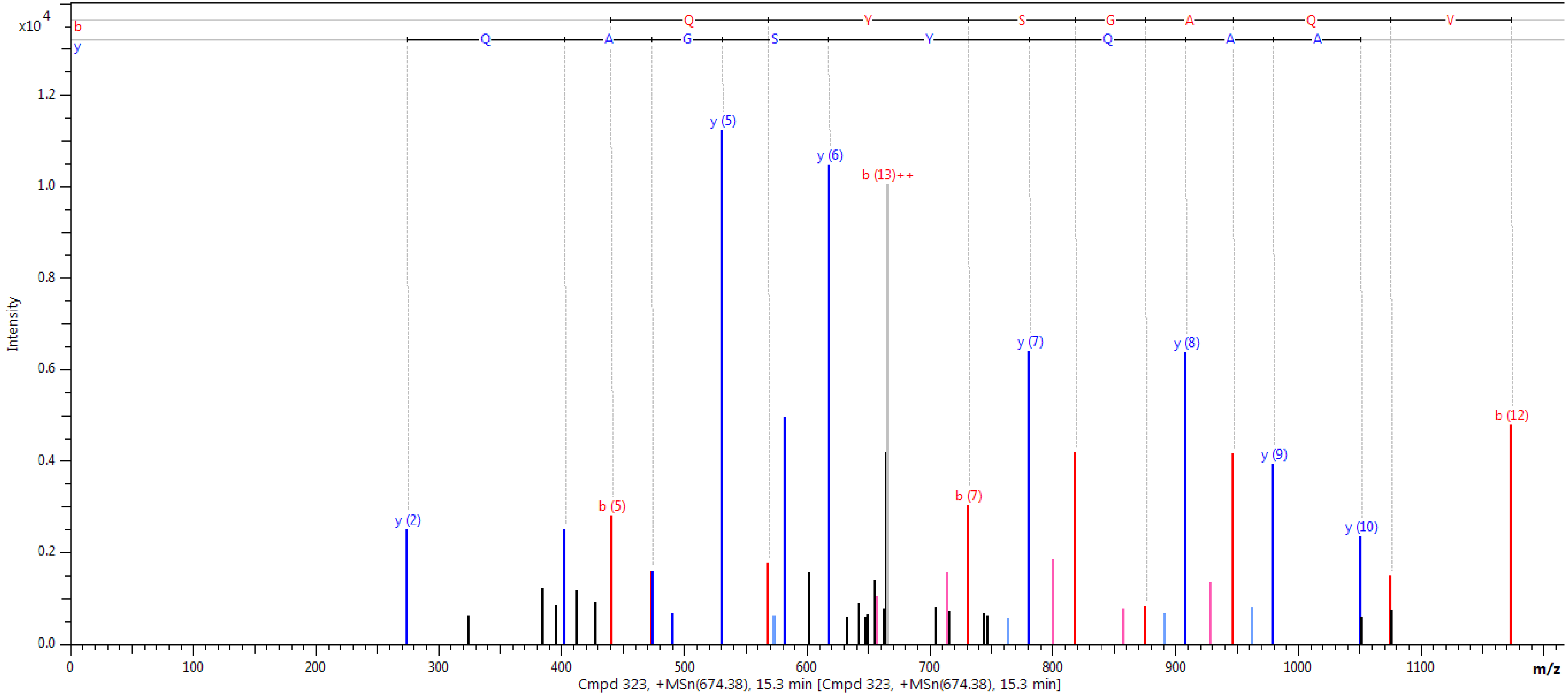

Spectrum Report

Source: M:/Documents/Lamb meat protein project/1. Characterisation of lamb skeletal proteome/Real run - 5 lambs from LCF/mgf_Obj_1/Sarc_4-20pc_sarc_15B-17B_concat_all_the_line_dele.mgf
Protein: PREDICTED: trans-1,2-dihydrobenzene-1,2-diol dehydrogenase [Ovis aries]
Accession: gi|426243119|ref|XP_004015411.1|
Parent m/z: 615.361, 2+
Sequence: R.SVLAQGTLGDLR.V
Score: 115.39715712064461

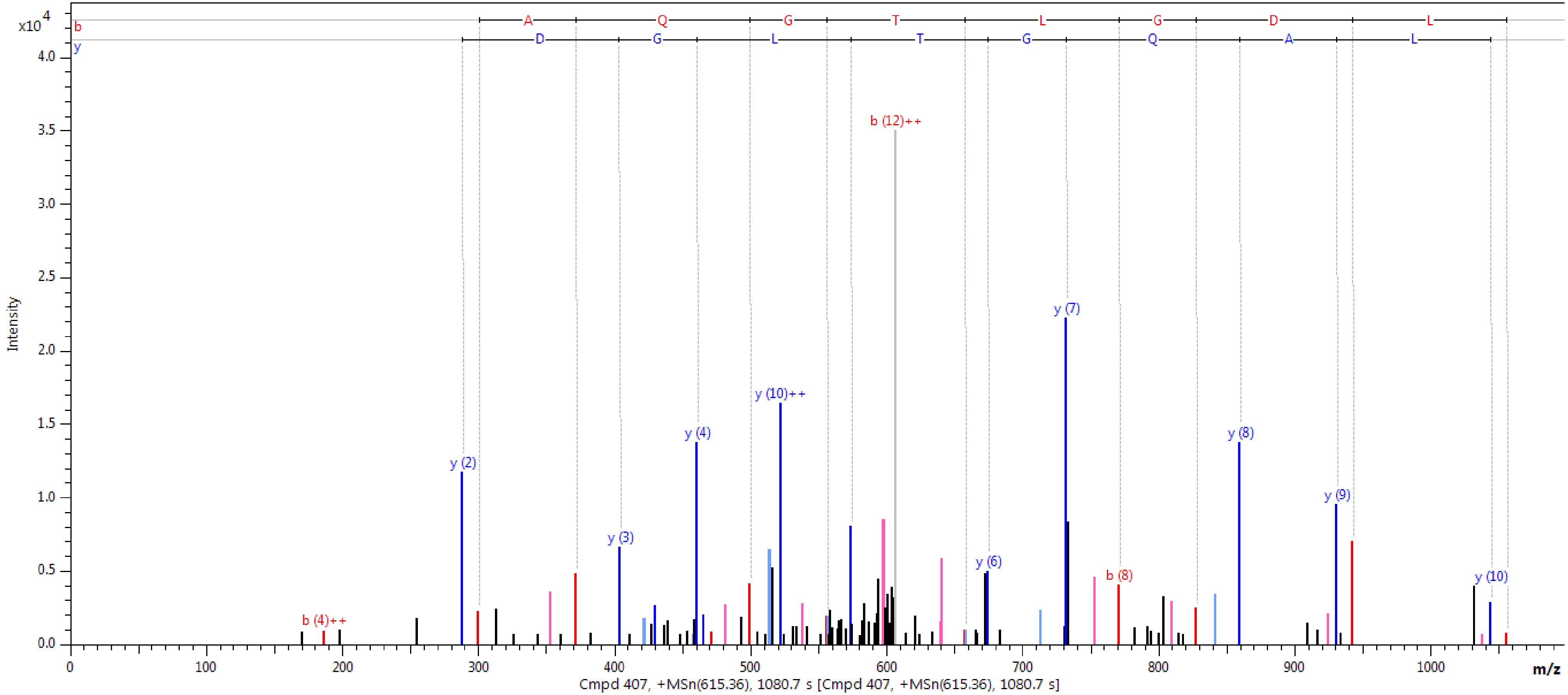

Spectrum Report

Source: M:/Documents/Lamb meat protein project/1. Characterisation of lamb skeletal proteome/Real run - 5 lambs from LCF/ mgf_Obj_1/Sarc_4-20pc_sarc_15B-17B_concat_all_the_line_dele.mgf
Protein: PREDICTED: profilin-2 [Ovis aries]
Accession: gi|426219397|ref|XP_004003912.1|
Sequence: K.SQGGEPTYNVAVGR.A
Parent m/z: 717.885, 2+
Score: 125.33199085286168

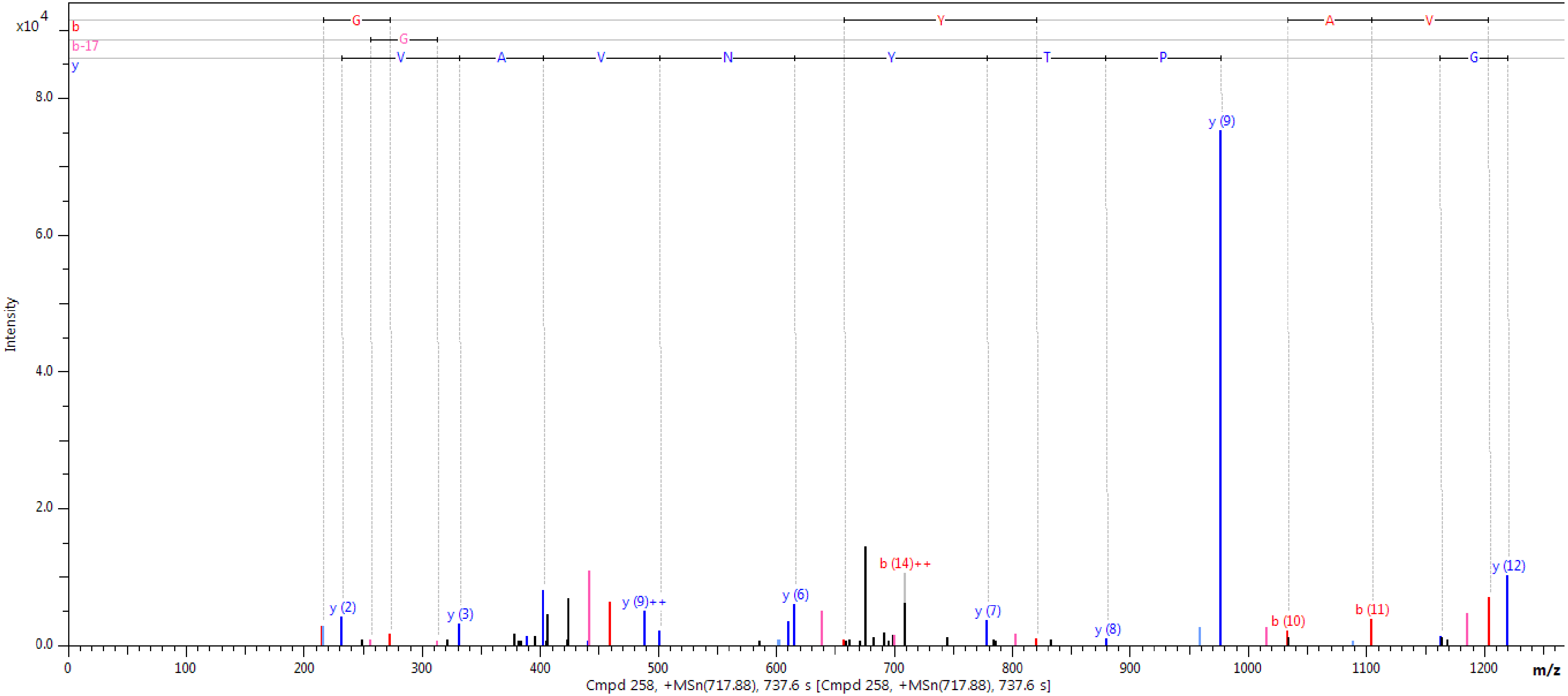

Spectrum Report

Source: M:/Documents/Lamb meat protein project/1. Characterisation of lamb skeletal proteome/Real run - 5 lambs from LCF/mgf_Obj_1/Sarc_4-20pc_sarc_15B-17B_concat_all_the_line_dele.mgf
Protein: PREDICTED: serpin A3-5-like, partial [Ovis aries]
Accession: gi|426248890|ref|XP_004018190.1|
Sequence: K.IFTDAADLSGVTGTR.N
Parent m/z: 762.403, 2+
Score: 153.62638376231394

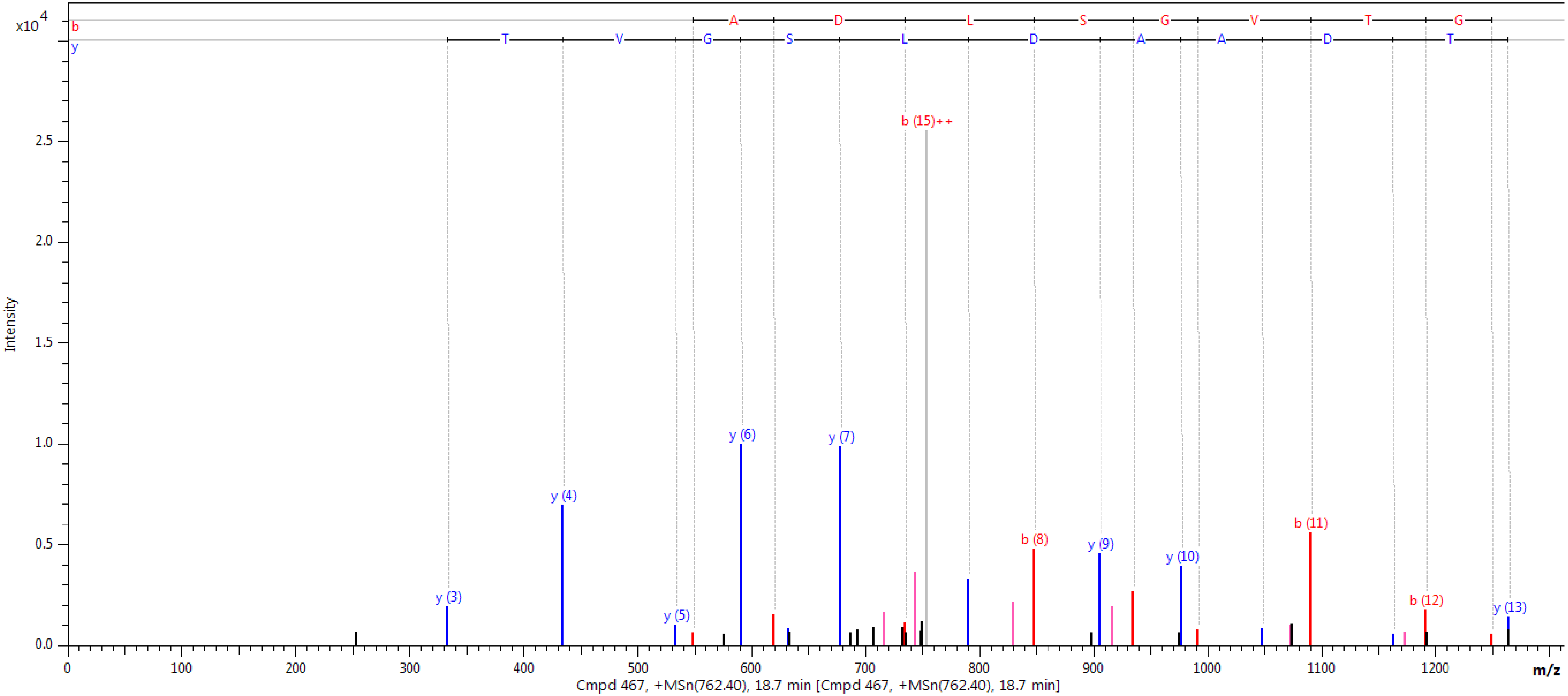

Spectrum Report

Source: M:/Documents/Lamb meat protein project/1. Characterisation of lamb skeletal proteome/Real run - 5 lambs from LCF/mgf_Obj_1/Sarc_4-20pc_sarc_15B-17B_concat_all_the_line_dele.mgf

Protein: PREDICTED: glycogenin-1 [Ovis aries]

Accession: gi|426219401|ref|XP_004003914.1|

Parent m/z: 908.959, 2+

Sequence: P.QDQAFVTLTTNDSYAK.G

Score: 153.62638376231394

Modification: Methyl: 2; Deamidated: 3

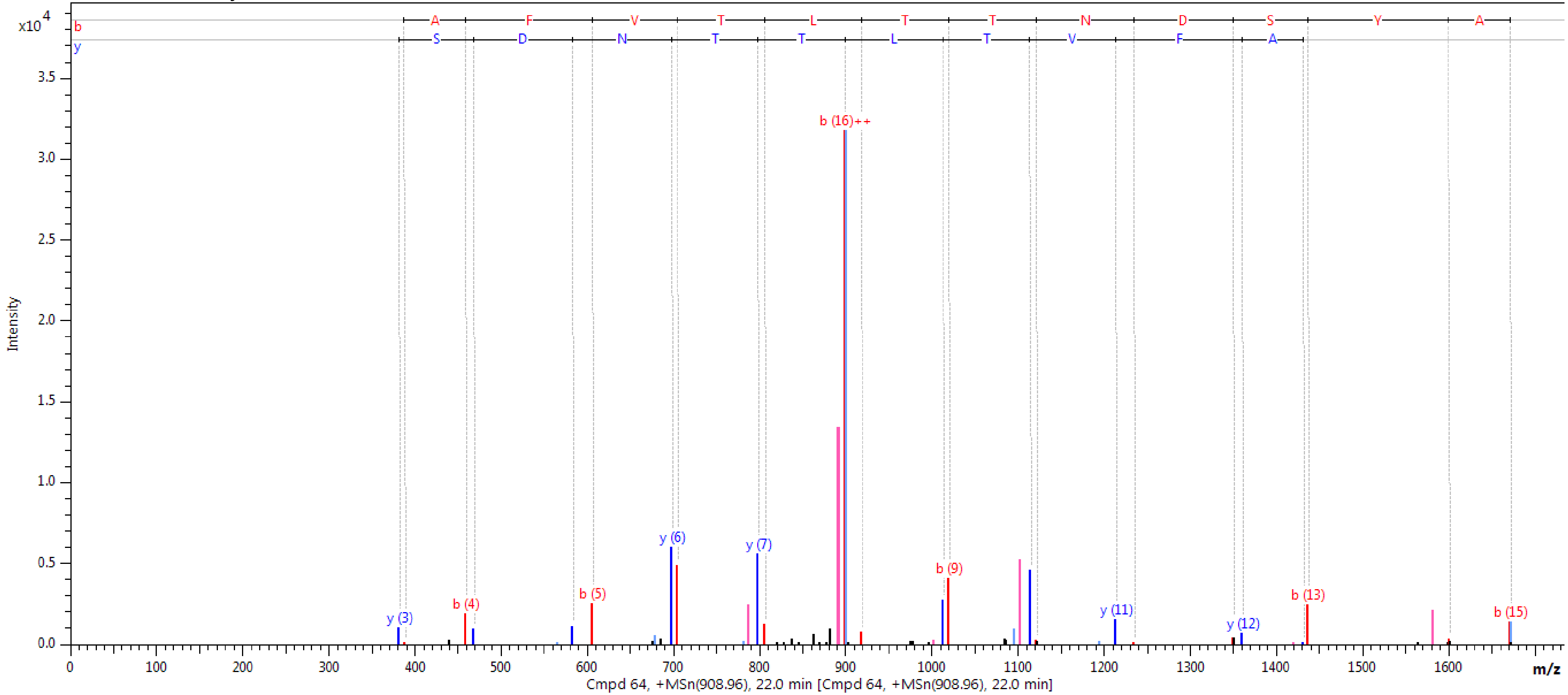

Spectrum Report

Source: M:/Documents/Lamb meat protein project/1. Characterisation of lamb skeletal proteome/Real run - 5 lambs from LCF/mgf_Obj_1/Sarc_4-20pc_sarc_15B-17B_concat_all_the_line_dele.mgf

Protein: PREDICTED: LOW QUALITY PROTEIN: heat shock protein beta-1 [Ovis aries]

Accession: gi|426255344|ref|XP_004021310.1| **Parent m/z:** 582.393, 2+

Sequence: R.LFDQAFGLPR.L **Score:** 72.81967735642456

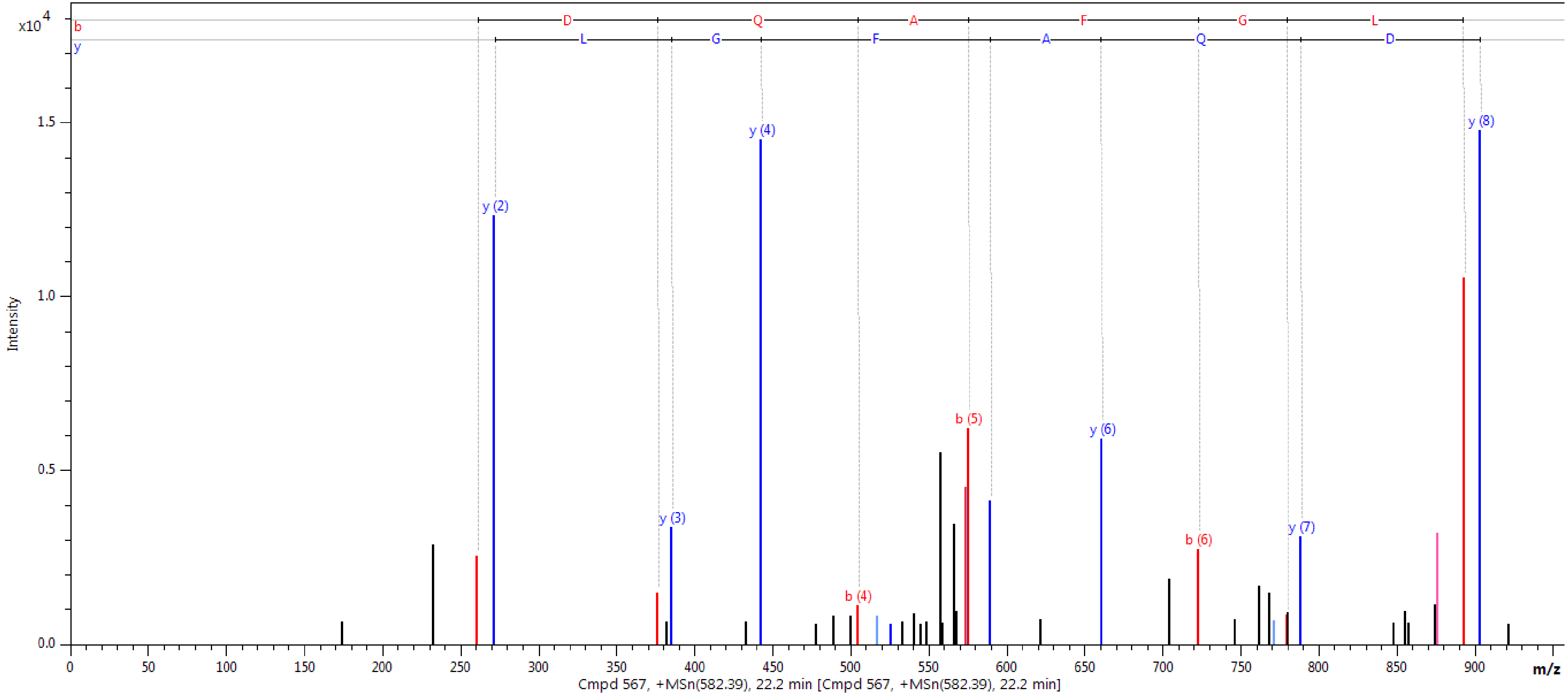

Spectrum Report

Source: M:/Documents/Lamb meat protein project/1. Characterisation of lamb skeletal proteome/Real run - 5 lambs from LCF/ mgf_Obj_1/Sarc_4-20pc_sarc_15B-17B_concat_all_the_line_dele.mgf
Protein: PREDICTED: protein S100-A11 [Ovis aries]
Accession: gi|426216616|ref|XP_004002557.1|
Sequence: R.CIESLIAVFQK.H
Parent m/z: 661.305, 2+
Score: 99.85471061043201

Modification: Propionamide: 1

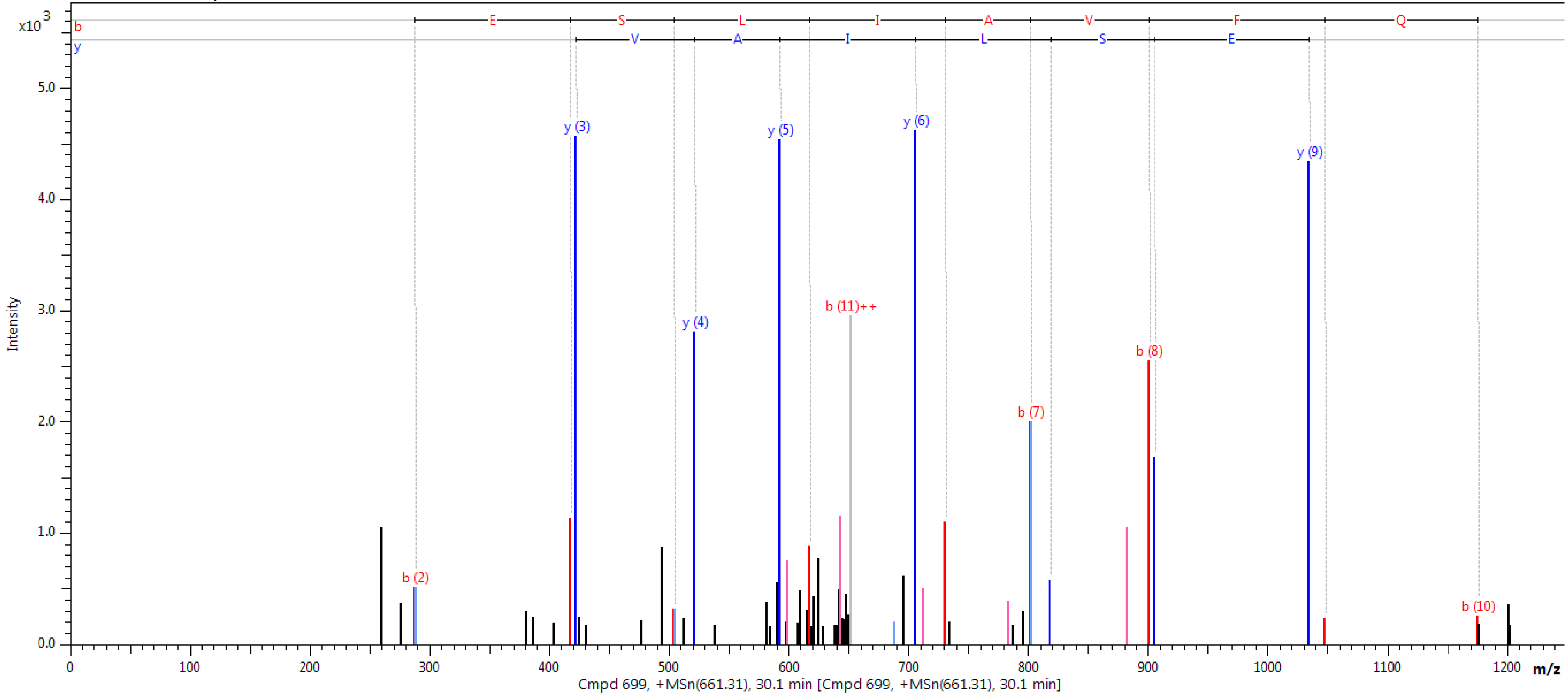

Spectrum Report

Source: M:/Documents/Lamb meat protein project/1. Characterisation of lamb skeletal proteome/Real run - 5 lambs from LCF/mgf_Obj_1/Sarc_4-20pc_sarc_15B-17B_concat_all_the_line_dele.mgf
Protein: PREDICTED: barrier-to-autointegration factor [Ovis aries]
Accession: gi|426252060|ref|XP_004019736.1| **Parent m/z:** 675.349, 2+
Sequence: K.AYVVLGQFLVLK.K **Score:** 93.52288498895128

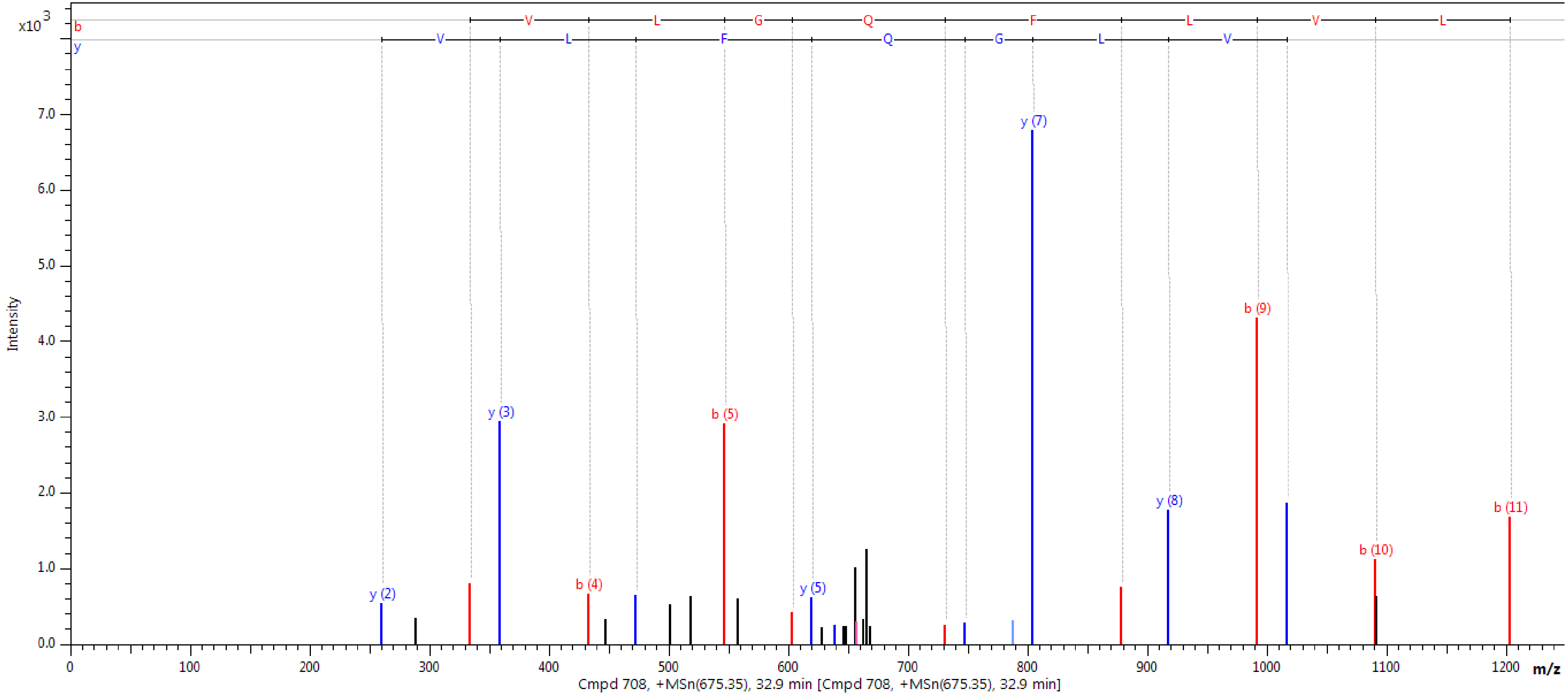

Spectrum Report

Source: M:/Documents/Lamb meat protein project/1. Characterisation of lamb skeletal proteome/Real run - 5 lambs from LCF/mgf_Obj_1/Sarc_4-20pc_sarc_15B-17B_concat_all_the_line_dele.mgf
Protein: PREDICTED: small ubiquitin-related modifier 3 [Ovis aries]
Accession: gi|426219527|ref|XP_004003973.1|
Parent m/z: 617.871, 2+
Sequence: K.VAGQDGSVVQFK.I
Score: 96.9837325959146

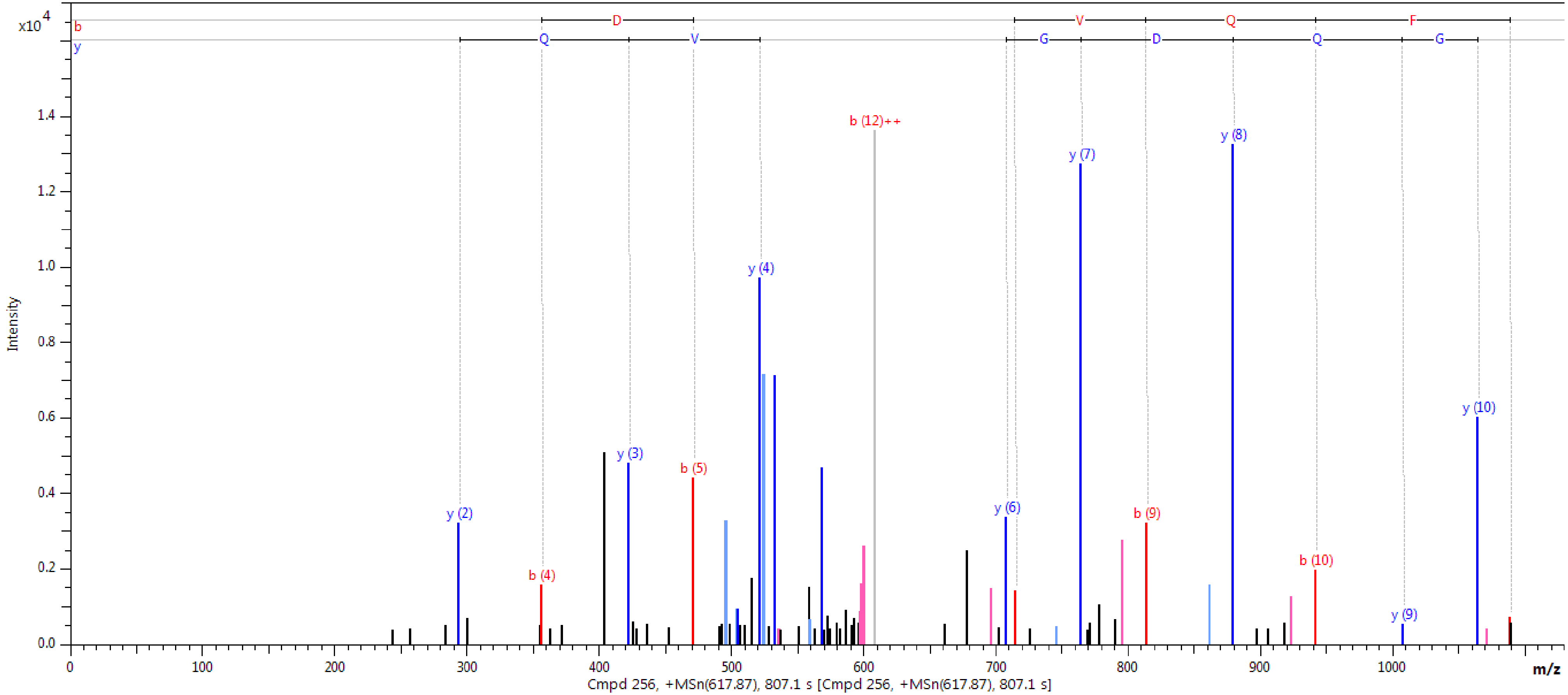

agresearch

Spectrum Report

Source: M:/Documents/Lamb meat protein project/1. Characterisation of lamb skeletal proteome/Real run - 5 lambs from LCF/mgf_Obj_1/Sarc_4-20pc_sarc_15B-17B_concat_all_the_line_dele.mgf
Protein: PREDICTED: myosin light chain 4 [Ovis aries]
Accession: gi|426238229|ref|XP_004013058.1|
Parent m/z: 691.89, 2+
Sequence: R.ALGQNPTNAEVLR.V
Score: 80.20007542760274

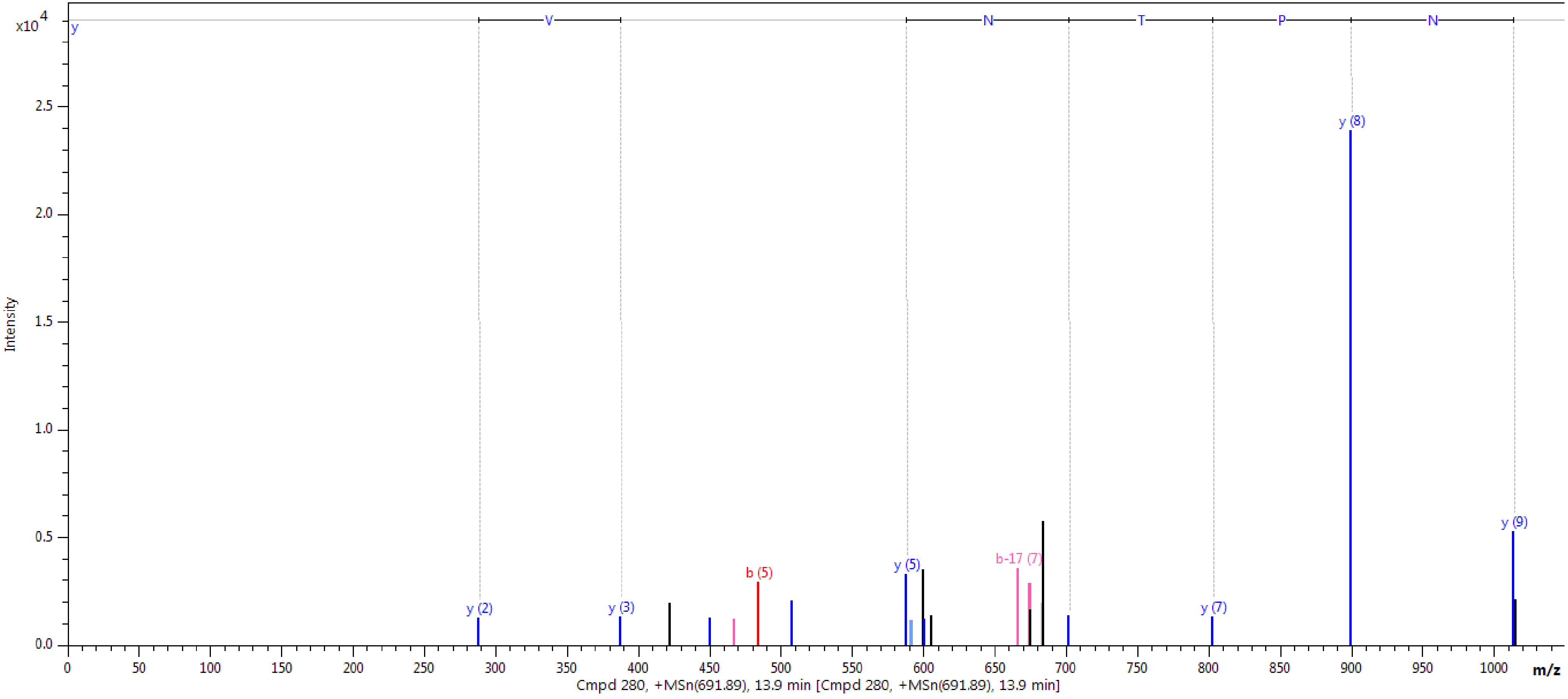

Spectrum Report

Source: M:/Documents/Lamb meat protein project/1. Characterisation of lamb skeletal proteome/Real run - 5 lambs from LCF/mgf_Obj_1/Sarc_4-20pc_sarc_15B-17B_concat_all_the_line_dele.mgf
Protein: PREDICTED: PDZ and LIM domain protein 7-like isoform 1 [Ovis aries]
Accession: gi|426229377|ref|XP_004008767.1| **Parent m/z:** 664.413, 2+
Sequence: K.VVLEGPAPWGFR.L **Score:** 72.87838733396454

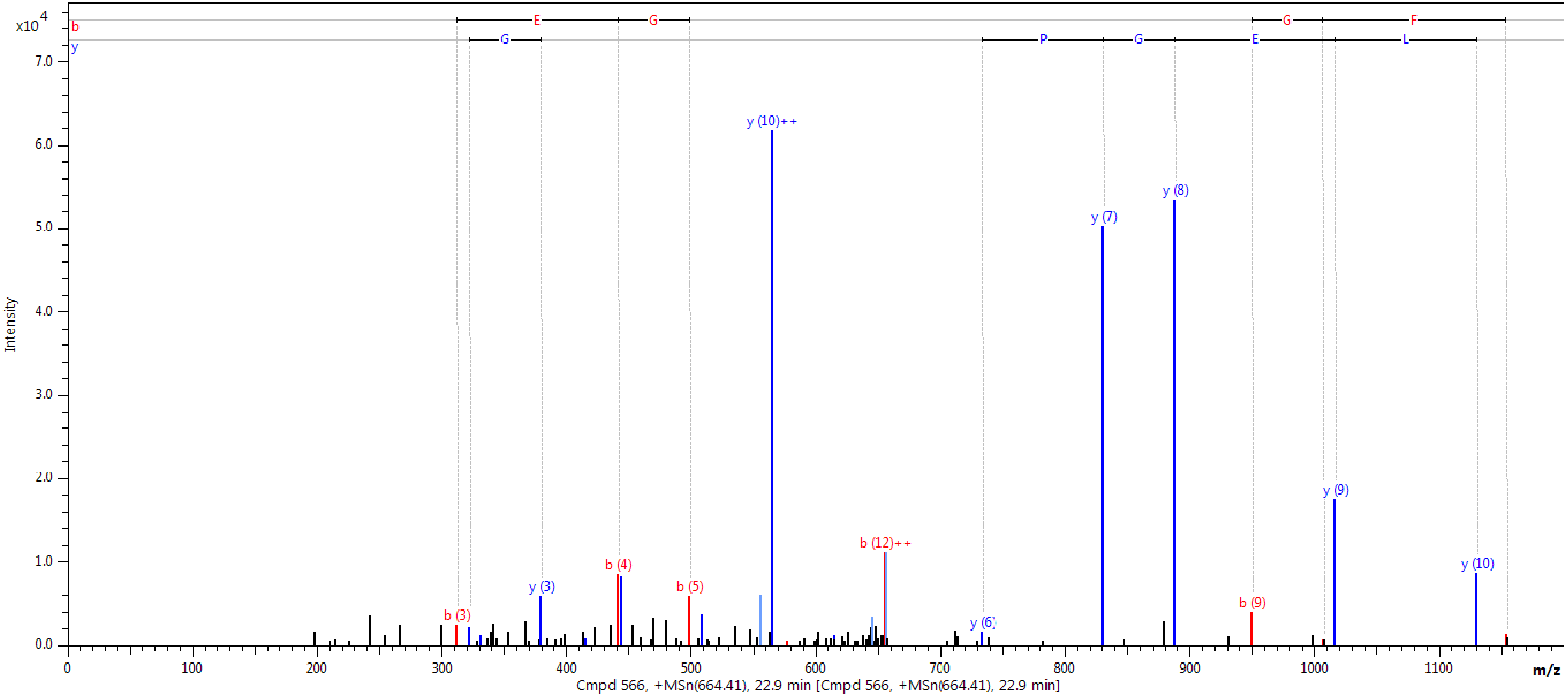

Spectrum Report

Source: M:/Documents/Lamb meat protein project/1. Characterisation of lamb skeletal proteome/Real run - 5 lambs from LCF/ mgf_Obj_1/Sarc_4-20pc_sarc_15B-17B_concat_all_the_line_dele.mgf
Protein: PREDICTED: isocitrate dehydrogenase [NADP], mitochondrial, partial [Ovis aries]
Accession: gi|426248712|ref|XP_004018103.1|
Parent m/z: 678.345, 2+
Sequence: K.TIEAEAAHGTVTR.H
Score: 59.200846931719894

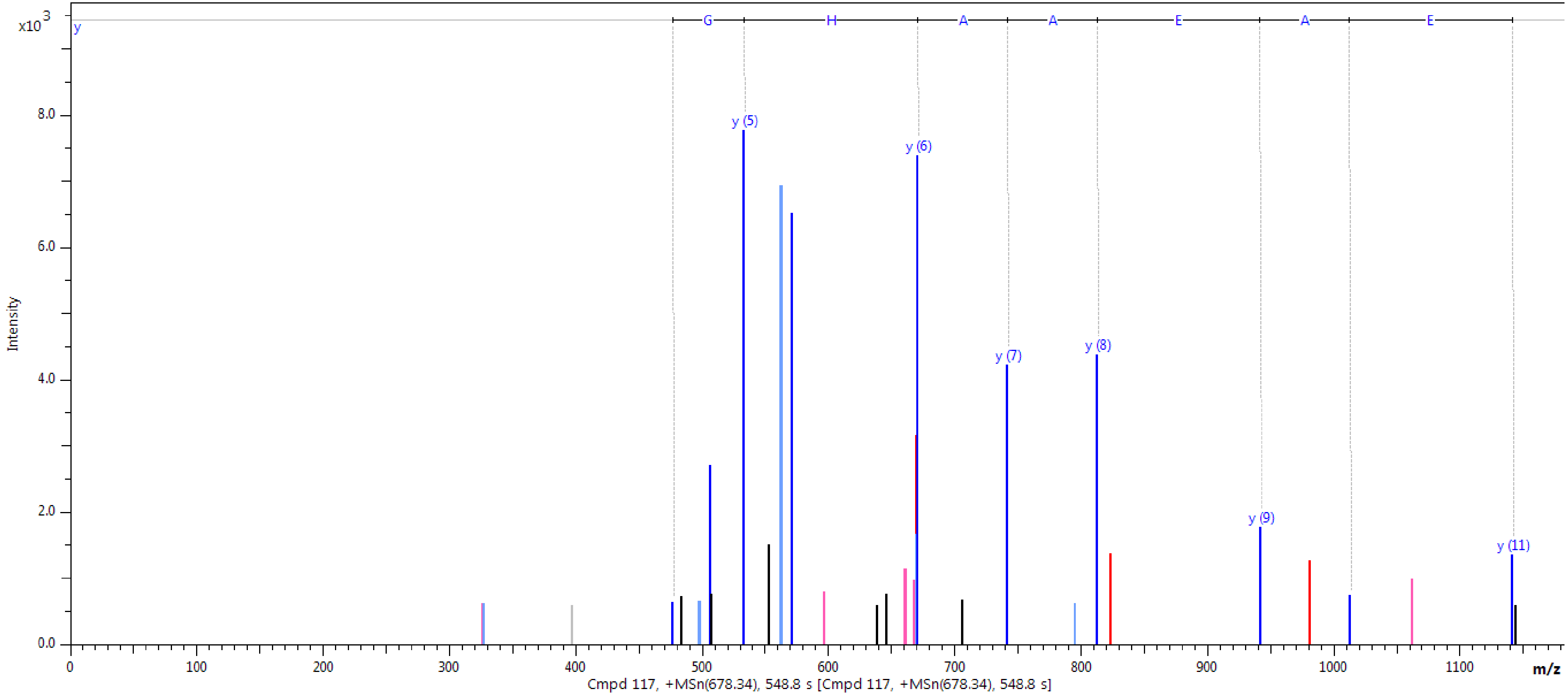

Spectrum Report

Source: M:/Documents/Lamb meat protein project/1. Characterisation of lamb skeletal proteome/Real run - 5 lambs from LCF/mgf_Obj_1/Sarc_4-20pc_sarc_15B-17B_concat_all_the_line_dele.mgf
Protein: PREDICTED: electron transfer flavoprotein subunit alpha, mitochondrial isoform 2 [Ovis aries]
Accession: gi|426248248|ref|XP_004017876.1|
Parent m/z: 684.437, 2+
Sequence: K.LDVAPISDIIAIK.S
Score: 112.20819967352756

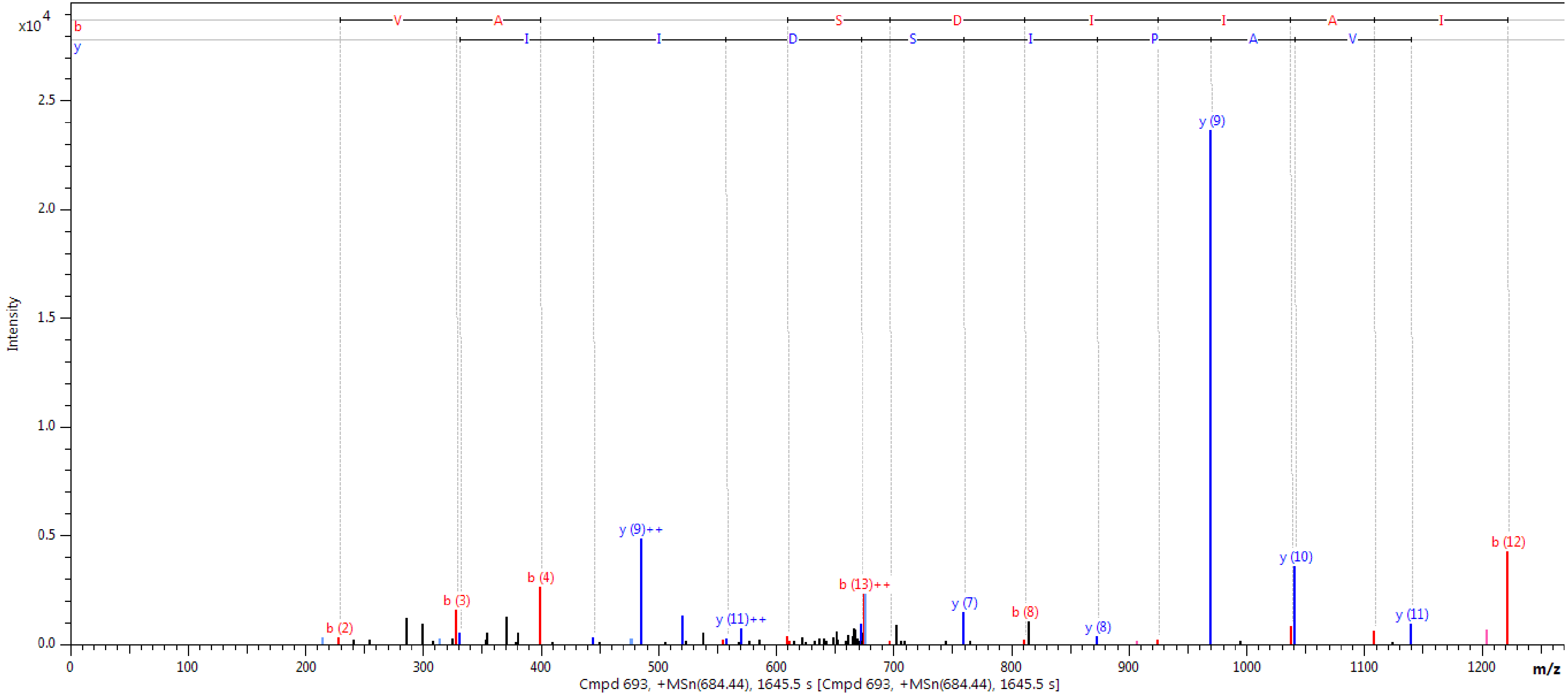

Spectrum Report

Source: M:/Documents/Lamb meat protein project/1. Characterisation of lamb skeletal proteome/Real run - 5 lambs from LCF/ mgf_Obj_1/Sarc_4-20pc_sarc_15B-17B_concat_all_the_line_dele.mgf
Protein: ceruloplasmin [Ovis aries]
Accession: gi|5281319|gb|AAD41477.1|AF134814_1
Sequence: R.EYTDASFSNQK.E
Parent m/z: 645.336, 2+
Score: 105.3769935487831

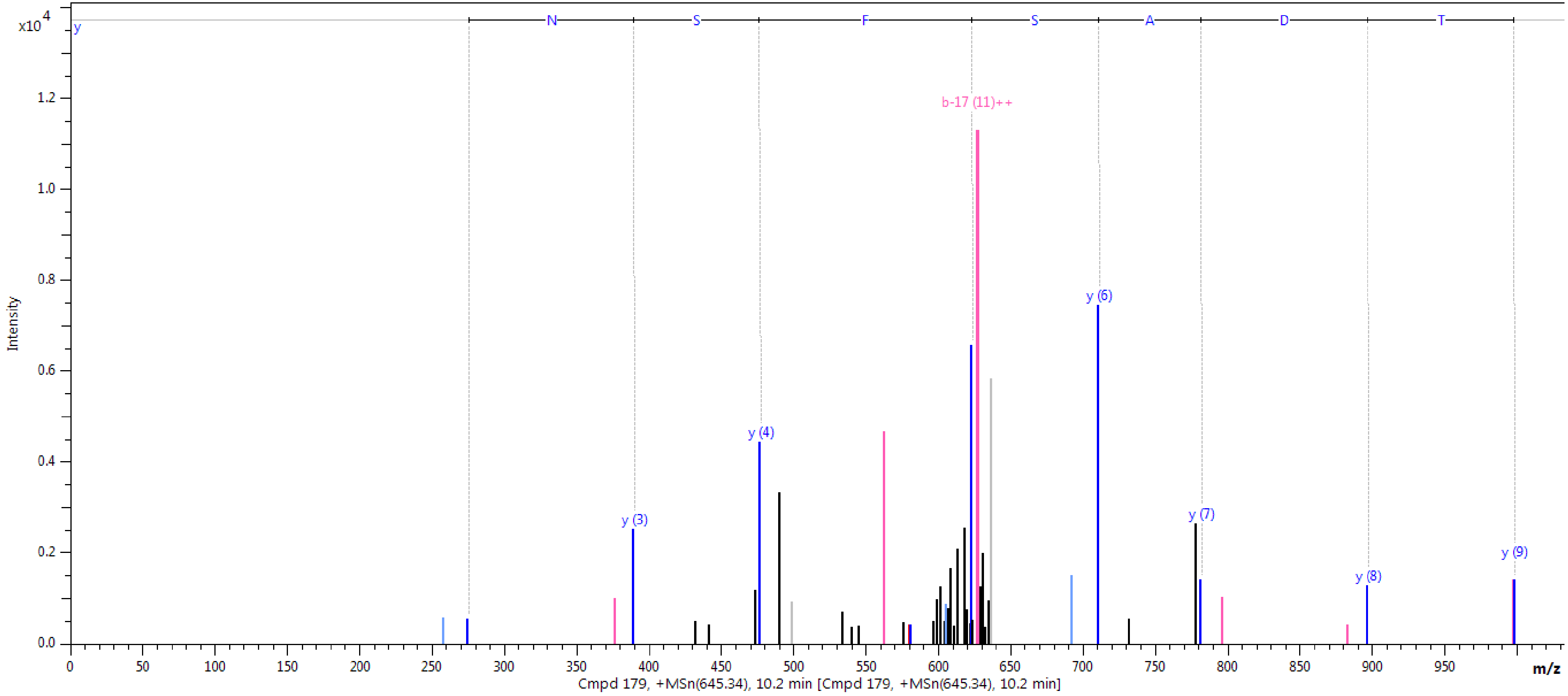

Spectrum Report

Source: M:/Documents/Lamb meat protein project/1. Characterisation of lamb skeletal proteome/Real run - 5 lambs from LCF/mgf_Obj_1/Sarc_4-20pc_sarc_15B-17B_concat_all_the_line_dele.mgf
Protein: PREDICTED: glycogen [starch] synthase, liver [Ovis aries]
Accession: gi|426225378|ref|XP_004006843.1| **Parent m/z:** 589.916, 2+
Sequence: K.VGGIYTVIQTK.A **Score:** 111.0308320338846

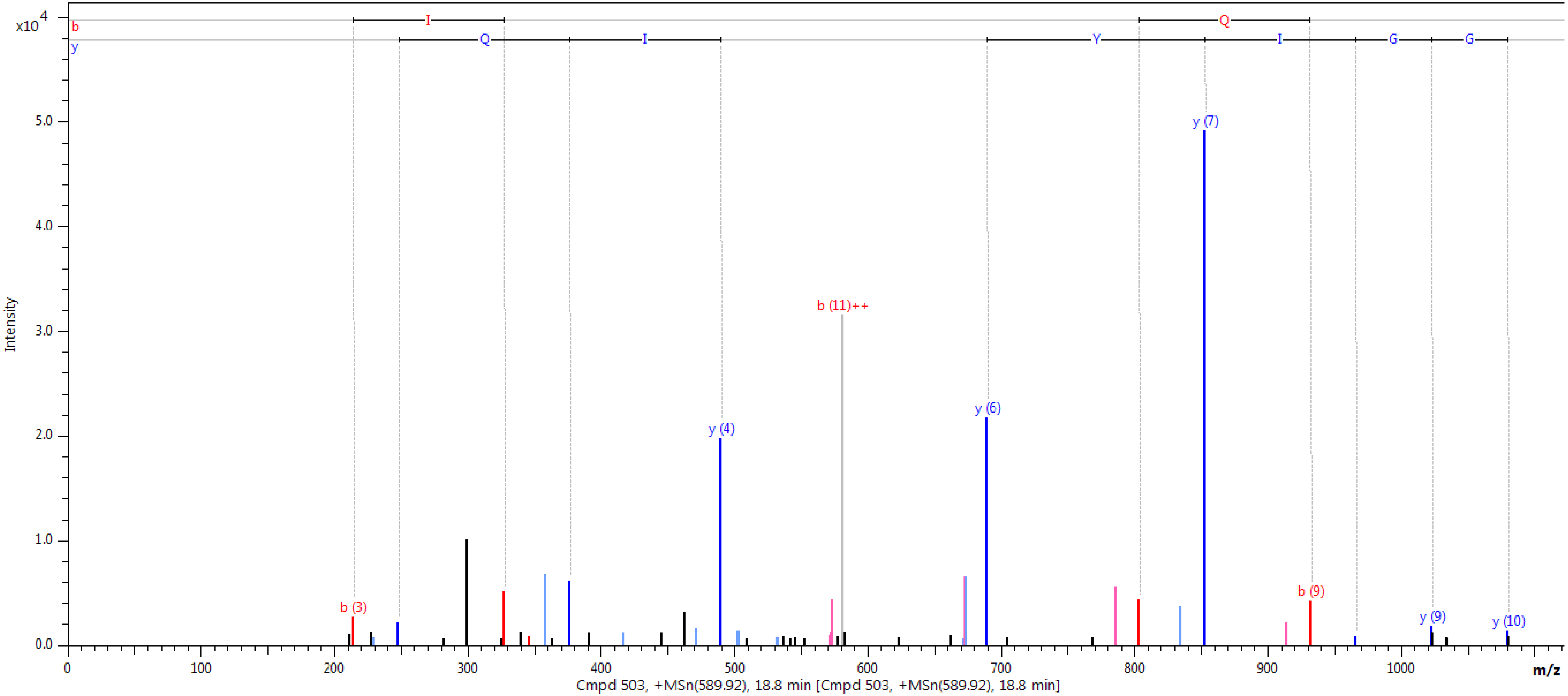

Spectrum Report

Source: M:/Documents/Lamb meat protein project/1. Characterisation of lamb skeletal proteome/Real run - 5 lambs from LCF/mgf_Obj_1/Sarc_4-20pc_sarc_15B-17B_concat_all_the_line_dele.mgf
Protein: PREDICTED: cadherin-13 isoform 2 [Ovis aries]
Accession: gi|426242244|ref|XP_004014984.1|
Sequence: R.INENTGSVSVTR.N
Parent m/z: 638.846, 2+
Score: 104.19913645680639

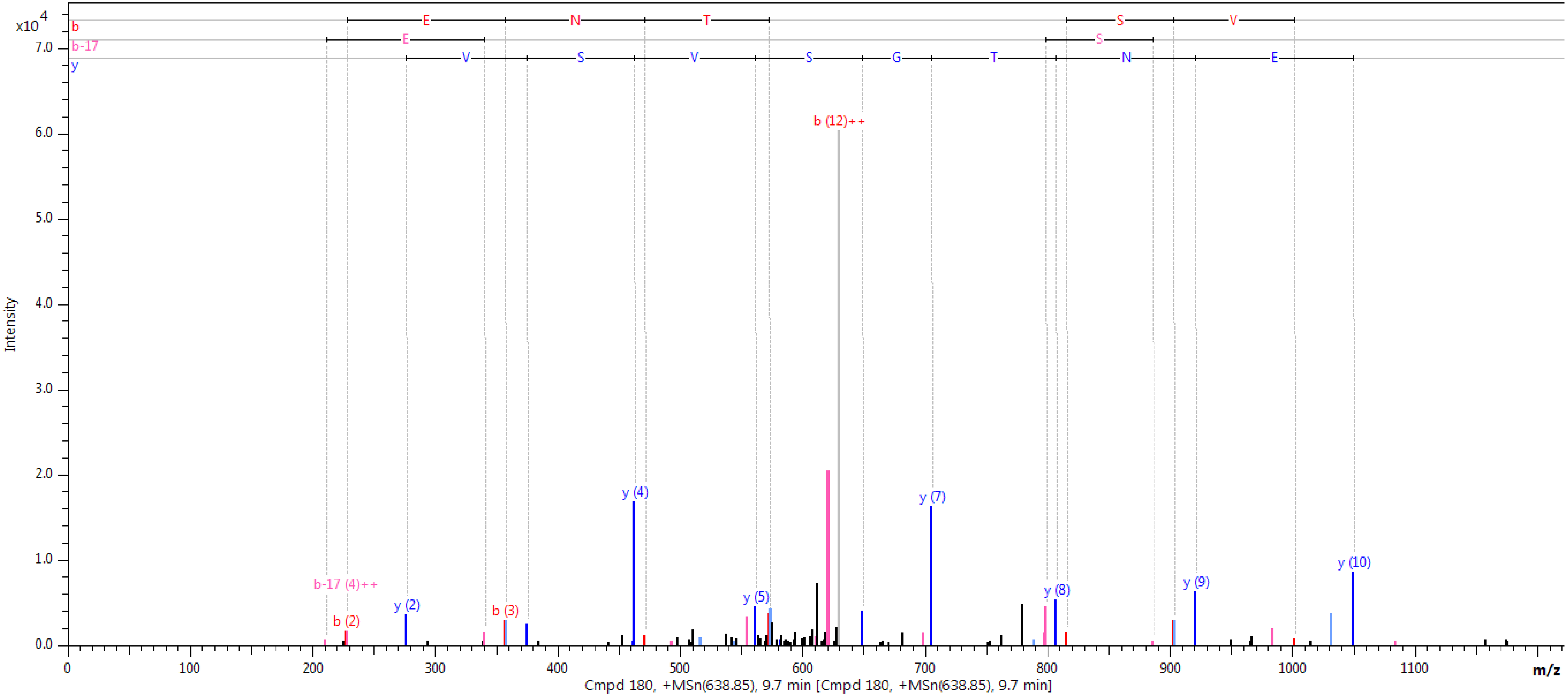

Spectrum Report

Source: M:/Documents/Lamb meat protein project/1. Characterisation of lamb skeletal proteome/Real run - 5 lambs from LCF/mgf_Obj_1/Sarc_4-20pc_sarc_15B-17B_concat_all_the_line_dele.mgf
Protein: PREDICTED: nidogen-1 [Ovis aries]
Accession: gi|426256054|ref|XP_004021660.1|
Sequence: R.VLFETDLVNPR.G
Parent m/z: 651.905, 2+
Score: 55.43861803449708

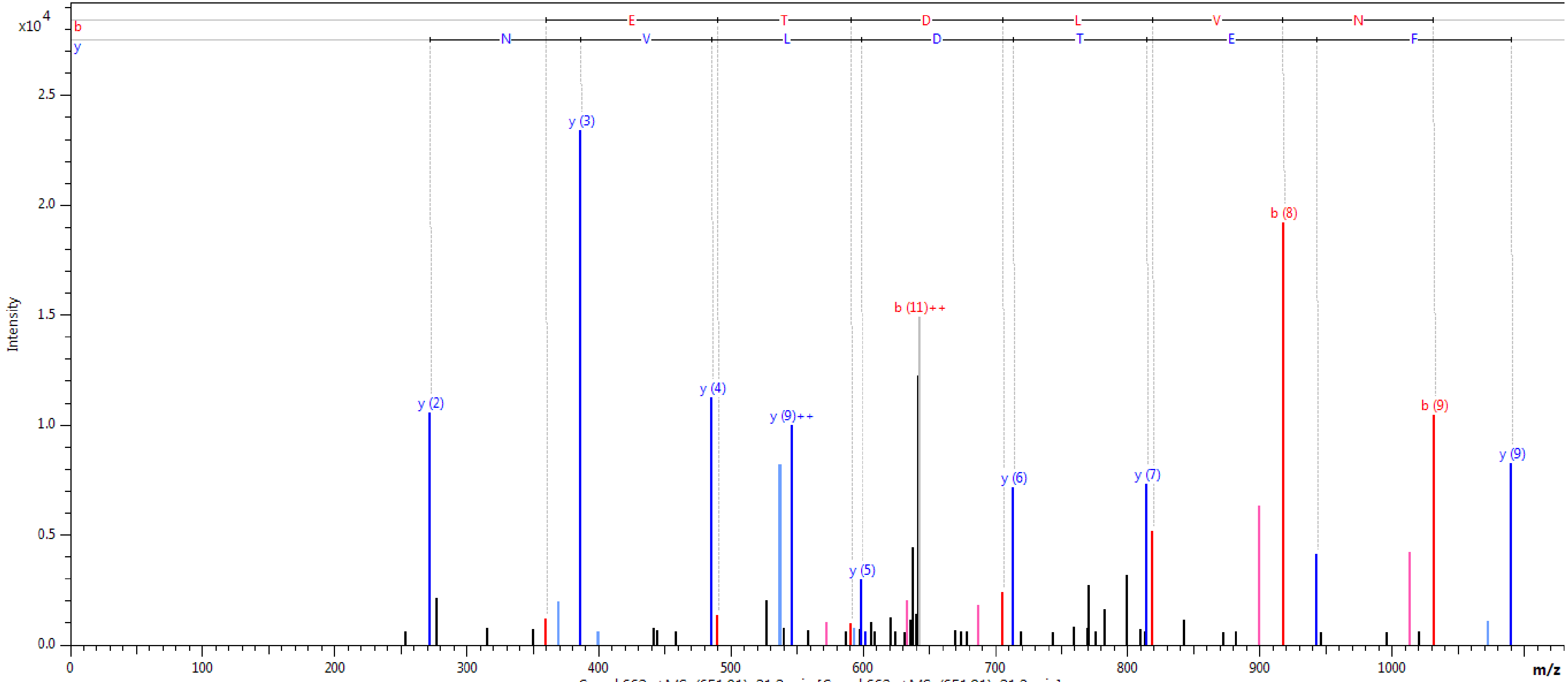

Cmpd 663, +MSn(651.91), 21.2 min [Cmpd 663, +MSn(651.91), 21.2 min]

Spectrum Report

Source: M:/Documents/Lamb meat protein project/1. Characterisation of lamb skeletal proteome/Real run - 5 lambs from LCF/mgf_Obj_1/Sarc_4-20pc_sarc_15B-17B_concat_all_the_line_dele.mgf

Protein: PREDICTED: phosphoglycolate phosphatase, partial [Ovis aries]

Accession: gi|426255033|ref|XP_004021170.1|

Parent m/z: 630.904, 2+

Sequence: K.TILTLTGVSSLR.D

Score: 102.62084929764553

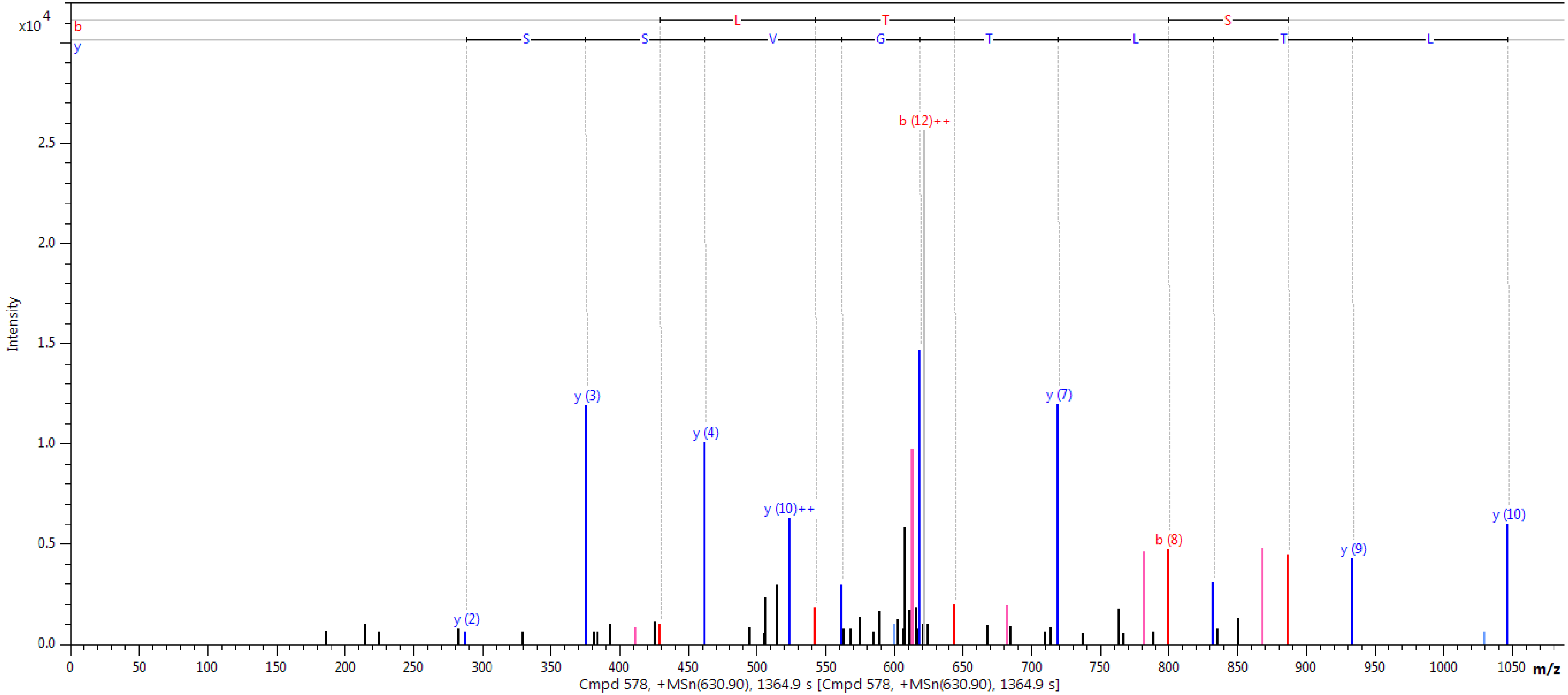

agresearch

Spectrum Report

Source: M:/Documents/Lamb meat protein project/1. Characterisation of lamb skeletal proteome/Real run - 5 lambs from LCF/mgf_Obj_1/Sarc_4-20pc_sarc_15B-17B_concat_all_the_line_dele.mgf

Protein: PREDICTED: glyoxalase domain-containing protein 4 [Ovis aries]

Accession: gi|426237266|ref|XP_004012582.1| **Parent m/z:** 677.417, 2+

Sequence: K.ILTPLVSLDTPGK.A **Score:** 71.59126410056308

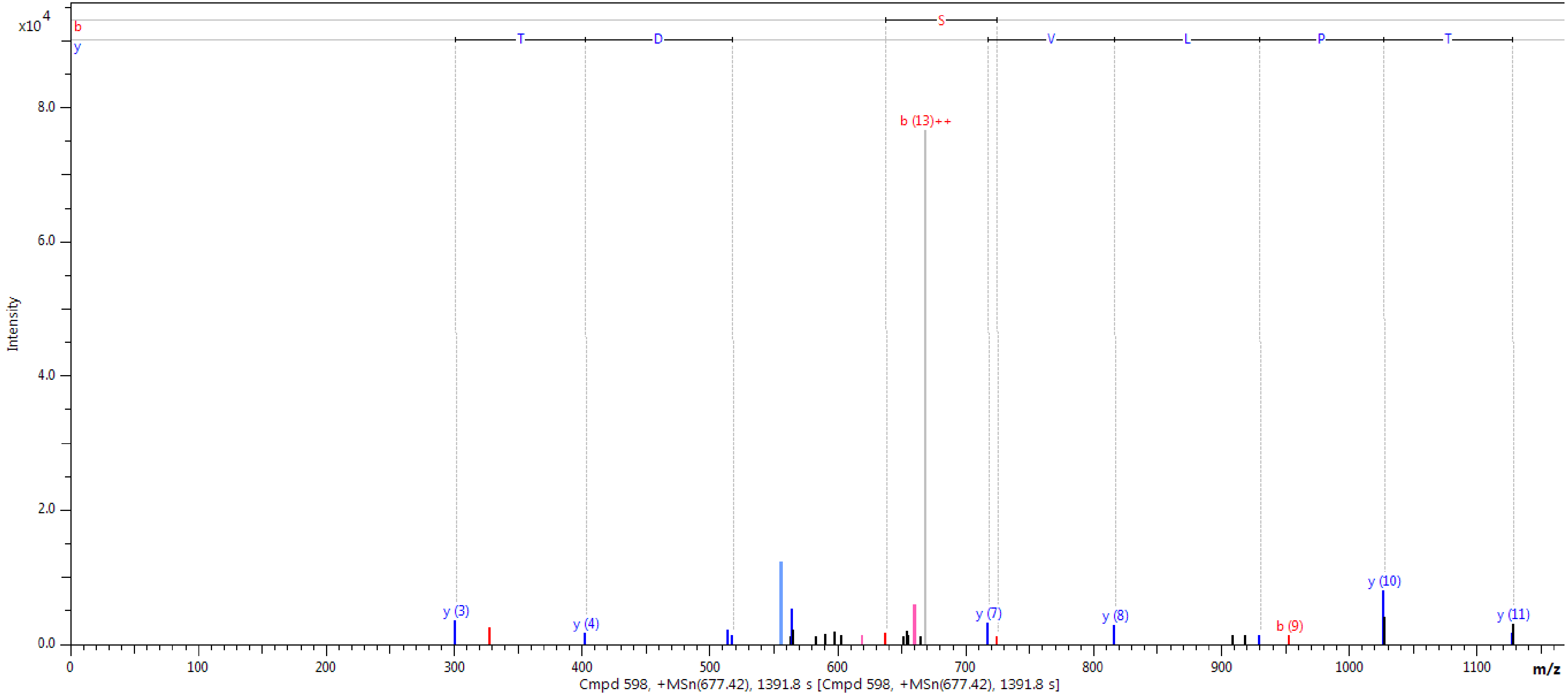

agresearch

Spectrum Report

Source: M:/Documents/Lamb meat protein project/1. Characterisation of lamb skeletal proteome/Real run - 5 lambs from LCF/mgf_Obj_1/Sarc_4-20pc_sarc_15B-17B_concat_all_the_line_dele.mgf
Protein: PREDICTED: proteasome subunit alpha type-7-like [Ovis aries]
Accession: gi|426253661|ref|XP_004020511.1| **Parent m/z:** 550.91, 2+
Sequence: R.ALLEVVQSGGK.N **Score:** 51.06217716210025

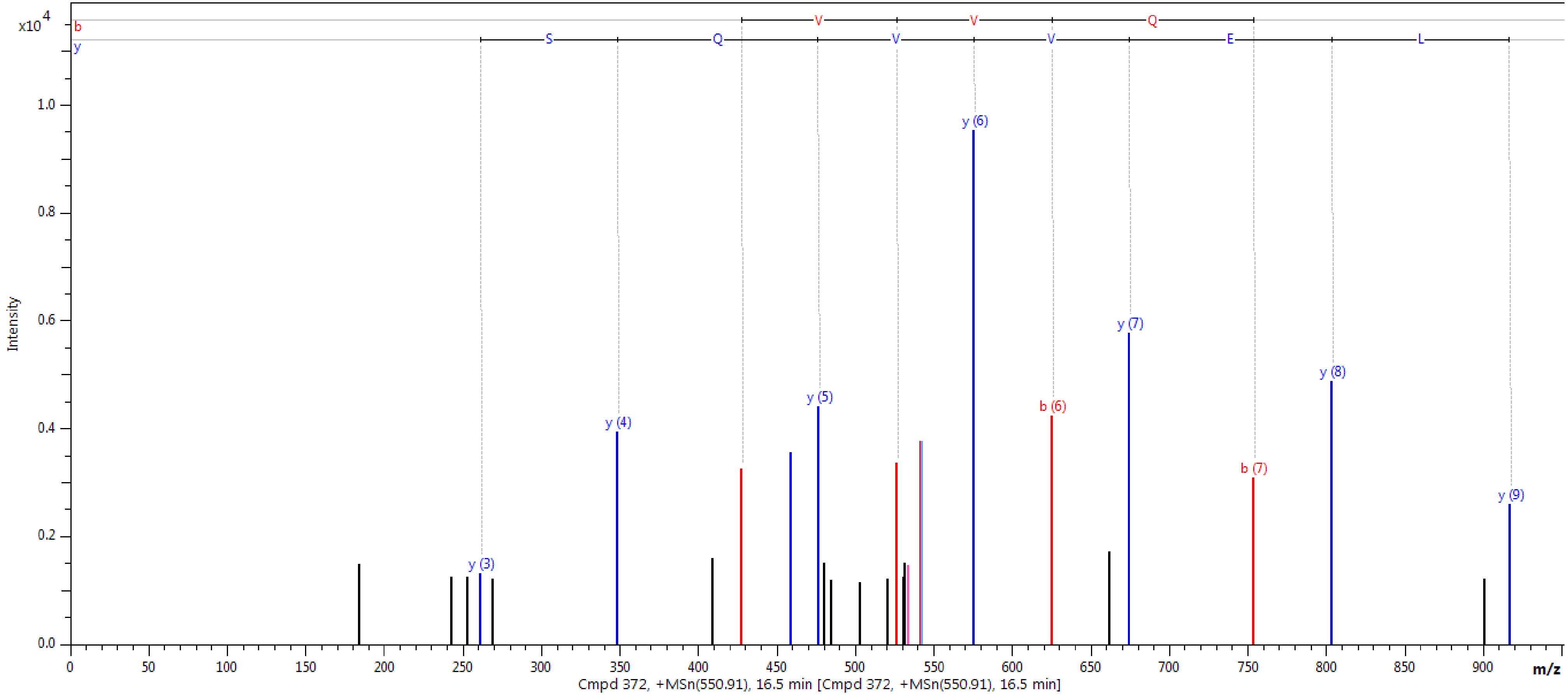

Spectrum Report

Source: M:/Documents/Lamb meat protein project/1. Characterisation of lamb skeletal proteome/Real run - 5 lambs from LCF/ mgf_Obj_1/Sarc_4-20pc_sarc_15B-17B_concat_all_the_line_dele.mgf
Protein: PREDICTED: LOW QUALITY PROTEIN: dual specificity protein phosphatase 3 [Ovis aries]
Accession: gi|426239075|ref|XP_004023423.1| **Parent m/z:** 645.916, 2+
Sequence: K.AADFIDQALAQK.N **Score:** 49.67627059758111

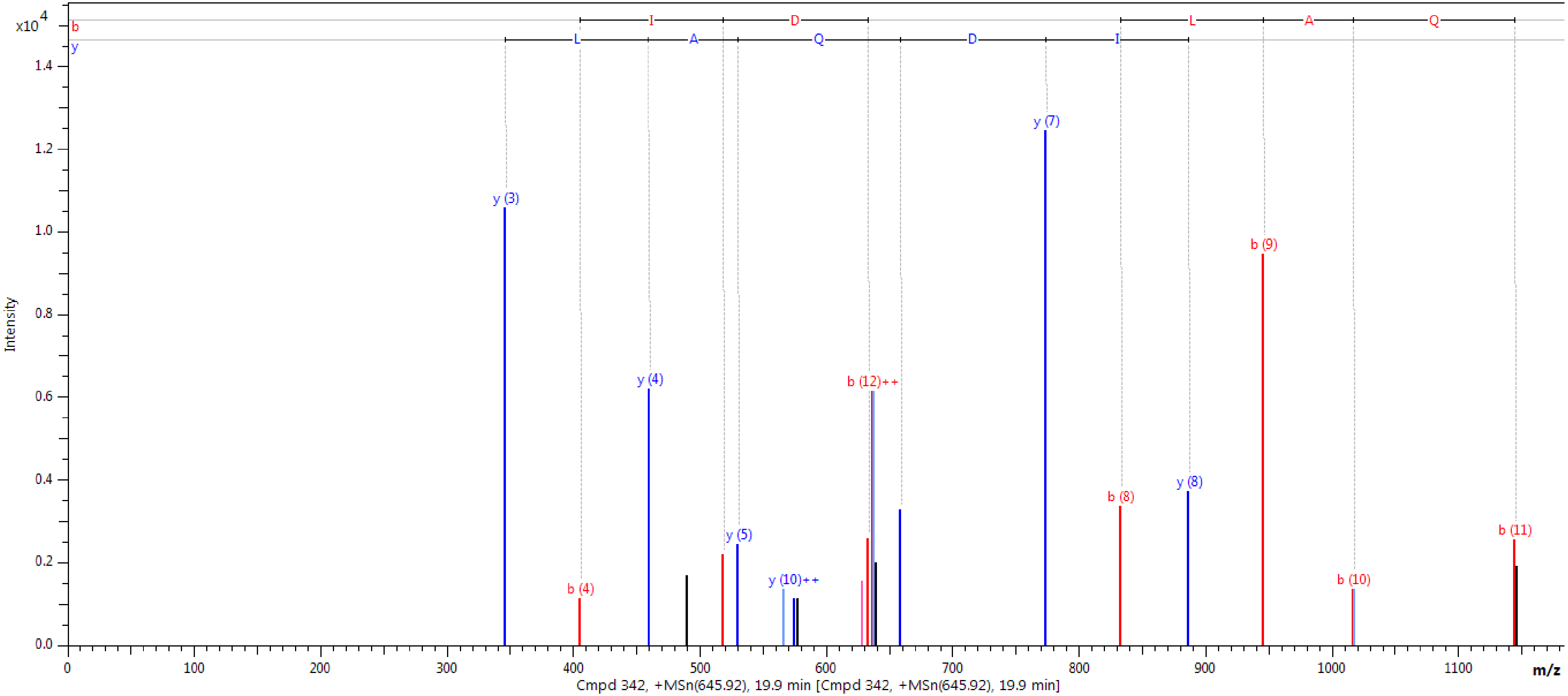

Spectrum Report

Source: M:/Documents/Lamb meat protein project/1. Characterisation of lamb skeletal proteome/Real run - 5 lambs from LCF/mgf_Obj_1/Sarc_4-20pc_sarc_15B-17B_concat_all_the_line_dele.mgf
Protein: PREDICTED: UPF0366 protein C11orf67 homolog [Ovis aries]
Accession: gi|426251545|ref|XP_004019482.1|
Parent m/z: 618.348, 2+
Sequence: K.EYNALATQGIR.V
Score: 98.90897514404101

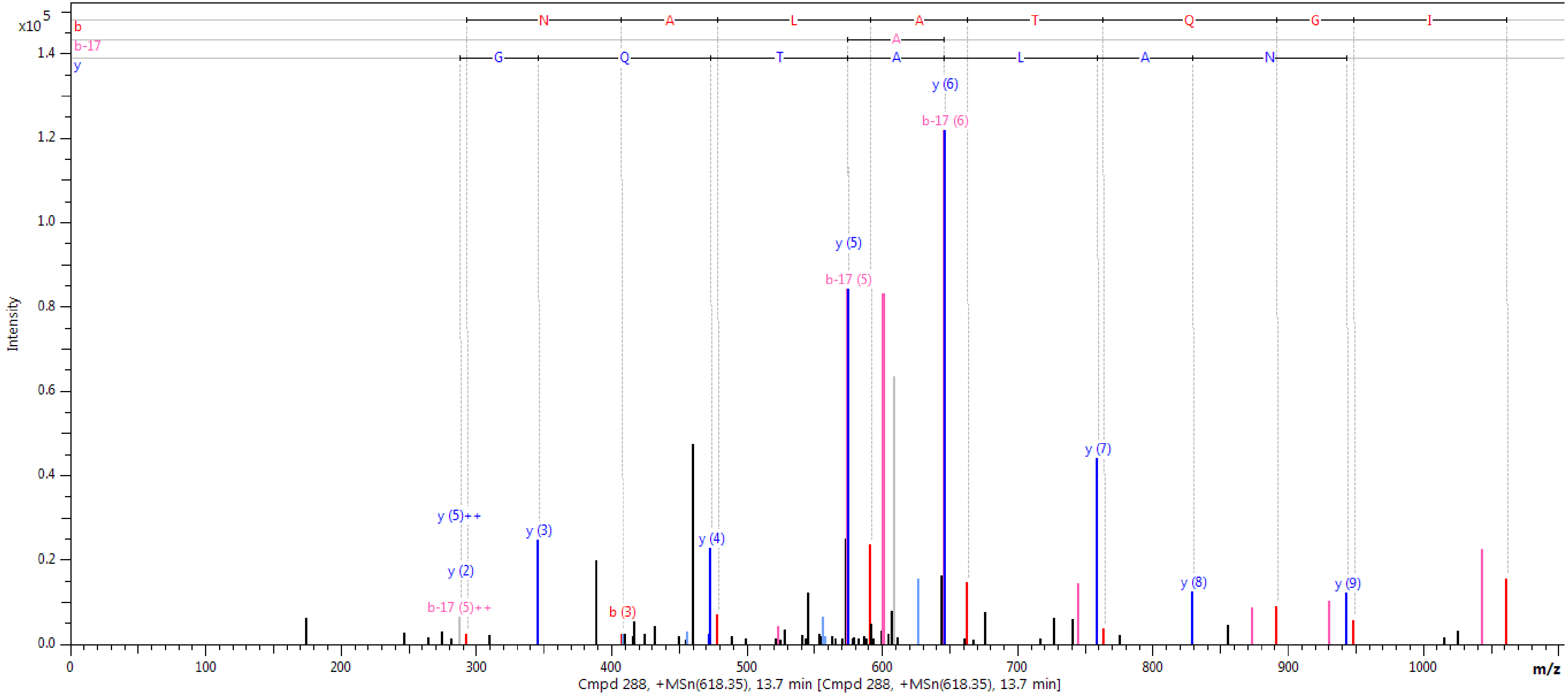

Source: M:/Documents/Lamb meat protein project/1. Characterisation of lamb skeletal proteome/Real run - 5 lambs from LCF/mgf_Obj_1/Sarc_4-20pc_sarc_15B-17B_concat_all_the_line_dele.mgf
Protein: PREDICTED: proteasome subunit beta type-5 [Ovis aries]
Accession: gi|426232754|ref|XP_004010385.1|
Parent m/z: 640.862, 2+
Sequence: R.ATAGAYIASQTVK.K
Score: 101.27693936064071

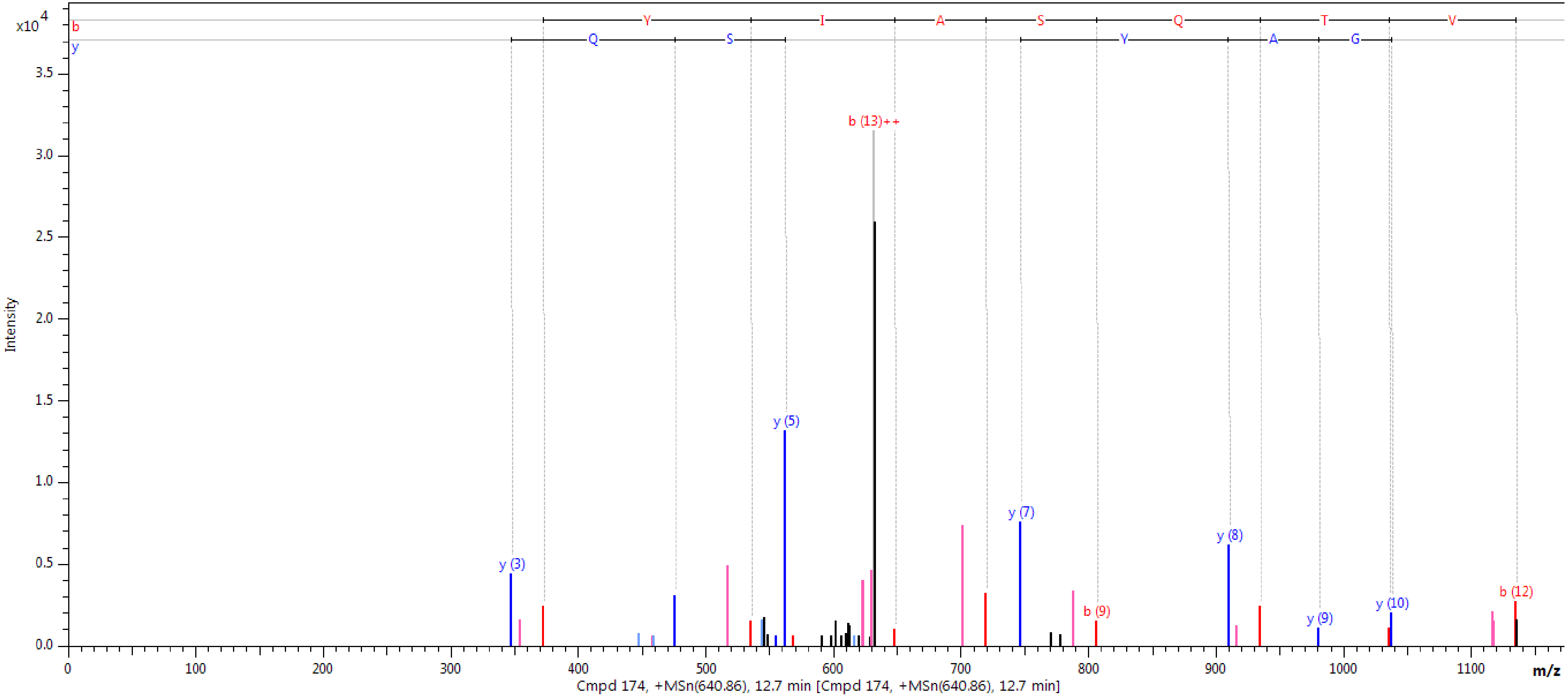

Spectrum Report

Source: M:/Documents/Lamb meat protein project/1. Characterisation of lamb skeletal proteome/Real run - 5 lambs from LCF/mgf_Obj_1/Sarc_4-20pc_sarc_15B-17B_concat_all_the_line_dele.mgf
Protein: PREDICTED: thioredoxin, mitochondrial [Ovis aries]
Accession: gi|426225227|ref|XP_004006768.1|
Sequence: S.TTFNIQDGPDFQDR.V
Parent m/z: 827.404, 2+
Score: 67.68790452413583

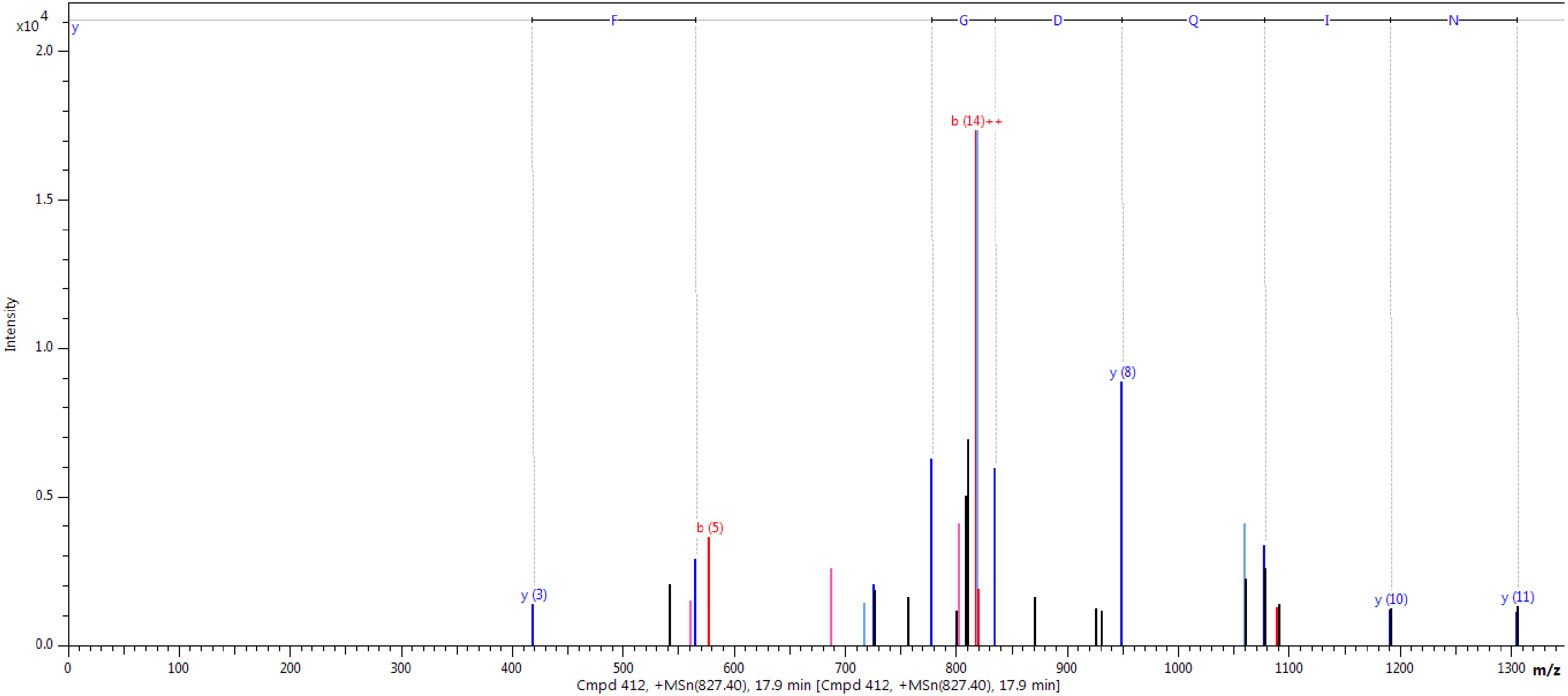

agresearch

Spectrum Report

Source: M:/Documents/Lamb meat protein project/1. Characterisation of lamb skeletal proteome/Real run - 5 lambs from LCF/ mgf_Obj_1/Sarc_4-20pc_sarc_15B-17B_concat_all_the_line_dele.mgf
Protein: beta-casein, partial [Ovis aries]
Accession: gi|499144288|gb|AGL76768.1|
Sequence: L.YQEPVIGPVRGPFPIL.-
Parent m/z: 891.555, 2+
Score: 88.32659030821088

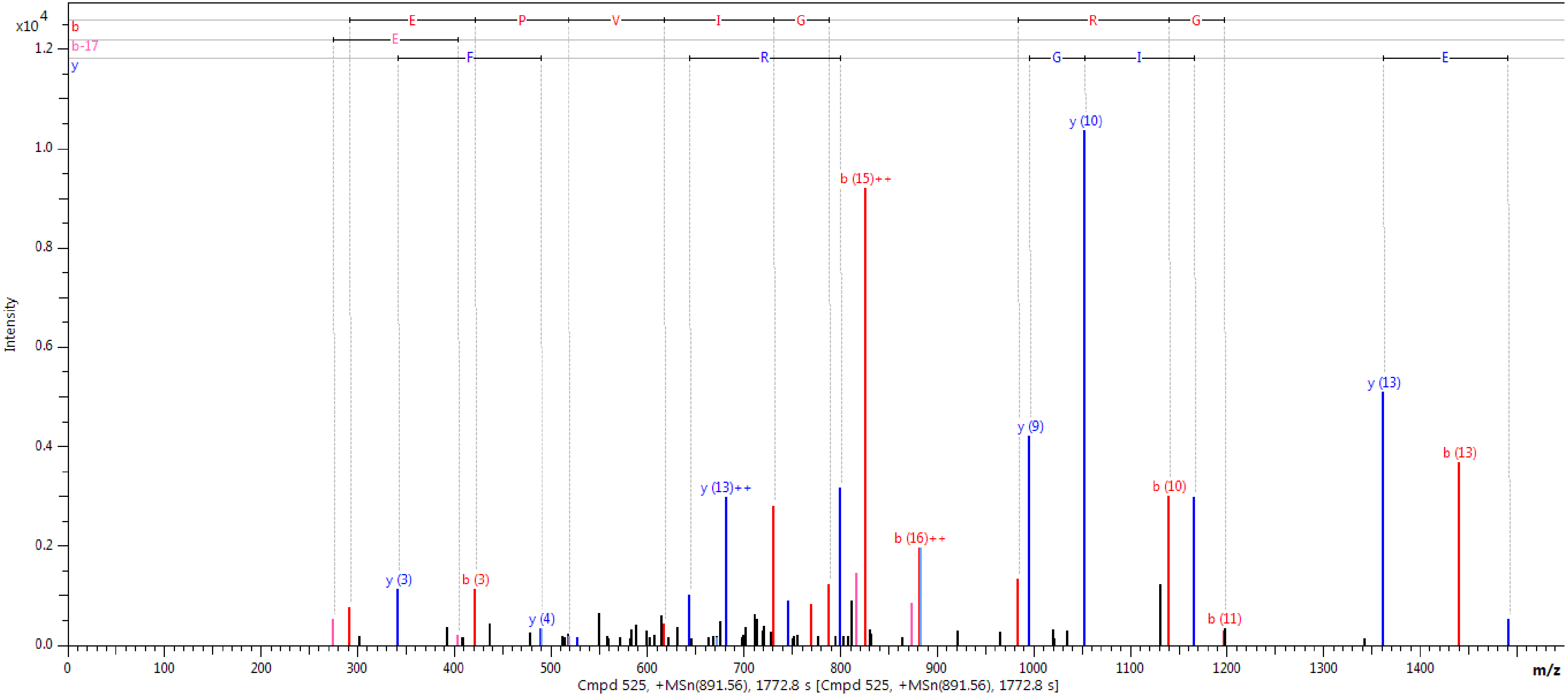

Spectrum Report

Source: M:/Documents/Lamb meat protein project/1. Characterisation of lamb skeletal proteome/Real run - 5 lambs from LCF/mgf_Obj_1/Sarc_4-20pc_sarc_15B-17B_concat_all_the_line_dele.mgf
Protein: PREDICTED: acyl carrier protein, mitochondrial-like, partial [Ovis aries]
Accession: gi|426258741|ref|XP_004022966.1|
Parent m/z: 756.322, 2+
Sequence: Y.SDAPPLTLEGIKDR.V
Score: 37.0993866364551

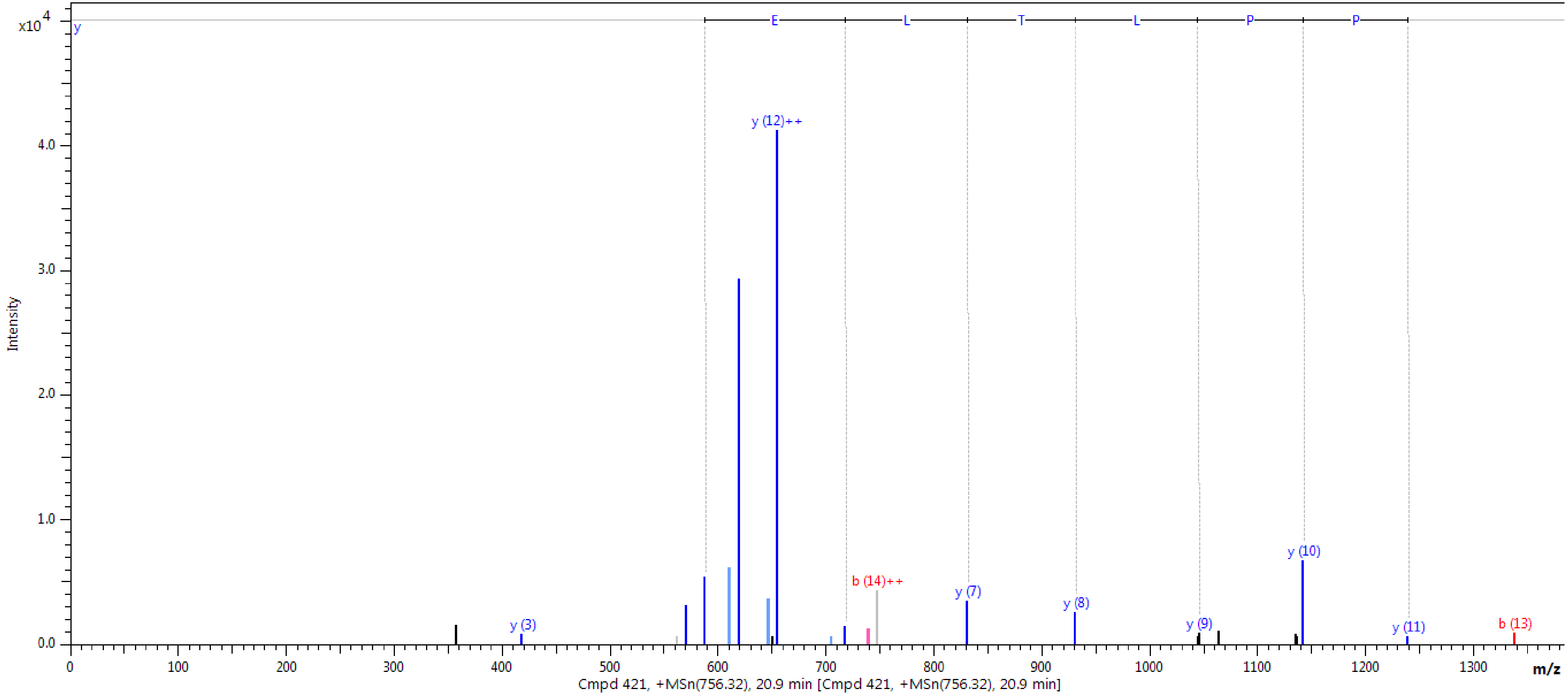

Spectrum Report

Source: M:/Documents/Lamb meat protein project/1. Characterisation of lamb skeletal proteome/Real run - 5 lambs from LCF/ mgf_Obj_1/Sarc_4-20pc_sarc_15B-17B_concat_all_the_line_dele.mgf
Protein: PREDICTED: protein CutA [Ovis aries]
Accession: gi|426250158|ref|XP_004018805.1|
Sequence: K.TQSSLVPALTDFVR.S
Parent m/z: 767.454, 2+
Score: 40.295959345790976

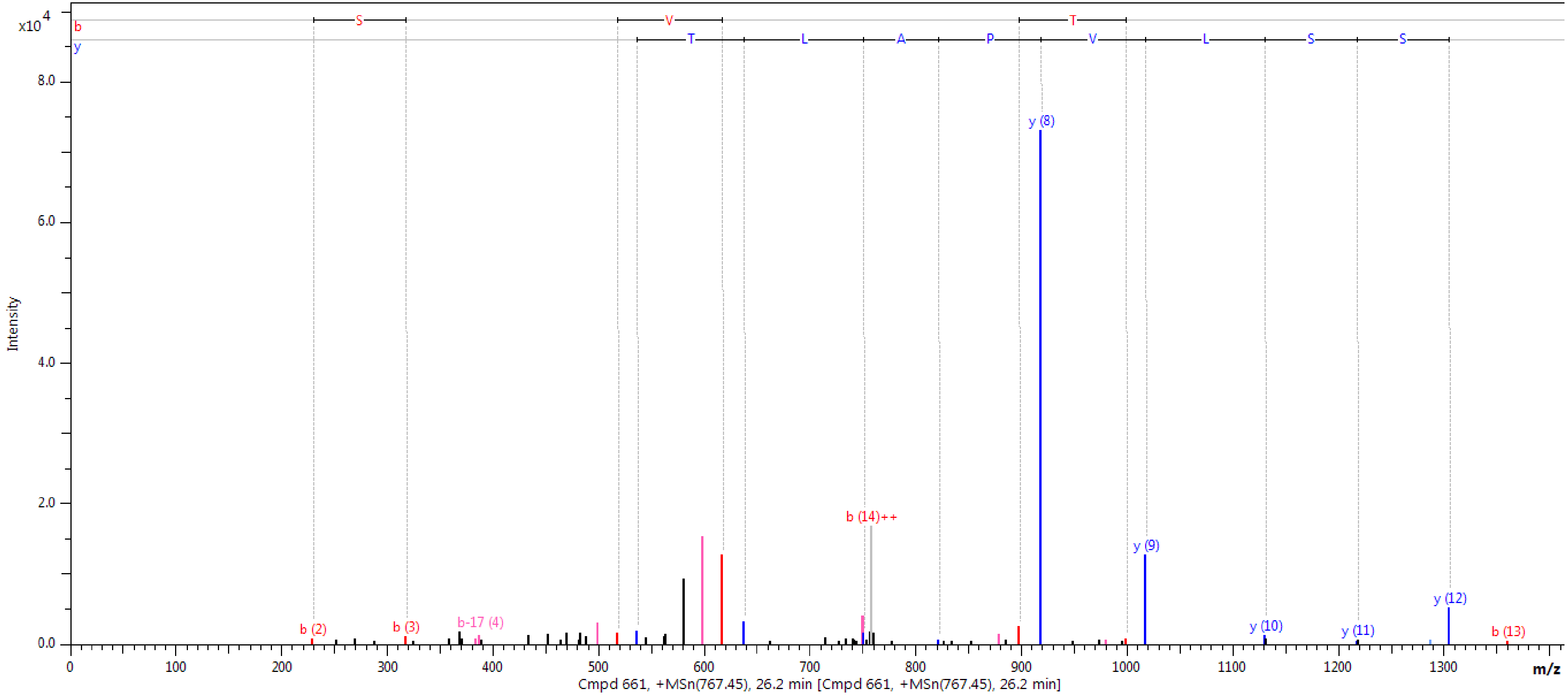

Spectrum Report

Source: M:/Documents/Lamb meat protein project/1. Characterisation of lamb skeletal proteome/Real run - 5 lambs from LCF/ mgf_Obj_1/Sarc_4-20pc_sarc_15B-17B_concat_all_the_line_dele.mgf
Protein: peroxiredoxin 6 [Ovis aries]
Accession: gi|469664895|gb|AGH70289.1|
Sequence: K.LSILYPATTGR.N
Parent m/z: 596.385, 2+
Score: 26.54547848624176

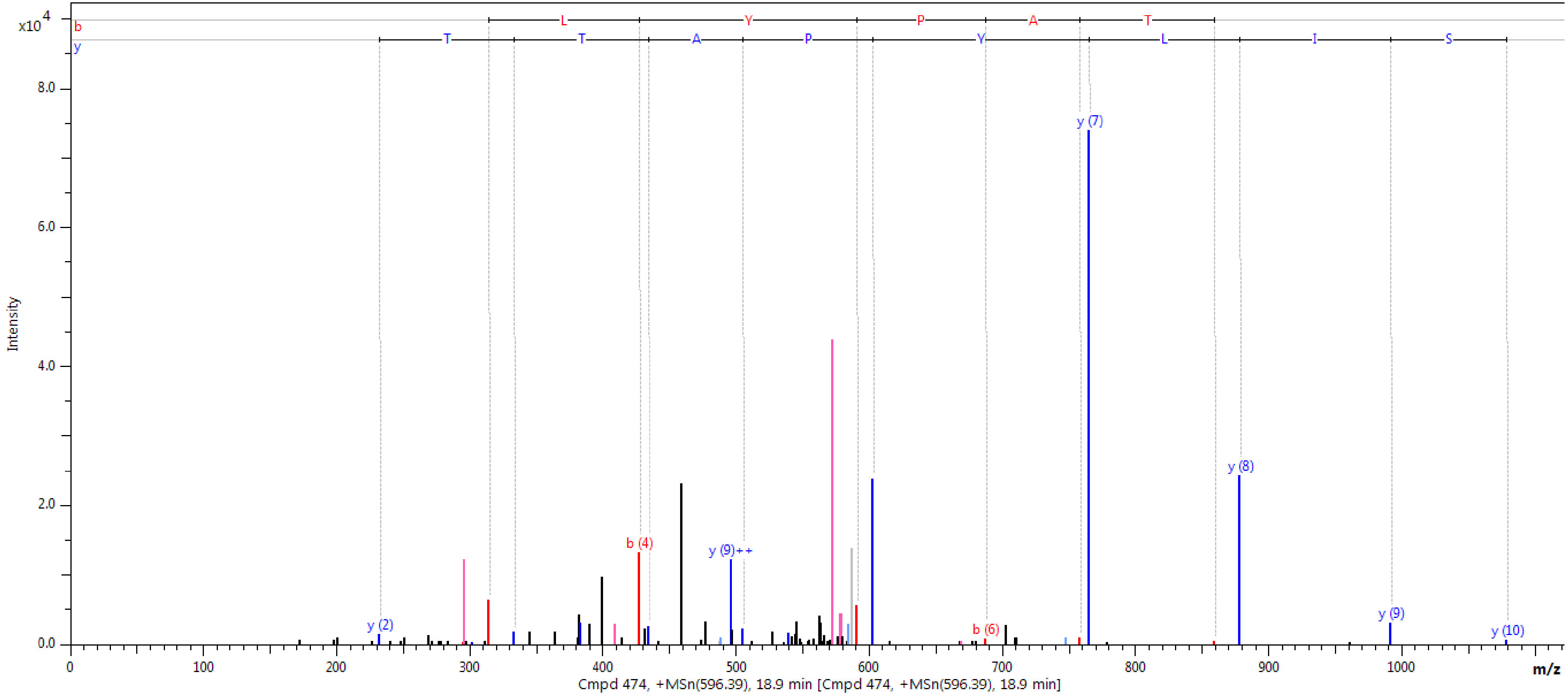

Source: M:/Documents/Lamb meat protein project/1. Characterisation of lamb skeletal proteome/Real run - 5 lambs from LCF/ mgf_Obj_1/Sarc_4-20pc_sarc_15B-17B_concat_all_the_line_dele.mgf
Protein: uterine myometrial annexin 2 [Ovis aries]
Accession: gi|86279630|gb|ABC94470.1|
Sequence: K.TPAQYDASELK.A
Parent m/z: 611.844, 2+
Score: 37.51752012738619

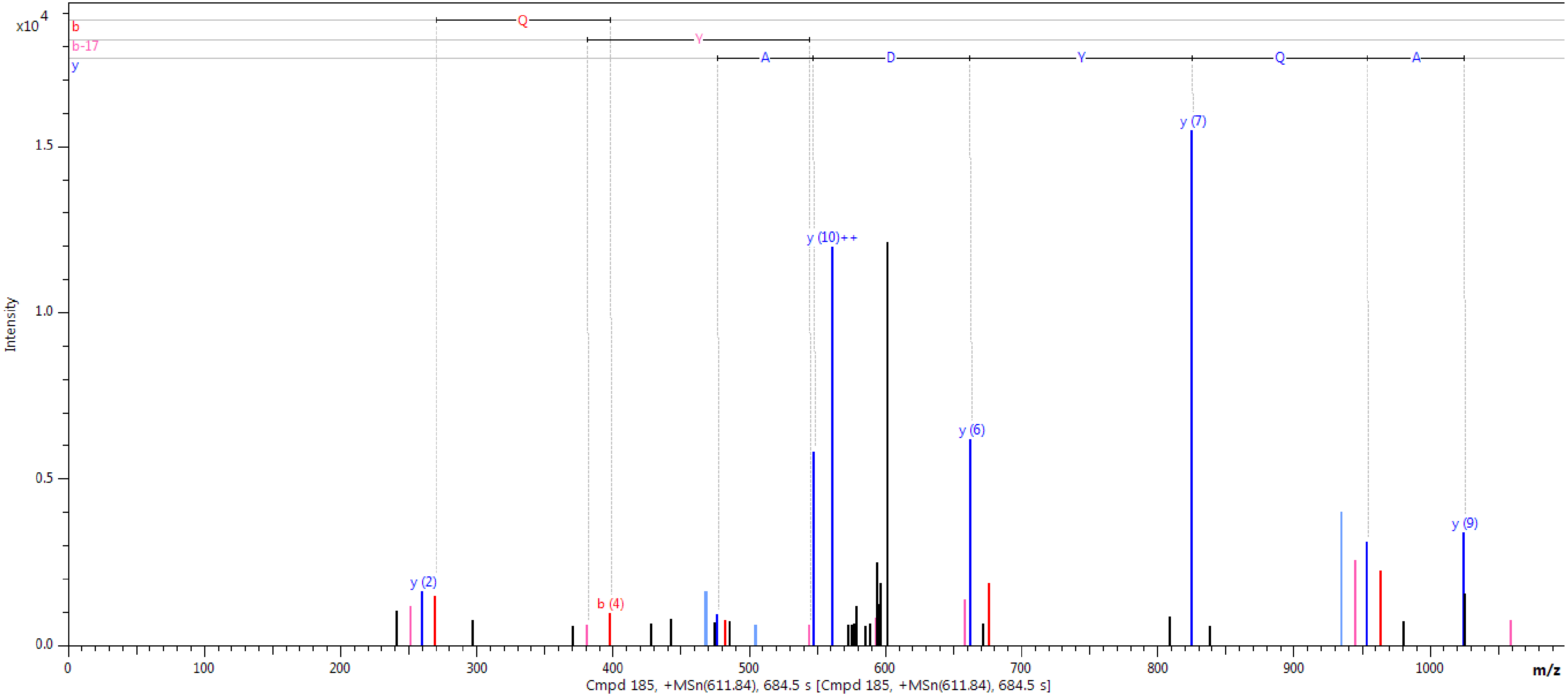

agresearch

Spectrum Report

Source: M:/Documents/Lamb meat protein project/1. Characterisation of lamb skeletal proteome/Real run - 5 lambs from LCF/mgf_Obj_1/Sarc_4-20pc_sarc_15B-17B_concat_all_the_line_dele.mgf
Protein: PREDICTED: proteasome subunit beta type-2 isoform 2 [Ovis aries]
Accession: gi|426215156|ref|XP_004001840.1| **Parent m/z:** 653.96, 2+
Sequence: R.FILNLPTFSVR.I **Score:** 27.08549005498247

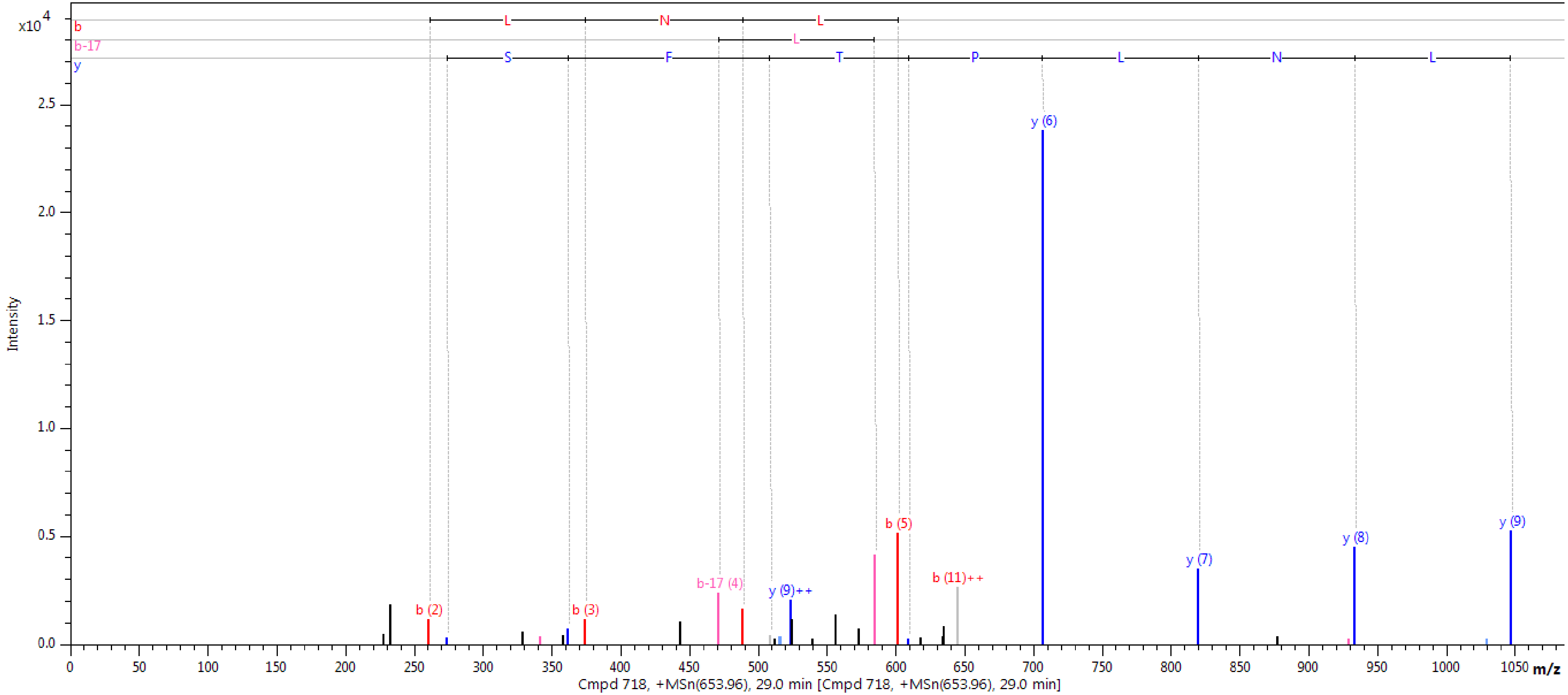

Spectrum Report

Source: M:/Documents/Lamb meat protein project/1. Characterisation of lamb skeletal proteome/Real run - 5 lambs from LCF/mgf_Obj_1/Sarc_4-20pc_sarc_15B-17B_concat_all_the_line_dele.mgf
Protein: PREDICTED: troponin C, skeletal muscle [Ovis aries]
Accession: gi|426242099|ref|XP_004014914.1|
Sequence: R.SYLSEEMIAEFK.A
Parent m/z: 731.861, 2+
Score: 46.080456743129574
Modification: Oxidation: 7

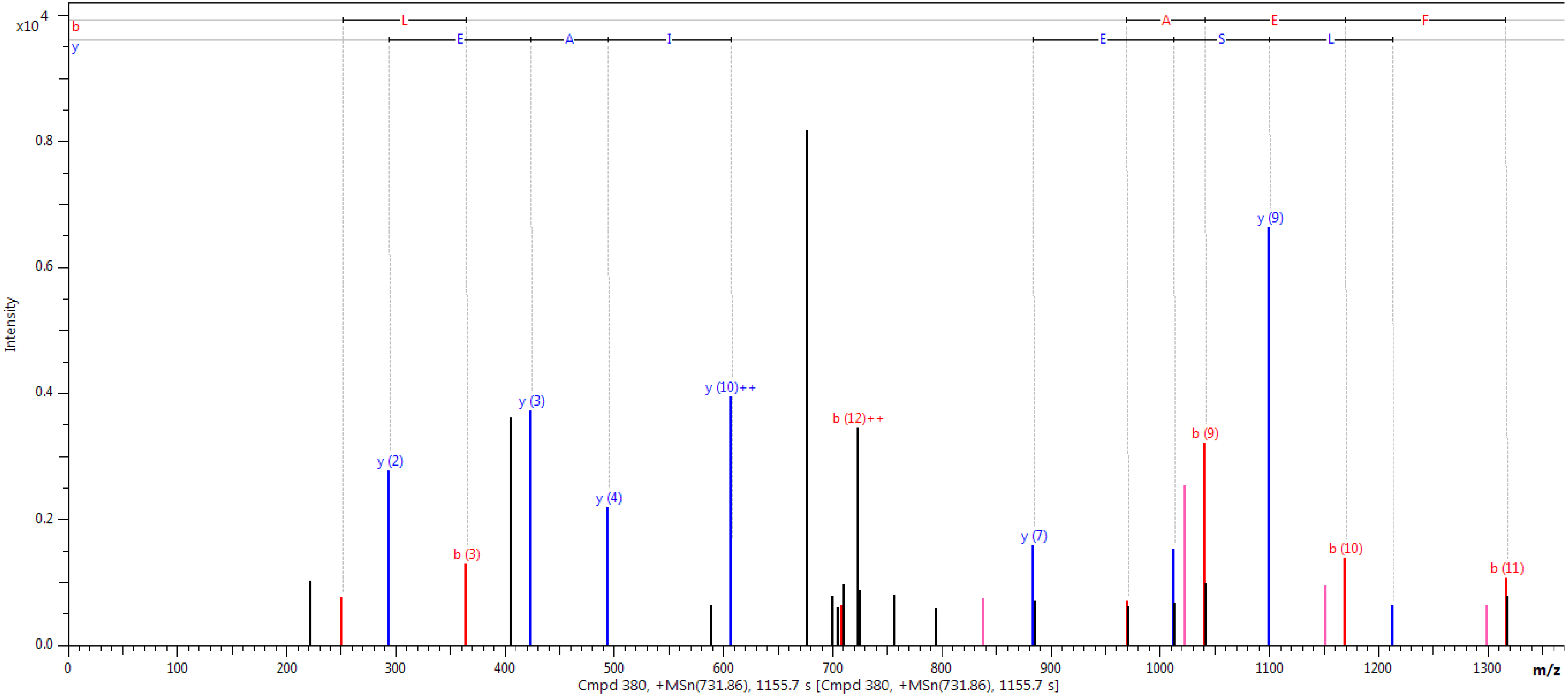

agresearch

Spectrum Report

Source: M:/Documents/Lamb meat protein project/1. Characterisation of lamb skeletal proteome/Real run - 5 lambs from LCF/mgf_Obj_1/Sarc_4-20pc_sarc_15B-17B_concat_all_the_line_dele.mgf

Protein: PREDICTED: GTP-binding nuclear protein Ran-like [Ovis aries]

Accession: gi|426233734|ref|XP_004010869.1|

Parent m/z: 607.872, 2+

Sequence: K.NLQYYDISAK.S

Score: 21.78331253797342

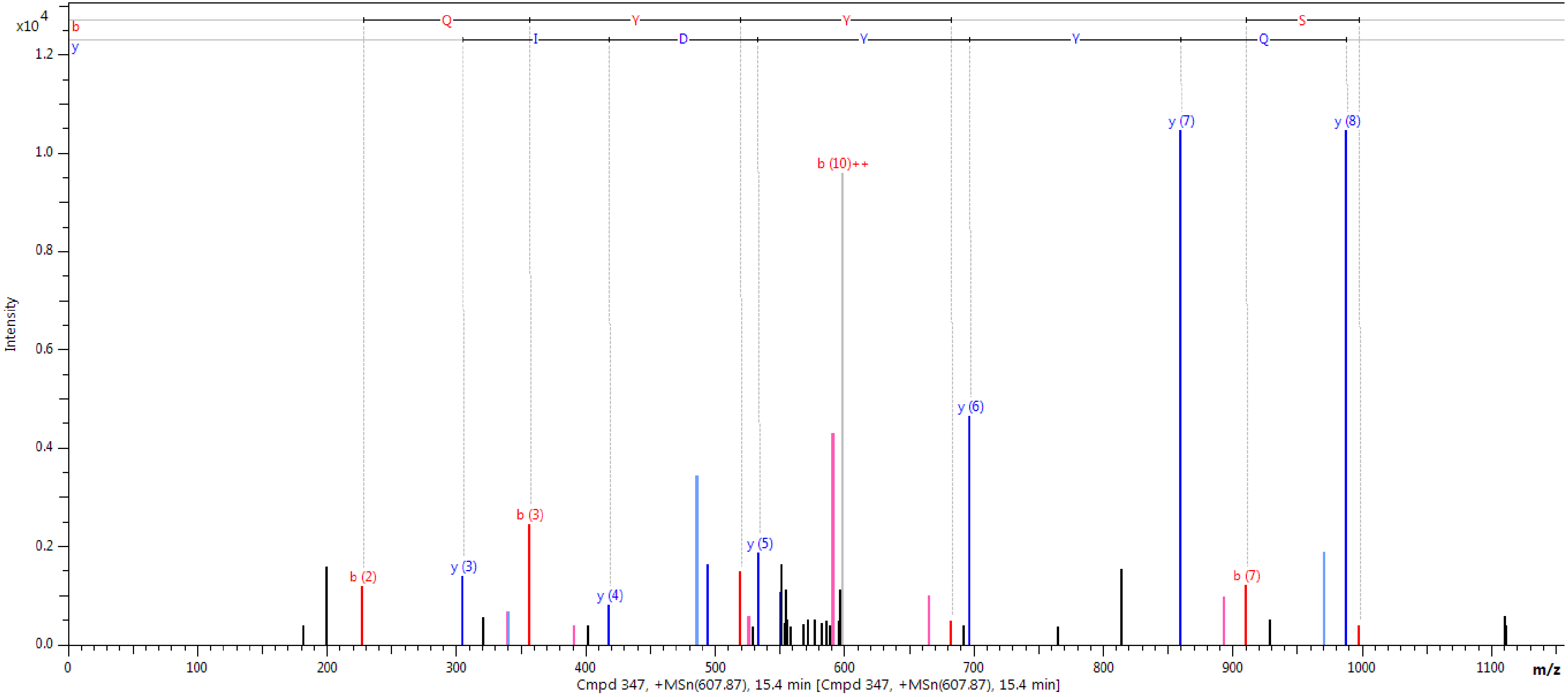

Spectrum Report

Source: M:/Documents/Lamb meat protein project/1. Characterisation of lamb skeletal proteome/Real run - 5 lambs from LCF/mgf_Obj_1/Sarc_4-20pc_sarc_15B-17B_concat_all_the_line_dele.mgf

Protein: PREDICTED: protein-L-isoaspartate(D-aspartate) O-methyltransferase [Ovis aries]

Accession: gi|426235212|ref|XP_004011583.1| **Parent m/z:** 637.386, 2+

Sequence: K.ELVDDSINNVR.K **Score:** 26.559065293596163

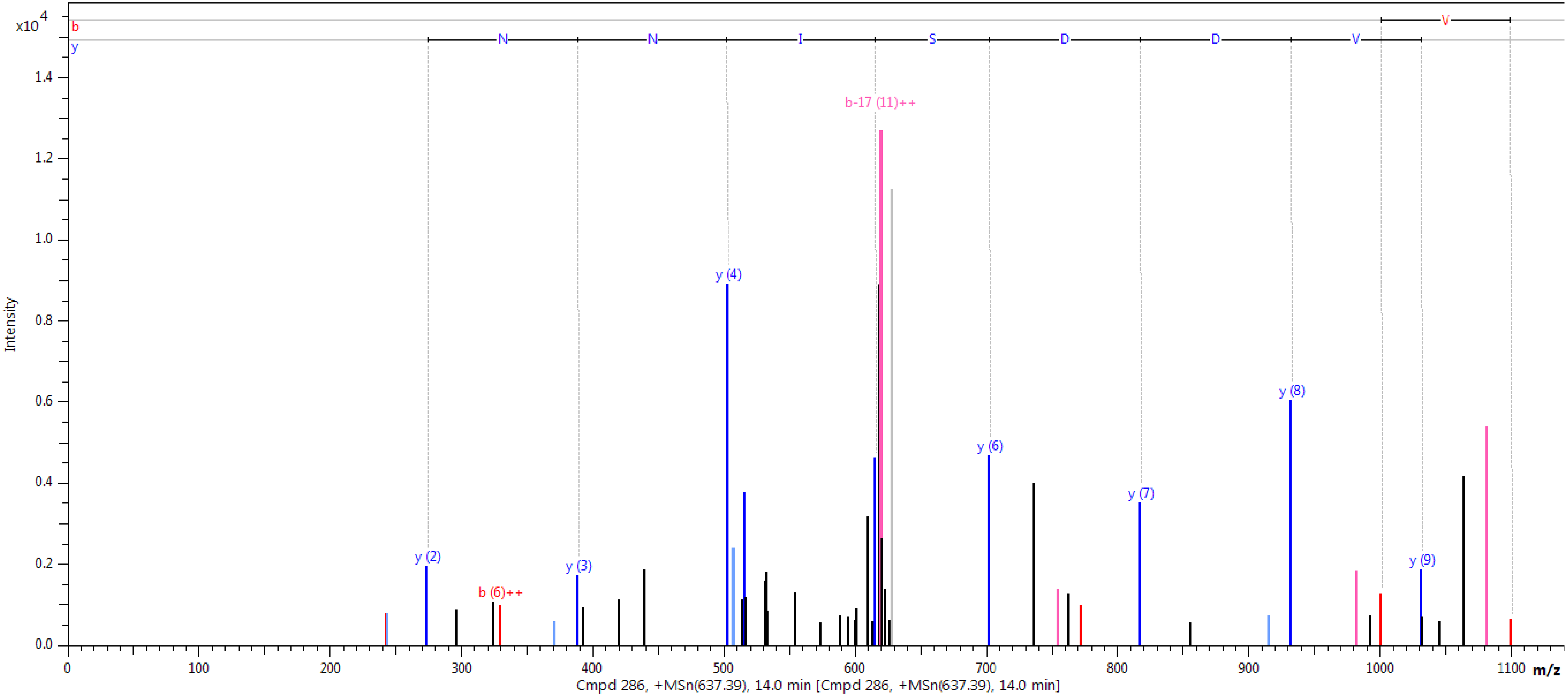

Spectrum Report

Source: M:/Documents/Lamb meat protein project/1. Characterisation of lamb skeletal proteome/Real run - 5 lambs from LCF/mgf_Obj_1/Sarc_4-20pc_sarc_15B-17B_concat_all_the_line_dele.mgf

Protein: hemoglobin beta

Accession: gi|229272|prf||681075A

Sequence: R.LLGDVLVVVLAR.H

Parent m/z: 633.984, 2+

Score: 26.755858355441944

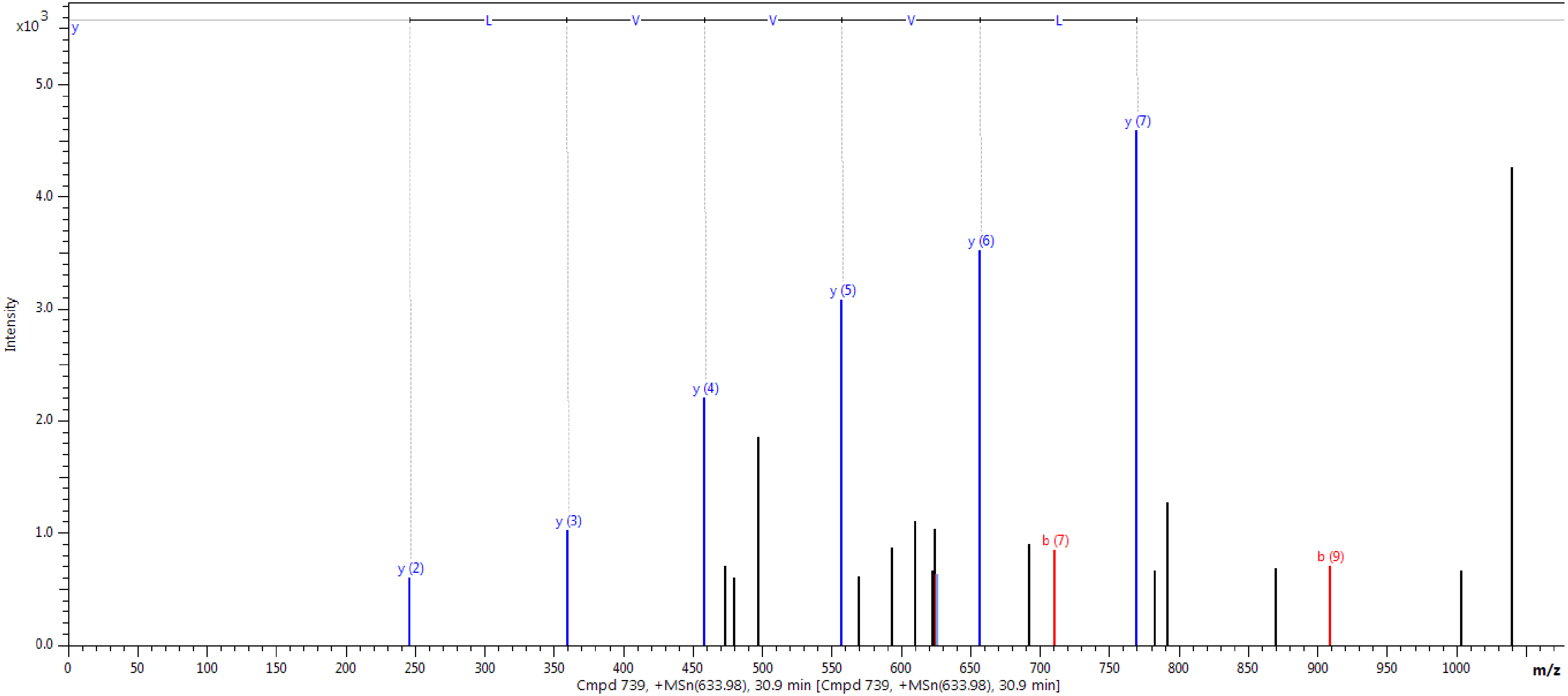

agresearch

Spectrum Report

Source: M:/Documents/Lamb meat protein project/1. Characterisation of lamb skeletal proteome/Real run - 5 lambs from LCF/mgf_Obj_1/Sarc_4-20pc_sarc_15B-17B_concat_all_the_line_dele.mgf
Protein: PREDICTED: protein-arginine deiminase type-2 [Ovis aries]
Accession: gi|426222856|ref|XP_004005598.1|
Parent m/z: 652.412, 2+
Sequence: R.ILIGSSFPLSGGR.R
Score: 49.15756272290985

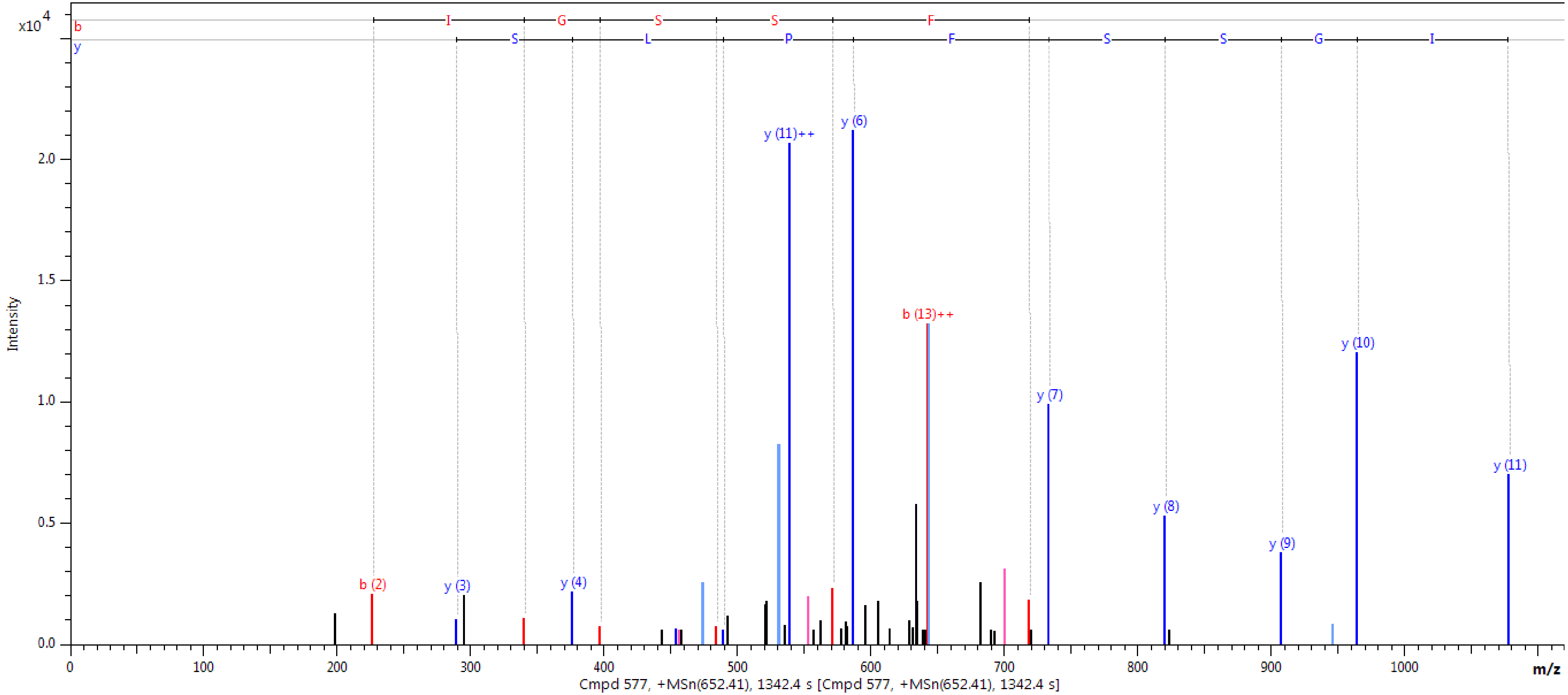

Spectrum Report

Source: M:/Documents/Lamb meat protein project/1. Characterisation of lamb skeletal proteome/Real run - 5 lambs from LCF/mgf_Obj_1/Sarc_4-20pc_sarc_15B-17B_concat_all_the_line_dele.mgf
Protein: PREDICTED: cytoplasmic aconitate hydratase isoform 1 [Ovis aries]
Accession: gi|426220547|ref|XP_004004476.1|
Parent m/z: 736.035, 2+
Sequence: R.YQQAGLPLIVLAGK.E
Score: 44.07327030964163

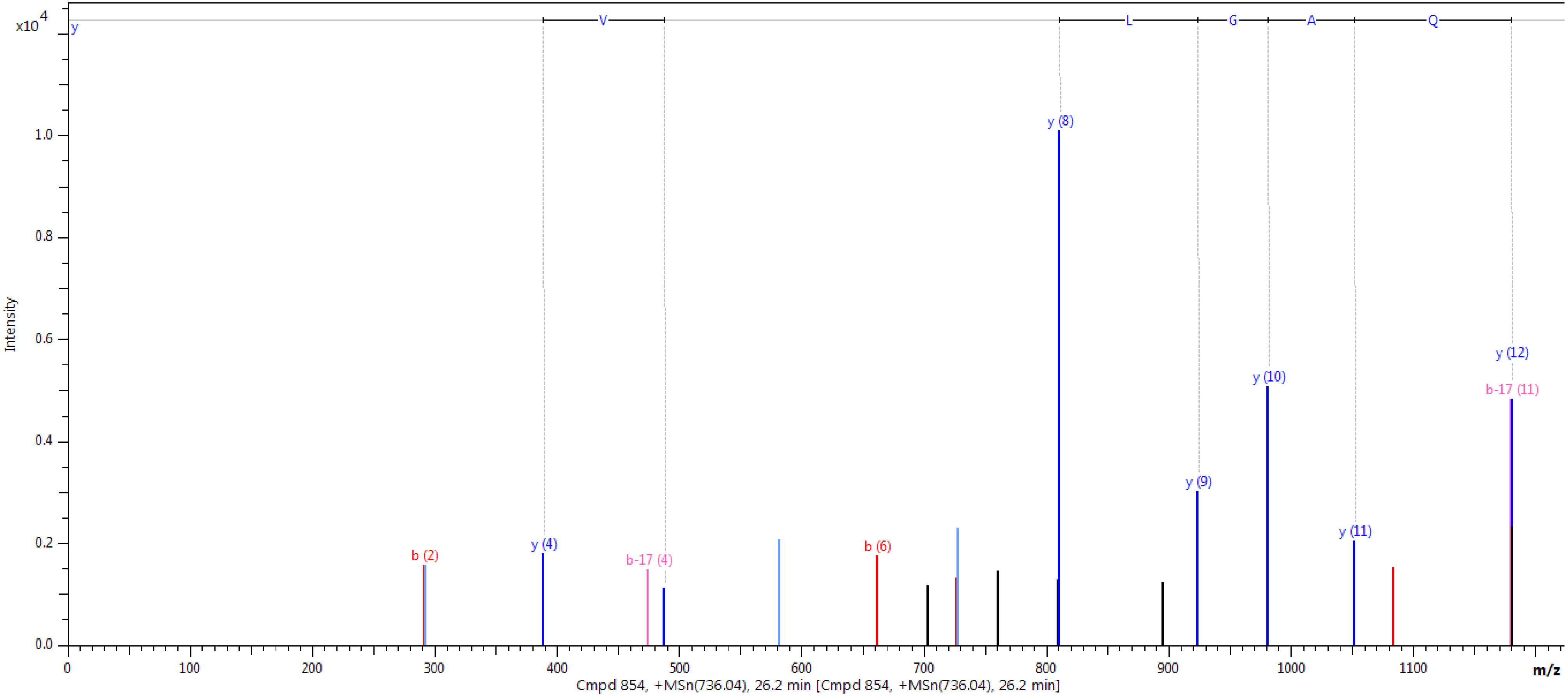

Spectrum Report

Source: M:/Documents/Lamb meat protein project/1. Characterisation of lamb skeletal proteome/Real run - 5 lambs from LCF/mgf_Obj_1/Sarc_4-20pc_sarc_15B-17B_concat_all_the_line_dele.mgf
Protein: PREDICTED: carboxymethylenebutenolidase homolog isoform 1 [Ovis aries]
Accession: gi|426246710|ref|XP_004017134.1|
Parent m/z: 503.884, 2+
Sequence: R.AGVSVYGIIK.D
Score: 26.543655749413936

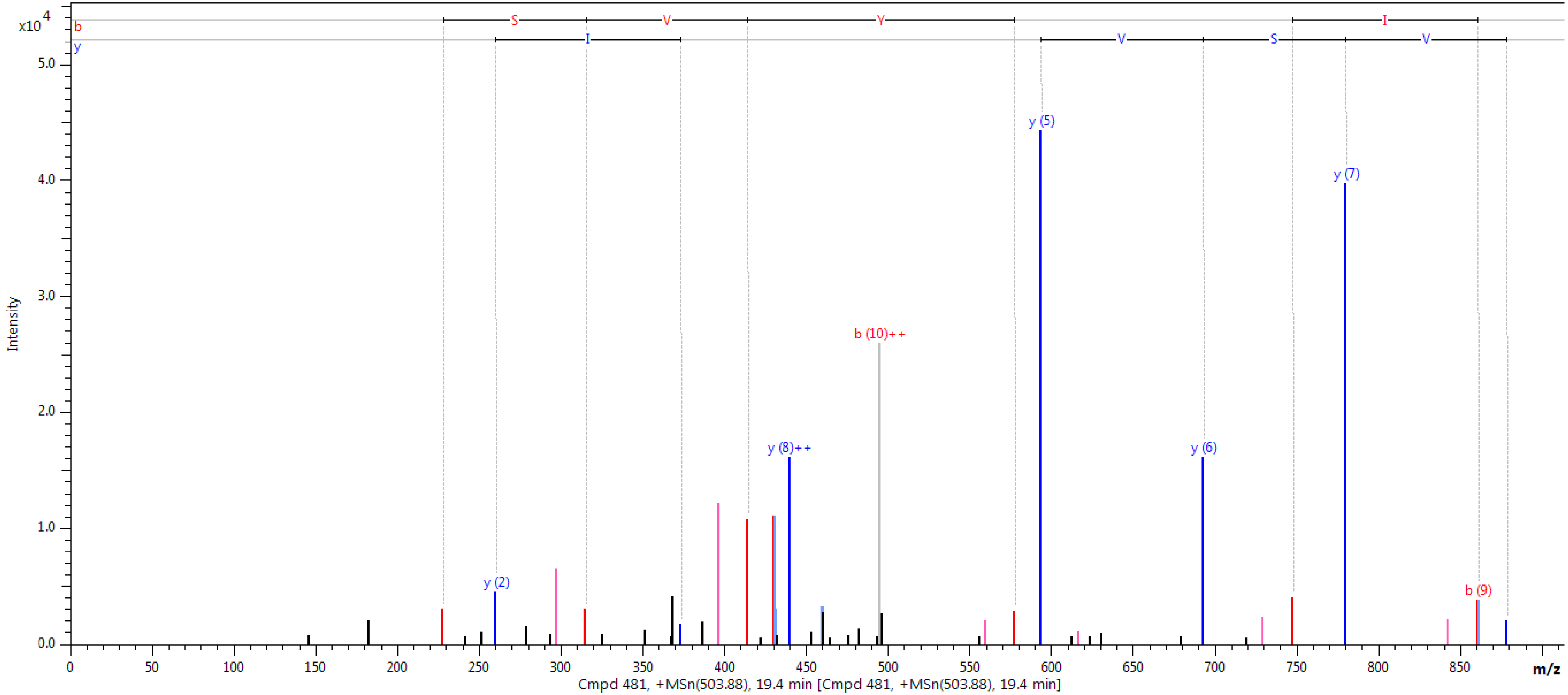

Spectrum Report

Source: M:/Documents/Lamb meat protein project/1. Characterisation of lamb skeletal proteome/Real run - 5 lambs from LCF/mgf_Obj_1/Sarc_4-20pc_sarc_15B-17B_concat_all_the_line_dele.mgf
Protein: PREDICTED: annexin A3 [Ovis aries]
Accession: gi|426231916|ref|XP_004009983.1|
Sequence: R.SEIDLLDIR.A
Parent m/z: 537.424, 2+
Score: 23.009084102302147

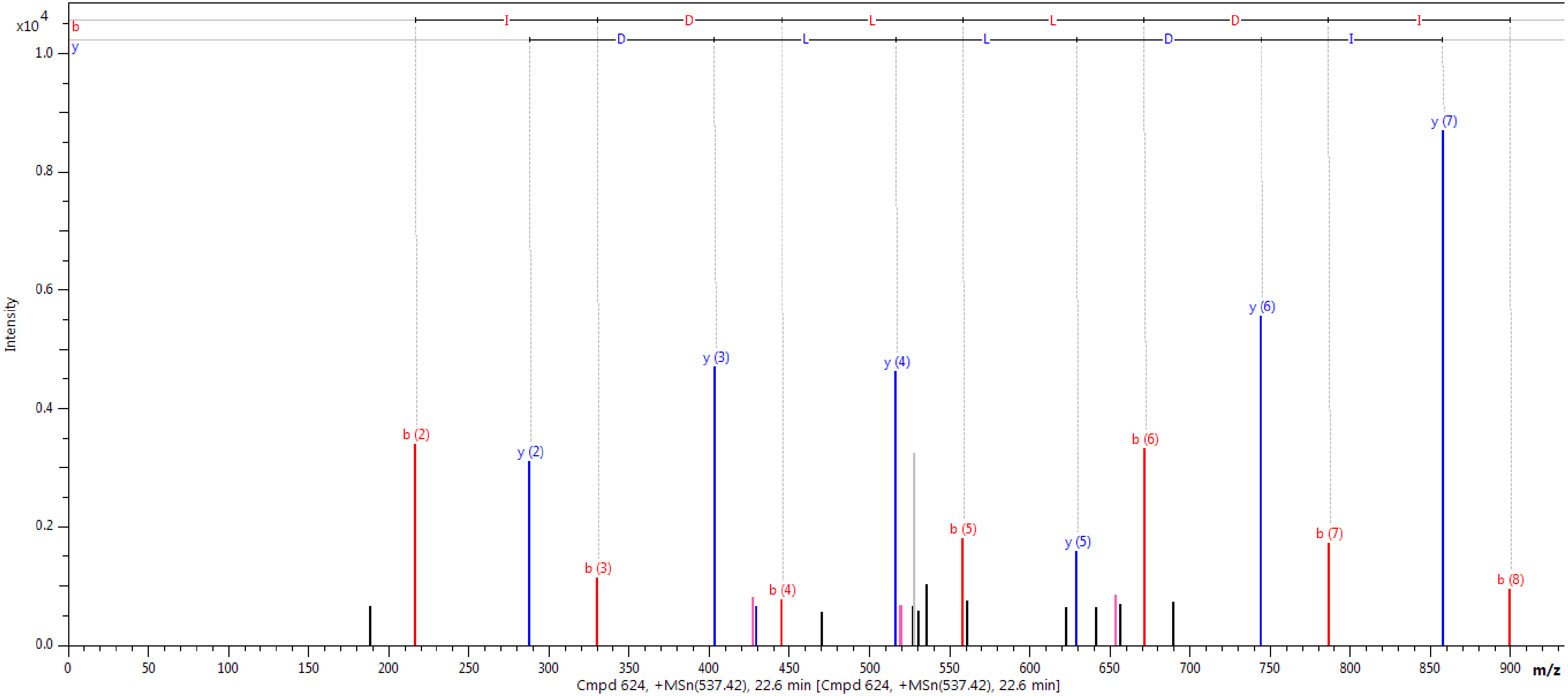

Spectrum Report

Source: M:/Documents/Lamb meat protein project/1. Characterisation of lamb skeletal proteome/Real run - 5 lambs from LCF/ mgf_Obj_1/Sarc_4-20pc_sarc_15B-17B_concat_all_the_line_dele.mgf

Protein: RecName: Full=Galectin-1; Short=Gal-1; AltName: Full=14 kDa lectin; AltName: Full=Beta-galactoside-binding lectin L-14-I; AltName: Full=Galaptin; AltName: Full=Lactose-binding lectin 1; AltName: Full=Lectin galactoside-binding soluble 1; AltName: Full=OPG-1; AltName: Full=S-Lac lectin 1

Accession: gi|3122339|sp|P81184.2|LEG1_SHEEP

Parent m/z: 523.743, 2+

Sequence: K.DGGAWGAEQR.E

Score: 34.54197006944697

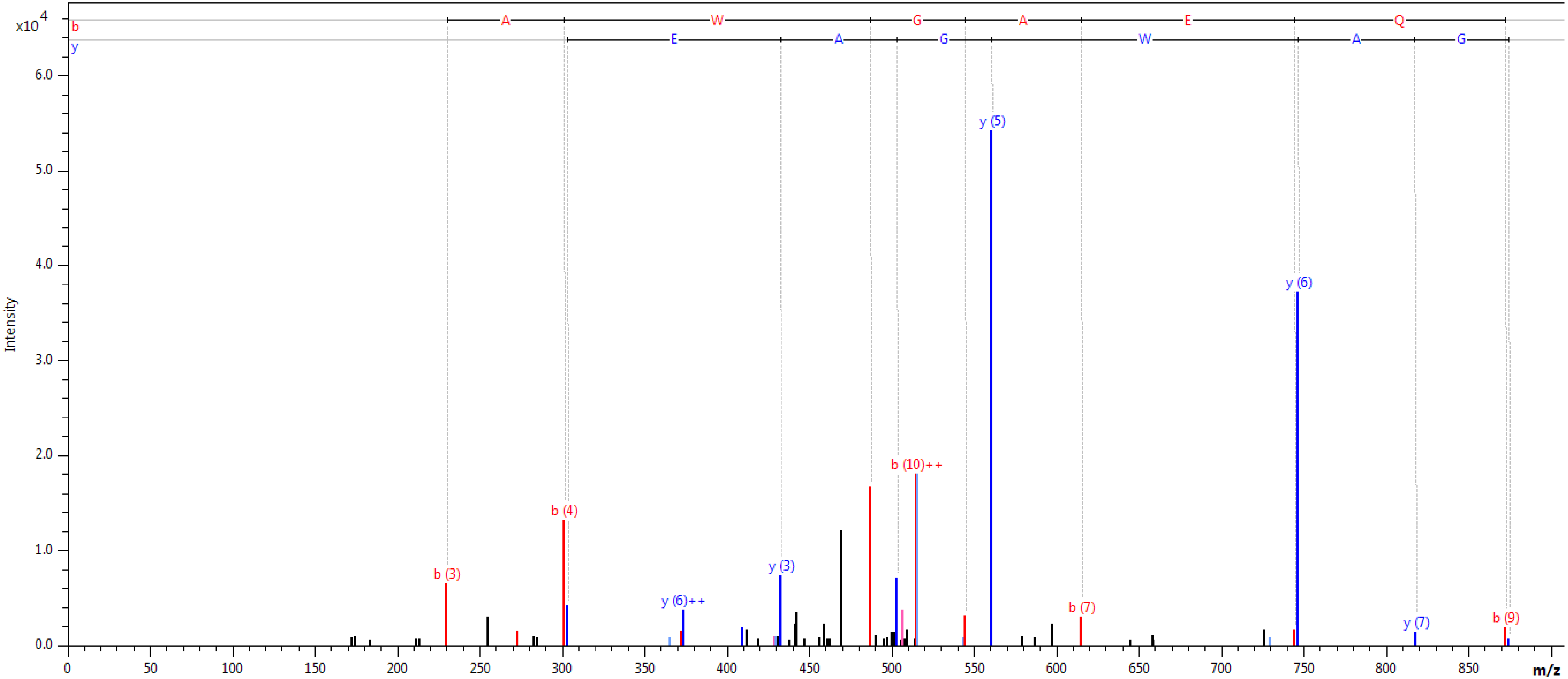

Cmpd 118, +MSn(523.74), 518.2 s [Cmpd 118, +MSn(523.74), 518.2 s]

agresearch

Spectrum Report

Source: M:/Documents/Lamb meat protein project/1. Characterisation of lamb skeletal proteome/Real run - 5 lambs from LCF/mgf_Obj_1/Sarc_4-20pc_sarc_15B-17B_concat_all_the_line_dele.mgf
Protein: PREDICTED: glutathione S-transferase omega-1-like [Ovis aries]
Accession: gi|426253387|ref|XP_004020378.1|
Parent m/z: 692.888, 2+
Sequence: K.NDPIVSSLPTDVK.T
Score: 35.13308880988391

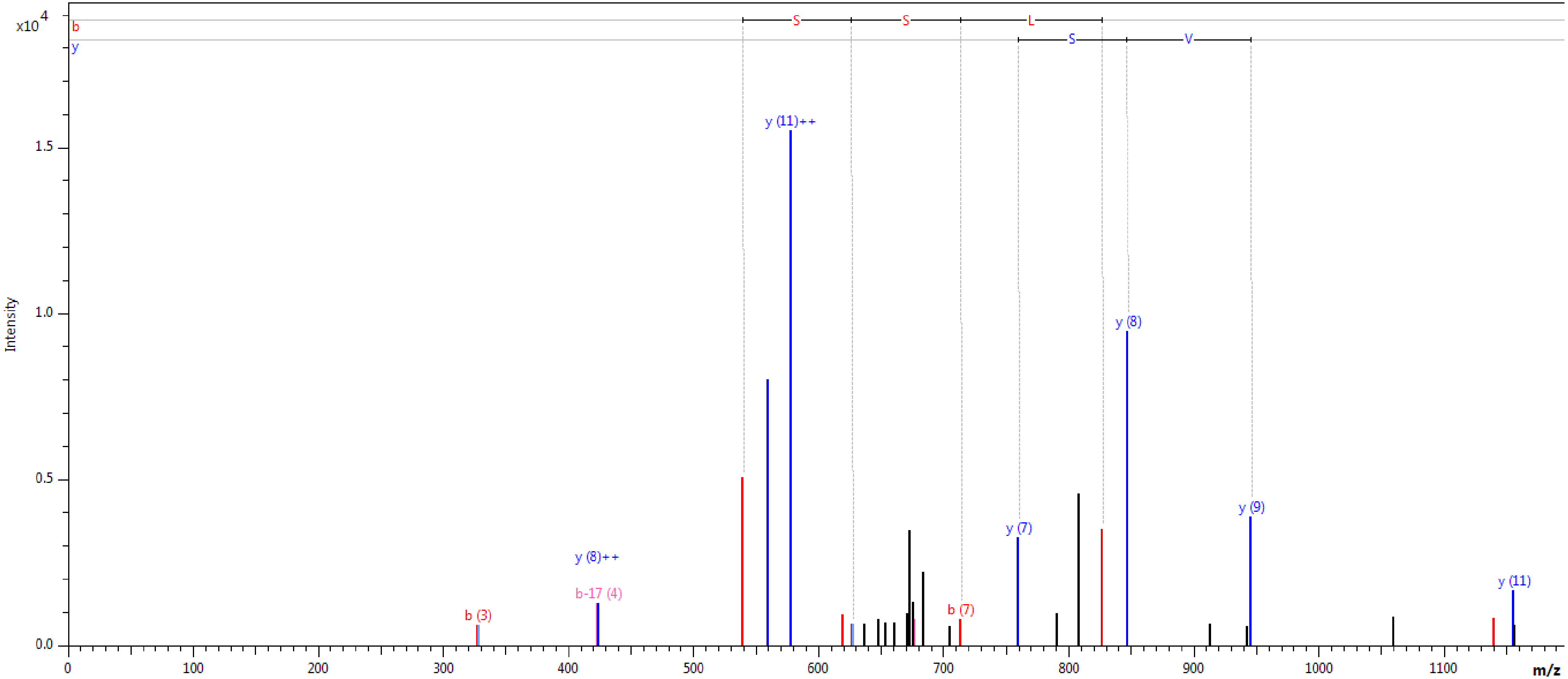

Cmpd 446, +MSn(692.89), 18.4 min [Cmpd 446, +MSn(692.89), 18.4 min]

Spectrum Report

Source: M:/Documents/Lamb meat protein project/1. Characterisation of lamb skeletal proteome/Real run - 5 lambs from LCF/mgf_Obj_1/Sarc_4-20pc_sarc_15B-17B_concat_all_the_line_dele.mgf
Protein: PREDICTED: 14 kDa phosphohistidine phosphatase-like, partial [Ovis aries]
Accession: gi|426258610|ref|XP_004022902.1|
Parent m/z: 636.323, 2+
Sequence: K.GYDCECLGGGR.I
Score: 22.70165649217371
Modification: Propionamide: 4, 6

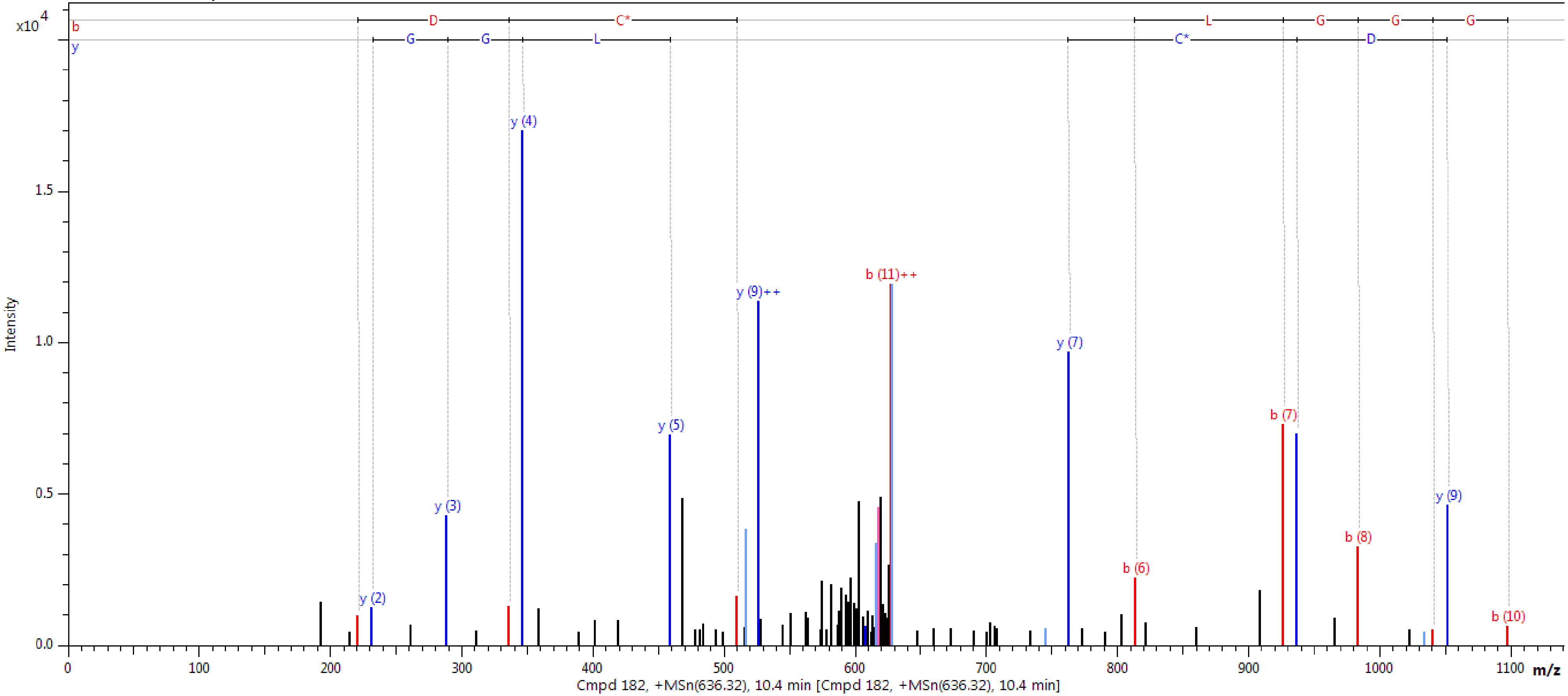

Spectrum Report

Source: M:/Documents/Lamb meat protein project/1. Characterisation of lamb skeletal proteome/Real run - 5 lambs from LCF/mgf_Obj_1/Sarc_4-20pc_sarc_15B-17B_concat_all_the_line_dele.mgf
Protein: PREDICTED: importin-5 isoform 2 [Ovis aries]
Accession: gi|426236611|ref|XP_004012261.1|
Sequence: K.SLVEIADTVPK.Y
Parent m/z: 586.431, 2+
Score: 26.466743803471104

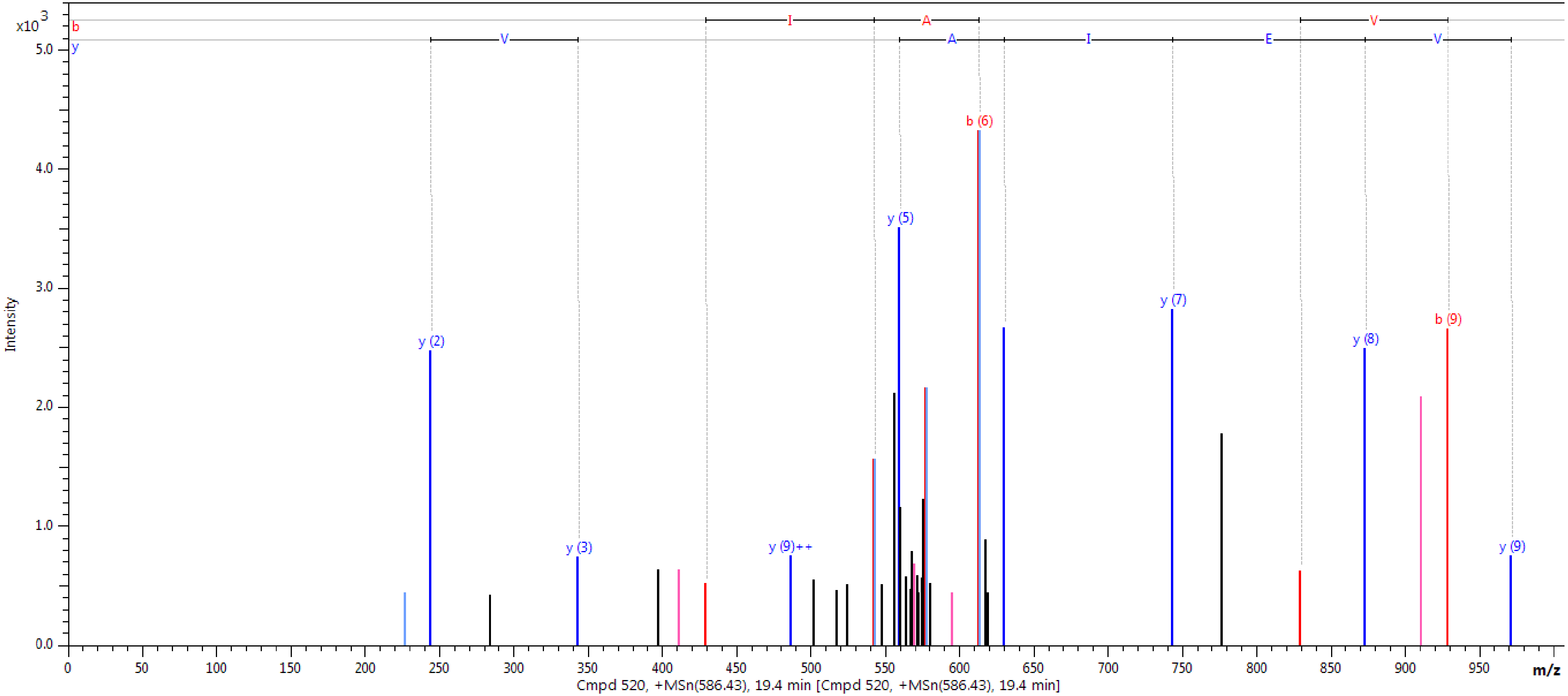

agresearch

Spectrum Report

Source: M:/Documents/Lamb meat protein project/1. Characterisation of lamb skeletal proteome/Real run - 5 lambs from LCF/mgf_Obj_1/Sarc_4-20pc_sarc_15B-17B_concat_all_the_line_dele.mgf
Protein: PREDICTED: proteasome subunit alpha type-5 isoform 1 [Ovis aries]
Accession: gi|426216148|ref|XP_004002329.1|
Sequence: R.LFQVEYAIEAIK.L
Parent m/z: 712.403, 2+
Score: 22.913131653088783

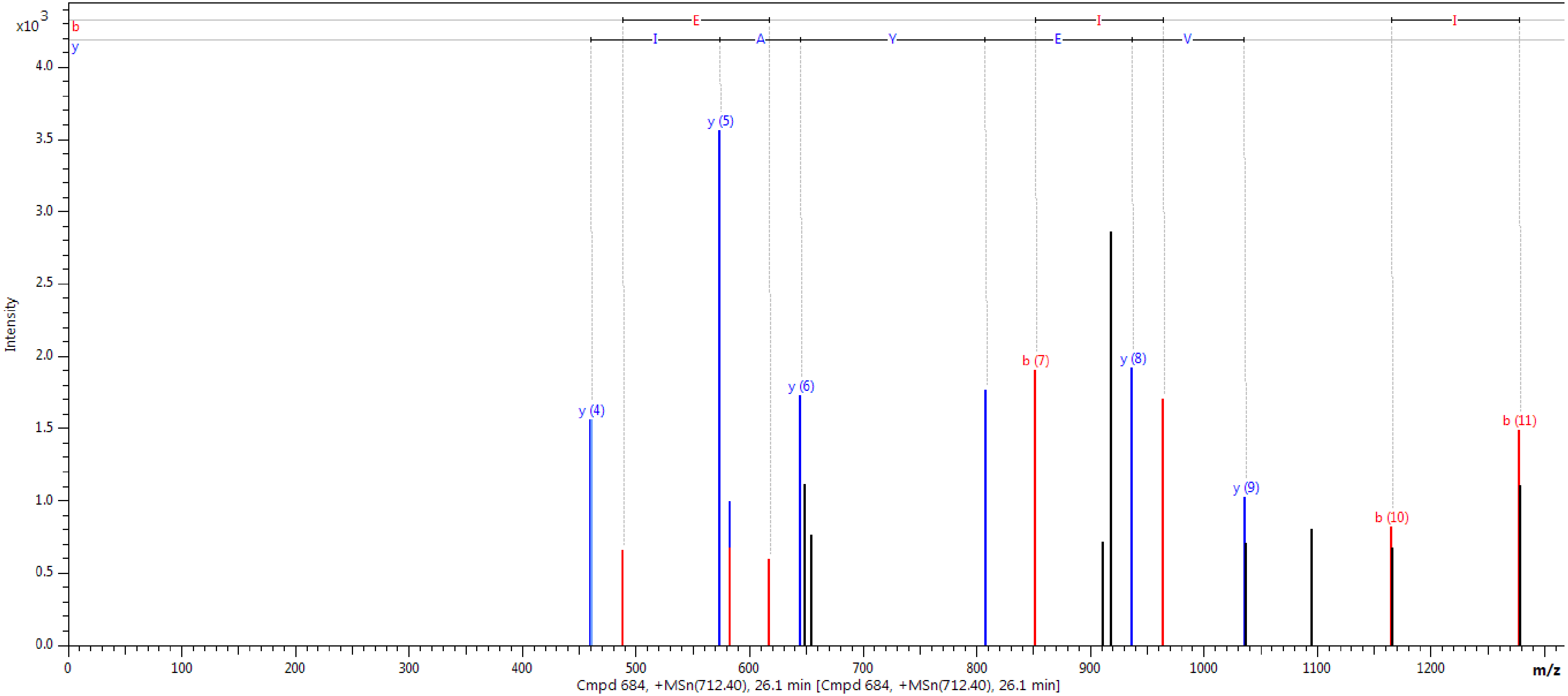

Spectrum Report

Source: M:/Documents/Lamb meat protein project/1. Characterisation of lamb skeletal proteome/Real run - 5 lambs from LCF/mgf_Obj_1/Sarc_4-20pc_sarc_15B-17B_concat_all_the_line_dele.mgf
Protein: immunoglobulin V lambda chain 5.1.18 [Ovis aries]
Accession: gi|26245571|gb|AAN77358.1|
Parent m/z: 857.48, 2+
Sequence: A.QAPLTQPSSVSGSLGQR.V
Score: 36.34455859970531

Modification: Deamidated: 6

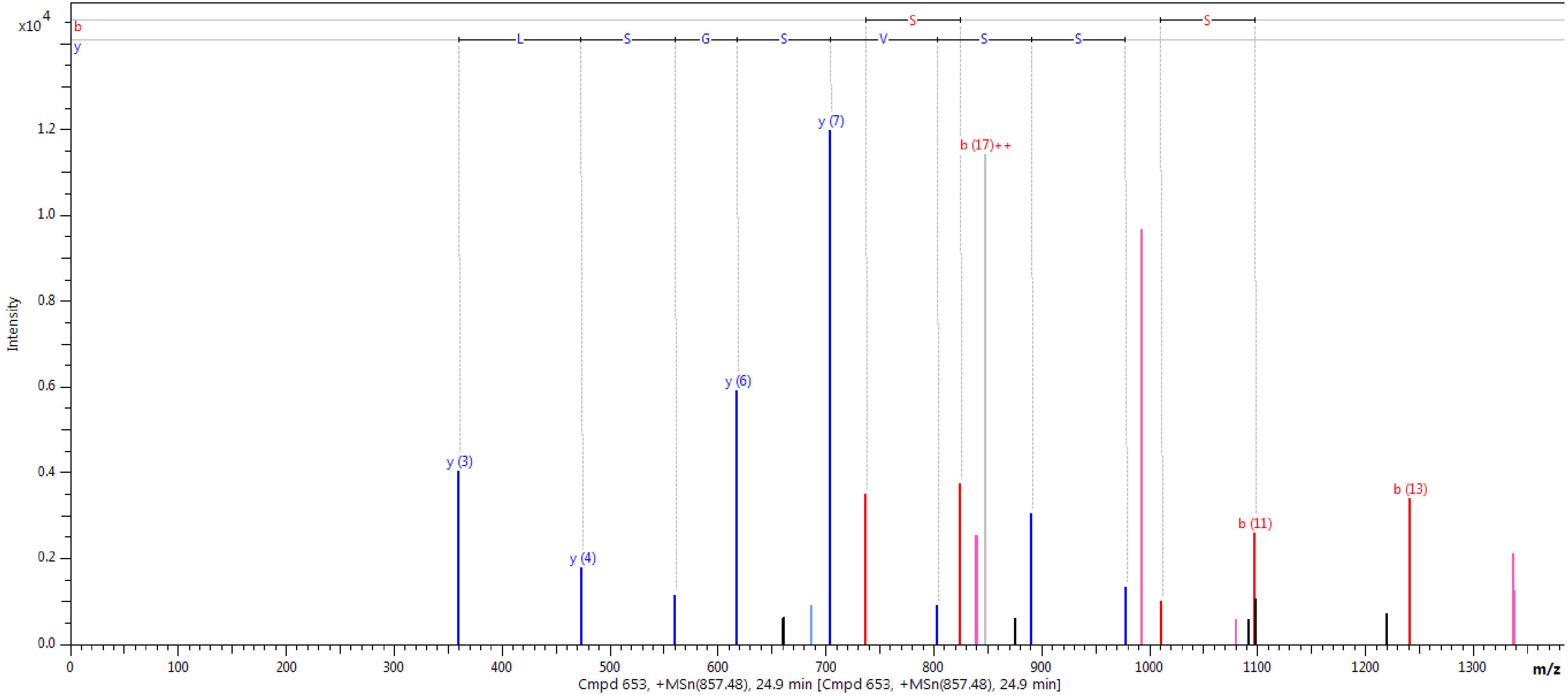

Spectrum Report

Source: M:/Documents/Lamb meat protein project/1. Characterisation of lamb skeletal proteome/Real run - 5 lambs from LCF/mgf_Obj_1/Sarc_4-20pc_sarc_15B-17B_concat_all_the_line_dele.mgf
Protein: PREDICTED: cullin-associated NEDD8-dissociated protein 1 isoform 2 [Ovis aries]
Accession: gi|426224763|ref|XP_004006538.1|
Parent m/z: 628.977, 2+
Sequence: K.LGTLSALDILIK.N
Score: 39.574638632778566

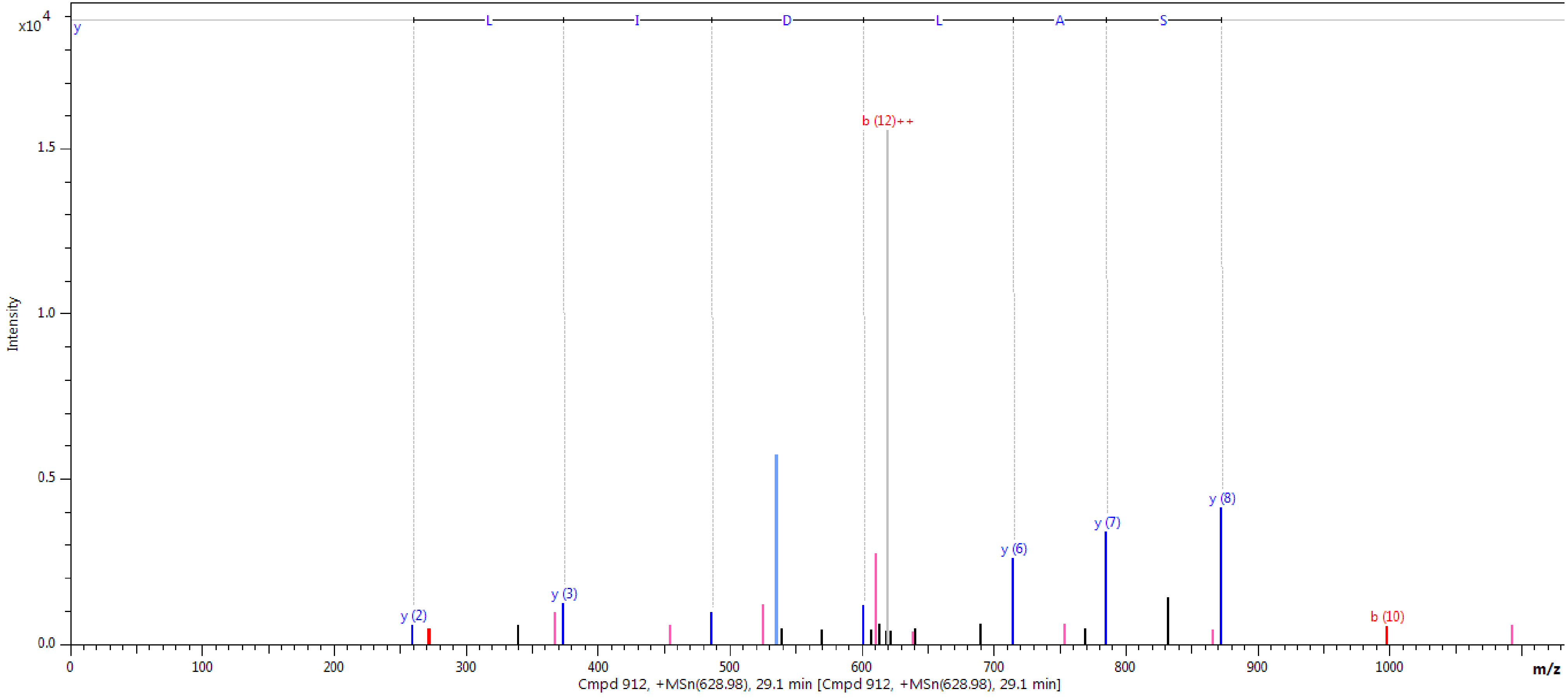

Spectrum Report

Source: M:/Documents/Lamb meat protein project/1. Characterisation of lamb skeletal proteome/Real run - 5 lambs from LCF/mgf_Obj_1/Sarc_4-20pc_sarc_15B-17B_concat_all_the_line_dele.mgf
Protein: PREDICTED: dihydrolipoyllysine-residue succinyltransferase component of 2-oxoglutarate dehydrogenase complex, mitochondrial [Ovis aries]
Accession: gi|426233678|ref|XP_004010842.1|
Parent m/z: 589.796, 2+
Sequence: K.AKPAAAPADAAPK.A
Score: 26.407452293638393

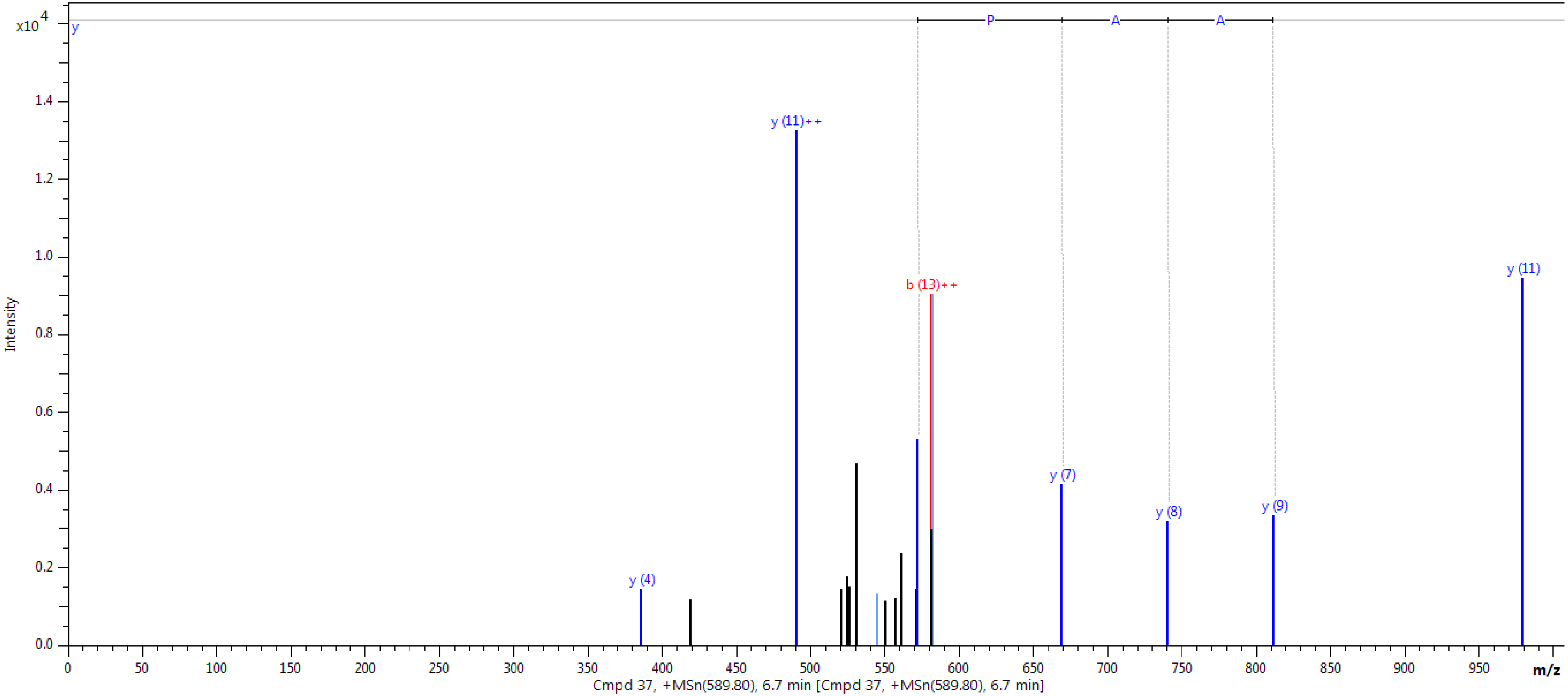

agresearch

Spectrum Report

Source: M:/Documents/Lamb meat protein project/1. Characterisation of lamb skeletal proteome/Real run - 5 lambs from LCF/mgf_Obj_1/Sarc_4-20pc_sarc_15B-17B_concat_all_the_line_dele.mgf

Protein: PREDICTED: complement C3-like, partial [Ovis aries]

Accession: gi|426258629|ref|XP_004022911.1|

Sequence: R.DYAGVFTDAGLTFK.T

Parent m/z: 752.955, 2+

Score: 23.215799629194493

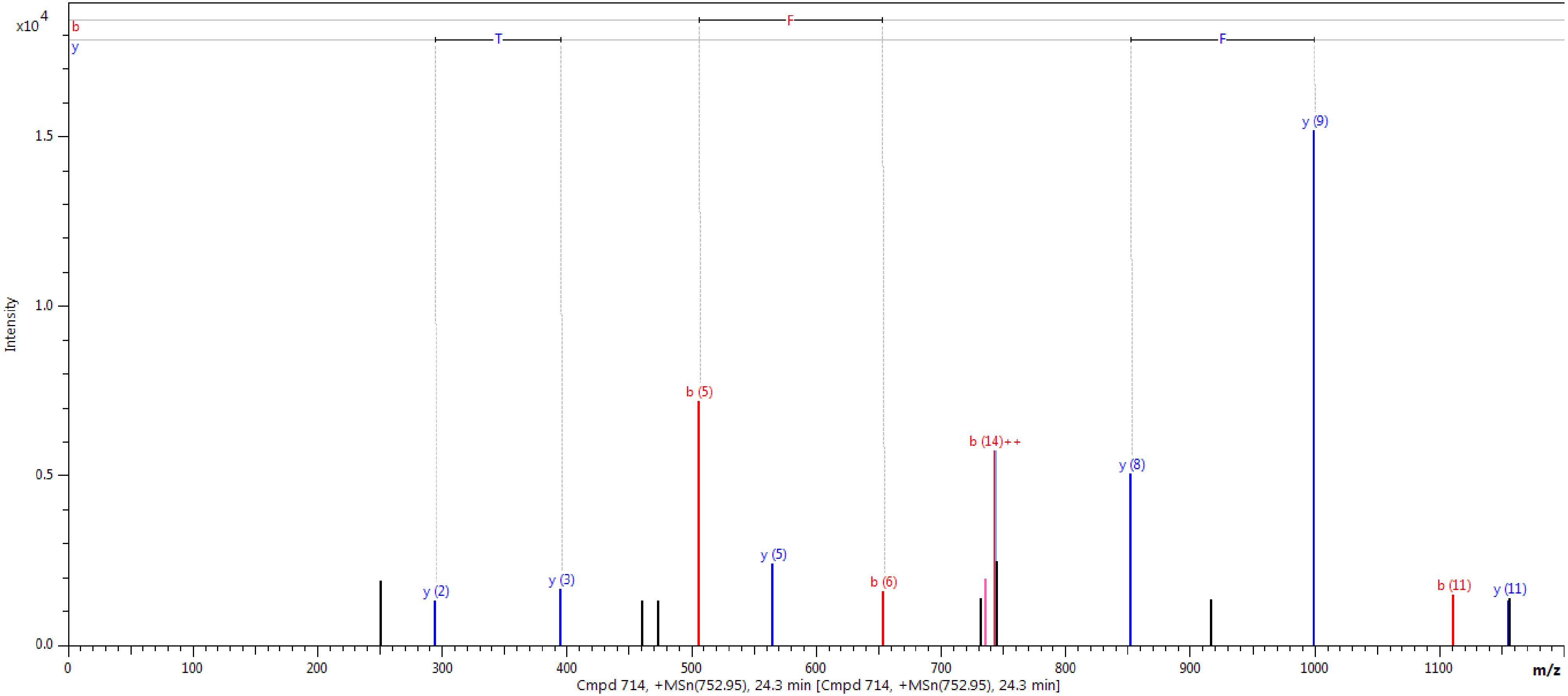

Spectrum Report

Source: M:/Documents/Lamb meat protein project/1. Characterisation of lamb skeletal proteome/Real run - 5 lambs from LCF/ mgf_Obj_1/Sarc_4-20pc_sarc_15B-17B_concat_all_the_line_dele.mgf
Protein: PREDICTED: protein S100-B [Ovis aries]
Accession: gi|426218413|ref|XP_004003441.1|
Parent m/z: 569.588, 3+
Sequence: K.AMVALIDVFHQYSGR.E
Score: 30.38720846120768

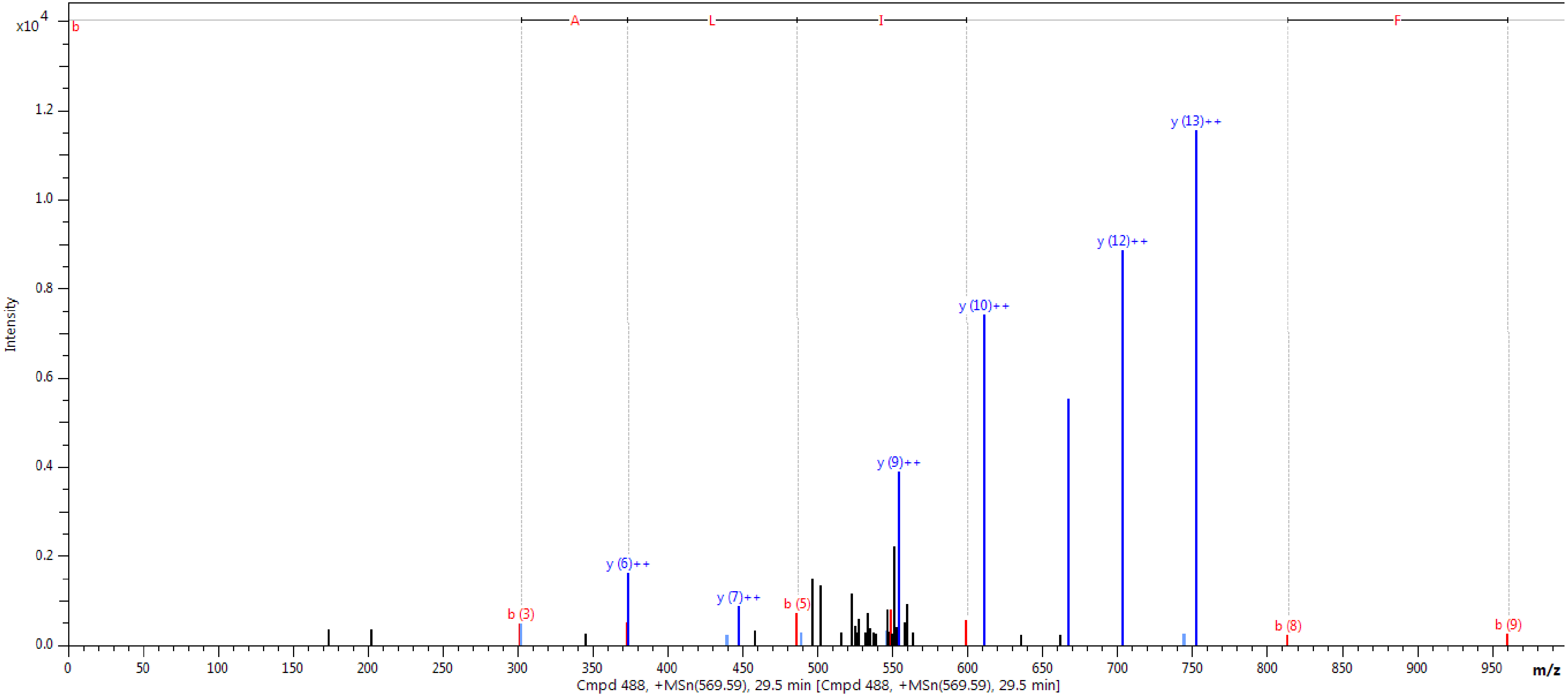

Spectrum Report

Source: M:/Documents/Lamb meat protein project/1. Characterisation of lamb skeletal proteome/Real run - 5 lambs from LCF/mgf_Obj_1/Sarc_4-20pc_sarc_15B-17B_concat_all_the_line_dele.mgf

Protein: PREDICTED: glycerol-3-phosphate dehydrogenase 1-like protein [Ovis aries]

Accession: gi|426249022|ref|XP_004018251.1|

Parent m/z: 769.969, 2+

Sequence: K.LTDIINNDHENVK.Y

Score: 43.42414516162694

Modification: Methyl: 3

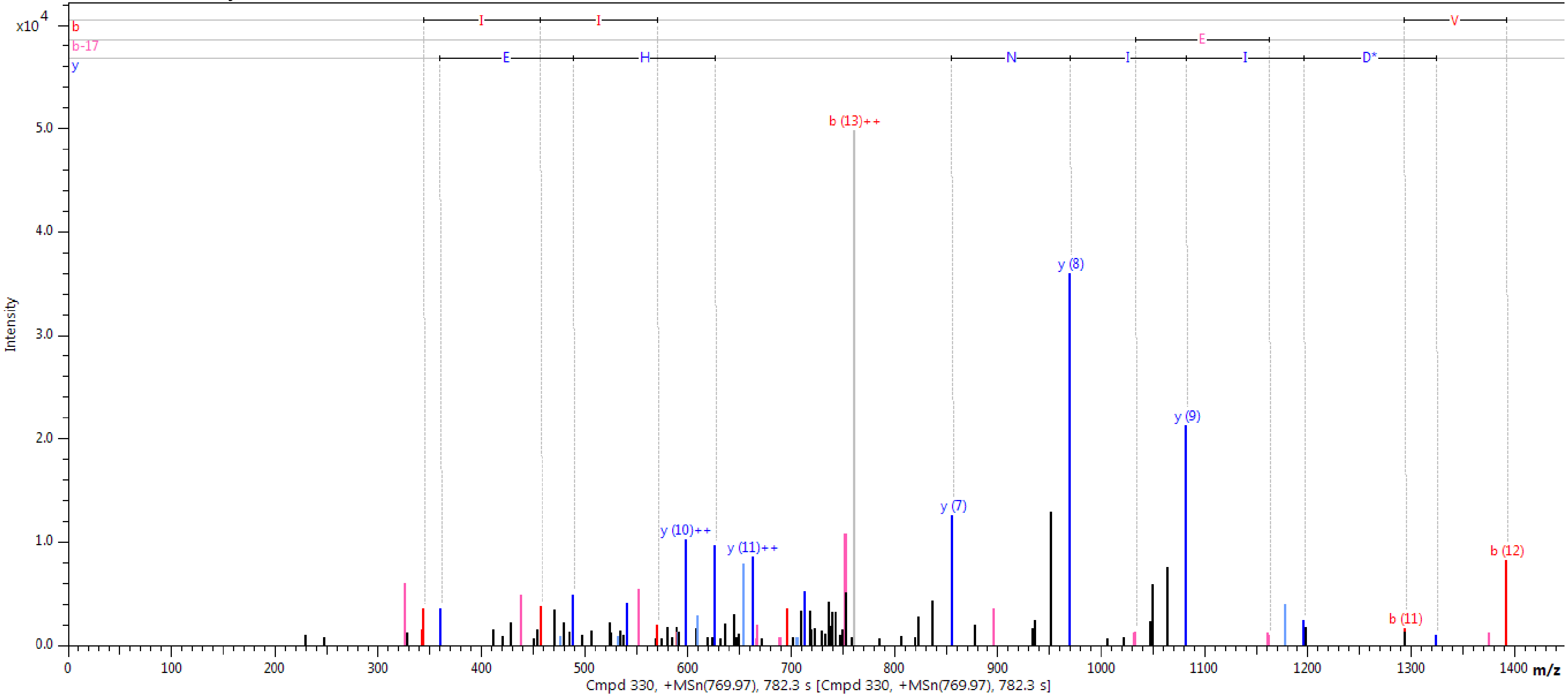

Supplement: Supplementary file 1 — Supplementary data [file mmc1.zip › Supple_data_3a_lamb_LL_prot_YMCD.pdf]
