# Supplementary material for: Data for in-depth characterisation of the lamb meat proteome from longissimus lumborum
Source: Data Brief. 2015 Feb 20;3:143–8. doi: 10.1016/j.dib.2015.02.006 (PMC4510072; doi:10.1016/j.dib.2015.02.006)

agresearch

Spectrum Report

Source: M:/Documents/Lamb meat protein project/1. Characterisation of lamb skeletal proteome/Real run - 5 lambs from LCF/ mgf_Obj_1/Myo_4-20pc_myo_15B-17B_concat_all_the_line_delet.mgf
Protein: PREDICTED: myosin light chain 1/3, skeletal muscle isoform isoform 2 [Ovis aries]
Accession: gi|426221486|ref|XP_004004941.1|
Parent m/z: 628.905, 2+
Sequence: M.SFSAEQIAEFK.E
Score: 55.21070061143779

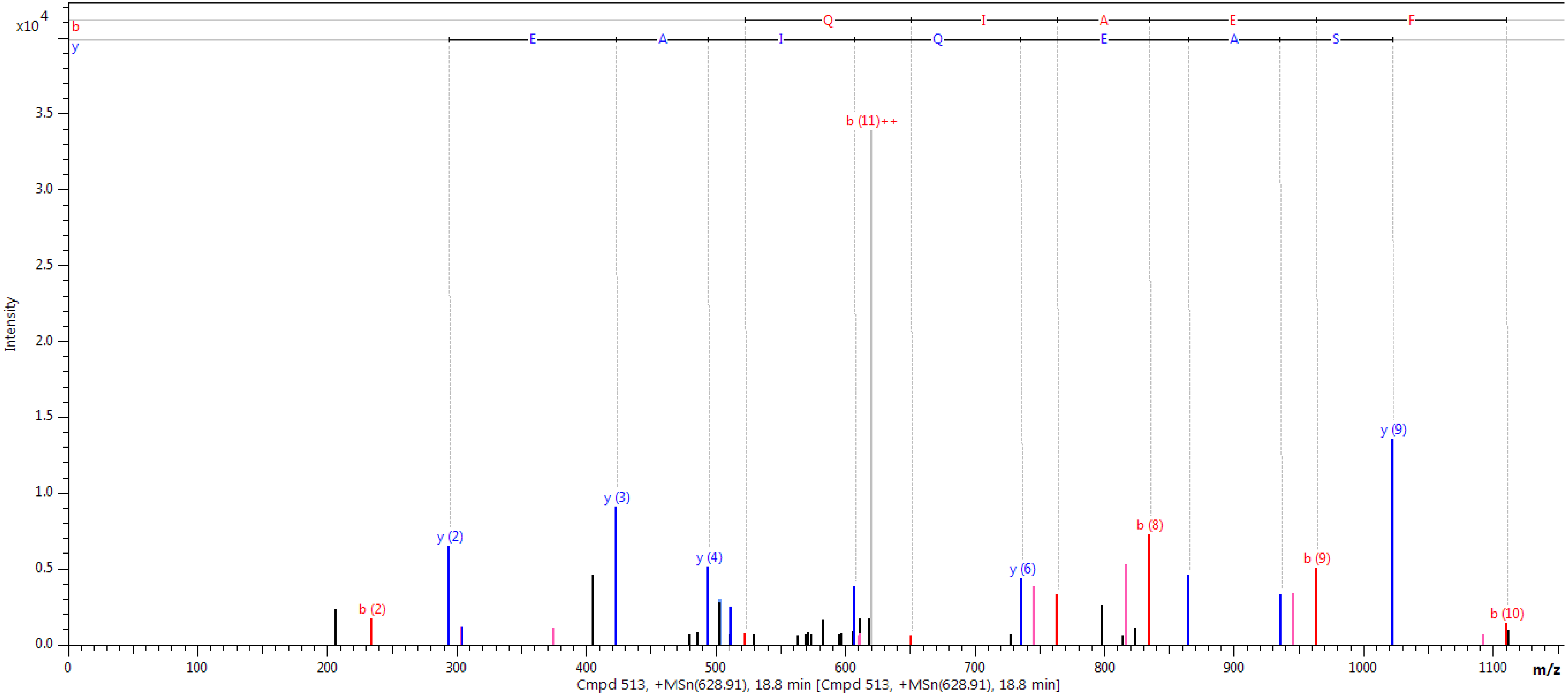

agresearch

Spectrum Report

Source: M:/Documents/Lamb meat protein project/1. Characterisation of lamb skeletal proteome/Real run - 5 lambs from LCF/ mgf_Obj_1/Myo_4-20pc_myo_15B-17B_concat_all_the_line_delet.mgf
Protein: beta actin [Ovis aries]
Accession: gi|2182269|gb|AAB60717.1|
Sequence: R.GYSFTTTAER.E
Parent m/z: 566.779, 2+
Score: 55.53815568578388

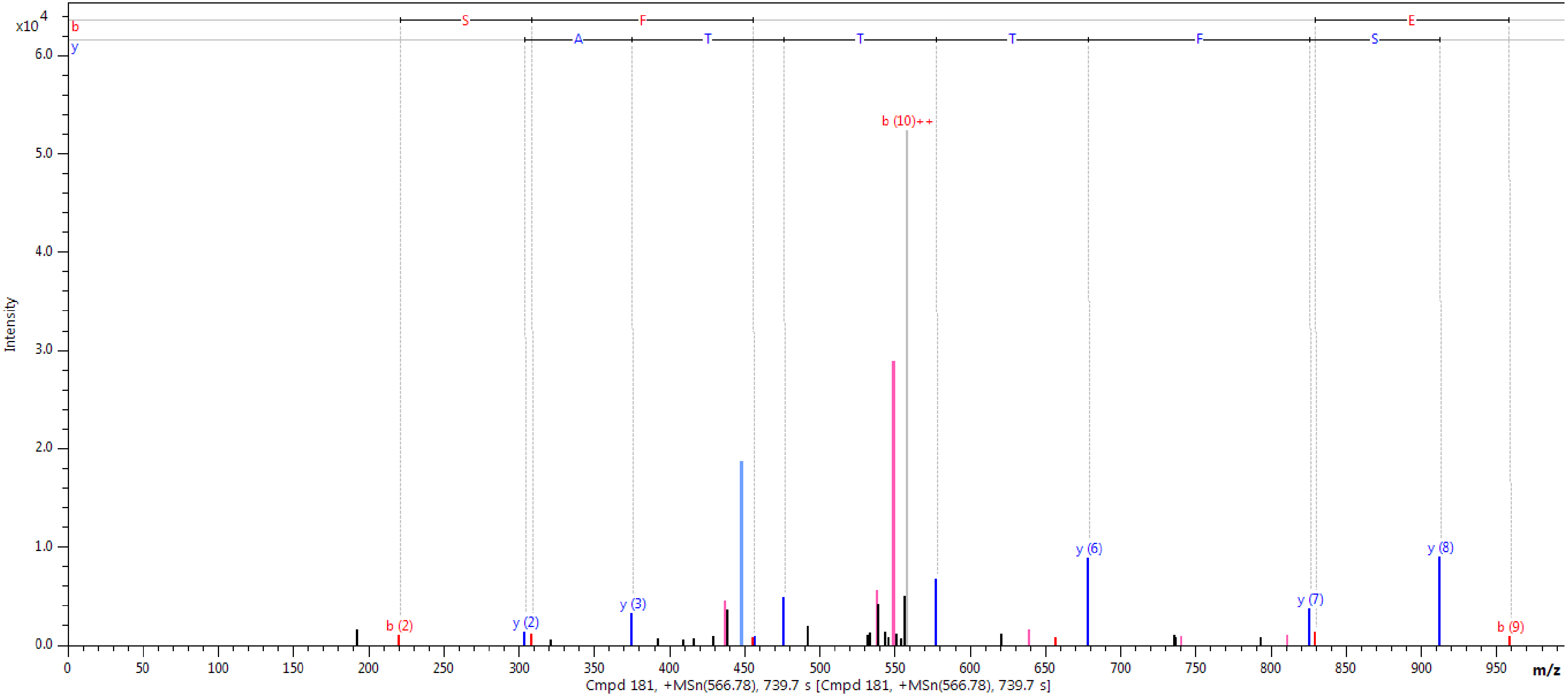

Spectrum Report

Source: M:/Documents/Lamb meat protein project/1. Characterisation of lamb skeletal proteome/Real run - 5 lambs from LCF/ mgf_Obj_1/Myo_4-20pc_myo_15B-17B_concat_all_the_line_delet.mgf
Protein: PREDICTED: troponin C, skeletal muscle [Ovis aries]
Accession: gi|426242099|ref|XP_004014914.1|
Parent m/z: 587.36, 2+
Sequence: M.VRTDQQAEAR.S
Score: 20.598287968701356

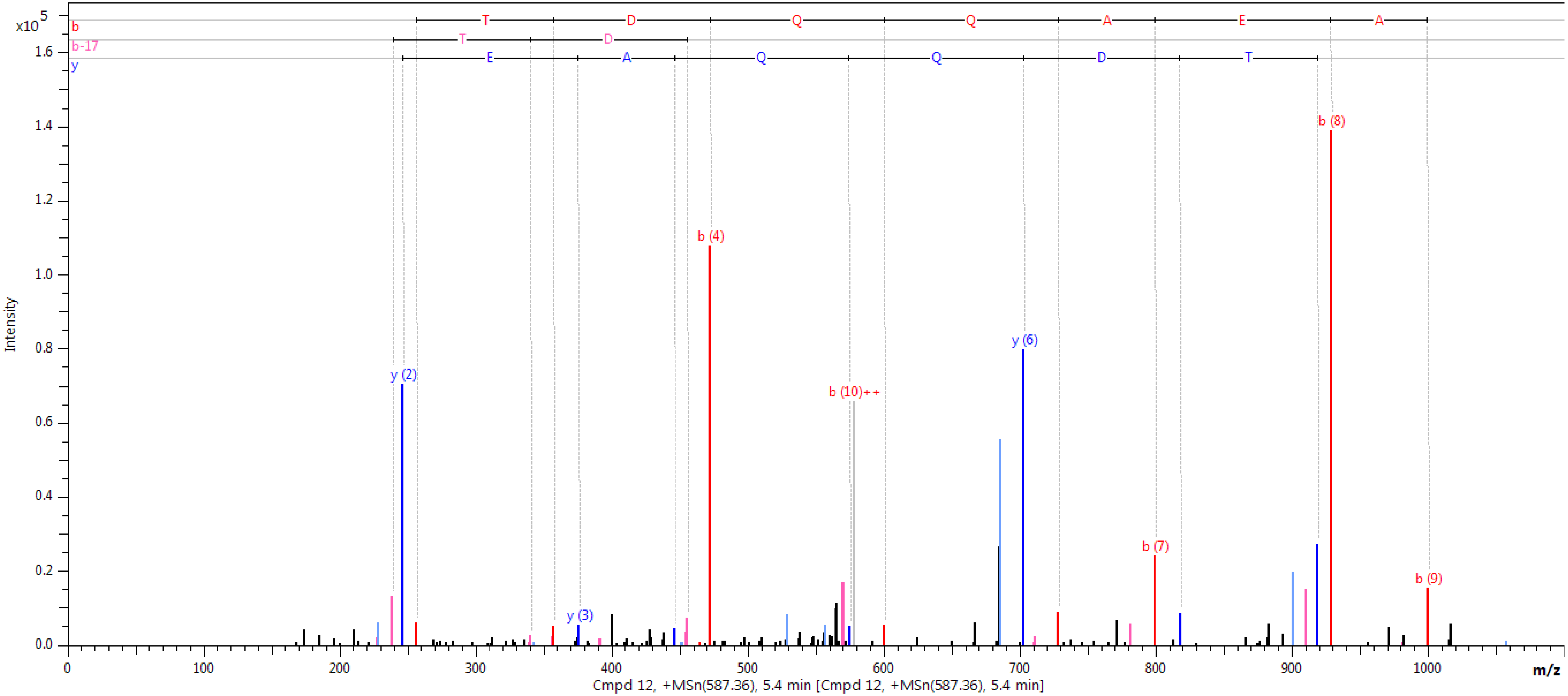

Spectrum Report

Source: M:/Documents/Lamb meat protein project/1. Characterisation of lamb skeletal proteome/Real run - 5 lambs from LCF/mgf_Obj_1/Myo_4-20pc_myo_15B-17B_concat_all_the_line_delet.mgf

Protein: beta globin chain [Ovis aries musimon]

Accession: gi|86129745|gb|ABC86524.1|

Parent m/z: 619.028, 3+

Sequence: R.HHGSEFTPVLQAEFQK.V

Score: 55.51051099337475

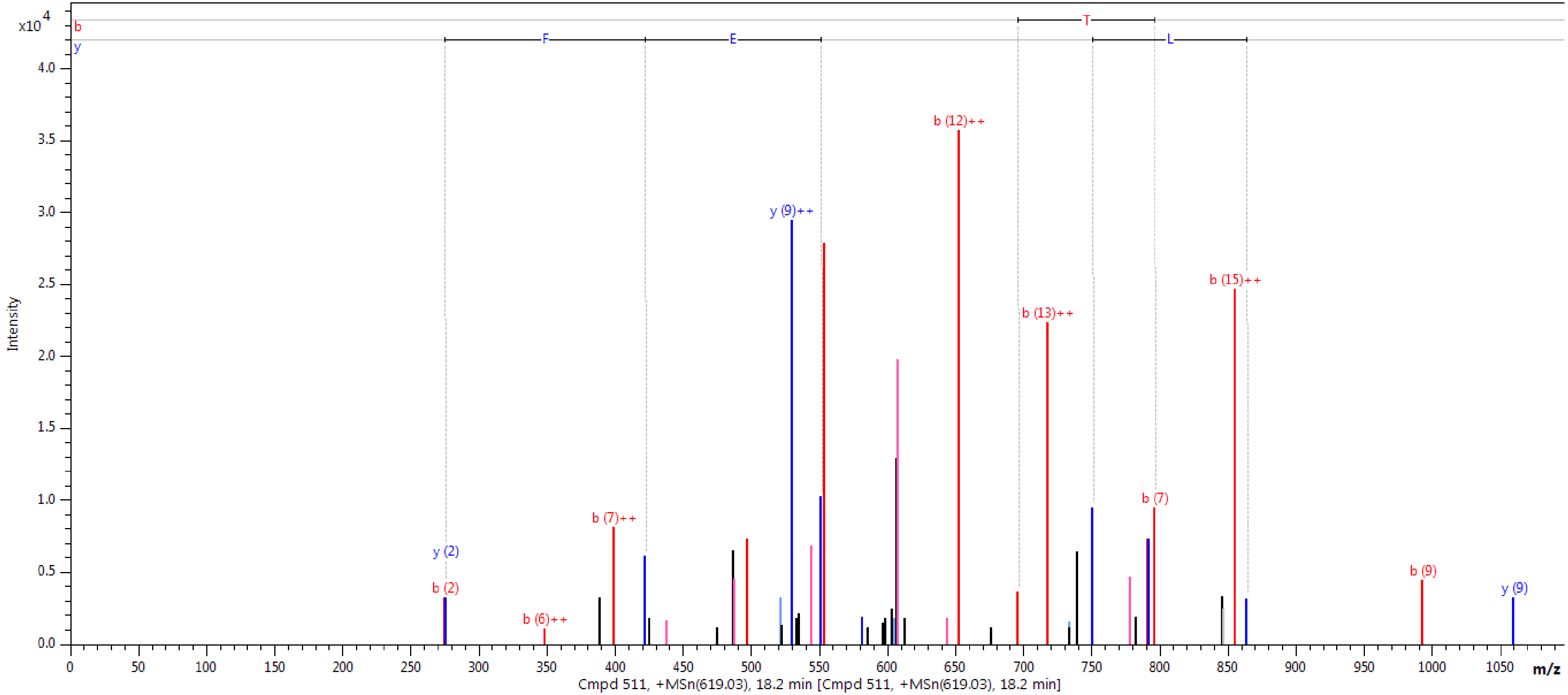

Spectrum Report

Source: M:/Documents/Lamb meat protein project/1. Characterisation of lamb skeletal proteome/Real run - 5 lambs from LCF/ mgf_Obj_1/Myo_4-20pc_myo_15B-17B_concat_all_the_line_delet.mgf
Protein: PREDICTED: tropomyosin alpha-1 chain isoform 2 [Ovis aries]
Accession: gi|426228838|ref|XP_004008503.1|
Parent m/z: 575.348, 2+
Sequence: K.MEIQEMQLK.E
Score: 30.661929300139885

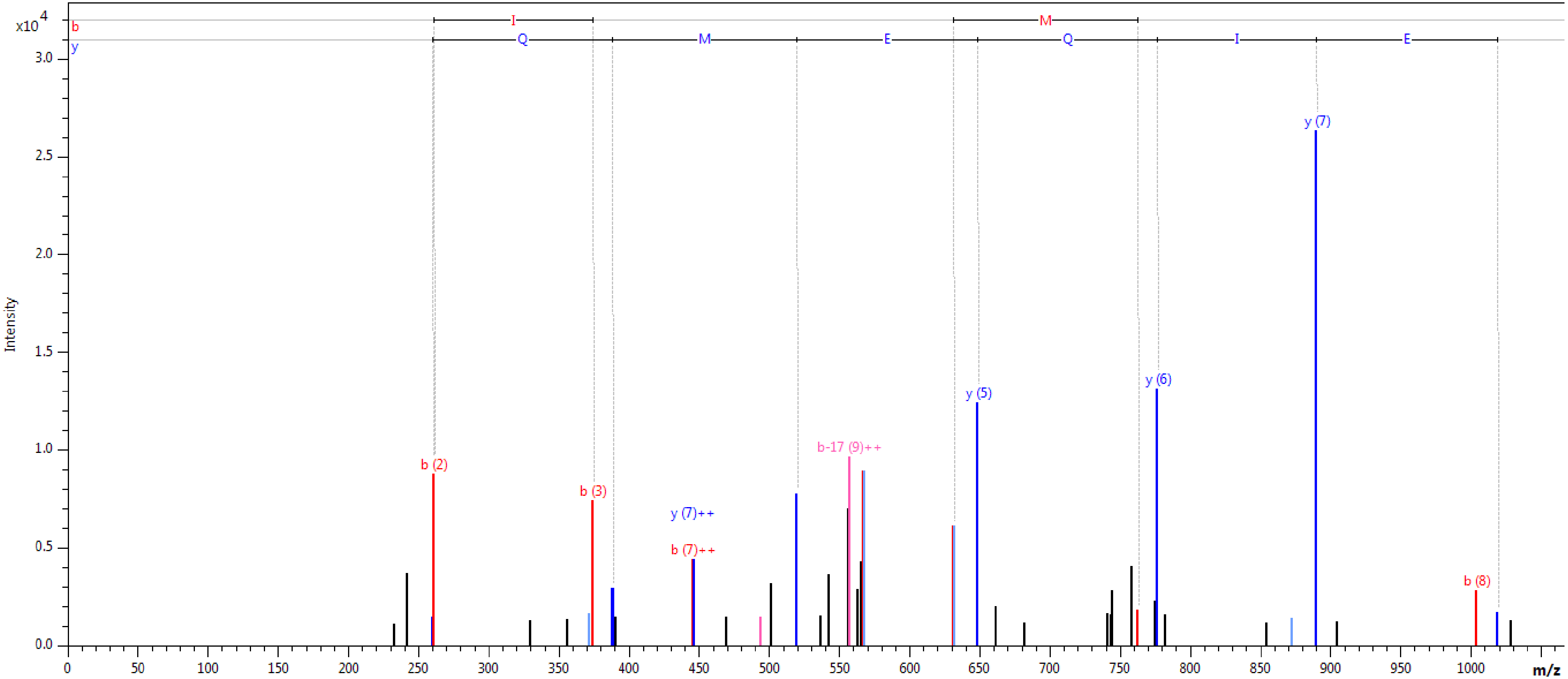

Cmpd 444, +MSn(575.35), 1042.9 s [Cmpd 444, +MSn(575.35), 1042.9 s]

Spectrum Report

Source: M:/Documents/Lamb meat protein project/1. Characterisation of lamb skeletal proteome/Real run - 5 lambs from LCF/ mgf_Obj_1/Myo_4-20pc_myo_15B-17B_concat_all_the_line_delet.mgf
Protein: thioredoxin [Ovis aries]
Accession: gi|397947|emb|CAA81083.1|
Sequence: K.YAFQEALNSAGEK.L
Parent m/z: 714.378, 2+
Score: 134.55437141930616

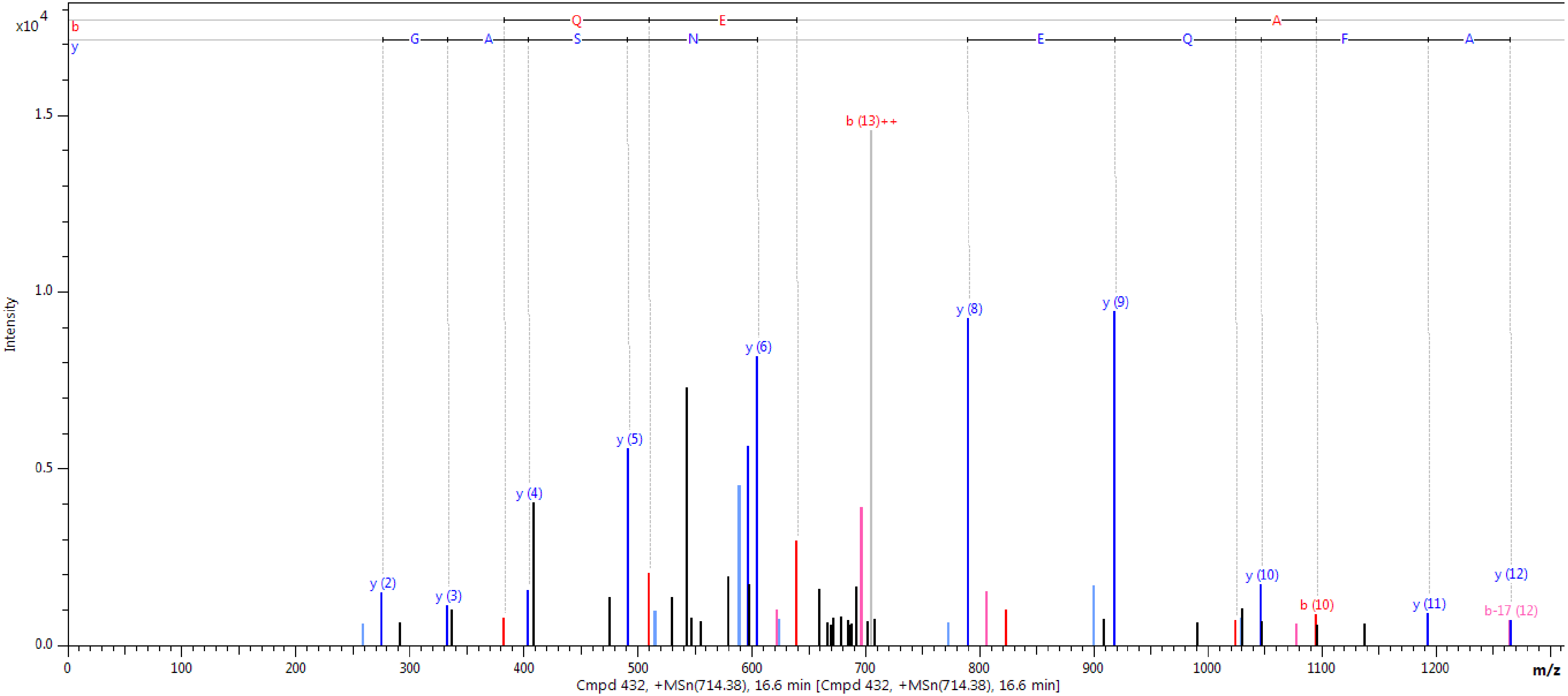

agresearch

Spectrum Report

Source: M:/Documents/Lamb meat protein project/1. Characterisation of lamb skeletal proteome/Real run - 5 lambs from LCF/mgf_Obj_1/Myo_4-20pc_myo_15B-17B_concat_all_the_line_delet.mgf

Protein: galectin-1 [Ovis aries]

Accession: gi|47779226|gb|AAT38511.1|

Parent m/z: 854.984, 2+

Sequence: R.LNLEAINYLAAGGDFK.I

Score: 154.23115257800333

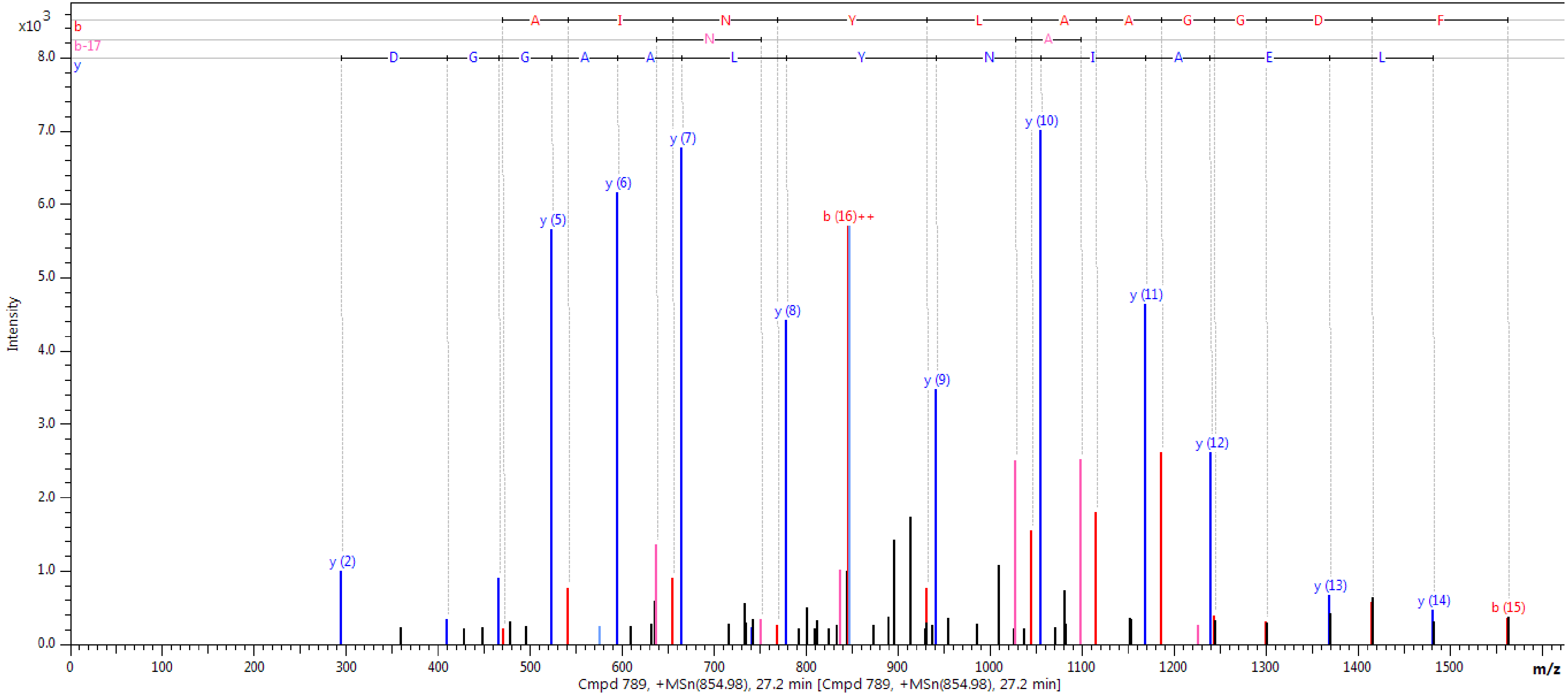

Source: M:/Documents/Lamb meat protein project/1. Characterisation of lamb skeletal proteome/Real run - 5 lambs from LCF/mgf_Obj_1/Myo_4-20pc_myo_15B-17B_concat_all_the_line_delet.mgf
Protein: PREDICTED: probable C->U-editing enzyme APOBEC-2 [Ovis aries]
Accession: gi|426250247|ref|XP_004018849.1| **Parent m/z:** 729.905, 2+
Sequence: K.TFLCYVVEAQSK.G **Score:** 137.87380979846483

Modification: Propionamide: 4

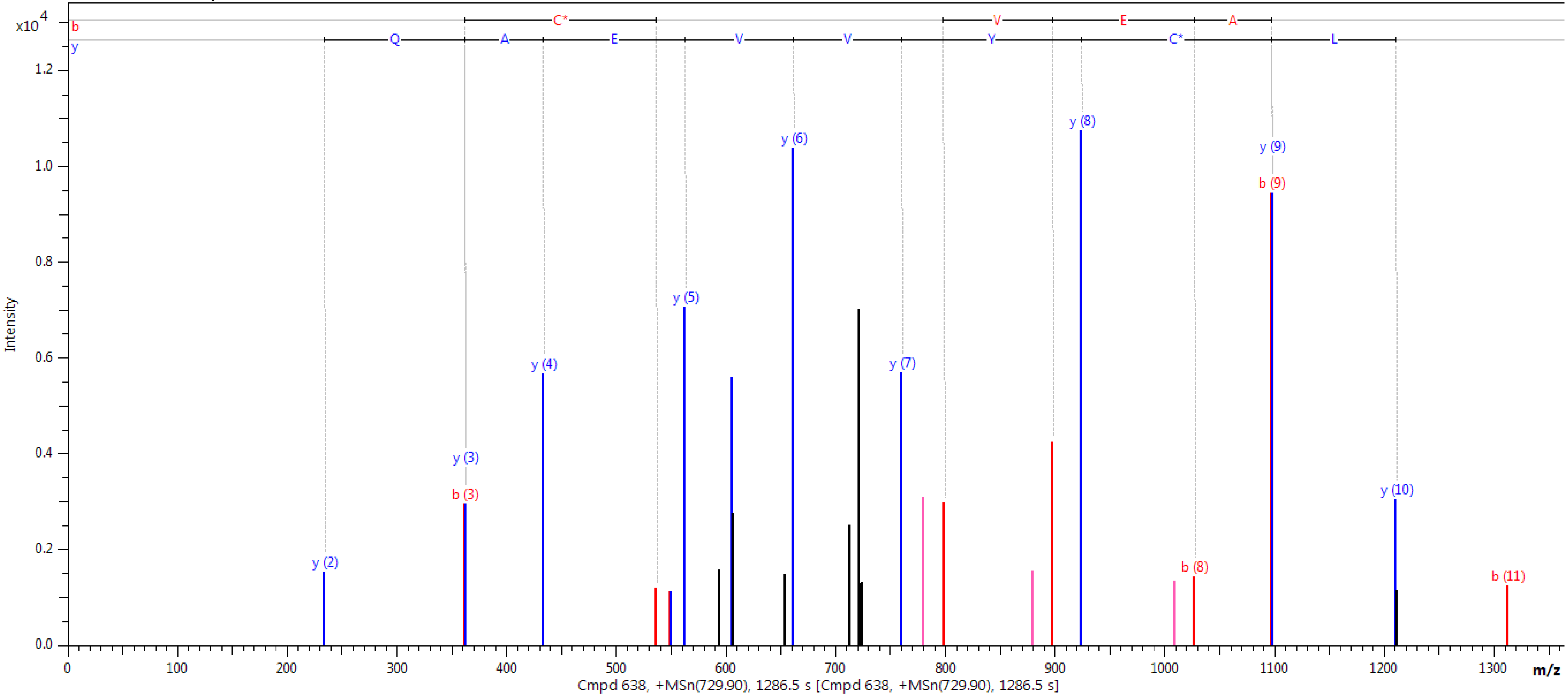

Spectrum Report

Source: M:/Documents/Lamb meat protein project/1. Characterisation of lamb skeletal proteome/Real run - 5 lambs from LCF/mgf_Obj_1/Myo_4-20pc_myo_15B-17B_concat_all_the_line_delet.mgf
Protein: PREDICTED: calcium/calmodulin-dependent protein kinase type II subunit alpha isoform 2 [Ovis aries]
Accession: gi|426229902|ref|XP_004009022.1|
Parent m/z: 652.356, 2+
Sequence: R.ITQYLDAGGIPR.T
Score: 138.55369758007762

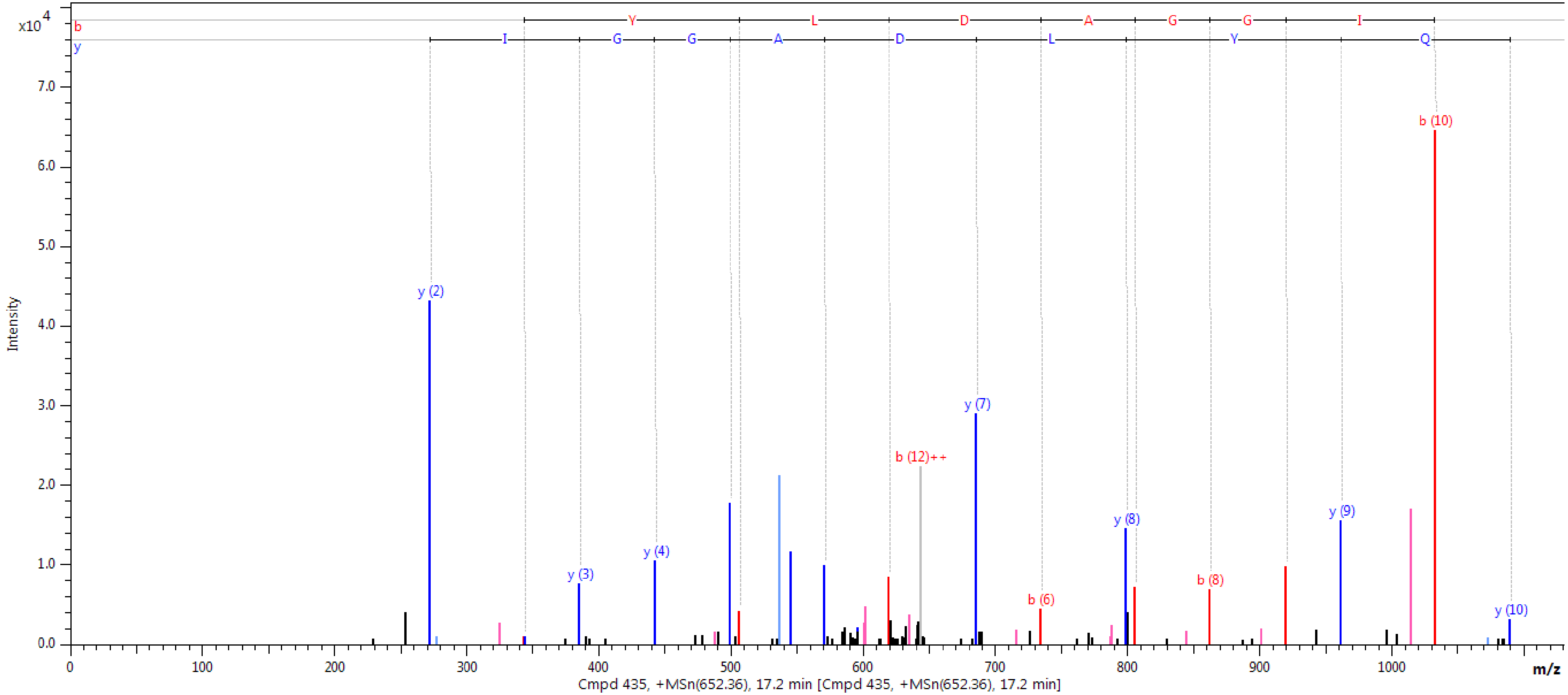

Spectrum Report

Source: M:/Documents/Lamb meat protein project/1. Characterisation of lamb skeletal proteome/Real run - 5 lambs from LCF/mgf_Obj_1/Myo_4-20pc_myo_15B-17B_concat_all_the_line_delet.mgf
Protein: PREDICTED: LOW QUALITY PROTEIN: phosphorylase b kinase regulatory subunit alpha, skeletal muscle isoform [Ovis aries
Accession: gi|426257240|ref|XP_004022240.1| **Parent m/z:** 802.477, 2+
Sequence: K.AALEALDELDLFGVK.G **Score:** 154.23115257800333

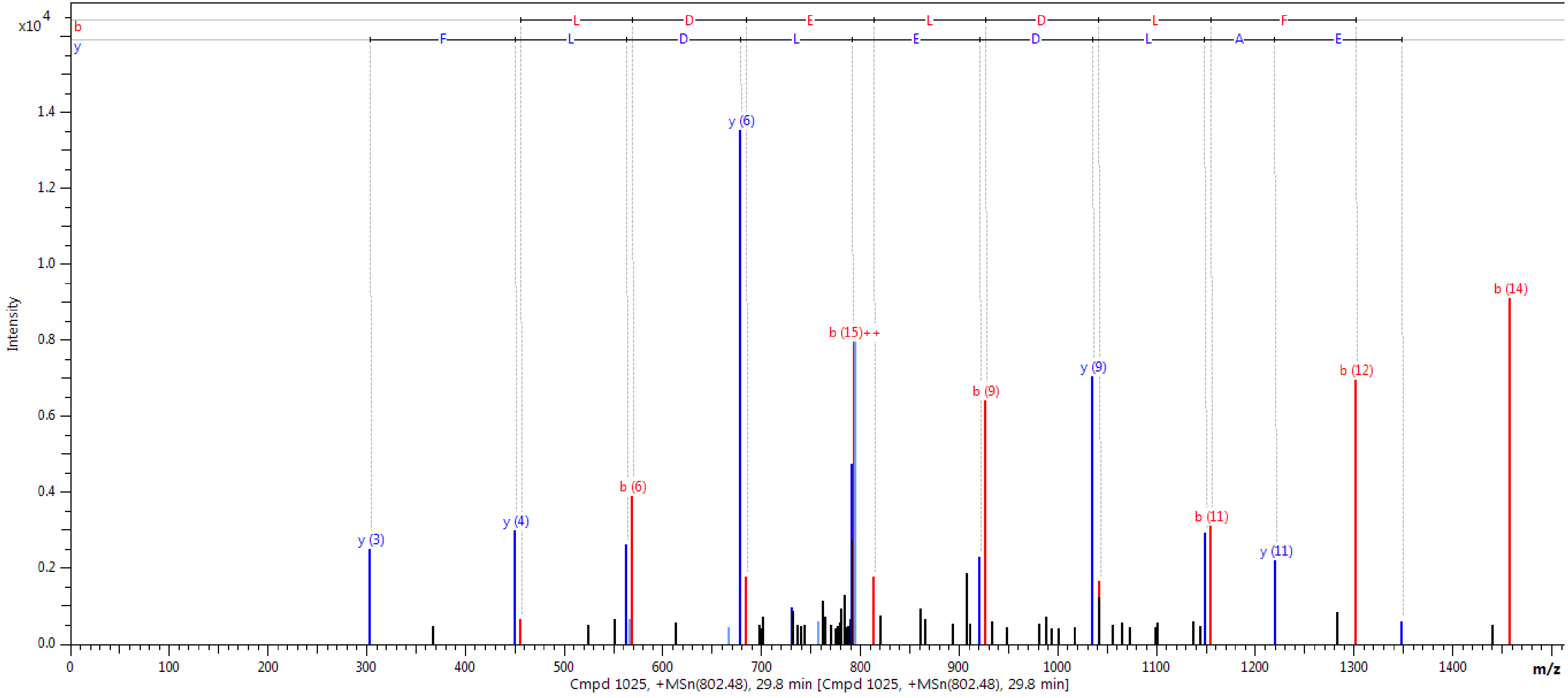

Spectrum Report

Source: M:/Documents/Lamb meat protein project/1. Characterisation of lamb skeletal proteome/Real run - 5 lambs from LCF/mgf_Obj_1/Myo_4-20pc_myo_15B-17B_concat_all_the_line_delet.mgf
Protein: PREDICTED: LOW QUALITY PROTEIN: prelamin-A/C [Ovis aries]
Accession: gi|426219007|ref|XP_004003722.1| **Parent m/z:** 770.348, 2+
Sequence: R.SVGGSGGGSFGDSLVTR.S **Score:** 28.329545536906554

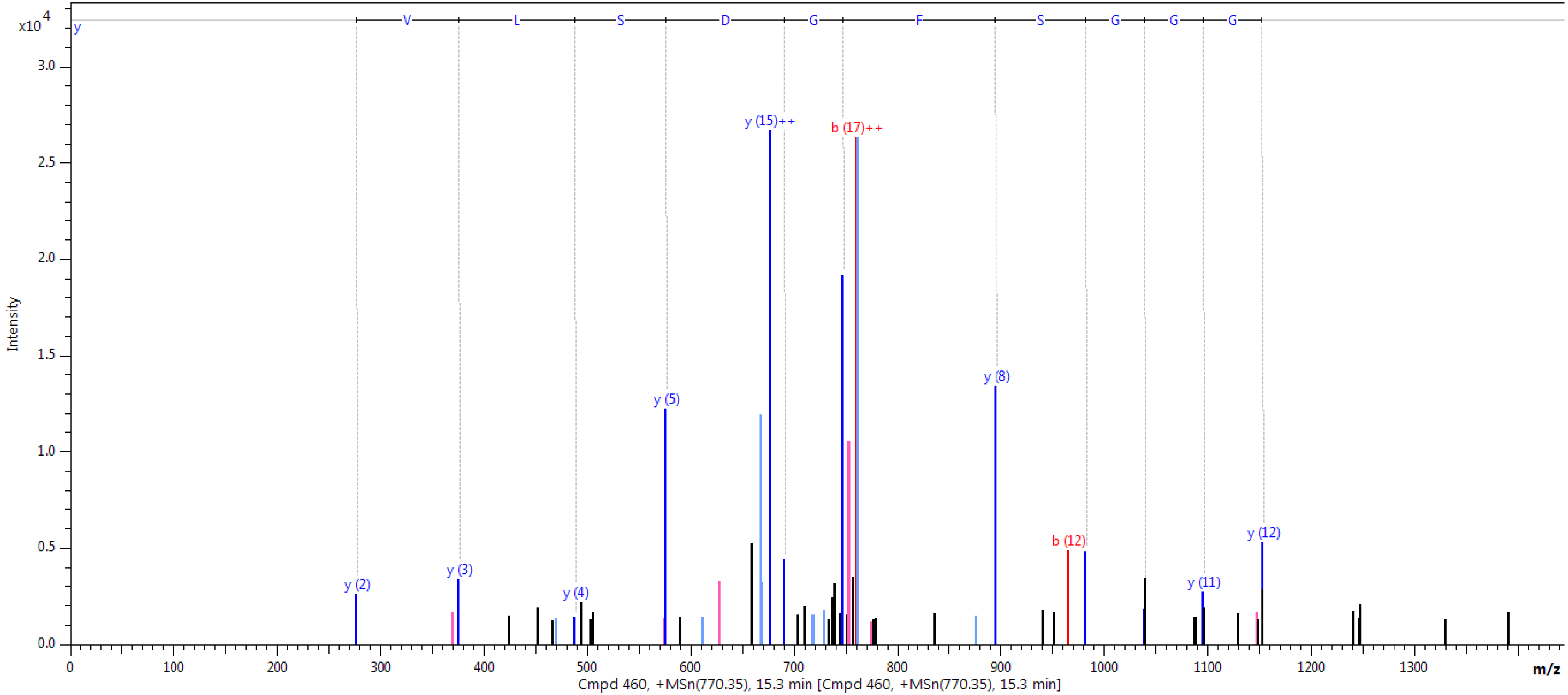

Spectrum Report

Source: M:/Documents/Lamb meat protein project/1. Characterisation of lamb skeletal proteome/Real run - 5 lambs from LCF/ mgf_Obj_1/Myo_4-20pc_myo_15B-17B_concat_all_the_line_delet.mgf
Protein: PREDICTED: calcium/calmodulin-dependent protein kinase type II subunit delta isoform 1 [Ovis aries]
Accession: gi|426231239|ref|XP_004009647.1| **Parent m/z:** 809.915, 2+
Sequence: R.FTDEYQLFEELGK.G **Score:** 145.44777391929938

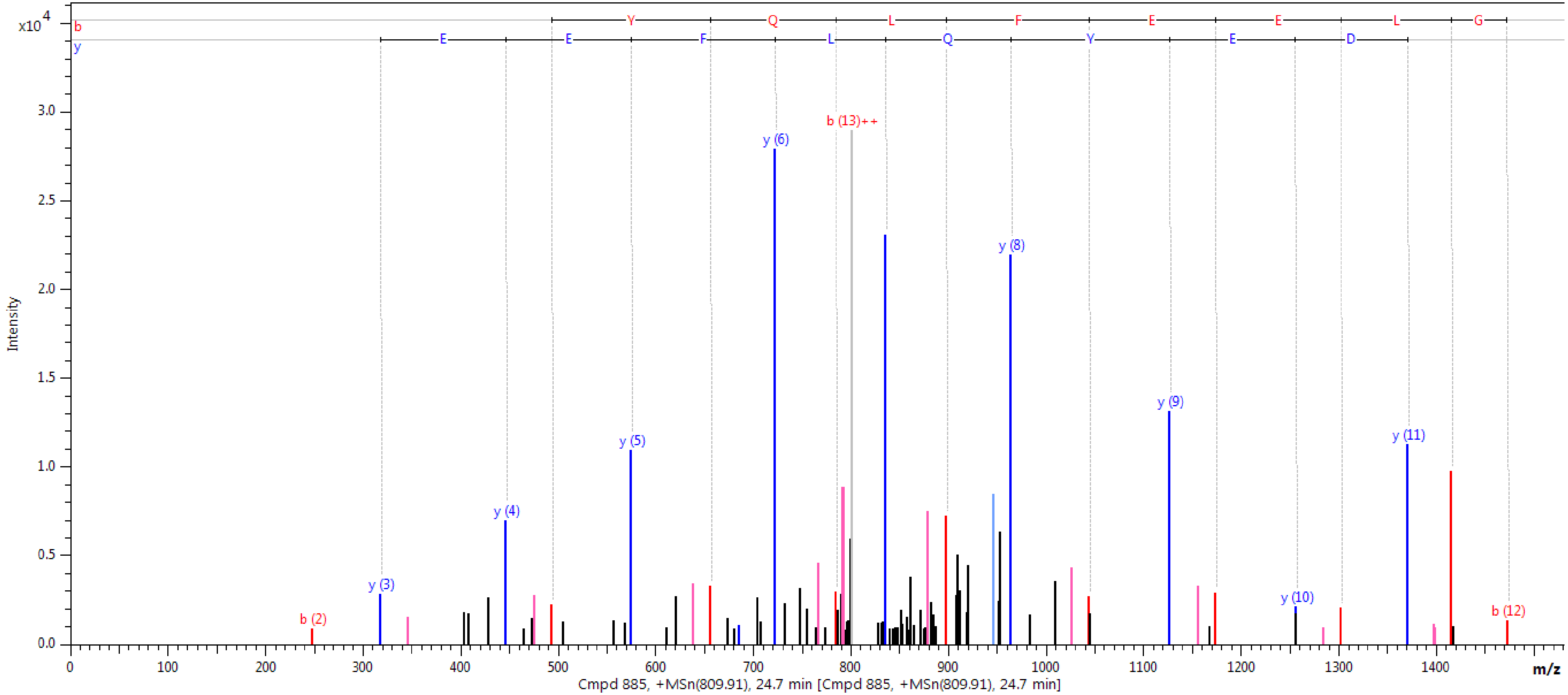

Spectrum Report

Source: M:/Documents/Lamb meat protein project/1. Characterisation of lamb skeletal proteome/Real run - 5 lambs from LCF/mgf_Obj_1/Myo_4-20pc_myo_15B-17B_concat_all_the_line_delet.mgf
Protein: PREDICTED: CDGSH iron-sulfur domain-containing protein 1-like [Ovis aries]
Accession: gi|426253273|ref|XP_004020323.1| **Parent m/z:** 818.481, 2+
Sequence: K.HNEETGDNVGPLIIK.K **Score:** 112.27140893042842

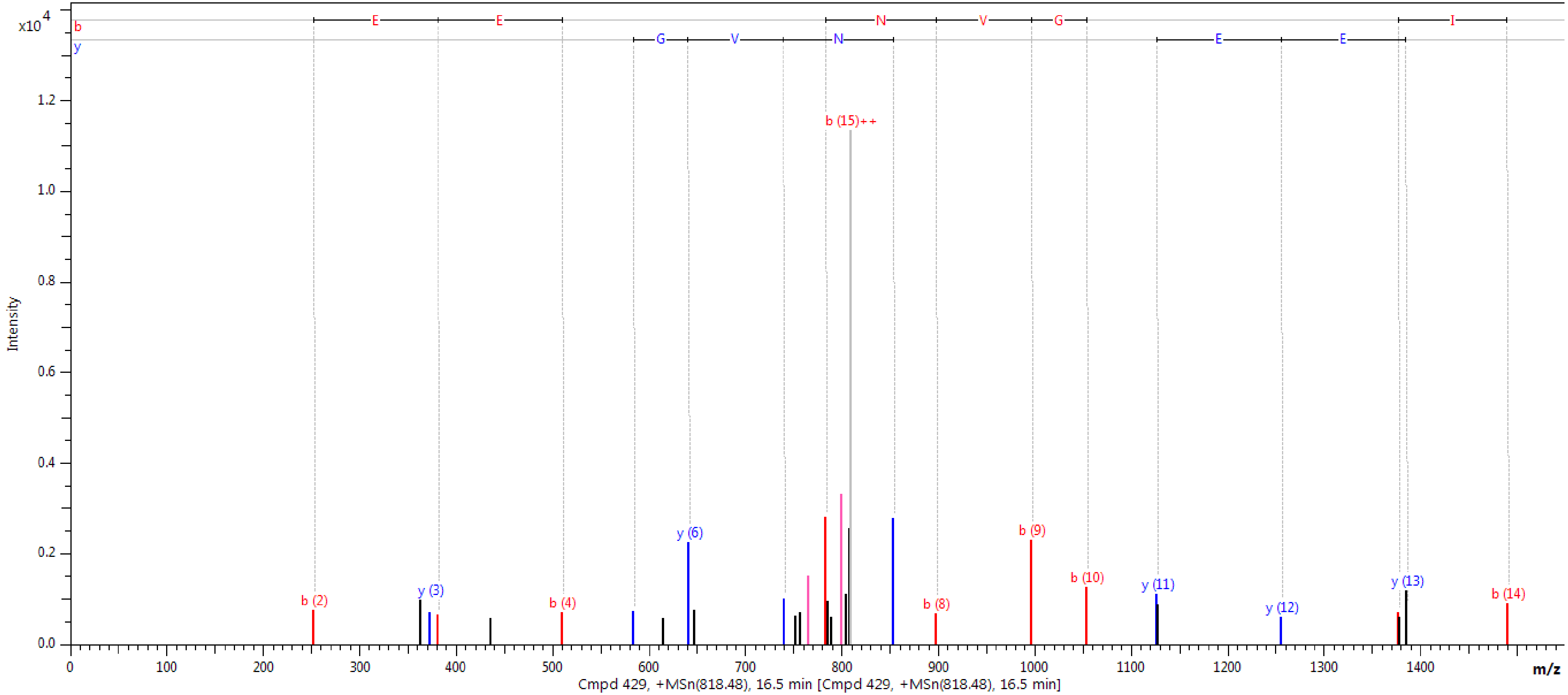

Spectrum Report

Source: M:/Documents/Lamb meat protein project/1. Characterisation of lamb skeletal proteome/Real run - 5 lambs from LCF/mgf_Obj_1/Myo_4-20pc_myo_15B-17B_concat_all_the_line_delet.mgf
Protein: myozenin 3 [Ovis aries]
Accession: gi|312144740|gb|ADQ28100.1|
Sequence: K.TPVPFGGTLVGETLPR.A
Parent m/z: 820.985, 2+
Score: 154.23115257800333

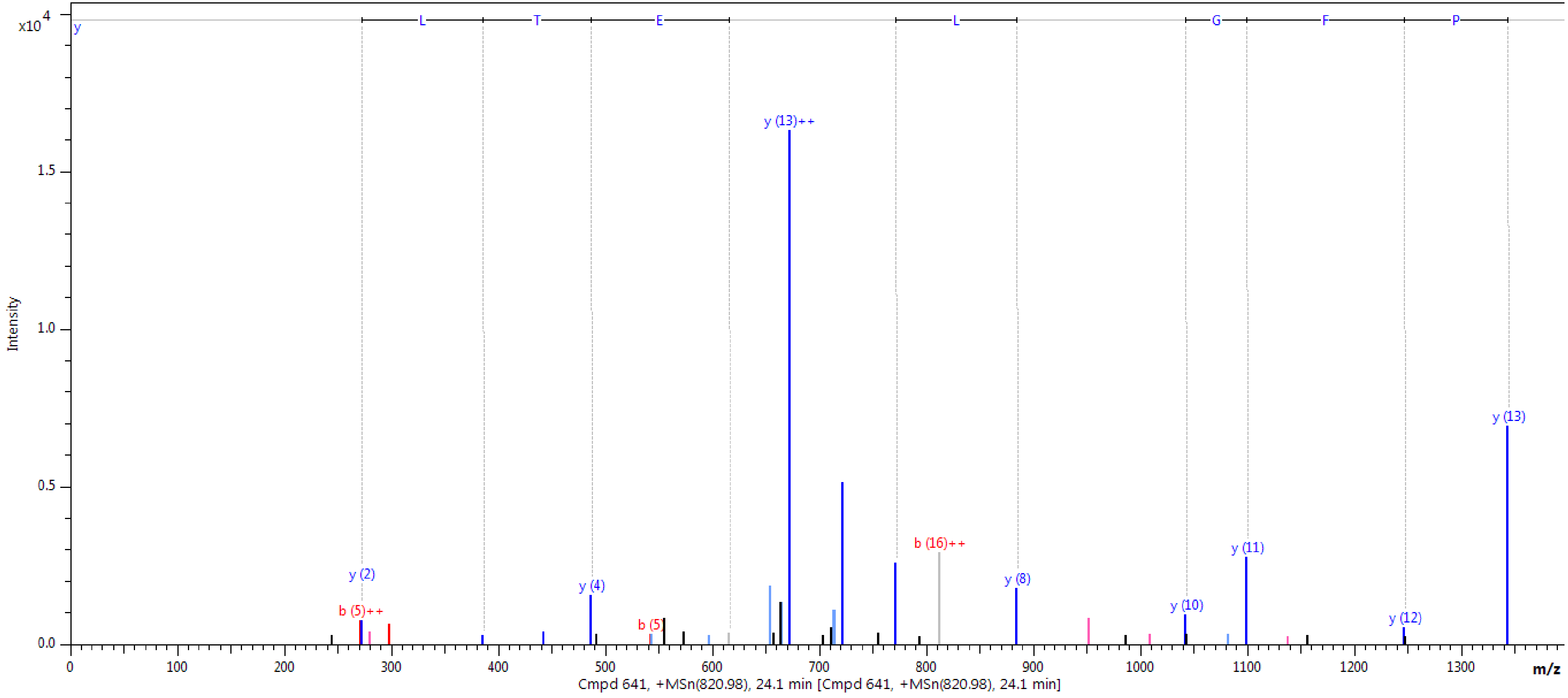

Spectrum Report

Source: M:/Documents/Lamb meat protein project/1. Characterisation of lamb skeletal proteome/Real run - 5 lambs from LCF/mgf_Obj_1/Myo_4-20pc_myo_15B-17B_concat_all_the_line_delet.mgf
Protein: PREDICTED: LOW QUALITY PROTEIN: filamin-B [Ovis aries]
Accession: gi|426249347|ref|XP_004018411.1|
Parent m/z: 759.922, 2+
Sequence: Q.LENVSVALEFLDR.E
Score: 66.33160693294823

Modification: Methyl: 12

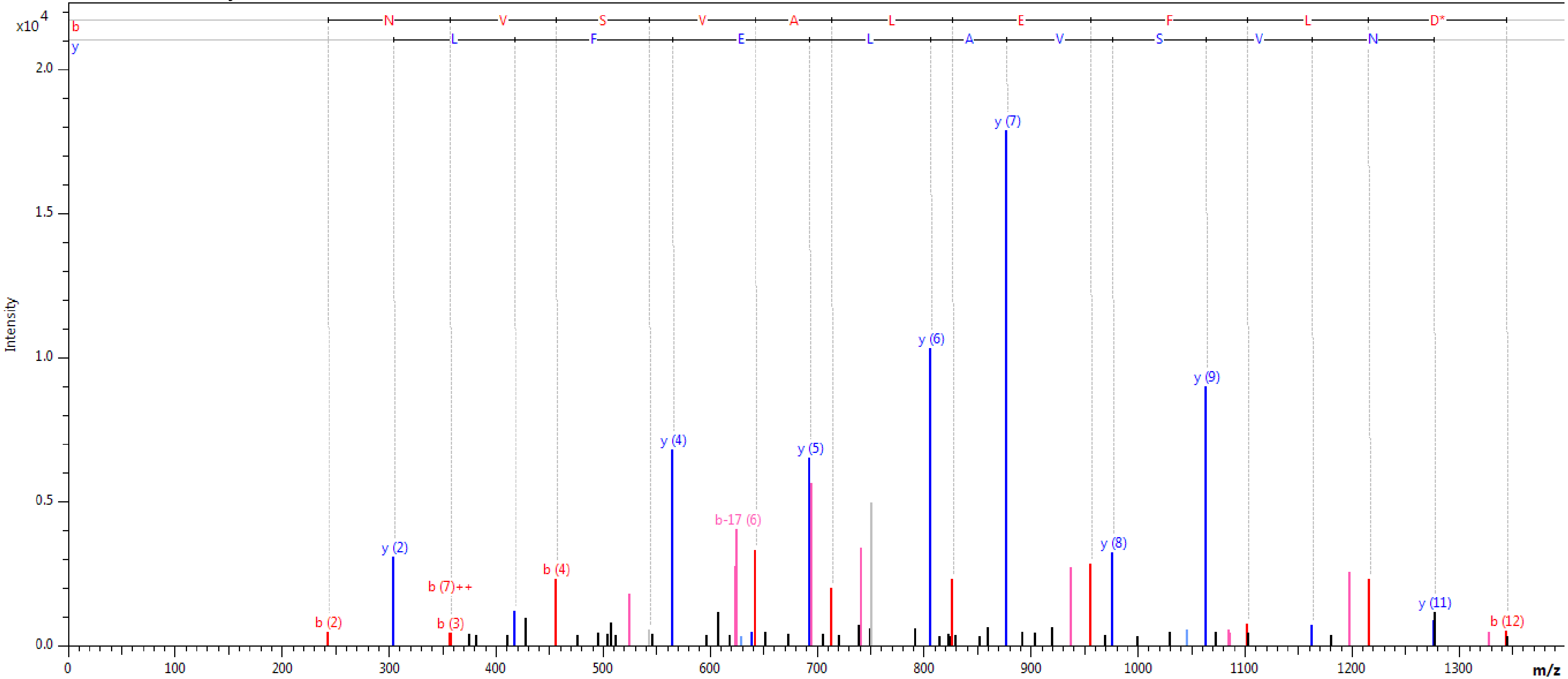

Cmpd 919, +MSn(759.92), 28.0 min [Cmpd 919, +MSn(759.92), 28.0 min]

Spectrum Report

Source: M:/Documents/Lamb meat protein project/1. Characterisation of lamb skeletal proteome/Real run - 5 lambs from LCF/ mgf_Obj_1/Myo_4-20pc_myo_15B-17B_concat_all_the_line_delet.mgf
Protein: HSP10 [Ovis aries]
Accession: gi|405113094|gb|AFR90222.1|
Sequence: K.VLQATVVAVGSGSK.G
Parent m/z: 658.477, 2+
Score: 104.29882222977454

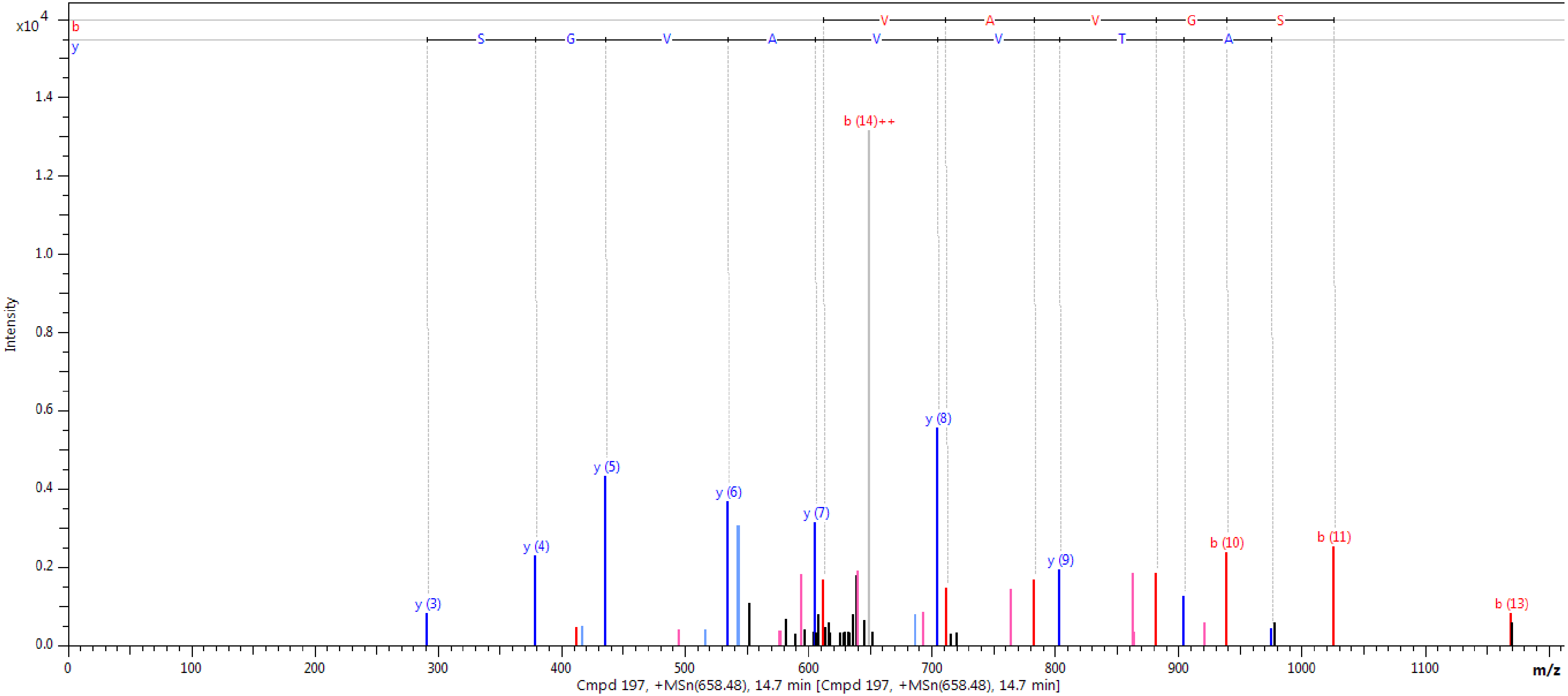

Spectrum Report

Source: M:/Documents/Lamb meat protein project/1. Characterisation of lamb skeletal proteome/Real run - 5 lambs from LCF/ mgf_Obj_1/Myo_4-20pc_myo_15B-17B_concat_all_the_line_delet.mgf
Protein: SLC25A3 [Ovis aries]
Accession: gi|213688914|gb|ACJ53940.1|
Sequence: R.IQTQPGYANTLR.D
Parent m/z: 681.376, 2+
Score: 55.89141818302666

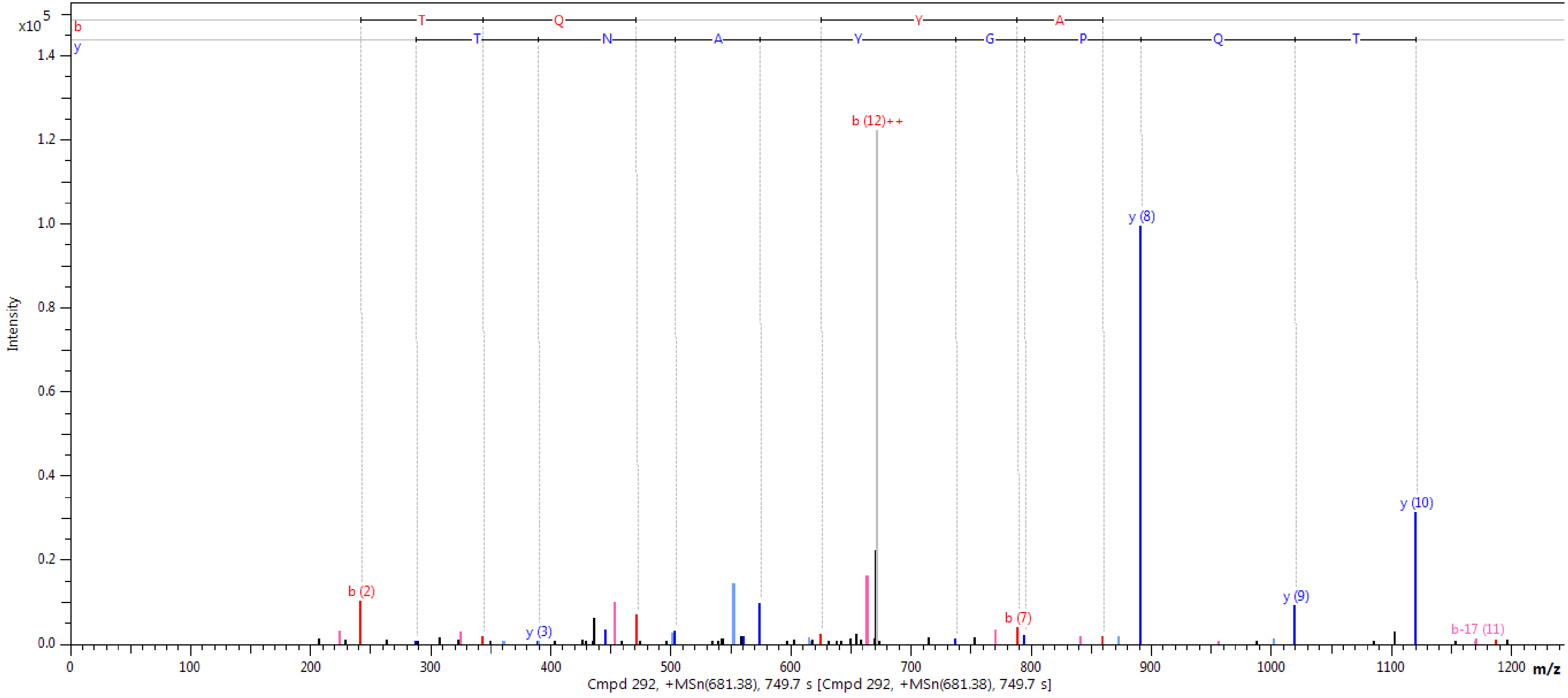

Spectrum Report

Source: M:/Documents/Lamb meat protein project/1. Characterisation of lamb skeletal proteome/Real run - 5 lambs from LCF/mgf_Obj_1/Myo_4-20pc_myo_15B-17B_concat_all_the_line_delet.mgf
Protein: PREDICTED: barrier-to-autointegration factor [Ovis aries]
Accession: gi|426252060|ref|XP_004019736.1|
Parent m/z: 675.351, 2+
Sequence: K.AYVVLGQFLVLK.K
Score: 55.89141818302666

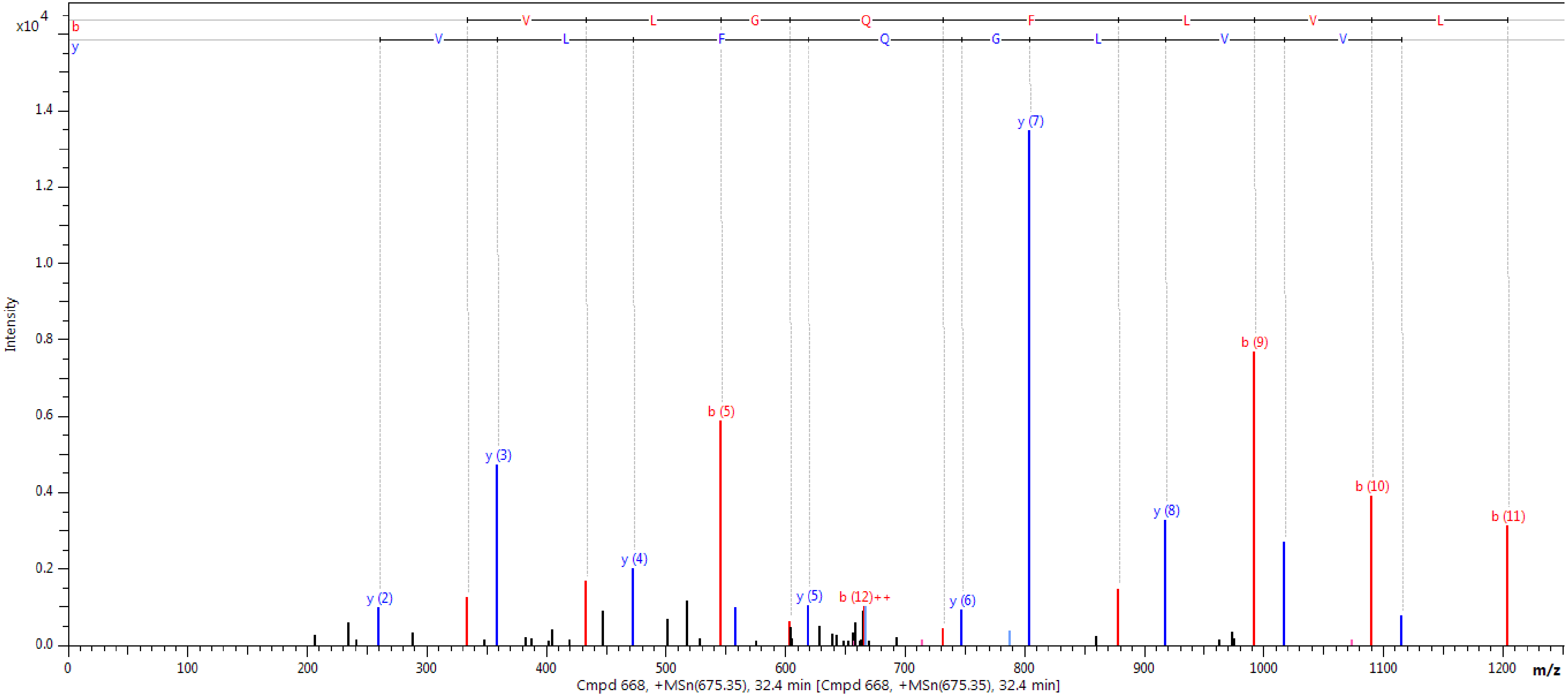

Spectrum Report

Source: M:/Documents/Lamb meat protein project/1. Characterisation of lamb skeletal proteome/Real run - 5 lambs from LCF/ mgf_Obj_1/Myo_4-20pc_myo_15B-17B_concat_all_the_line_delet.mgf
Protein: PREDICTED: lumican [Ovis aries]
Accession: gi|426224280|ref|XP_004006300.1|
Sequence: K.LPSGLPVSLLTLYLDNNK.I
Parent m/z: 979.079, 2+
Score: 94.40506330452504

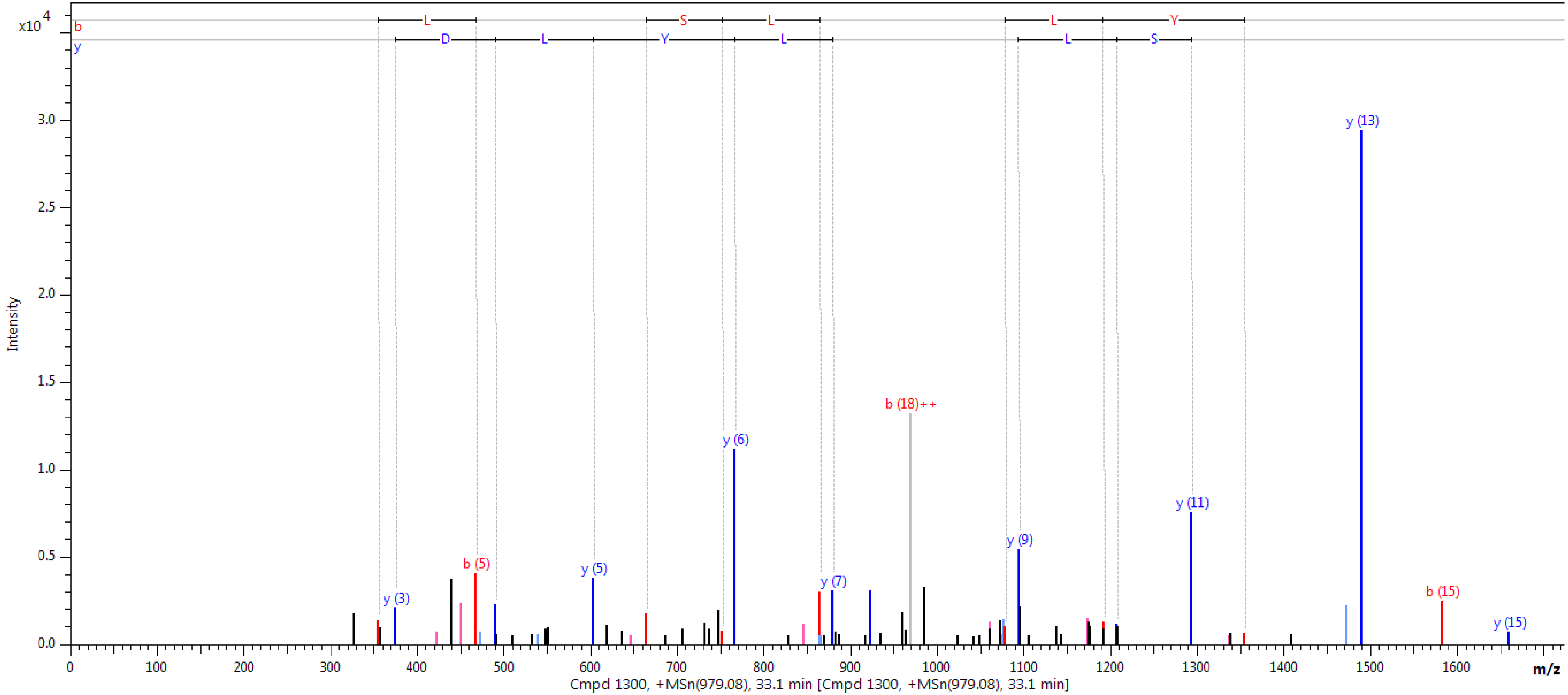

Spectrum Report

Source: M:/Documents/Lamb meat protein project/1. Characterisation of lamb skeletal proteome/Real run - 5 lambs from LCF/mgf_Obj_1/Myo_4-20pc_myo_15B-17B_concat_all_the_line_delet.mgf

Protein: PREDICTED: SH3 domain-binding glutamic acid-rich protein [Ovis aries]

Accession: gi|426218329|ref|XP_004003401.1|

Parent m/z: 654.398, 2+

Sequence: K.VFVATSSGSIAIR.K

Score: 55.89141818302666

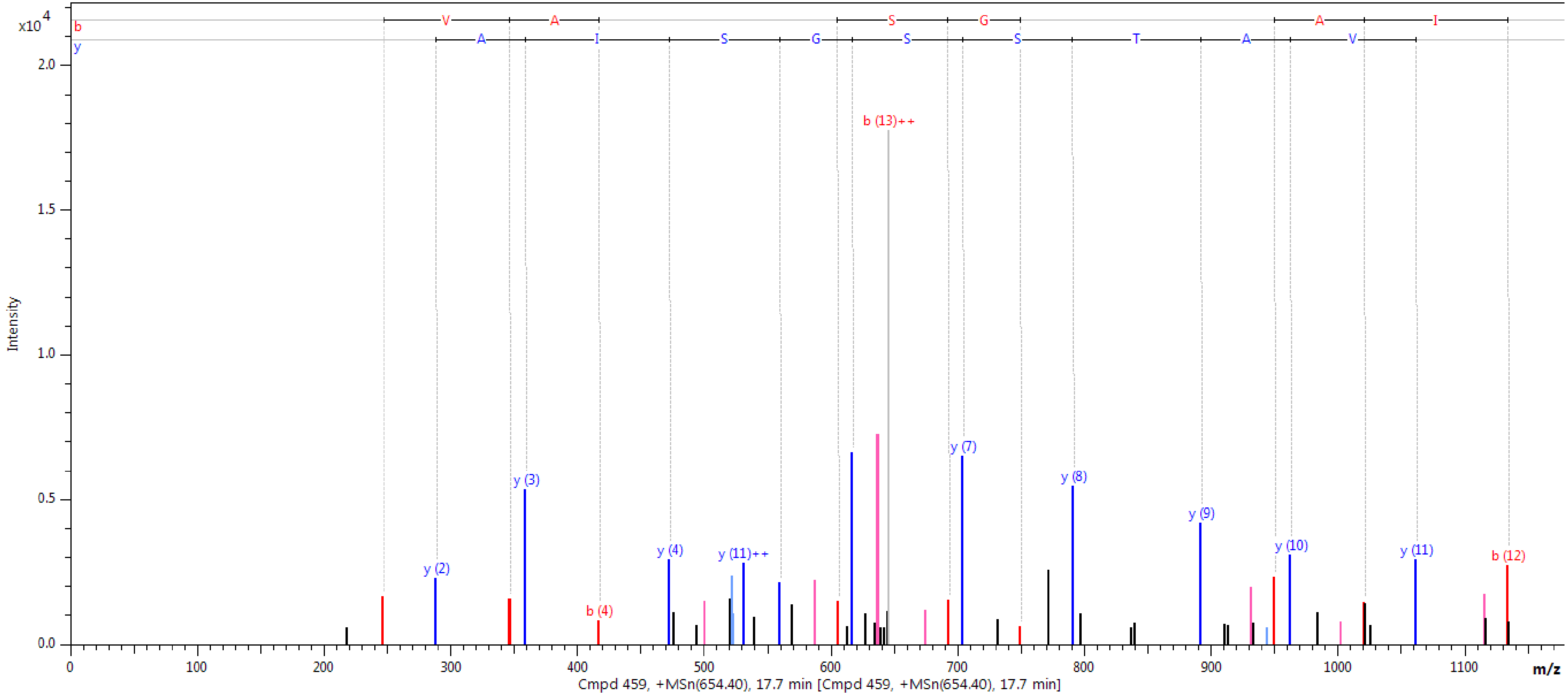

Spectrum Report

Source: M:/Documents/Lamb meat protein project/1. Characterisation of lamb skeletal proteome/Real run - 5 lambs from LCF/mgf_Obj_1/Myo_4-20pc_myo_15B-17B_concat_all_the_line_delet.mgf
Protein: PREDICTED: pyruvate dehydrogenase protein X component-like isoform 2 [Ovis aries]
Accession: gi|426245306|ref|XP_004016454.1|
Parent m/z: 509.291, 2+
Sequence: R.VVDDELATR.F
Score: 55.89141818302666

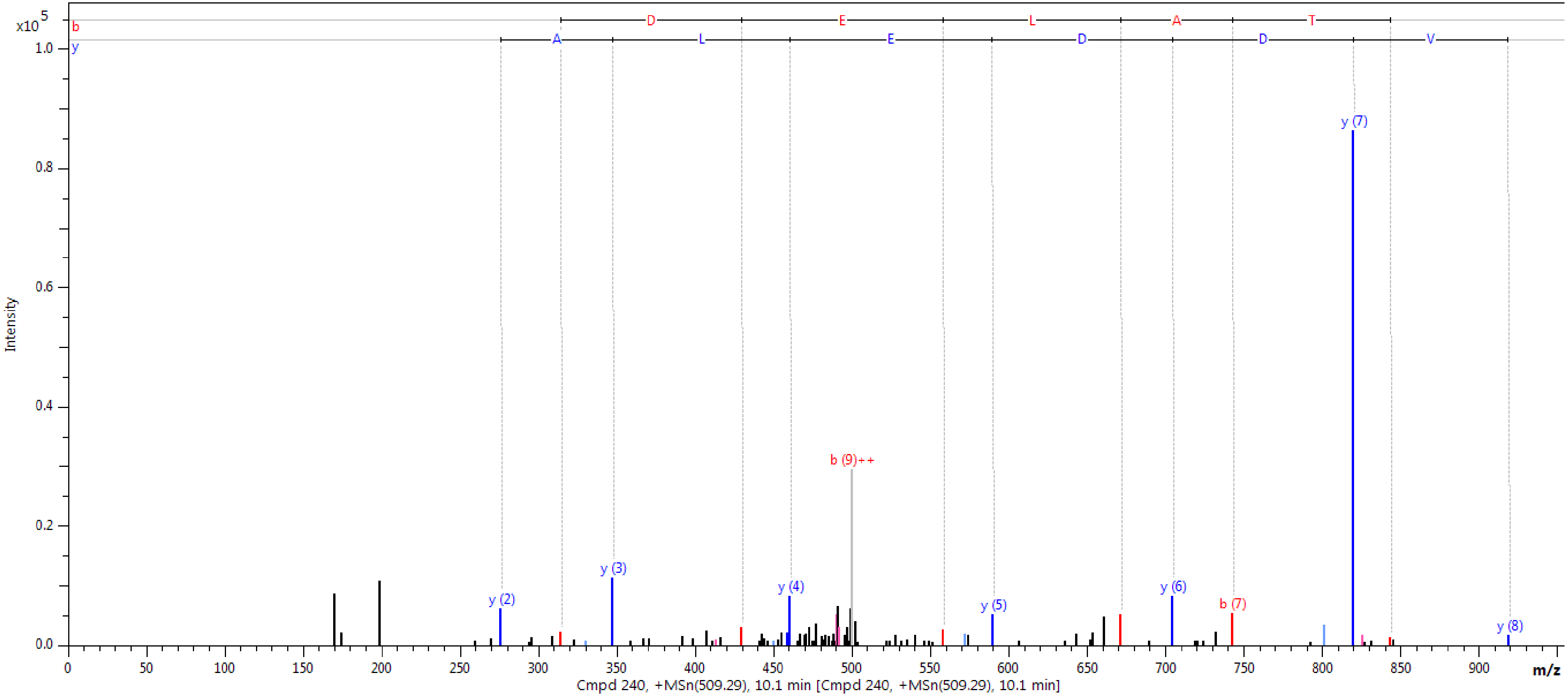

Spectrum Report

Source: M:/Documents/Lamb meat protein project/1. Characterisation of lamb skeletal proteome/Real run - 5 lambs from LCF/mgf_Obj_1/Myo_4-20pc_myo_15B-17B_concat_all_the_line_delet.mgf

Protein: PREDICTED: NADH dehydrogenase [ubiquinone] iron-sulfur protein 8, mitochondrial [Ovis aries]

Accession: gi|426252584|ref|XP_004019986.1|

Parent m/z: 707.928, 2+

Sequence: R.AAQTLLWTELIR.G

Score: 55.89141818302666

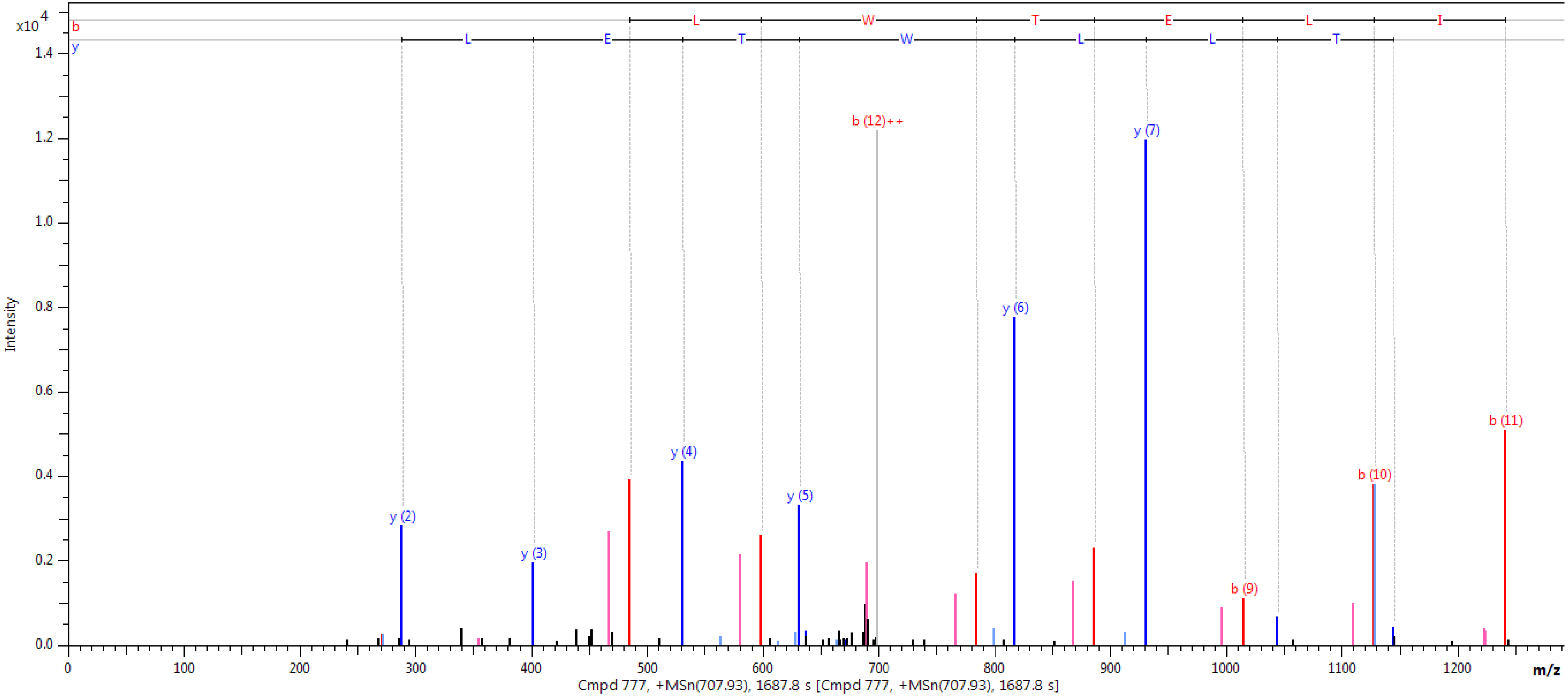

Spectrum Report

Source: M:/Documents/Lamb meat protein project/1. Characterisation of lamb skeletal proteome/Real run - 5 lambs from LCF/ mgf_Obj_1/Myo_4-20pc_myo_15B-17B_concat_all_the_line_delet.mgf
Protein: PREDICTED: cytochrome c oxidase subunit 6A2, mitochondrial-like [Ovis aries]
Accession: gi|426254623|ref|XP_004020976.1|
Parent m/z: 607.912, 2+
Sequence: R.VNPLPTGYEKP.-
Score: 55.89141818302666

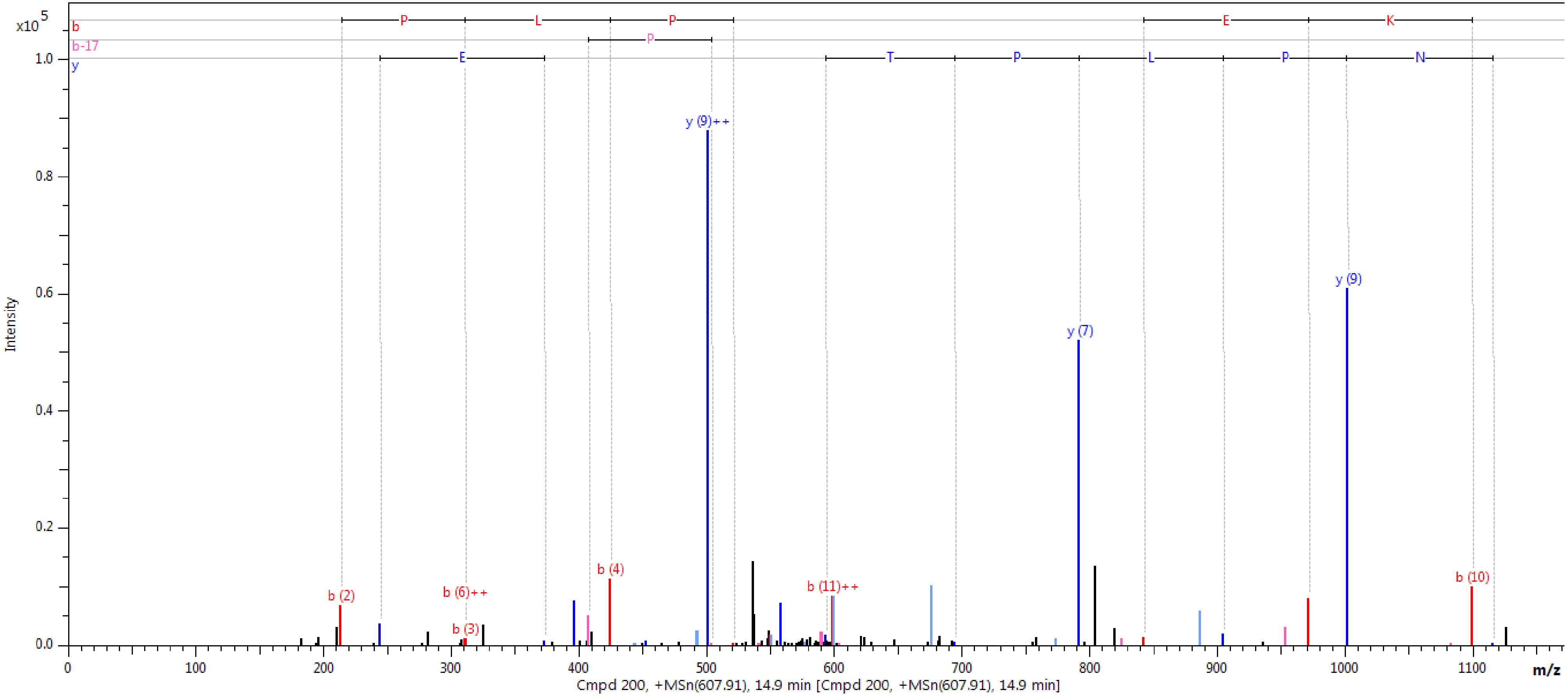

Spectrum Report

Source: M:/Documents/Lamb meat protein project/1. Characterisation of lamb skeletal proteome/Real run - 5 lambs from LCF/mgf_Obj_1/Myo_4-20pc_myo_15B-17B_concat_all_the_line_delet.mgf
Protein: homologue to Acylphosphatase-2, partial [Ovis aries: Oar v3]
Accession: gi|1999009905|gb|1999009905.1|
Parent m/z: 665.376, 2+
Sequence: K.GTVTGQVQGPEEK.V
Score: 55.89141818302666

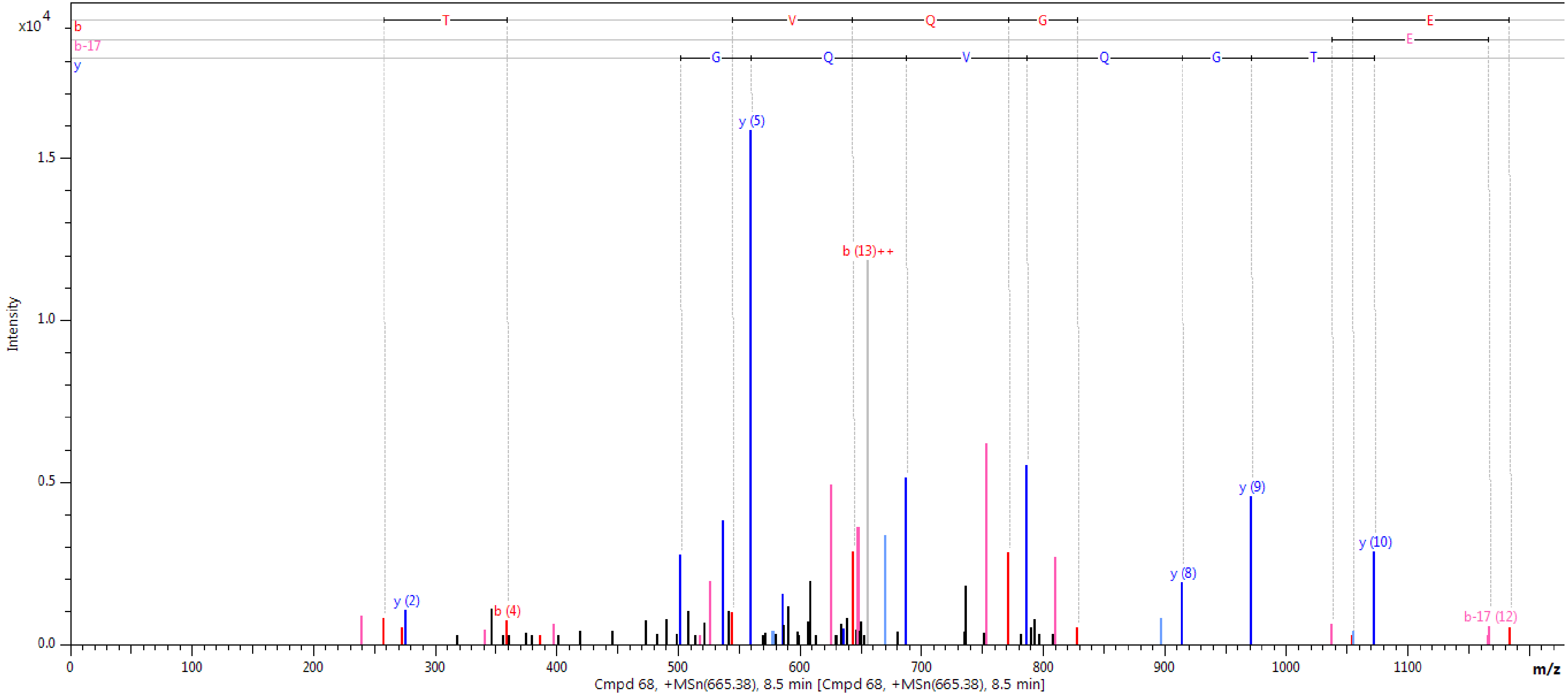

agresearch

Spectrum Report

Source: M:/Documents/Lamb meat protein project/1. Characterisation of lamb skeletal proteome/Real run - 5 lambs from LCF/mgf_Obj_1/Myo_4-20pc_myo_15B-17B_concat_all_the_line_delet.mgf
Protein: PREDICTED: glycogen [starch] synthase, liver [Ovis aries]
Accession: gi|426225378|ref|XP_004006843.1|
Parent m/z: 589.883, 2+
Sequence: K.VGGIYTVIQTK.A
Score: 55.89141818302666

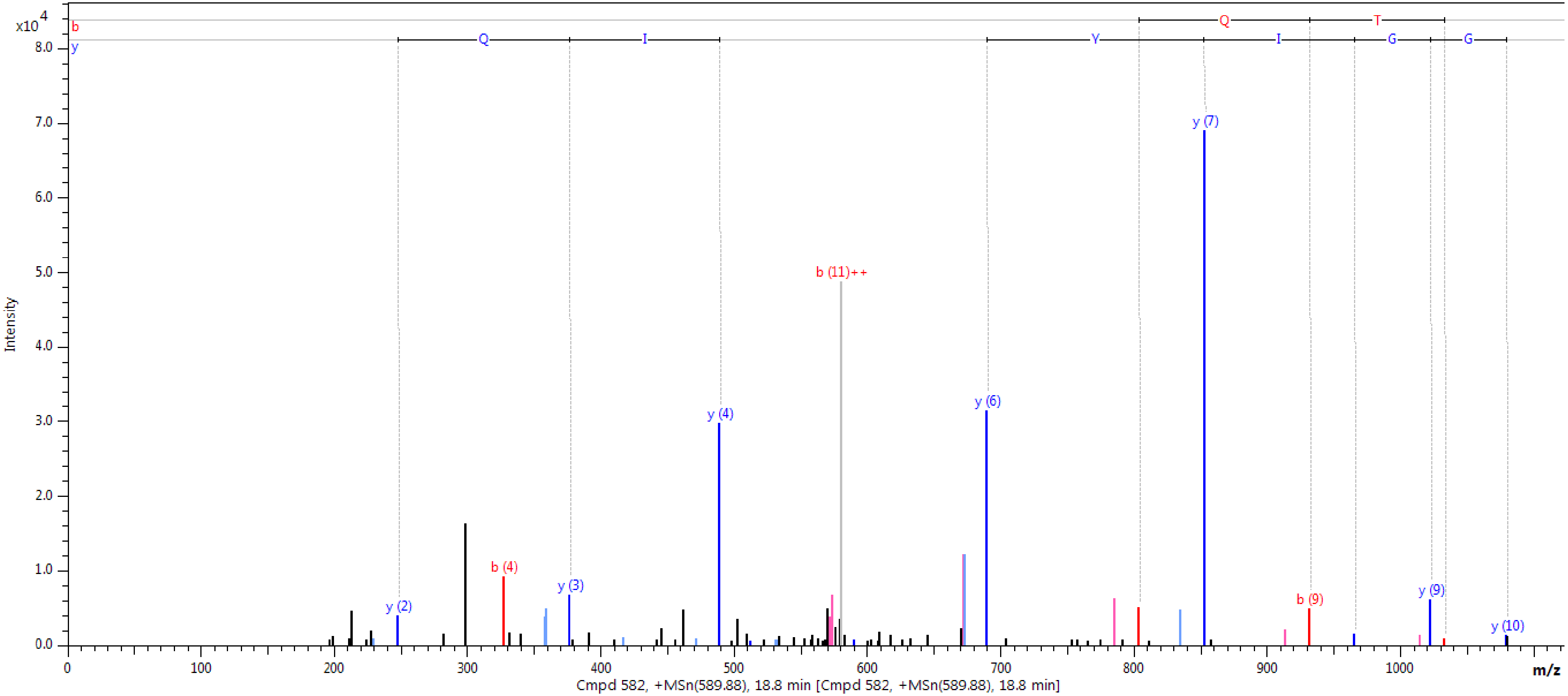

Spectrum Report

Source: M:/Documents/Lamb meat protein project/1. Characterisation of lamb skeletal proteome/Real run - 5 lambs from LCF/mgf_Obj_1/Myo_4-20pc_myo_15B-17B_concat_all_the_line_delet.mgf
Protein: PREDICTED: muscle-related coiled-coil protein [Ovis aries]
Accession: gi|426219785|ref|XP_004004098.1| **Parent m/z:** 658.351, 2+
Sequence: K.VAAIVDSVQASQK.R **Score:** 55.89141818302666

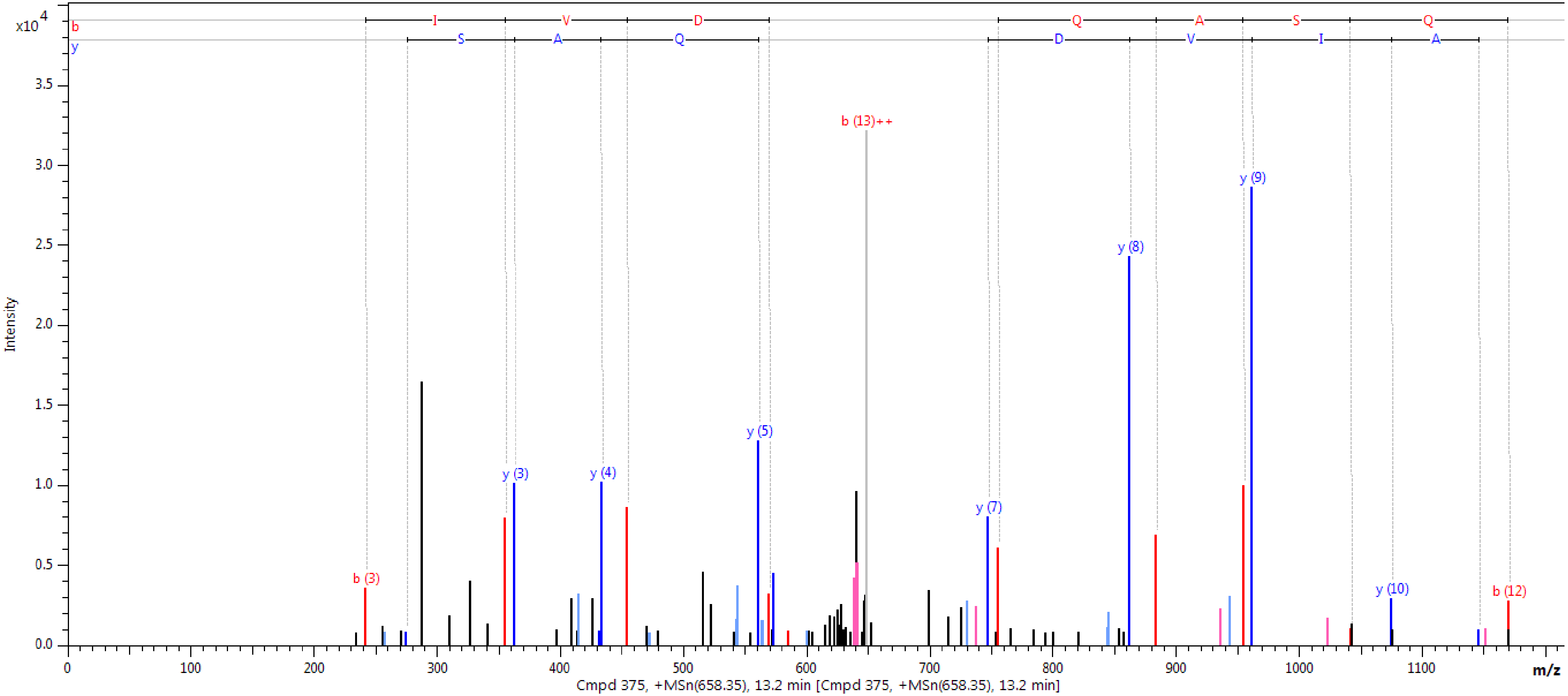

Spectrum Report

Source: M:/Documents/Lamb meat protein project/1. Characterisation of lamb skeletal proteome/Real run - 5 lambs from LCF/mgf_Obj_1/Myo_4-20pc_myo_15B-17B_concat_all_the_line_delet.mgf

Protein: PREDICTED: LOW QUALITY PROTEIN: cullin-associated NEDD8-dissociated protein 2 [Ovis aries]

Accession: gi|426250014|ref|XP_004018737.1|

Parent m/z: 689.479, 2+

Sequence: K.LVLVNPPFLLPR.F

Score: 55.89141818302666

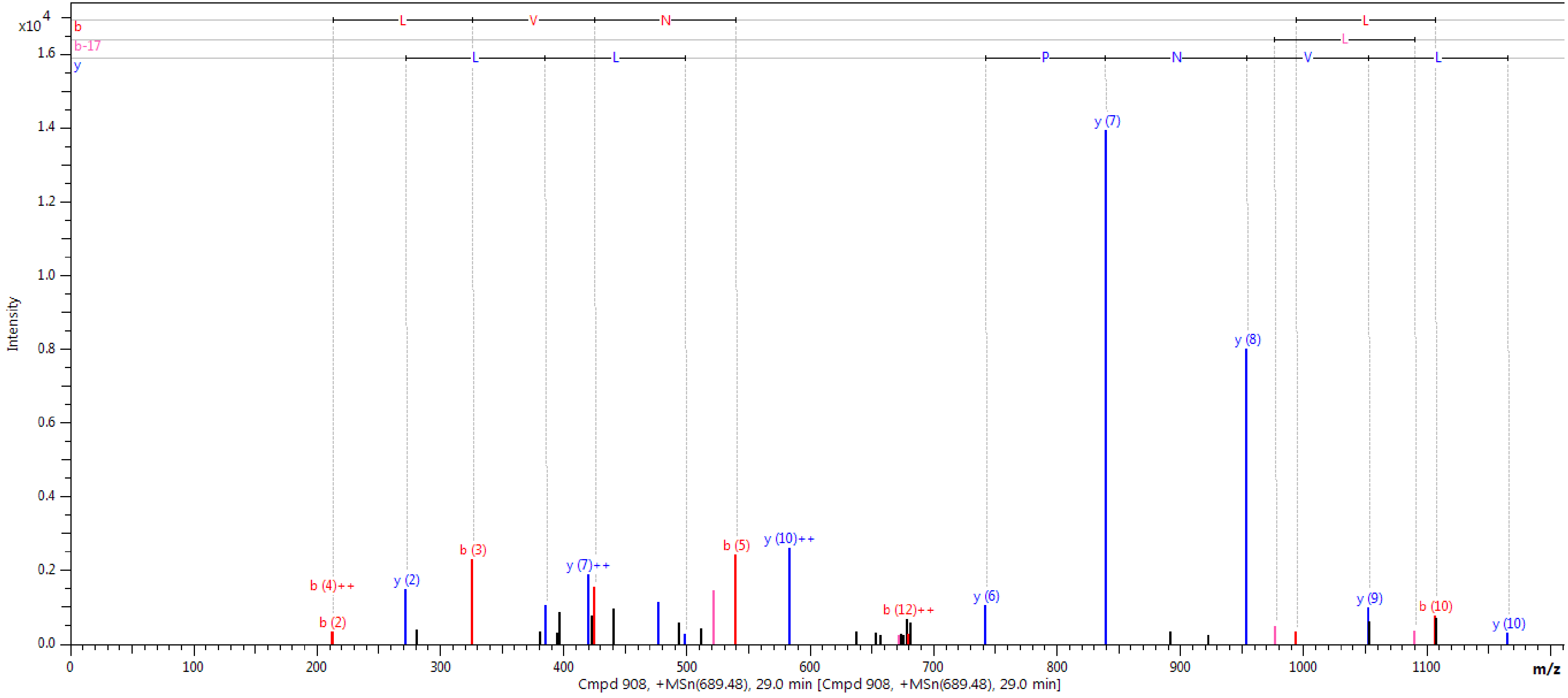

Spectrum Report

Source: M:/Documents/Lamb meat protein project/1. Characterisation of lamb skeletal proteome/Real run - 5 lambs from LCF/mgf_Obj_1/Myo_4-20pc_myo_15B-17B_concat_all_the_line_delet.mgf
Protein: PREDICTED: membrane-associated progesterone receptor component 2-like, partial [Ovis aries]
Accession: gi|426247626|ref|XP_004017580.1|
Parent m/z: 736.907, 2+
Sequence: K.FYGPAGPYGIFAGR.D
Score: 55.89141818302666

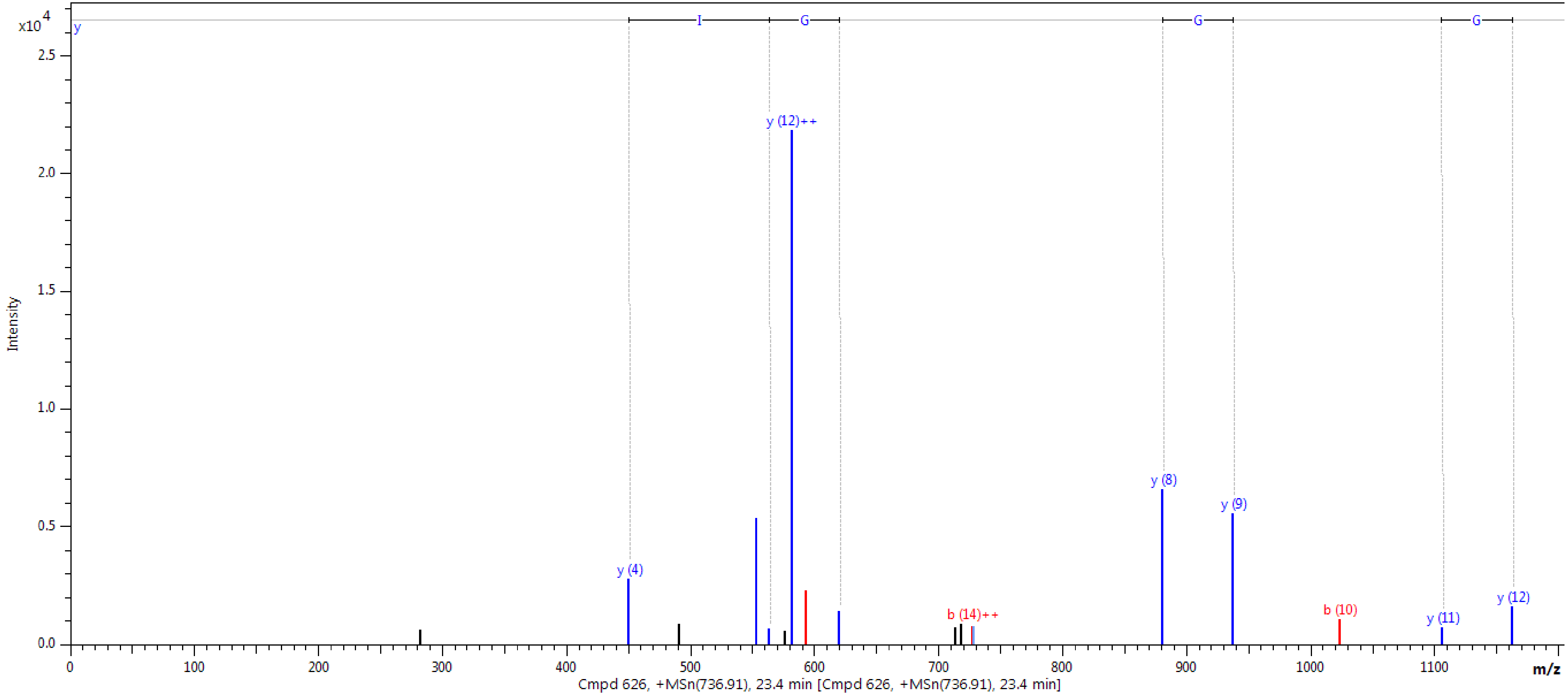

Spectrum Report

Source: M:/Documents/Lamb meat protein project/1. Characterisation of lamb skeletal proteome/Real run - 5 lambs from LCF/mgf_Obj_1/Myo_4-20pc_myo_15B-17B_concat_all_the_line_delet.mgf
Protein: PREDICTED: prenylcysteine oxidase-like [Ovis aries]
Accession: gi|426223380|ref|XP_004005853.1|
Parent m/z: 753.446, 2+
Sequence: R.SNLVSGLVMSIEEK.T
Score: 55.89141818302666

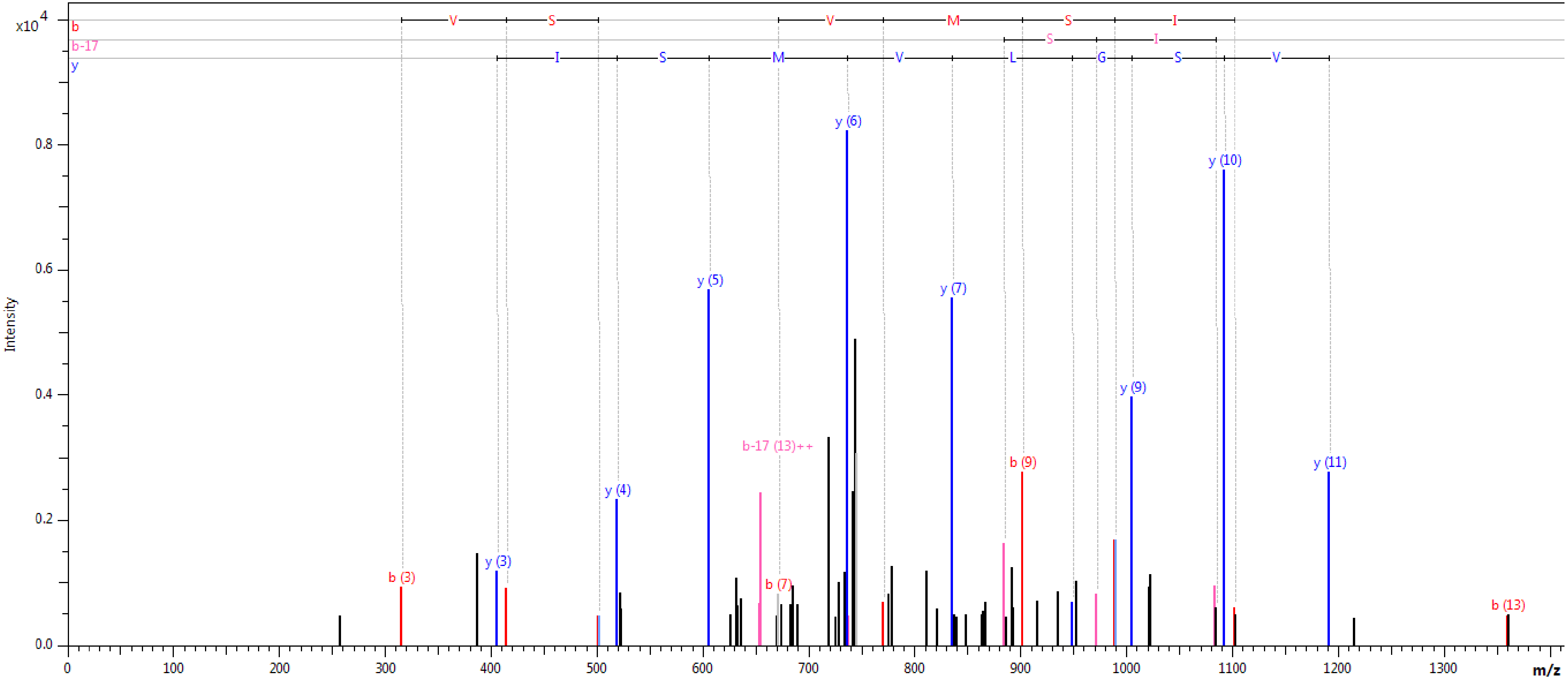

Cmpd 950, +MSn(753.45), 26.8 min [Cmpd 950, +MSn(753.45), 26.8 min]

Spectrum Report

Source: M:/Documents/Lamb meat protein project/1. Characterisation of lamb skeletal proteome/Real run - 5 lambs from LCF/mgf_Obj_1/Myo_4-20pc_myo_15B-17B_concat_all_the_line_delet.mgf
Protein: PREDICTED: smoothelin-like protein 2-like [Ovis aries]
Accession: gi|426238773|ref|XP_004013322.1|
Sequence: R.SQSFGVASASSIK.Q
Parent m/z: 634.803, 2+
Score: 55.89141818302666

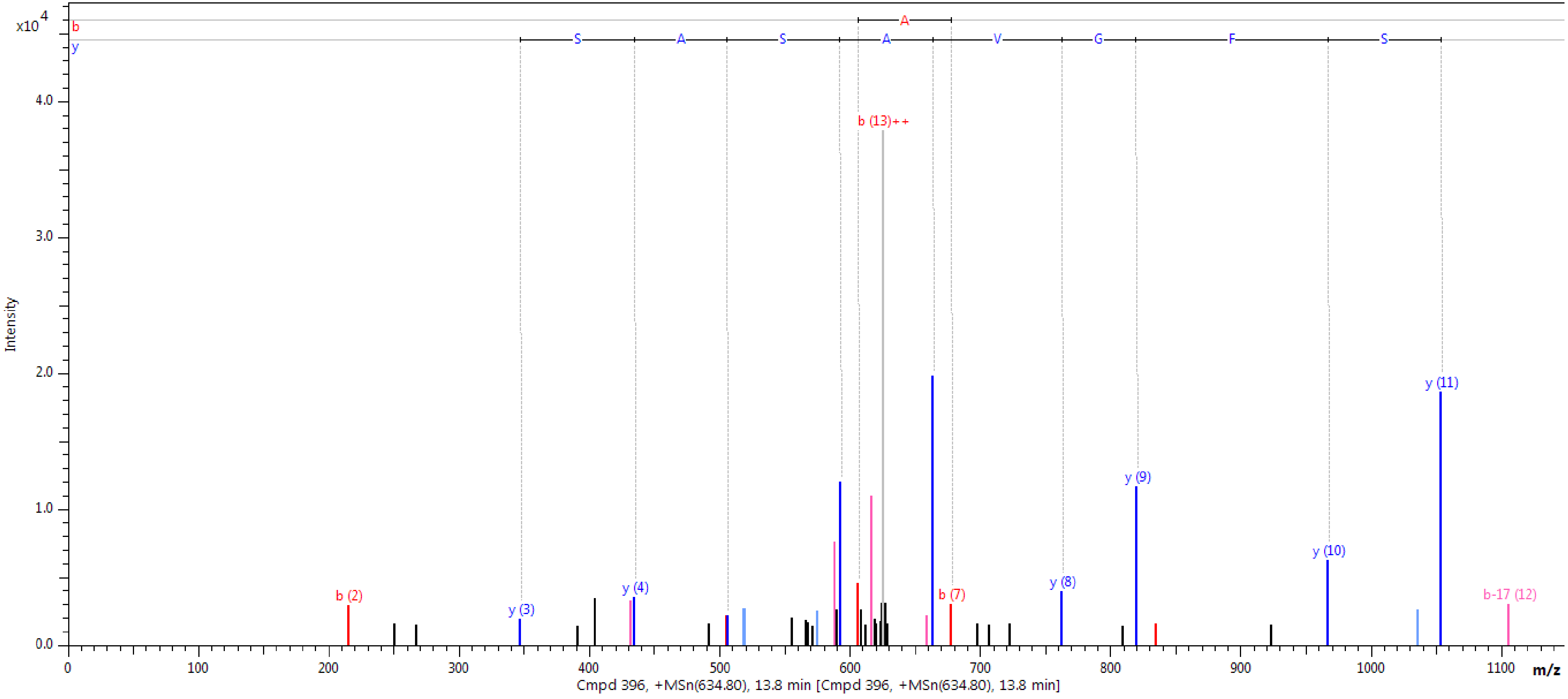

Spectrum Report

Source: M:/Documents/Lamb meat protein project/1. Characterisation of lamb skeletal proteome/Real run - 5 lambs from LCF/ mgf_Obj_1/Myo_4-20pc_myo_15B-17B_concat_all_the_line_delet.mgf
Protein: PREDICTED: NADH dehydrogenase [ubiquinone] 1 subunit C2 [Ovis aries]
Accession: gi|426251539|ref|XP_004019479.1|
Parent m/z: 628.883, 2+
Sequence: R.APLQFLPDEAR.S
Score: 55.89141818302666

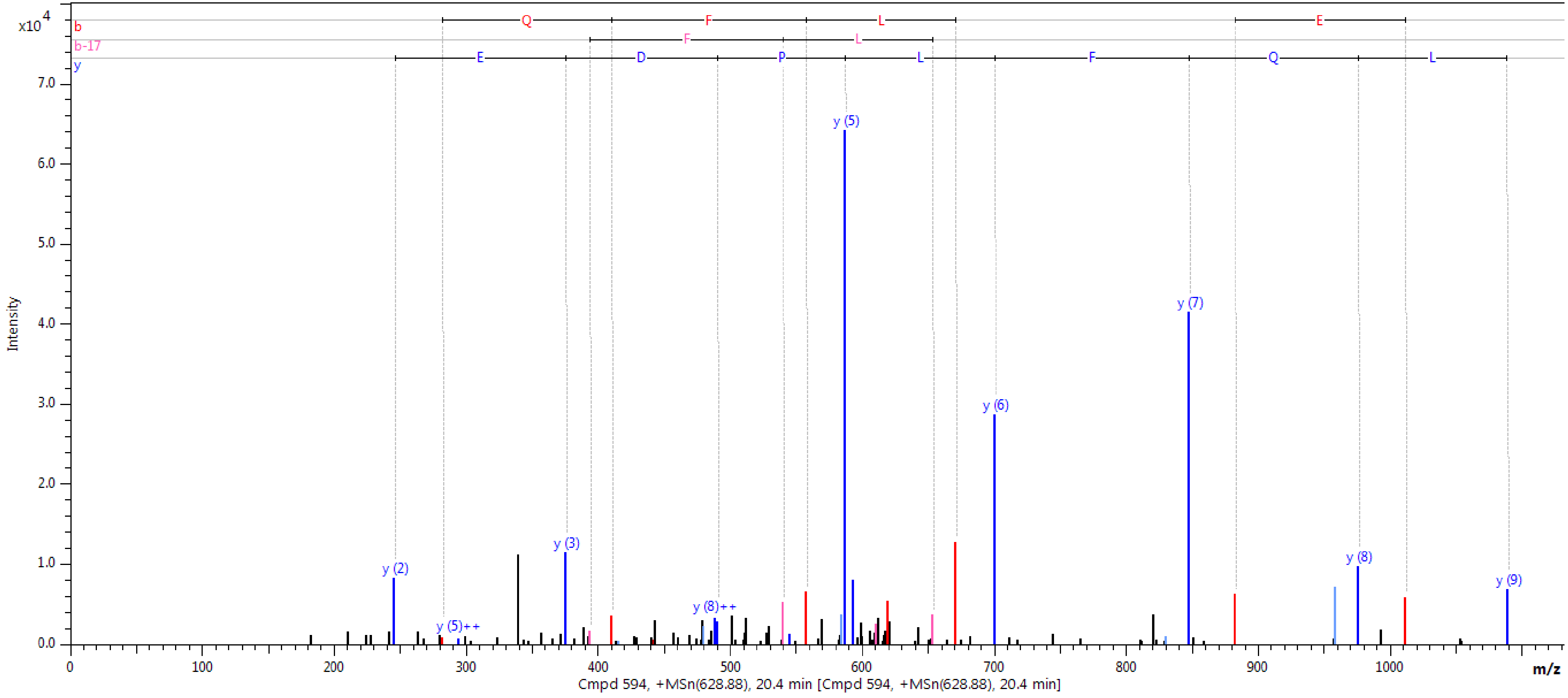

Spectrum Report

Source: M:/Documents/Lamb meat protein project/1. Characterisation of lamb skeletal proteome/Real run - 5 lambs from LCF/mgf_Obj_1/Myo_4-20pc_myo_15B-17B_concat_all_the_line_delet.mgf
Protein: PREDICTED: transmembrane protein 109 [Ovis aries]
Accession: gi|426252368|ref|XP_004019886.1|
Parent m/z: 770.927, 2+
Sequence: R.EAPVDLLSQIGGSVR.G
Score: 55.89141818302666

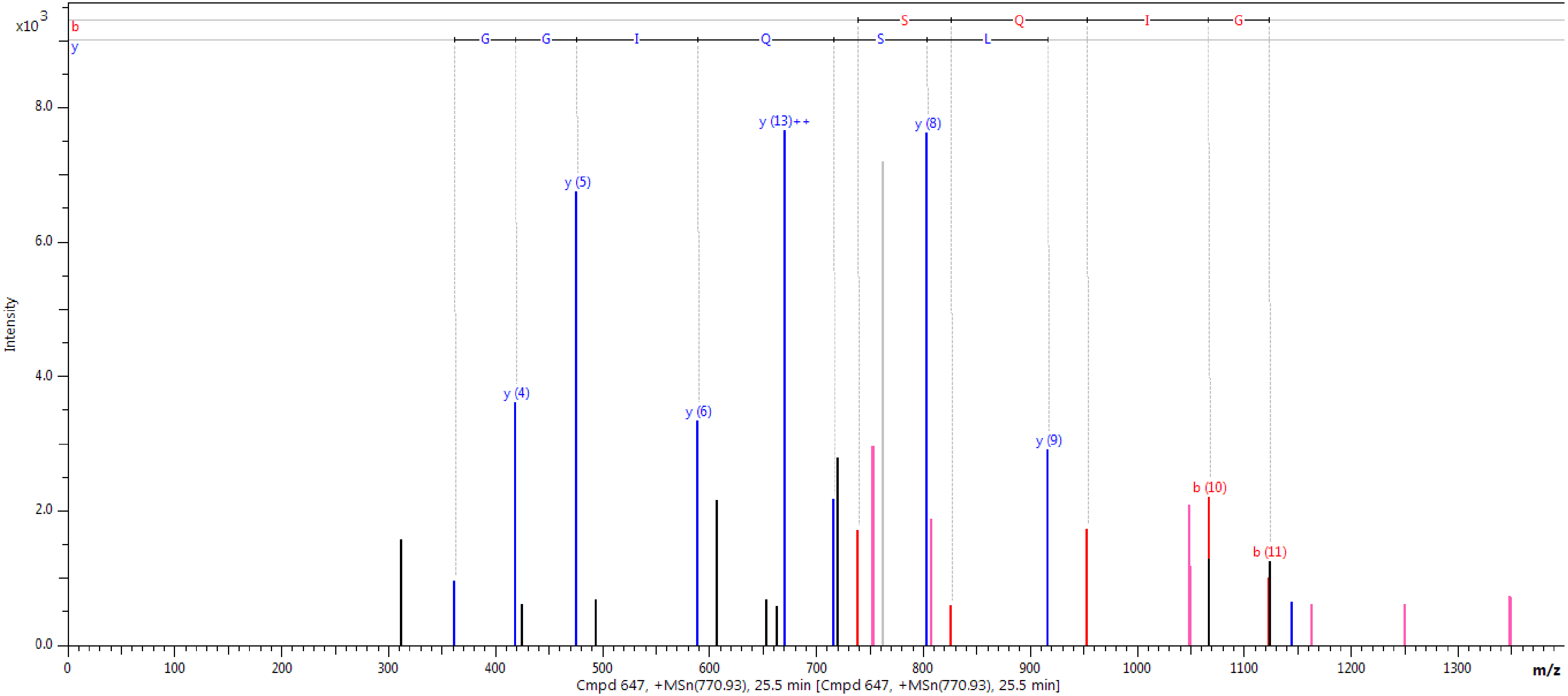

Spectrum Report

Source: M:/Documents/Lamb meat protein project/1. Characterisation of lamb skeletal proteome/Real run - 5 lambs from LCF/ mgf_Obj_1/Myo_4-20pc_myo_15B-17B_concat_all_the_line_delet.mgf
Protein: PREDICTED: NADH dehydrogenase [ubiquinone] 1 beta subcomplex subunit 5, mitochondrial isoform 2 [Ovis aries]
Accession: gi|426217878|ref|XP_004003179.1|
Parent m/z: 631.901, 2+
Sequence: R.TMAILQIESEK.A
Score: 55.89141818302666

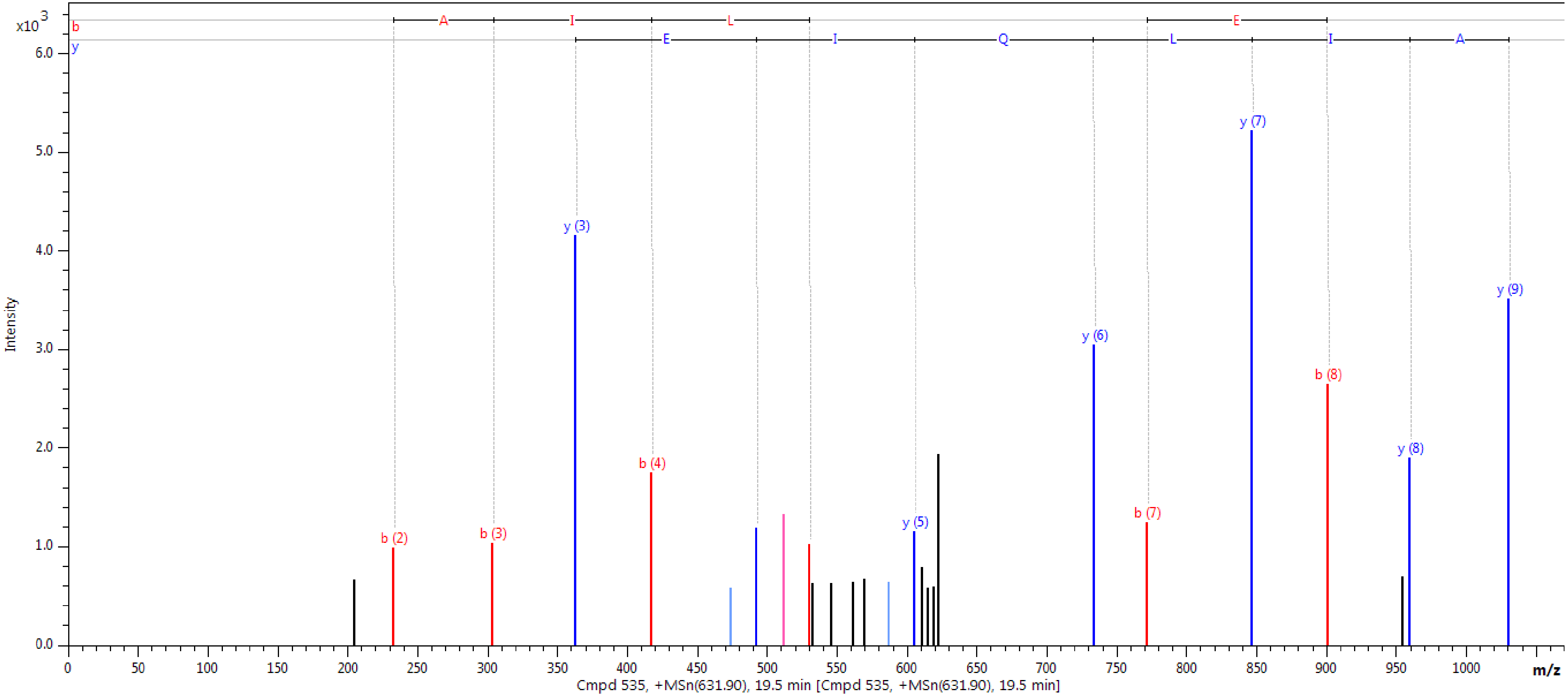

Spectrum Report

Source: M:/Documents/Lamb meat protein project/1. Characterisation of lamb skeletal proteome/Real run - 5 lambs from LCF/mgf_Obj_1/Myo_4-20pc_myo_15B-17B_concat_all_the_line_delet.mgf
Protein: PREDICTED: acyl-coenzyme A thioesterase 13 [Ovis aries]
Accession: gi|426250860|ref|XP_004019151.1|
Parent m/z: 641.387, 2+
Sequence: R.SISFASVDLTNK.A
Score: 56.4860378373239

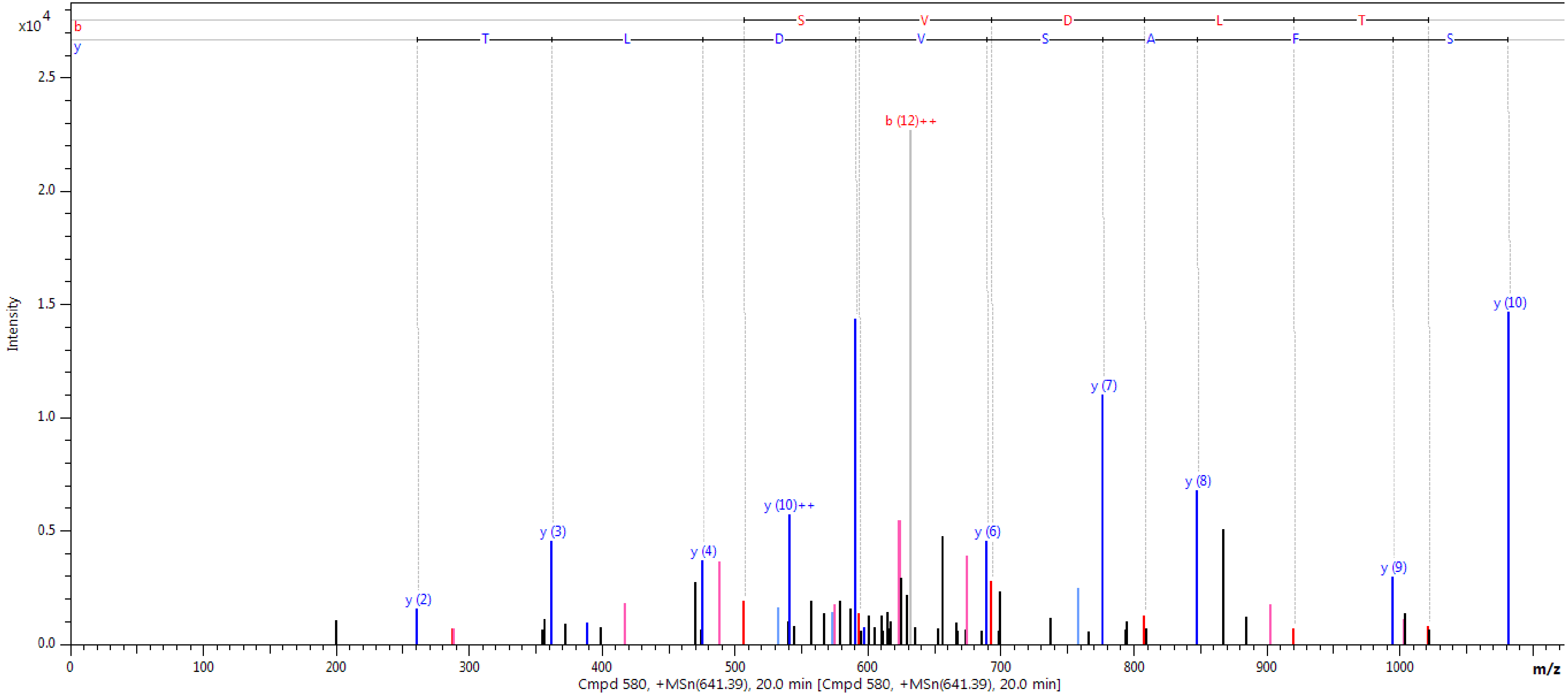

Spectrum Report

Source: M:/Documents/Lamb meat protein project/1. Characterisation of lamb skeletal proteome/Real run - 5 lambs from LCF/ mgf_Obj_1/Myo_4-20pc_myo_15B-17B_concat_all_the_line_delet.mgf
Protein: PREDICTED: uncharacterized protein LOC101118195 [Ovis aries]
Accession: gi|426235556|ref|XP_004011746.1|
Parent m/z: 527.373, 2+
Sequence: K.VLLEGPGGLAK.R
Score: 55.89141818302666

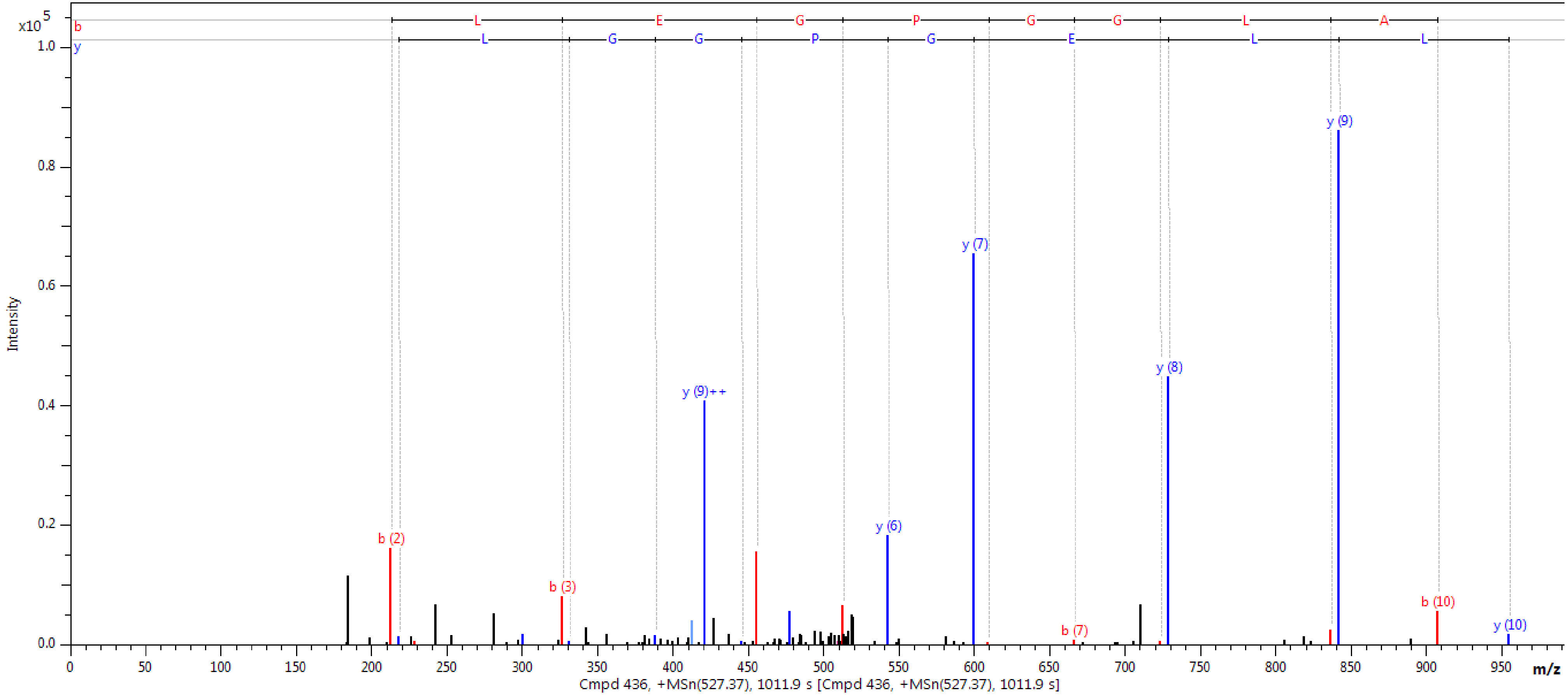

agresearch

Spectrum Report

Source: M:/Documents/Lamb meat protein project/1. Characterisation of lamb skeletal proteome/Real run - 5 lambs from LCF/mgf_Obj_1/Myo_4-20pc_myo_15B-17B_concat_all_the_line_delet.mgf
Protein: similar to Serine/threonine-protein kinase SIK3, partial [Ovis aries: Oar v3]
Accession: gi|1999014709|gb|1999014709.1|
Parent m/z: 699.917, 2+
Sequence: R.DYVAQFEASALGK.Q
Score: 55.89141818302666

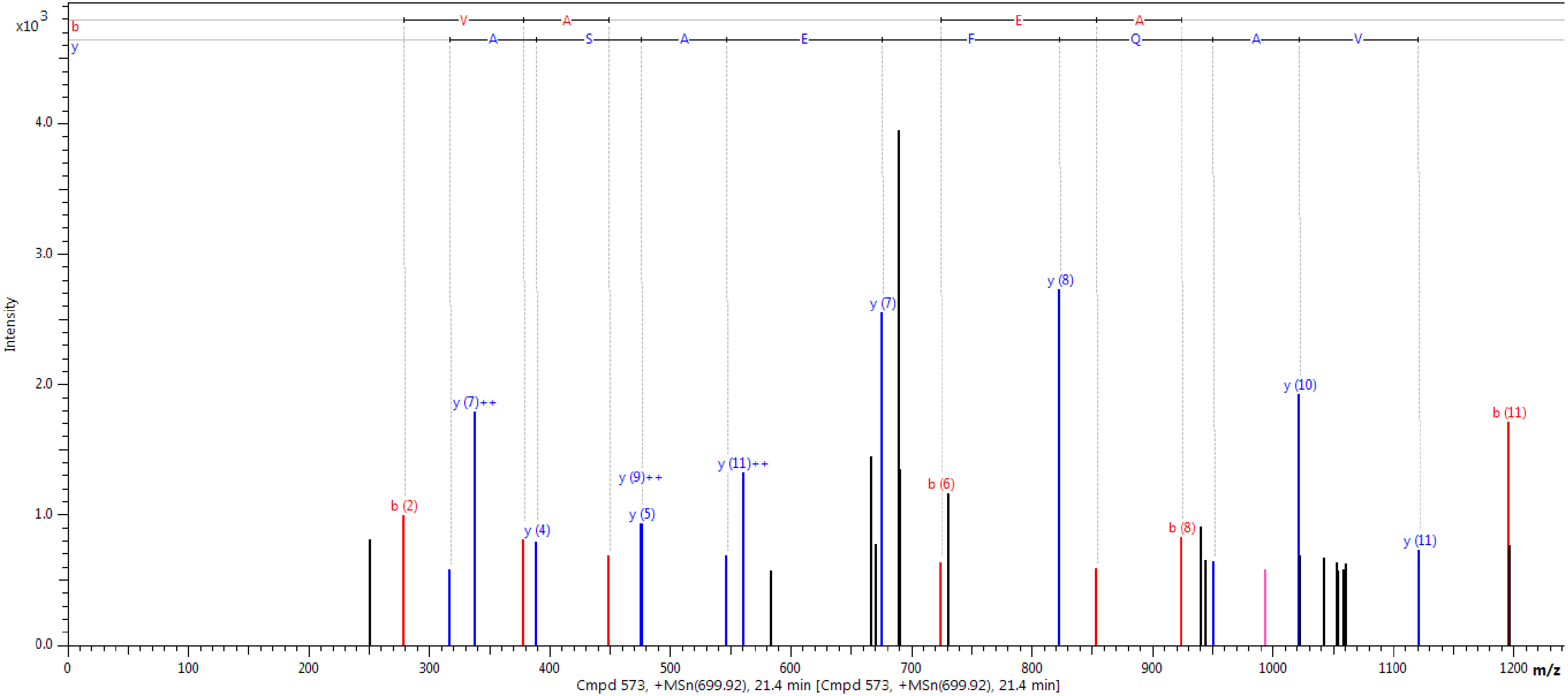

agresearch

Spectrum Report

Source: M:/Documents/Lamb meat protein project/1. Characterisation of lamb skeletal proteome/Real run - 5 lambs from LCF/mgf_Obj_1/Myo_4-20pc_myo_15B-17B_concat_all_the_line_delet.mgf
Protein: ribosomal protein S15a [Ovis aries]
Accession: gi|78558752|gb|ABB46363.1|
Sequence: K.IVVNLTGR.L
Parent m/z: 436.295, 2+
Score: 20.591026783359005

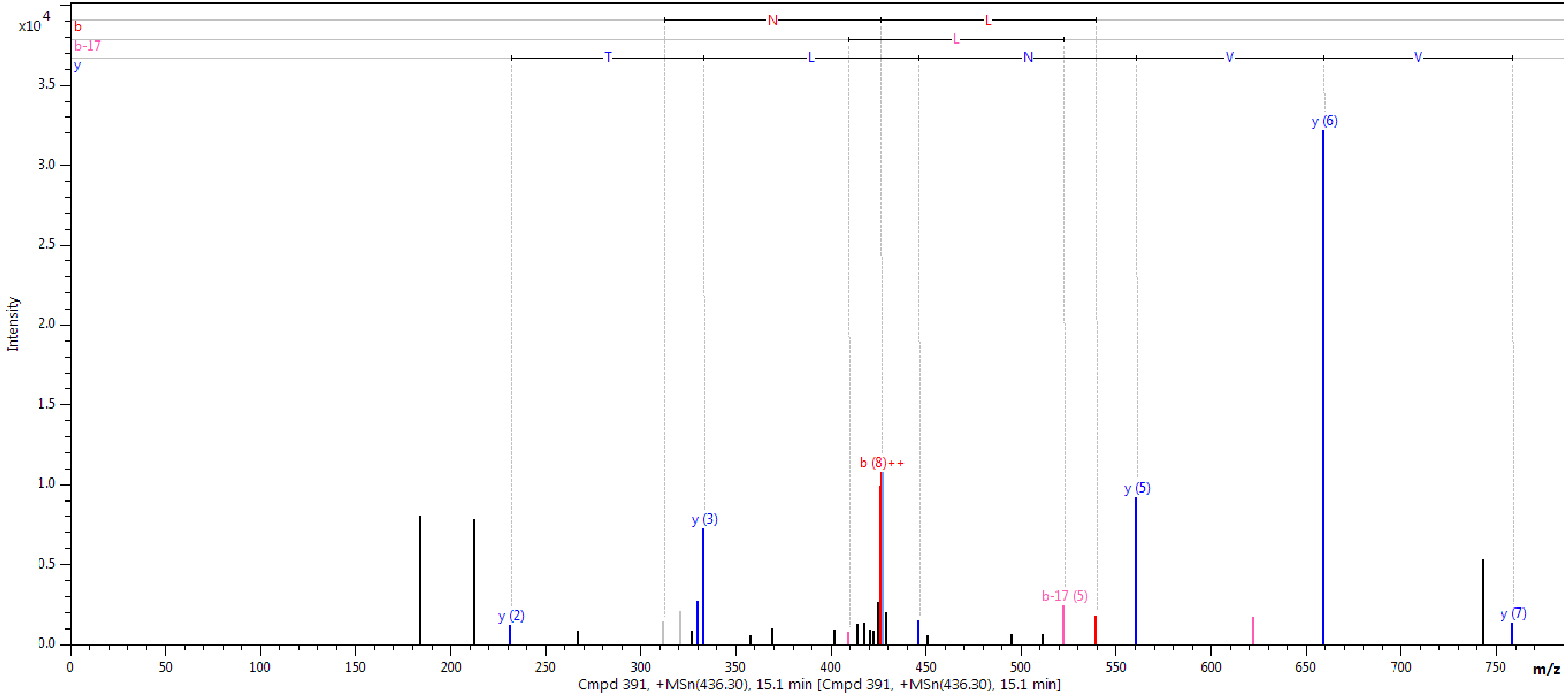

Spectrum Report

Source: M:/Documents/Lamb meat protein project/1. Characterisation of lamb skeletal proteome/Real run - 5 lambs from LCF/mgf_Obj_1/Myo_4-20pc_myo_15B-17B_concat_all_the_line_delet.mgf
Protein: Histone 1 family member partial (tentative) cs39 [Ovis aries]
Accession: gi|999000104|gb|99900000.104|
Parent m/z: 554.328, 2+
Sequence: K.ALAAAGYDVEK.N
Score: 30.354726138521798

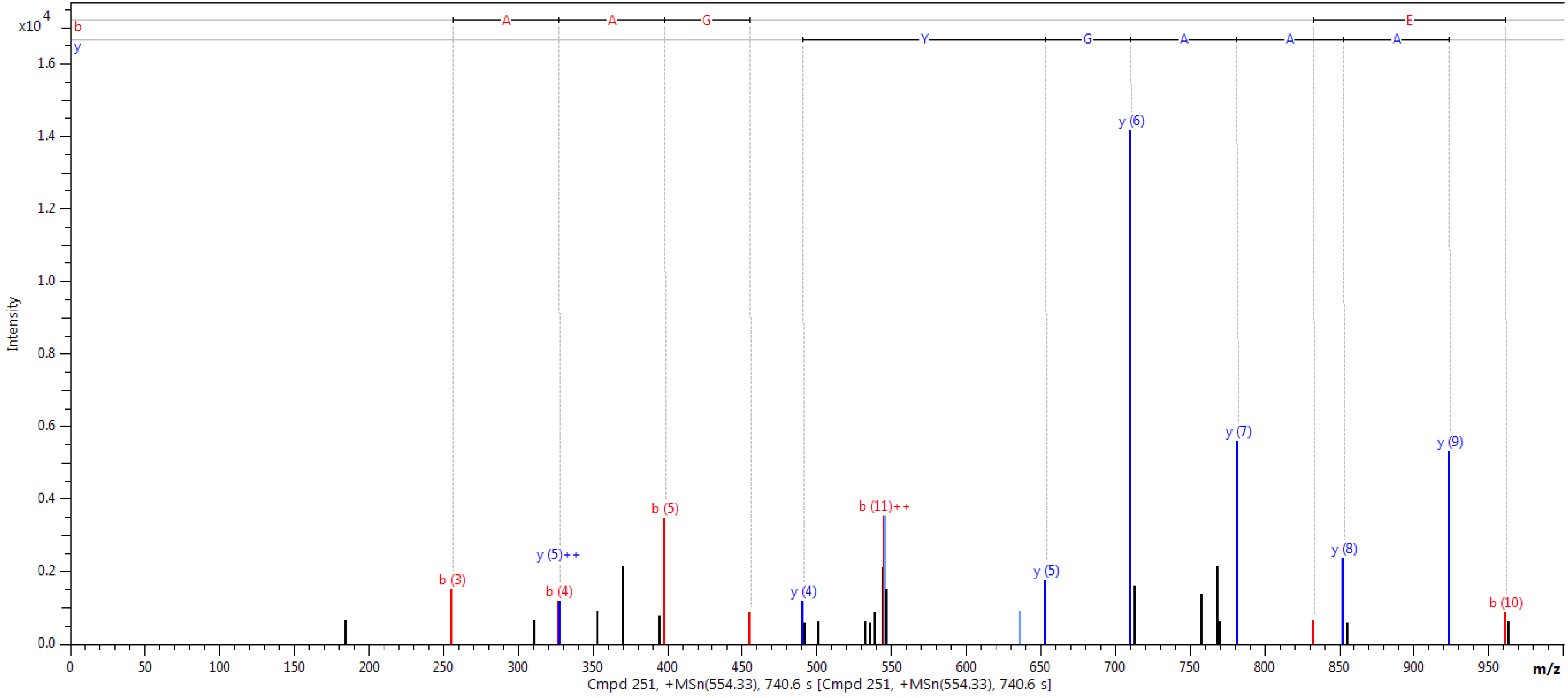

Spectrum Report

Source: M:/Documents/Lamb meat protein project/1. Characterisation of lamb skeletal proteome/Real run - 5 lambs from LCF/mgf_Obj_1/Myo_4-20pc_myo_15B-17B_concat_all_the_line_delet.mgf
Protein: RecName: Full=Troponin T, cardiac muscle; Short=TnTc; AltName: Full=Cardiac muscle troponin T; Short=cTnT
Accession: gi|1717775|sp|P50751.2|TNNT2_SHEEP **Parent m/z:** 508.791, 2+
Sequence: R.VDFDDIHR.K **Score:** 42.460723996515746

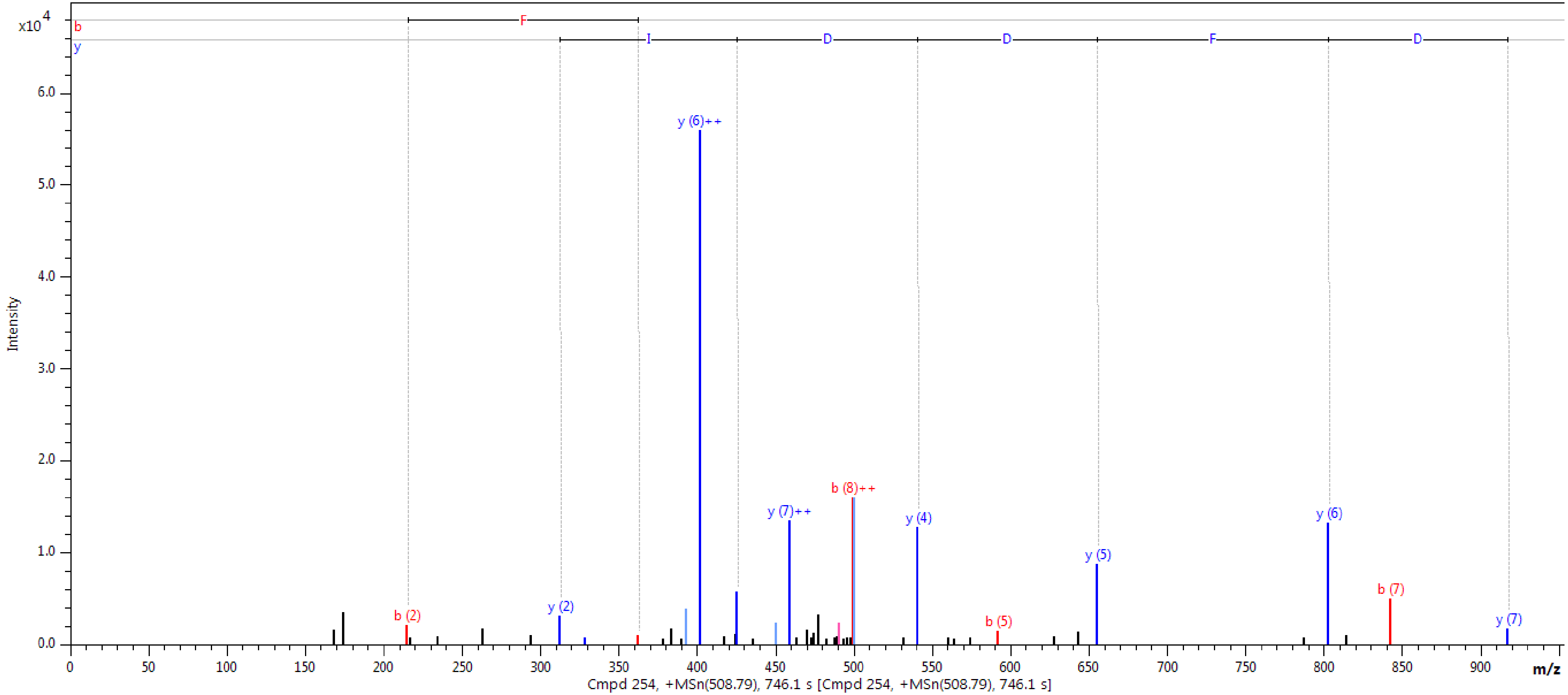

Spectrum Report

Source: M:/Documents/Lamb meat protein project/1. Characterisation of lamb skeletal proteome/Real run - 5 lambs from LCF/mgf_Obj_1/Myo_4-20pc_myo_15B-17B_concat_all_the_line_delet.mgf
Protein: PREDICTED: 40S ribosomal protein S16 [Ovis aries]
Accession: gi|426243798|ref|XP_004015735.1|
Parent m/z: 547.94, 2+
Sequence: K.LLEPVLLLGK.E
Score: 27.144111889081085

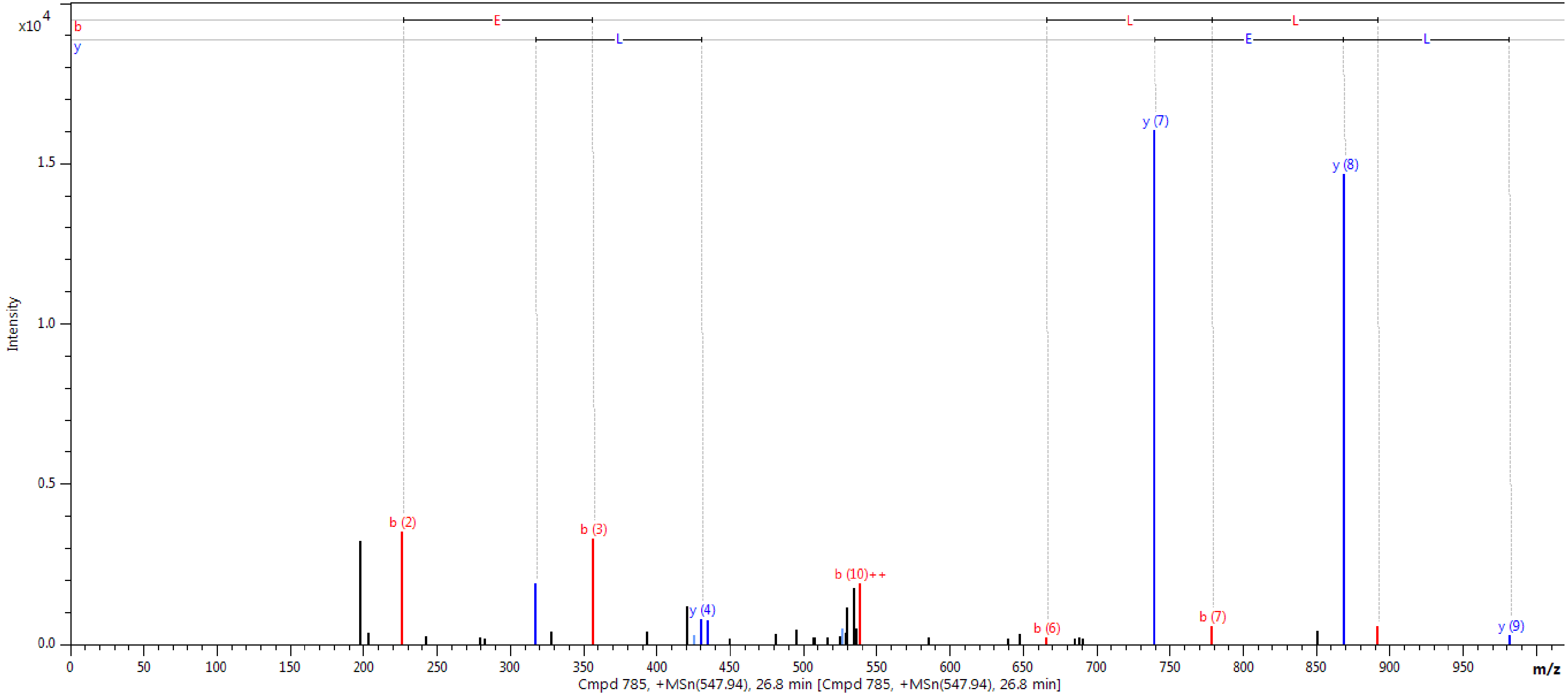

Spectrum Report

Source: M:/Documents/Lamb meat protein project/1. Characterisation of lamb skeletal proteome/Real run - 5 lambs from LCF/mgf_Obj_1/Myo_4-20pc_myo_15B-17B_concat_all_the_line_delet.mgf
Protein: PREDICTED: SH3 domain-binding glutamic acid-rich-like protein 3 [Ovis aries]
Accession: gi|426221901|ref|XP_004005144.1|
Parent m/z: 528.807, 2+
Sequence: R.VYSTSVTGSR.E
Score: 55.8914013203976

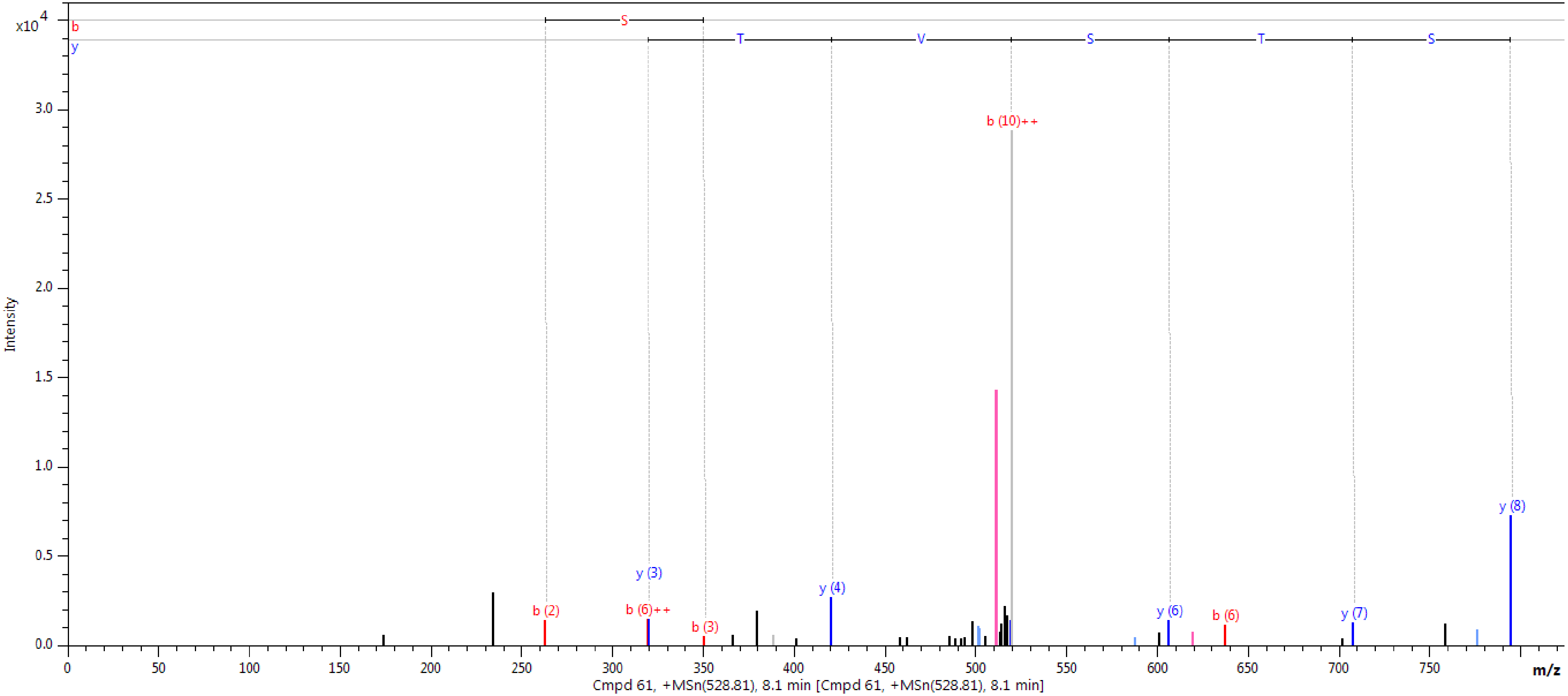

agresearch

Spectrum Report

Source: M:/Documents/Lamb meat protein project/1. Characterisation of lamb skeletal proteome/Real run - 5 lambs from LCF/mgf_Obj_1/Myo_4-20pc_myo_15B-17B_concat_all_the_line_delet.mgf

Protein: uterine myometrial annexin 2 [Ovis aries]

Accession: gi|86279630|gb|ABC94470.1|

Sequence: K.TPAQYDASELK.A

Parent m/z: 611.825, 2+

Score: 42.731106737647224

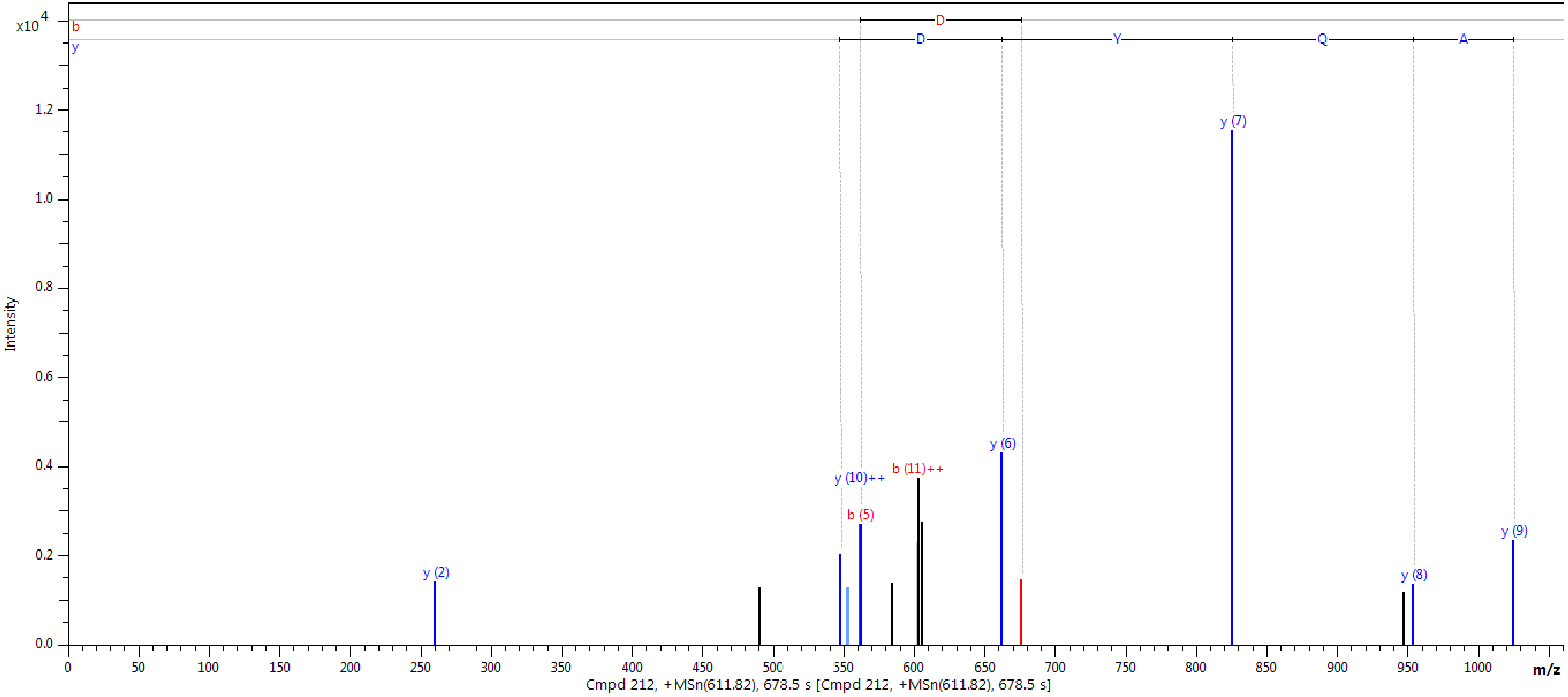

Spectrum Report

Source: M:/Documents/Lamb meat protein project/1. Characterisation of lamb skeletal proteome/Real run - 5 lambs from LCF/mgf_Obj_1/Myo_4-20pc_myo_15B-17B_concat_all_the_line_delet.mgf
Protein: gelsolin isoform b, partial [Ovis aries]
Accession: gi|414148023|gb|AFW98884.1|
Sequence: K.AGALNSNDAFVLK.T
Parent m/z: 660.408, 2+
Score: 43.026712518084686

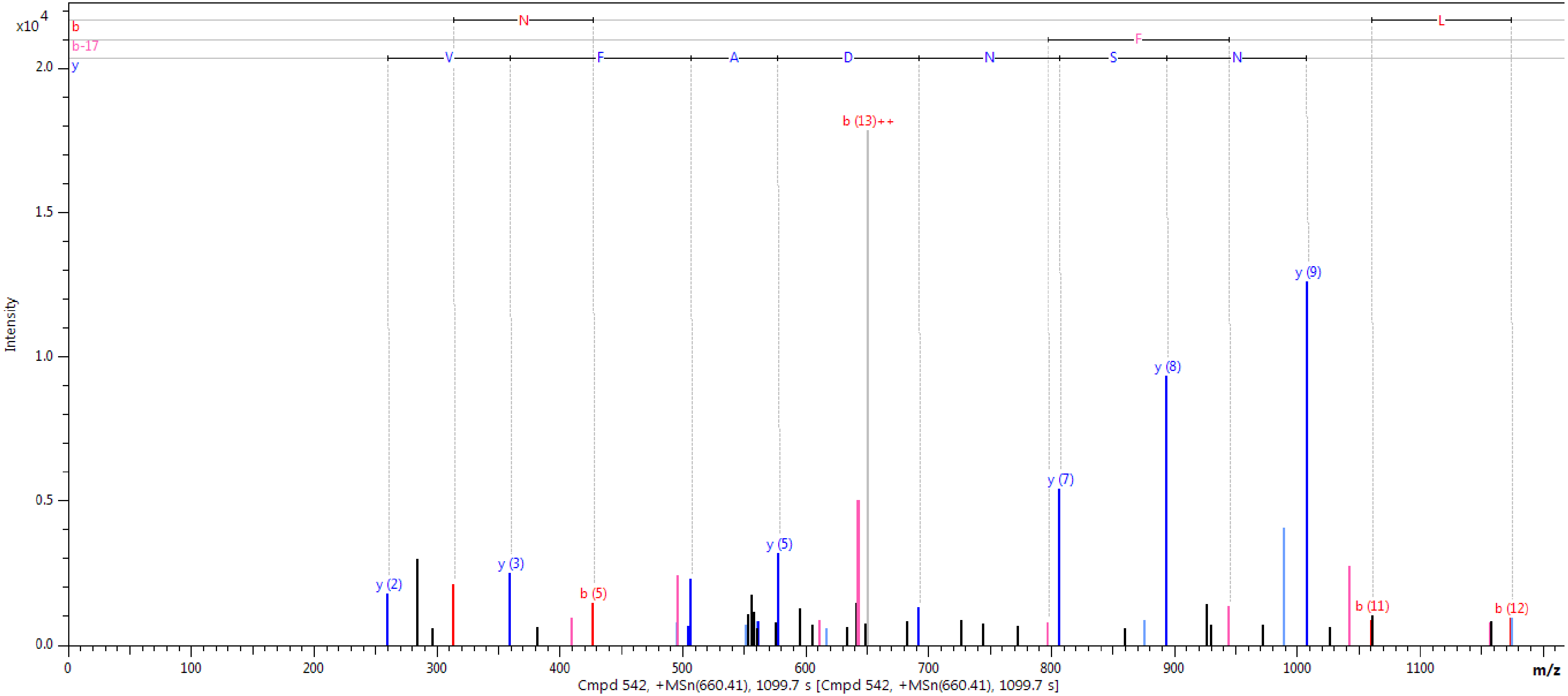

Spectrum Report

Source: M:/Documents/Lamb meat protein project/1. Characterisation of lamb skeletal proteome/Real run - 5 lambs from LCF/mgf_Obj_1/Myo_4-20pc_myo_15B-17B_concat_all_the_line_delet.mgf
Protein: protein kinase C inhibitor KCIP-1 isoform e - sheep (fragment)
Accession: gi|109126|pir||S10808 **Parent m/z:** 547.864, 2+
Sequence: R.YLAEVAAQDD.- **Score:** 27.059682856124248

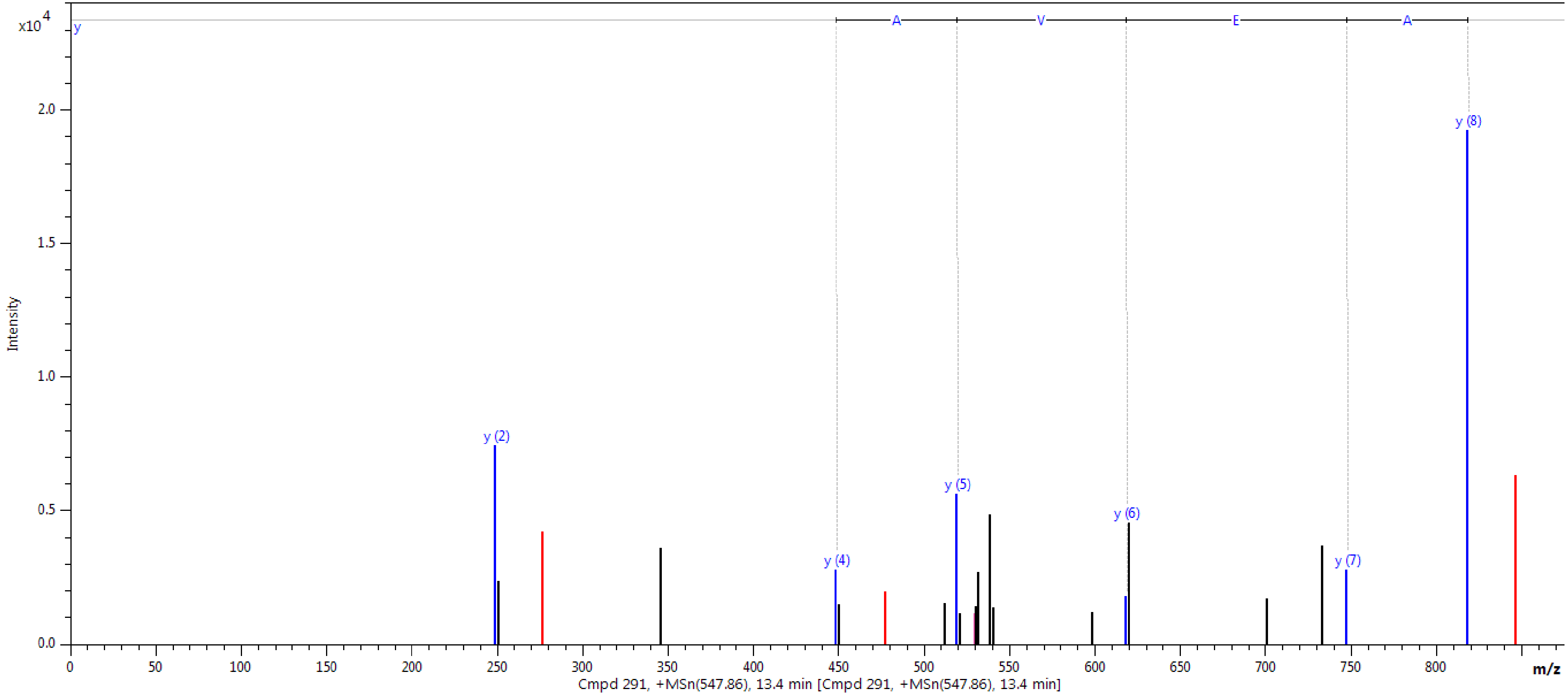

Spectrum Report

Source: M:/Documents/Lamb meat protein project/1. Characterisation of lamb skeletal proteome/Real run - 5 lambs from LCF/ mgf_Obj_1/Myo_4-20pc_myo_15B-17B_concat_all_the_line_delet.mgf
Protein: PREDICTED: NADH dehydrogenase [ubiquinone] 1 beta subcomplex subunit 1 [Ovis aries]
Accession: gi|426248502|ref|XP_004018002.1|
Parent m/z: 486.794, 2+
Sequence: M.MNLLQVVR.D
Score: 53.19932036355383

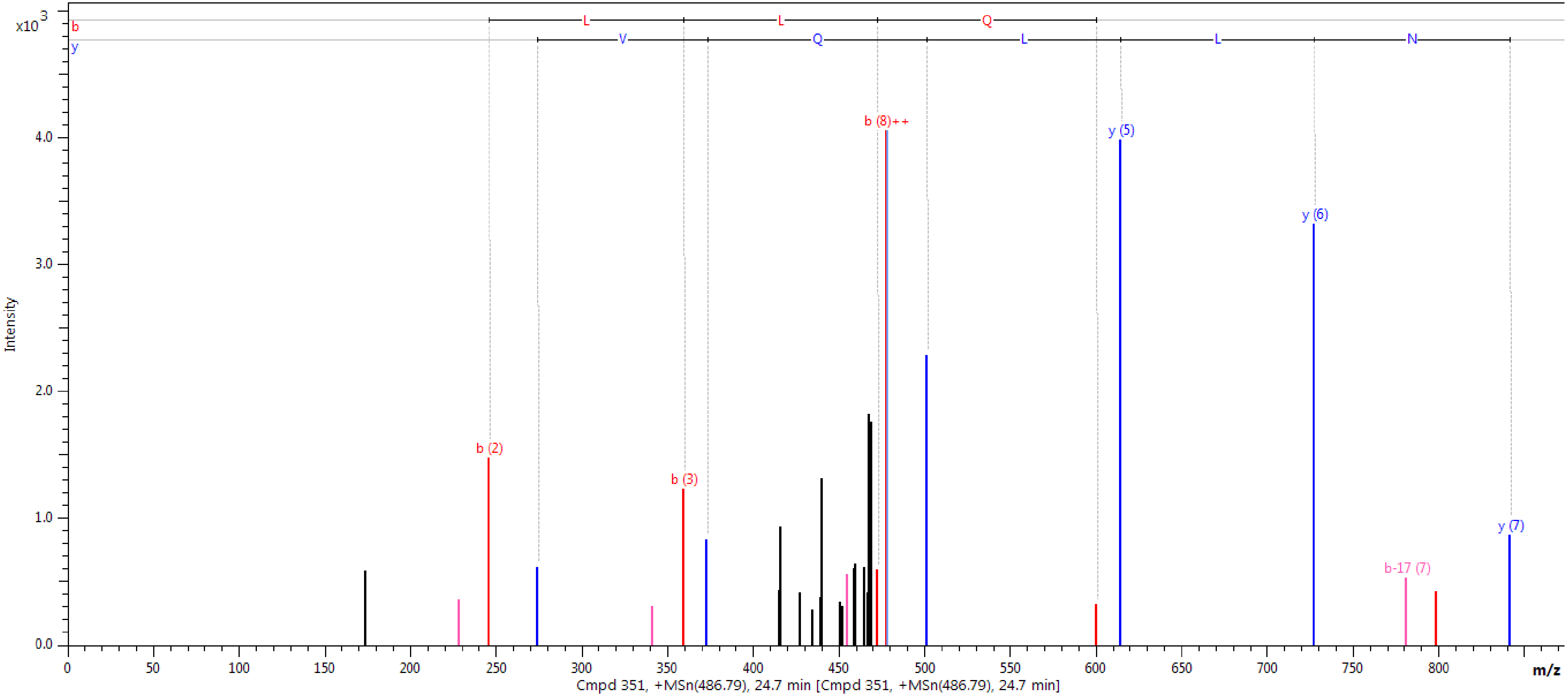

agresearch

Spectrum Report

Source: M:/Documents/Lamb meat protein project/1. Characterisation of lamb skeletal proteome/Real run - 5 lambs from LCF/ mgf_Obj_1/Myo_4-20pc_myo_15B-17B_concat_all_the_line_delet.mgf
Protein: mitochondrial ATP synthase O subunit [Ovis aries]
Accession: gi|222092853|gb|ACM43308.1|
Parent m/z: 533.05, 3+
Sequence: K.LVRPPVQIYGIEGR.Y
Score: 46.18280397803332

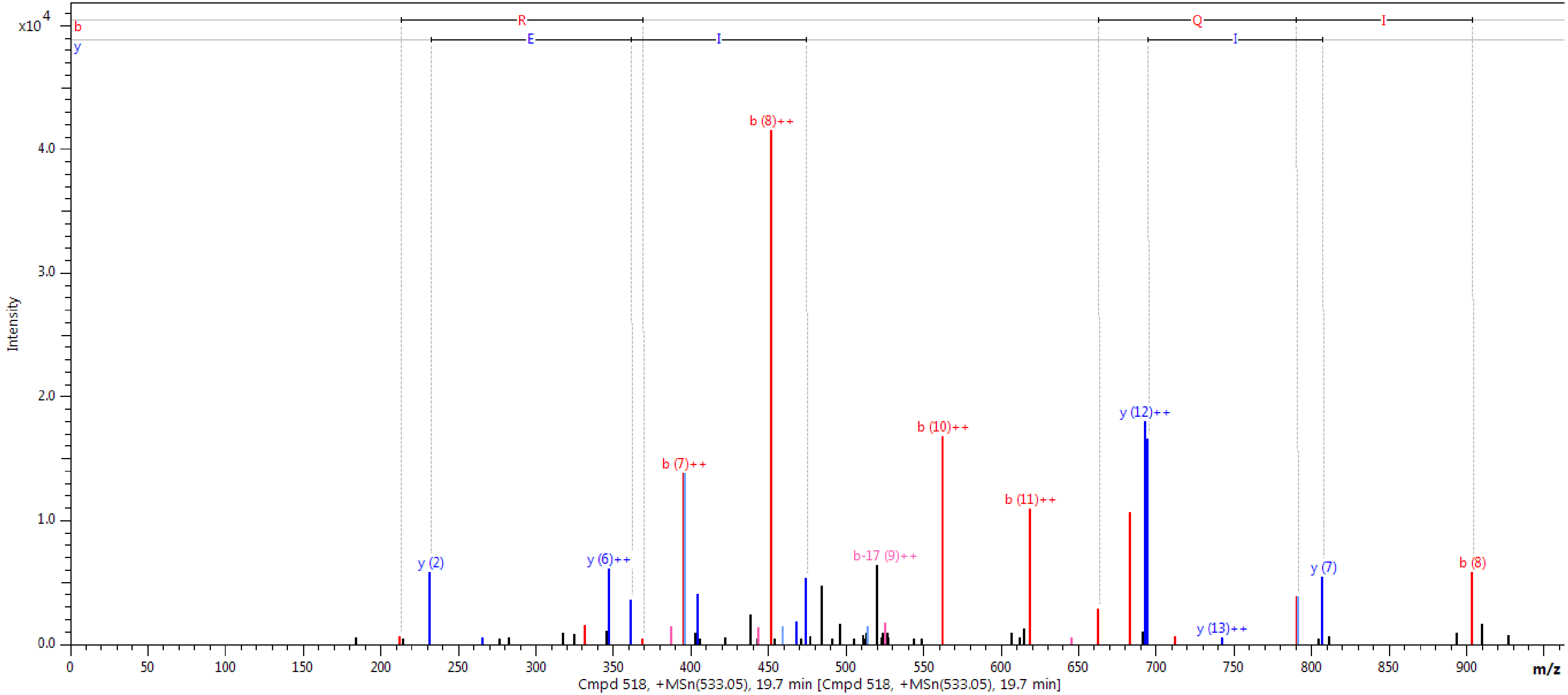

Spectrum Report

Source: M:/Documents/Lamb meat protein project/1. Characterisation of lamb skeletal proteome/Real run - 5 lambs from LCF/mgf_Obj_1/Myo_4-20pc_myo_15B-17B_concat_all_the_line_delet.mgf
Protein: PREDICTED: synaptophysin-like protein 1 [Ovis aries]
Accession: gi|426228364|ref|XP_004008281.1|
Sequence: K.TITAAFGYPFR.L
Parent m/z: 622.381, 2+
Score: 26.066301481178286

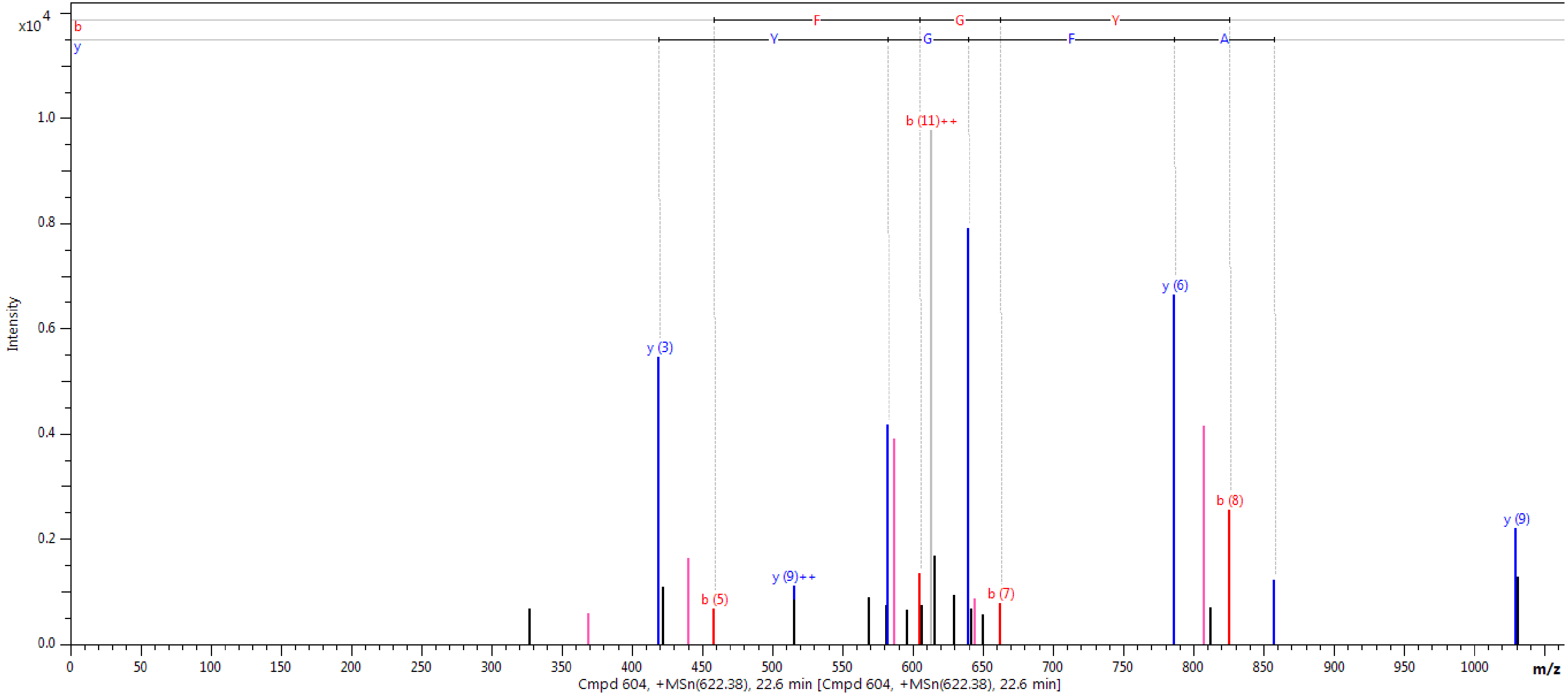

Spectrum Report

Source: M:/Documents/Lamb meat protein project/1. Characterisation of lamb skeletal proteome/Real run - 5 lambs from LCF/mgf_Obj_1/Myo_4-20pc_myo_15B-17B_concat_all_the_line_delet.mgf
Protein: PREDICTED: LOW QUALITY PROTEIN: ryanodine receptor 1 [Ovis aries]
Accession: gi|426243766|ref|XP_004015719.1| **Parent m/z:** 600.906, 2+
Sequence: R.LAVFAQPIVSR.A **Score:** 55.89141818302666

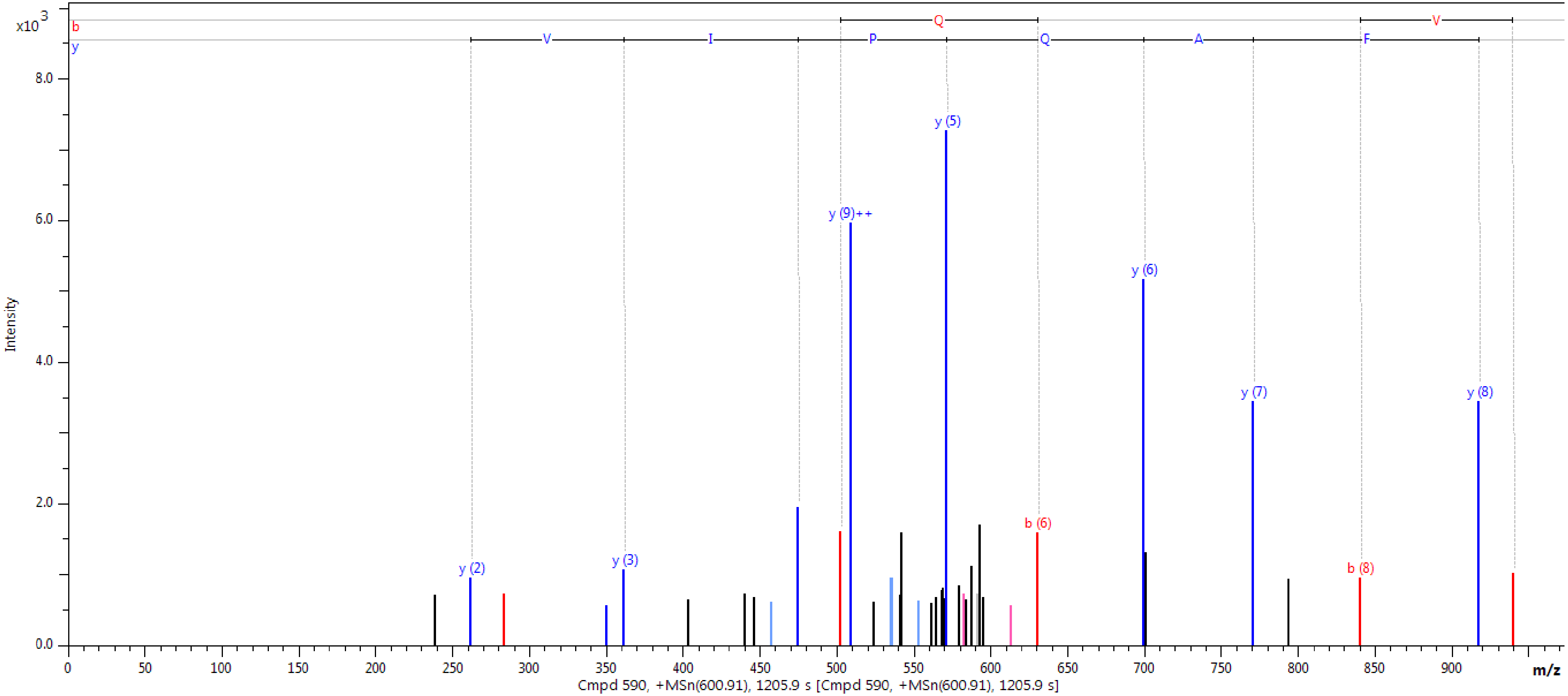

Spectrum Report

Source: M:/Documents/Lamb meat protein project/1. Characterisation of lamb skeletal proteome/Real run - 5 lambs from LCF/ mgf_Obj_1/Myo_4-20pc_myo_15B-17B_concat_all_the_line_delet.mgf
Protein: PREDICTED: isocitrate dehydrogenase [NADP], mitochondrial, partial [Ovis aries]
Accession: gi|426248712|ref|XP_004018103.1|
Sequence: R.LIDDMVAQVLK.S
Parent m/z: 622.884, 2+
Score: 54.65533473519328

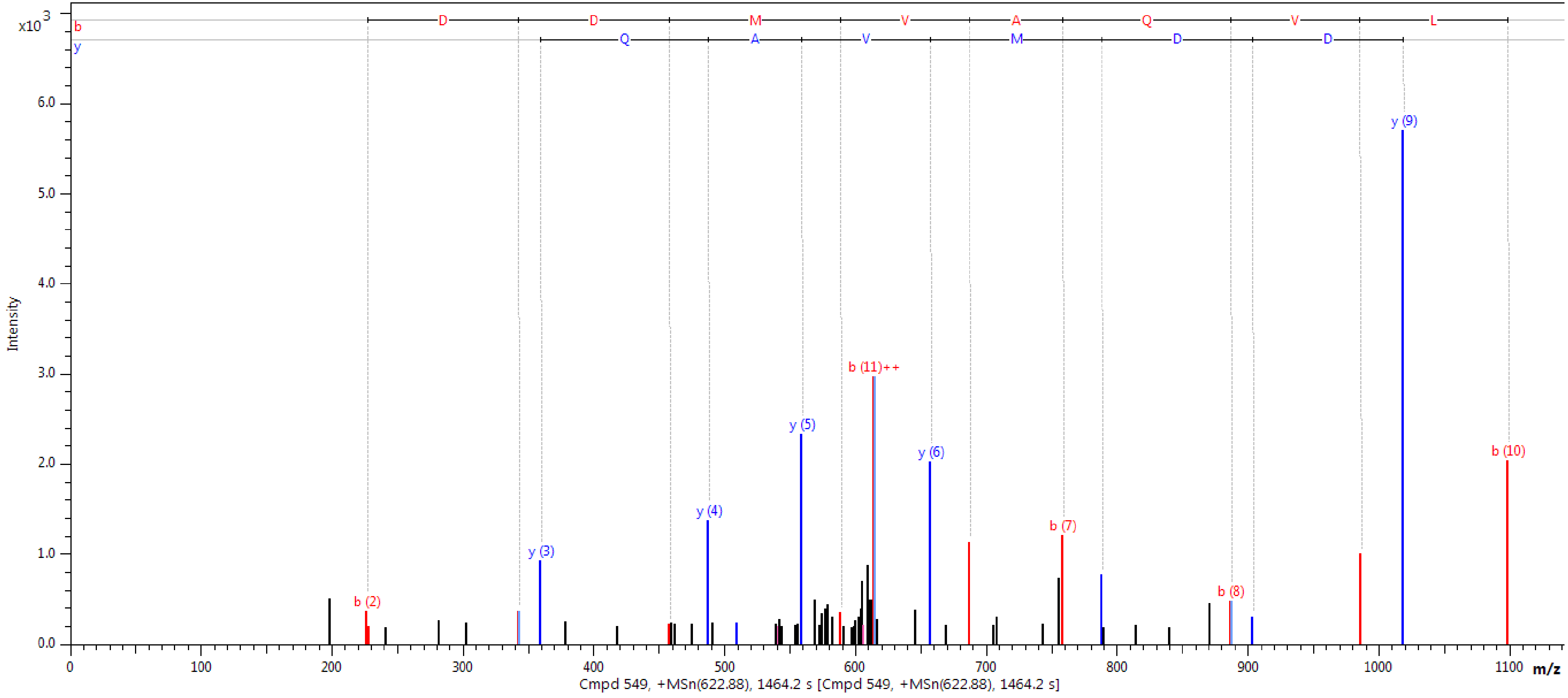

Spectrum Report

Source: M:/Documents/Lamb meat protein project/1. Characterisation of lamb skeletal proteome/Real run - 5 lambs from LCF/mgf_Obj_1/Myo_4-20pc_myo_15B-17B_concat_all_the_line_delet.mgf

Protein: similar to O-acetyl-ADP-ribose deacetylase MACROD1, partial [Ovis aries: Oar v3]

Accession: gi|1999002311|gb|1999002311.1|

Parent m/z: 576.332, 2+

Sequence: K.VDLSTSTDWK.E

Score: 55.279753022997184

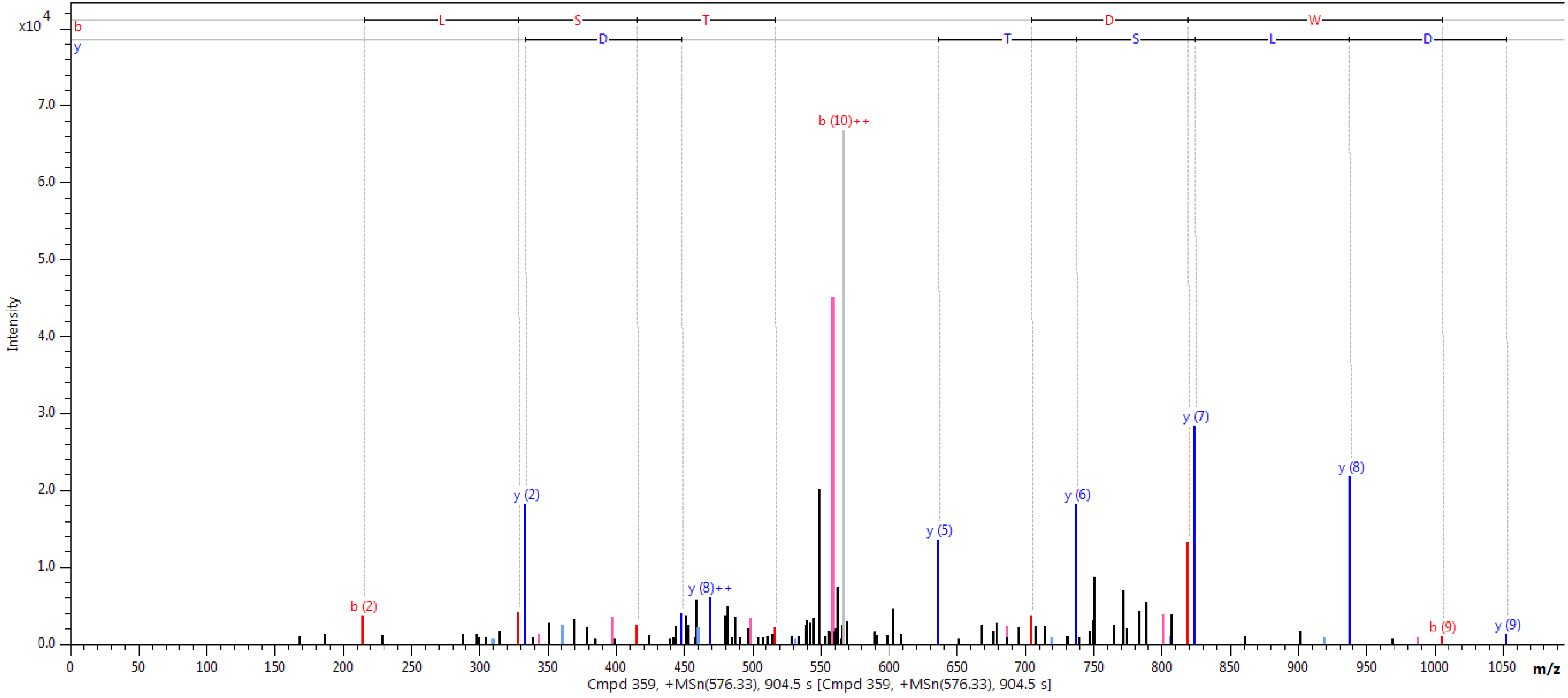

Spectrum Report

Source: M:/Documents/Lamb meat protein project/1. Characterisation of lamb skeletal proteome/Real run - 5 lambs from LCF/mgf_Obj_1/Myo_4-20pc_myo_15B-17B_concat_all_the_line_delet.mgf
Protein: PREDICTED: pyruvate dehydrogenase E1 component subunit beta, mitochondrial isoform 2 [Ovis aries]
Accession: gi|426249337|ref|XP_004018406.1|
Parent m/z: 621.361, 2+
Sequence: K.ILEDNSVPQVK.D
Score: 22.843208278191707

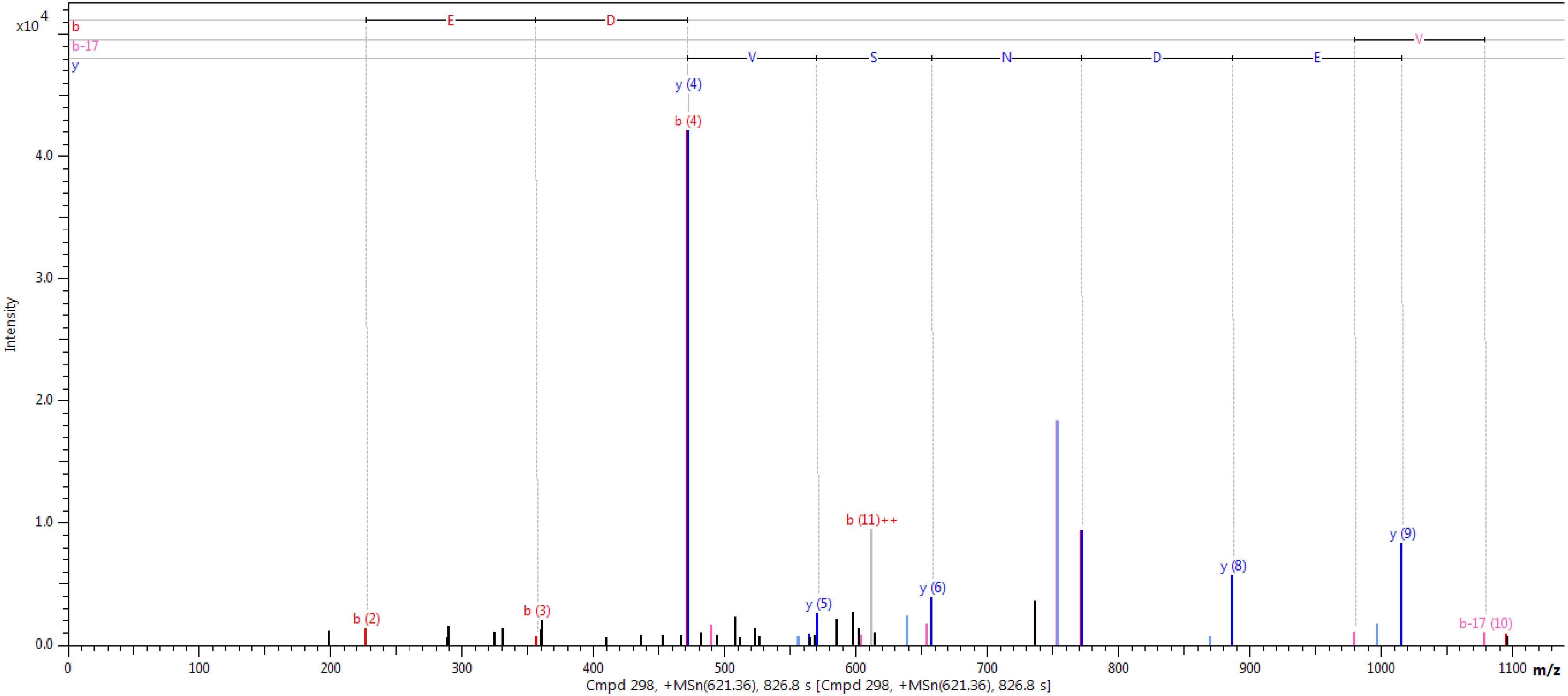

Spectrum Report

Source: M:/Documents/Lamb meat protein project/1. Characterisation of lamb skeletal proteome/Real run - 5 lambs from LCF/mgf_Obj_1/Myo_4-20pc_myo_15B-17B_concat_all_the_line_delet.mgf
Protein: PREDICTED: four and a half LIM domains protein 3 isoform 1 [Ovis aries]
Accession: gi|426215212|ref|XP_004001868.1|
Sequence: K.TLTQGGVTYR.D
Parent m/z: 548.319, 2+
Score: 36.50868233393256

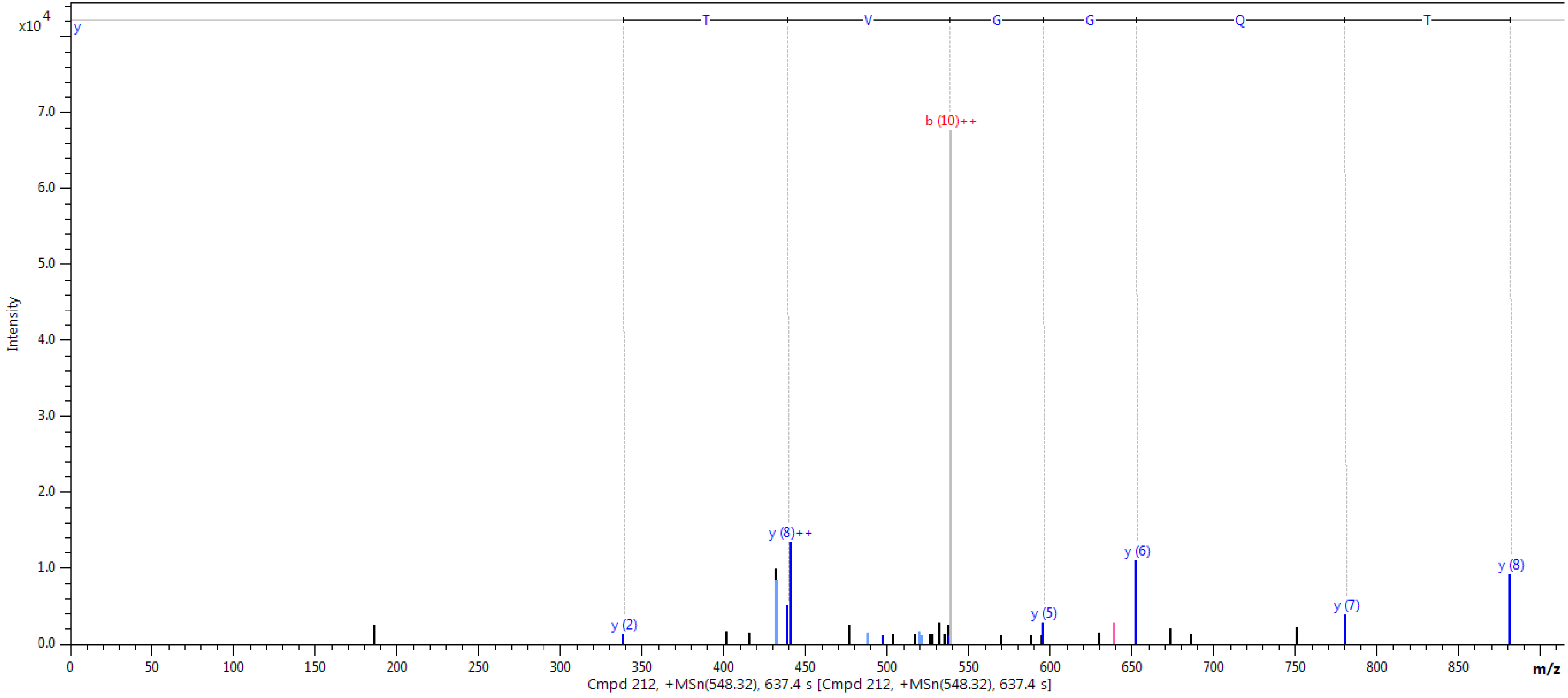

Spectrum Report

Source: M:/Documents/Lamb meat protein project/1. Characterisation of lamb skeletal proteome/Real run - 5 lambs from LCF/mgf_Obj_1/Myo_4-20pc_myo_15B-17B_concat_all_the_line_delet.mgf
Protein: PREDICTED: apolipoprotein O [Ovis aries]
Accession: gi|426256784|ref|XP_004022017.1|
Sequence: R.TQLEESISHLR.H
Parent m/z: 656.908, 2+
Score: 27.06142653645781

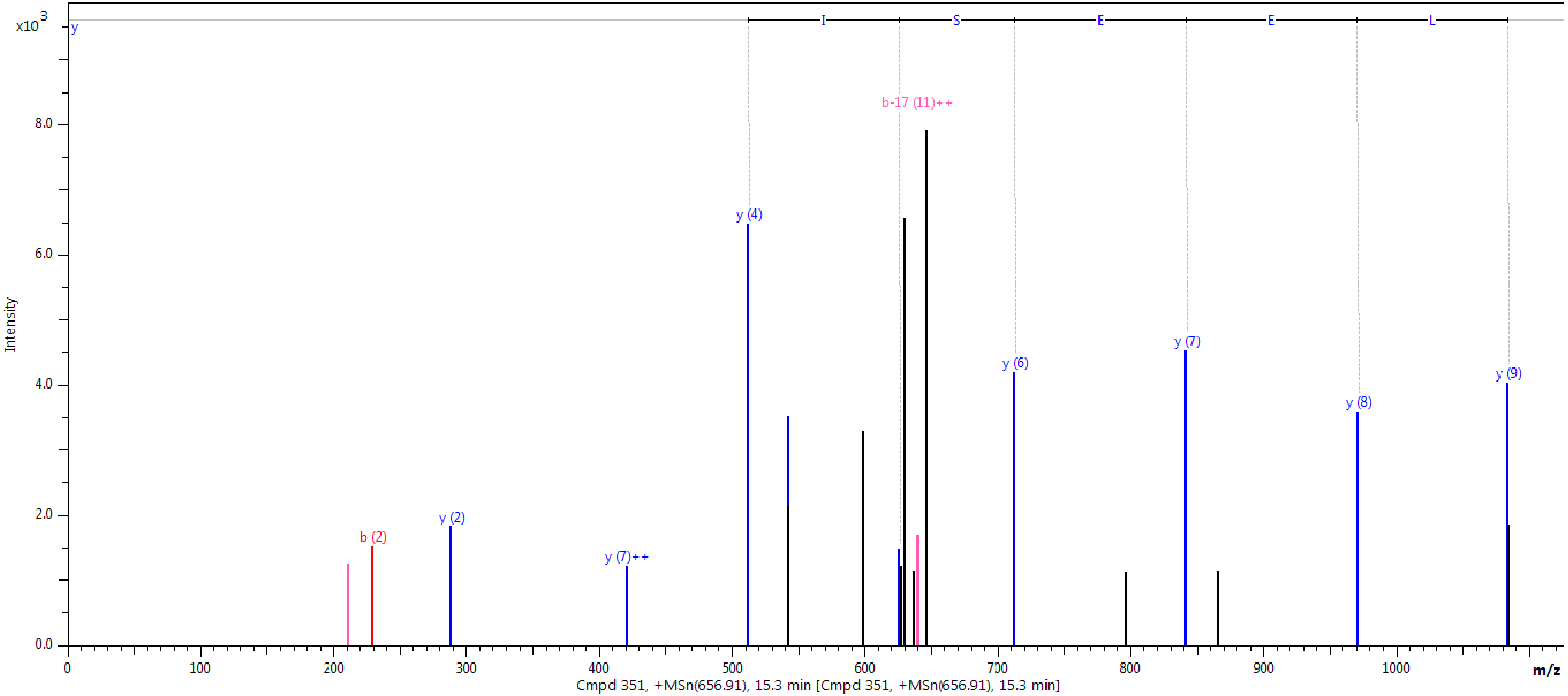

Spectrum Report

Source: M:/Documents/Lamb meat protein project/1. Characterisation of lamb skeletal proteome/Real run - 5 lambs from LCF/mgf_Obj_1/Myo_4-20pc_myo_15B-17B_concat_all_the_line_delet.mgf

Protein: PREDICTED: microsomal glutathione S-transferase 3 isoform 1 [Ovis aries]

Accession: gi|426217057|ref|XP_004002770.1| **Parent m/z:** 776.402, 2+

Sequence: R.VLYAYGYYTGEPR.K **Score:** 50.44408930647357

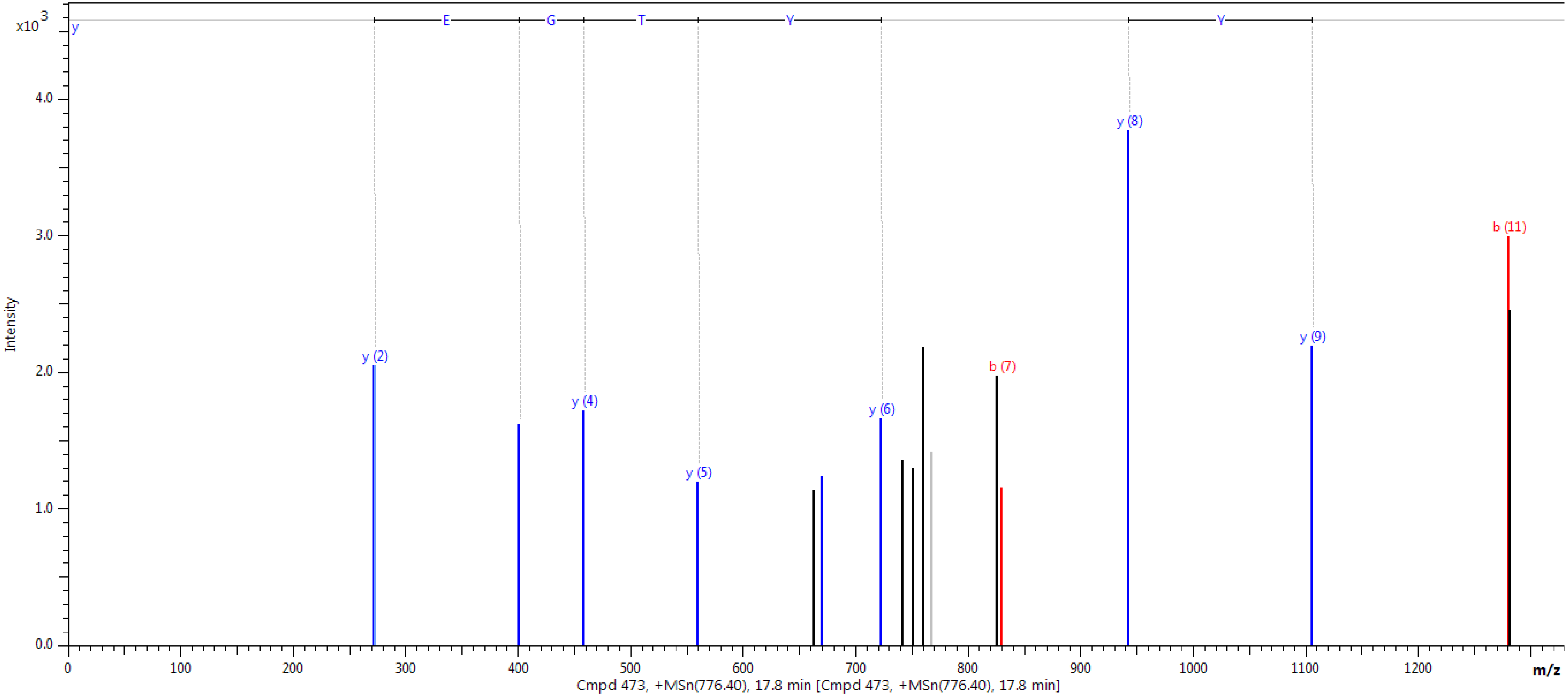

Spectrum Report

Source: M:/Documents/Lamb meat protein project/1. Characterisation of lamb skeletal proteome/Real run - 5 lambs from LCF/mgf_Obj_1/Myo_4-20pc_myo_15B-17B_concat_all_the_line_delet.mgf
Protein: PREDICTED: F-actin-capping protein subunit beta [Ovis aries]
Accession: gi|426222052|ref|XP_004005219.1|
Parent m/z: 677.317, 2+
Sequence: K.SGSGTMNLGGSLTR.Q
Score: 26.979267800819745

Modification: Oxidation: 6

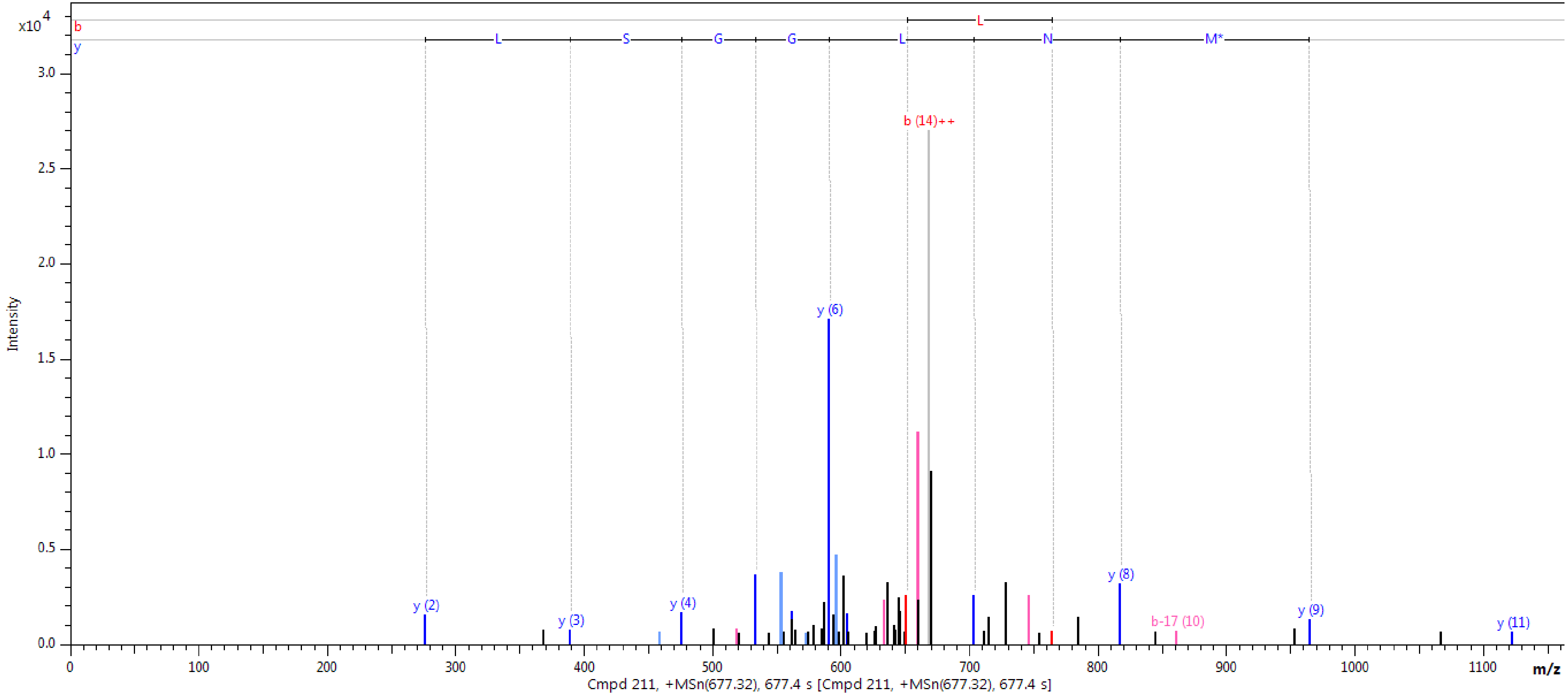

agresearch

Spectrum Report

Source: M:/Documents/Lamb meat protein project/1. Characterisation of lamb skeletal proteome/Real run - 5 lambs from LCF/mgf_Obj_1/Myo_4-20pc_myo_15B-17B_concat_all_the_line_delet.mgf
Protein: PREDICTED: T-complex protein 1 subunit beta [Ovis aries]
Accession: gi|426224723|ref|XP_004006518.1| **Parent m/z:** 752.486, 2+
Sequence: R.LSSFIGAIAIGDLVK.S **Score:** 24.81761200430949

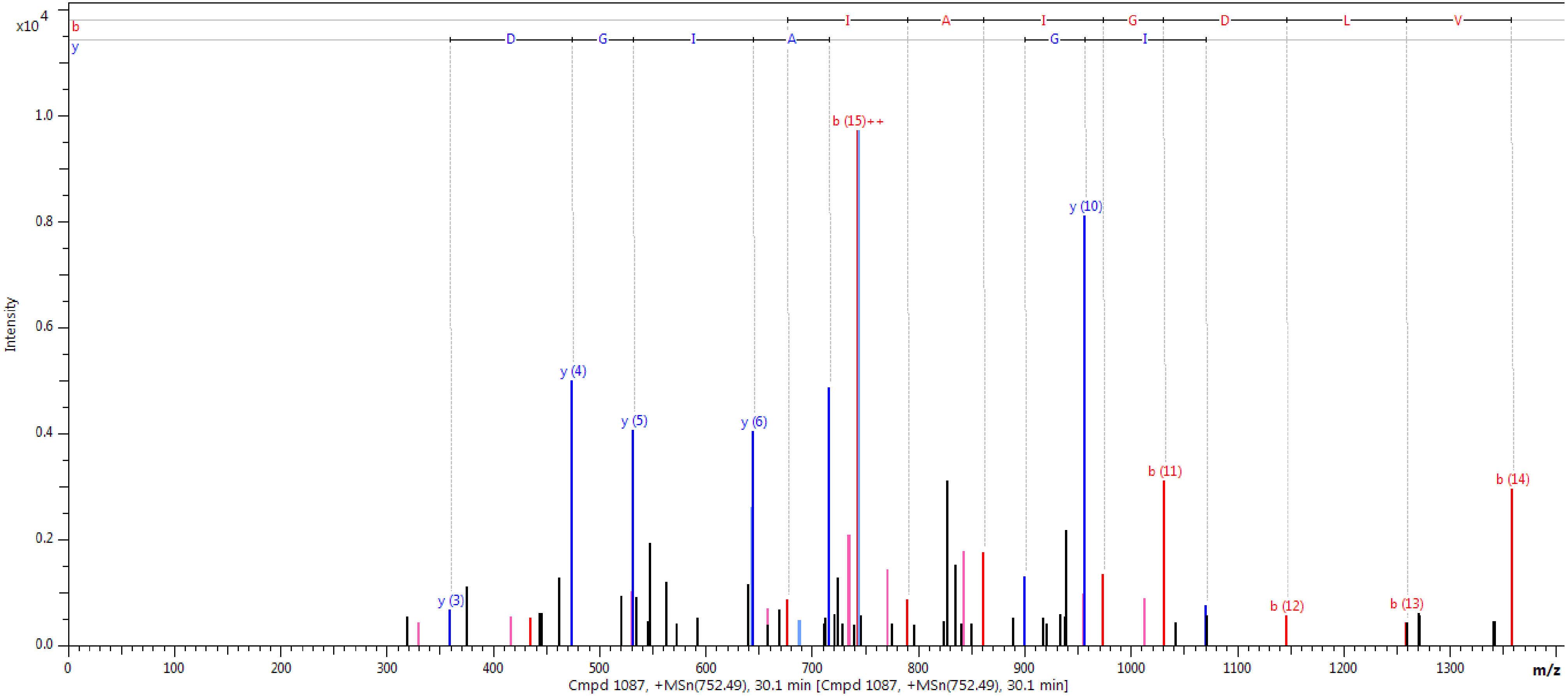

Spectrum Report

Source: M:/Documents/Lamb meat protein project/1. Characterisation of lamb skeletal proteome/Real run - 5 lambs from LCF/mgf_Obj_1/Myo_4-20pc_myo_15B-17B_concat_all_the_line_delet.mgf
Protein: PREDICTED: phosphoglucomutase-like protein 5 [Ovis aries]
Accession: gi|426220364|ref|XP_004004386.1|
Parent m/z: 886.043, 2+
Sequence: R.VEIVDPVDIYLNLLR.T
Score: 27.063038407956654

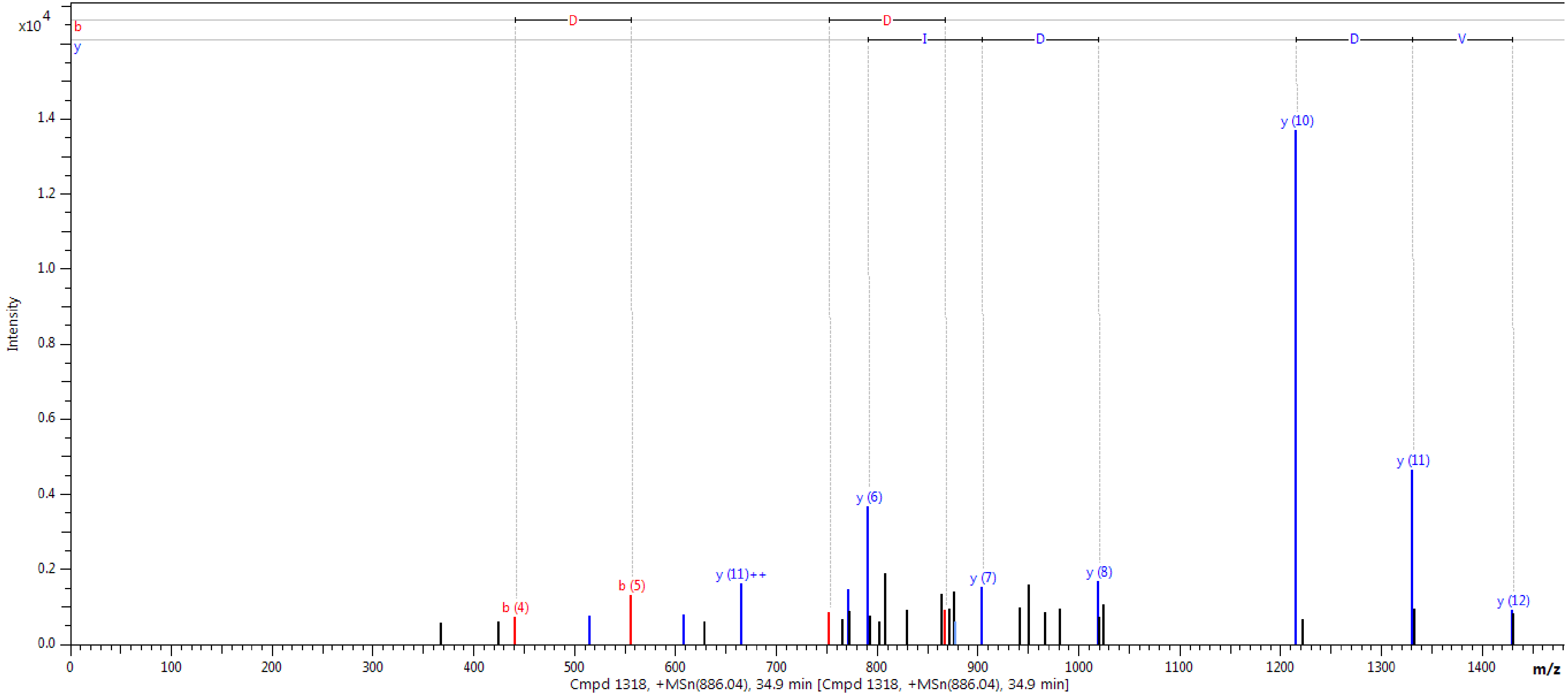

Spectrum Report

Source: M:/Documents/Lamb meat protein project/1. Characterisation of lamb skeletal proteome/Real run - 5 lambs from LCF/mgf_Obj_1/Myo_4-20pc_myo_15B-17B_concat_all_the_line_delet.mgf
Protein: PREDICTED: cytochrome c oxidase subunit 7A1, mitochondrial-like [Ovis aries]
Accession: gi|426242757|ref|XP_004015237.1|
Parent m/z: 540.248, 2+
Sequence: K.GGATDNILYR.V
Score: 29.18433079710131

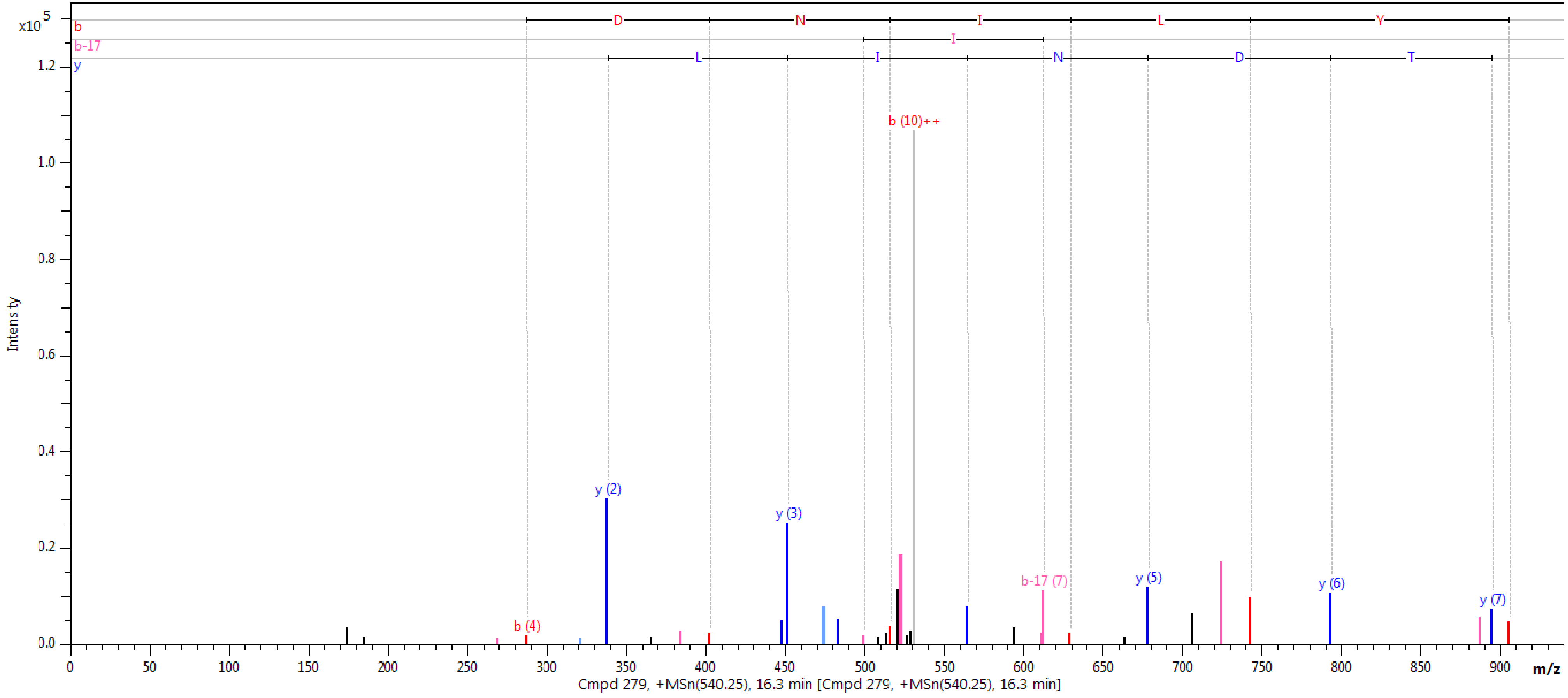

Spectrum Report

Source: M:/Documents/Lamb meat protein project/1. Characterisation of lamb skeletal proteome/Real run - 5 lambs from LCF/mgf_Obj_1/Myo_4-20pc_myo_15B-17B_concat_all_the_line_delet.mgf
Protein: PREDICTED: inositol 1,4,5-trisphosphate receptor type 1 isoform 2 [Ovis aries]
Accession: gi|426249236|ref|XP_004018356.1|
Parent m/z: 444.242, 2+
Sequence: K.QDLDQLR.S
Score: 26.71365603663427

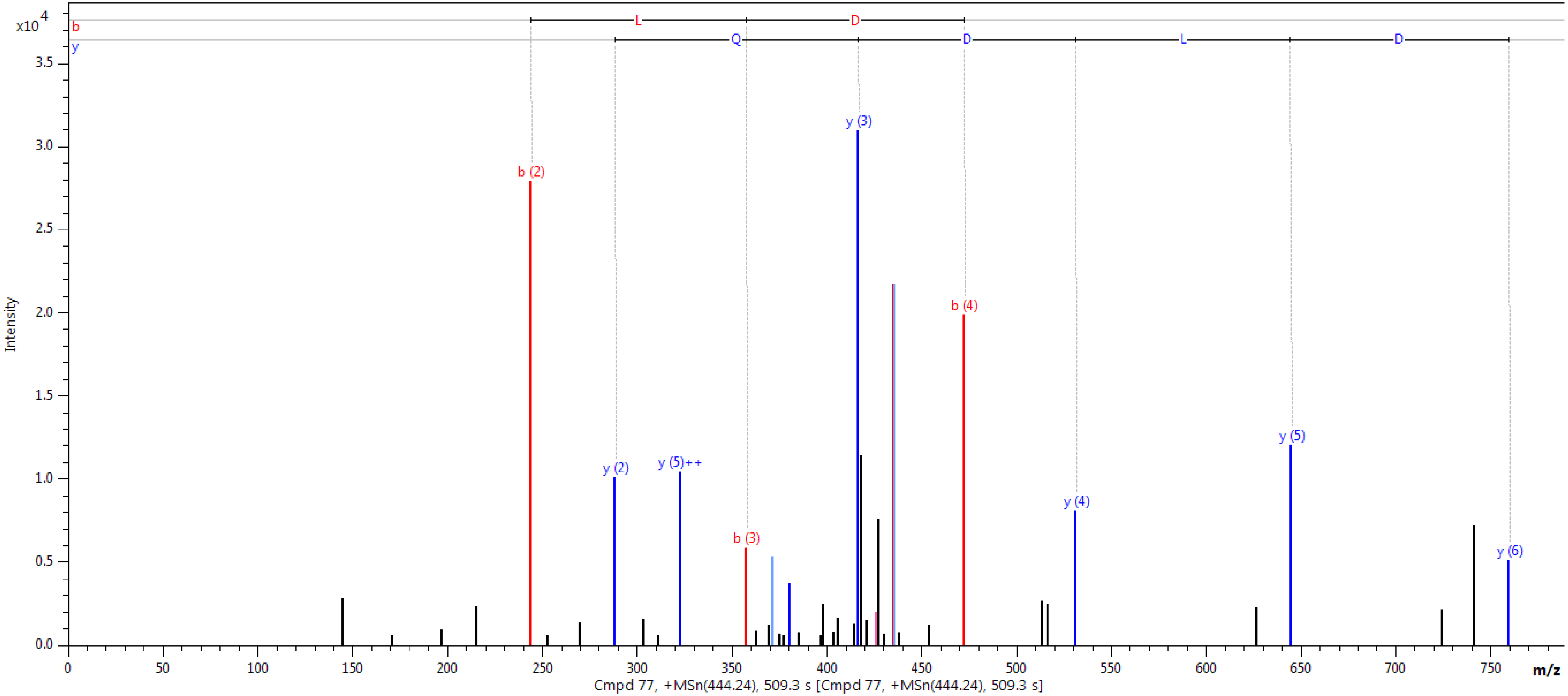

Spectrum Report

Source: M:/Documents/Lamb meat protein project/1. Characterisation of lamb skeletal proteome/Real run - 5 lambs from LCF/mgf_Obj_1/Myo_4-20pc_myo_15B-17B_concat_all_the_line_delet.mgf
Protein: PREDICTED: NADH dehydrogenase [ubiquinone] 1 alpha subcomplex subunit 11 [Ovis aries]
Accession: gi|426229103|ref|XP_004008632.1| **Parent m/z:** 589.416, 2+
Sequence: K.TPTSFLEGVAR.T **Score:** 55.8439404526471

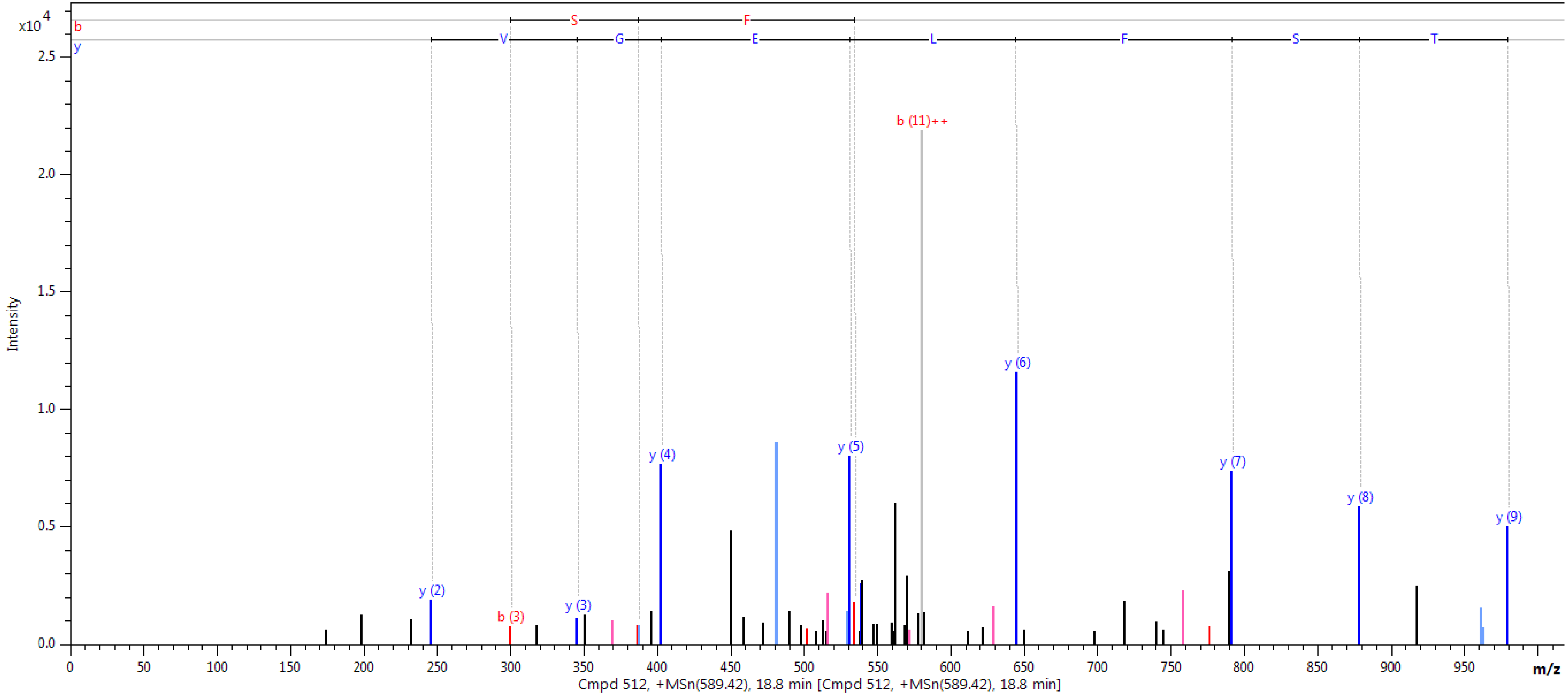

Spectrum Report

Source: M:/Documents/Lamb meat protein project/1. Characterisation of lamb skeletal proteome/Real run - 5 lambs from LCF/mgf_Obj_1/Myo_4-20pc_myo_15B-17B_concat_all_the_line_delet.mgf
Protein: PREDICTED: laminin subunit gamma-1 [Ovis aries]
Accession: gi|426240008|ref|XP_004013907.1|
Sequence: R.LSAEDLVLEGAGLR.V
Parent m/z: 721.927, 2+
Score: 52.78967196066244

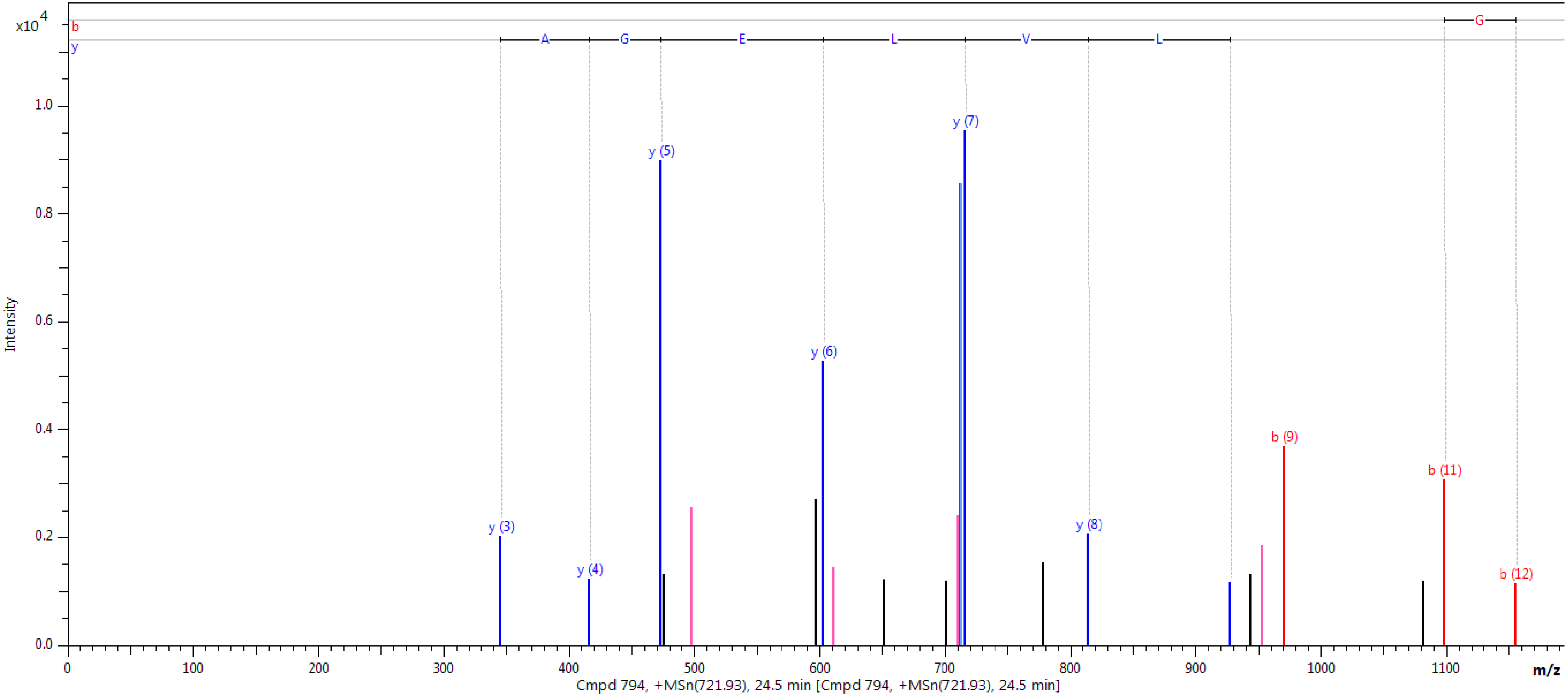

Spectrum Report

Source: M:/Documents/Lamb meat protein project/1. Characterisation of lamb skeletal proteome/Real run - 5 lambs from LCF/mgf_Obj_1/Myo_4-20pc_myo_15B-17B_concat_all_the_line_delet.mgf
Protein: PREDICTED: 60S ribosomal protein L18 [Ovis aries]
Accession: gi|426243101|ref|XP_004015402.1|
Sequence: K.ILTFDQLALDSPK.G
Parent m/z: 730.947, 2+
Score: 24.797300642731862

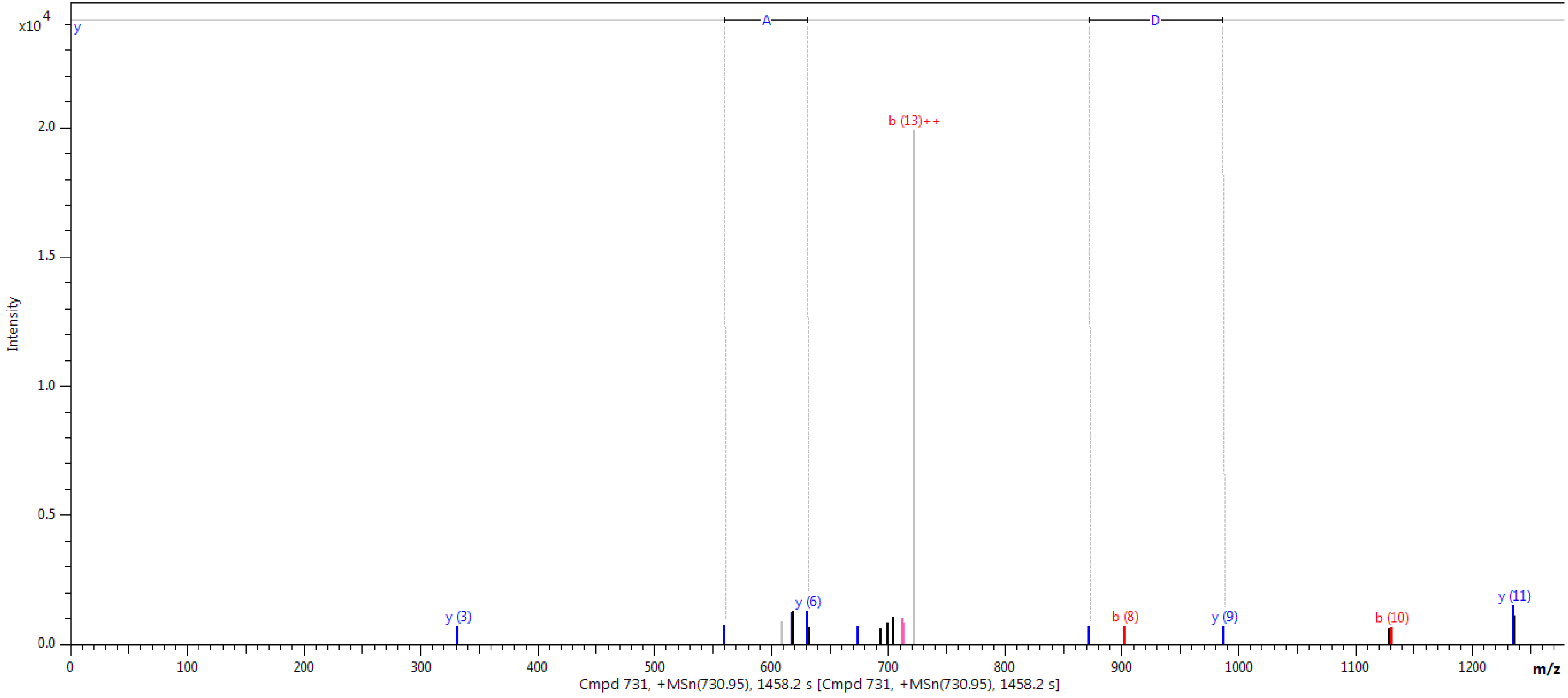

agresearch

Spectrum Report

Source: M:/Documents/Lamb meat protein project/1. Characterisation of lamb skeletal proteome/Real run - 5 lambs from LCF/ mgf_Obj_1/Myo_4-20pc_myo_15B-17B_concat_all_the_line_delet.mgf
Protein: PREDICTED: enoyl-CoA hydratase, mitochondrial-like [Ovis aries]
Accession: gi|426253507|ref|XP_004020434.1|
Parent m/z: 563.853, 2+
Sequence: G.AAFEYIITAK.K
Score: 34.607030445740484

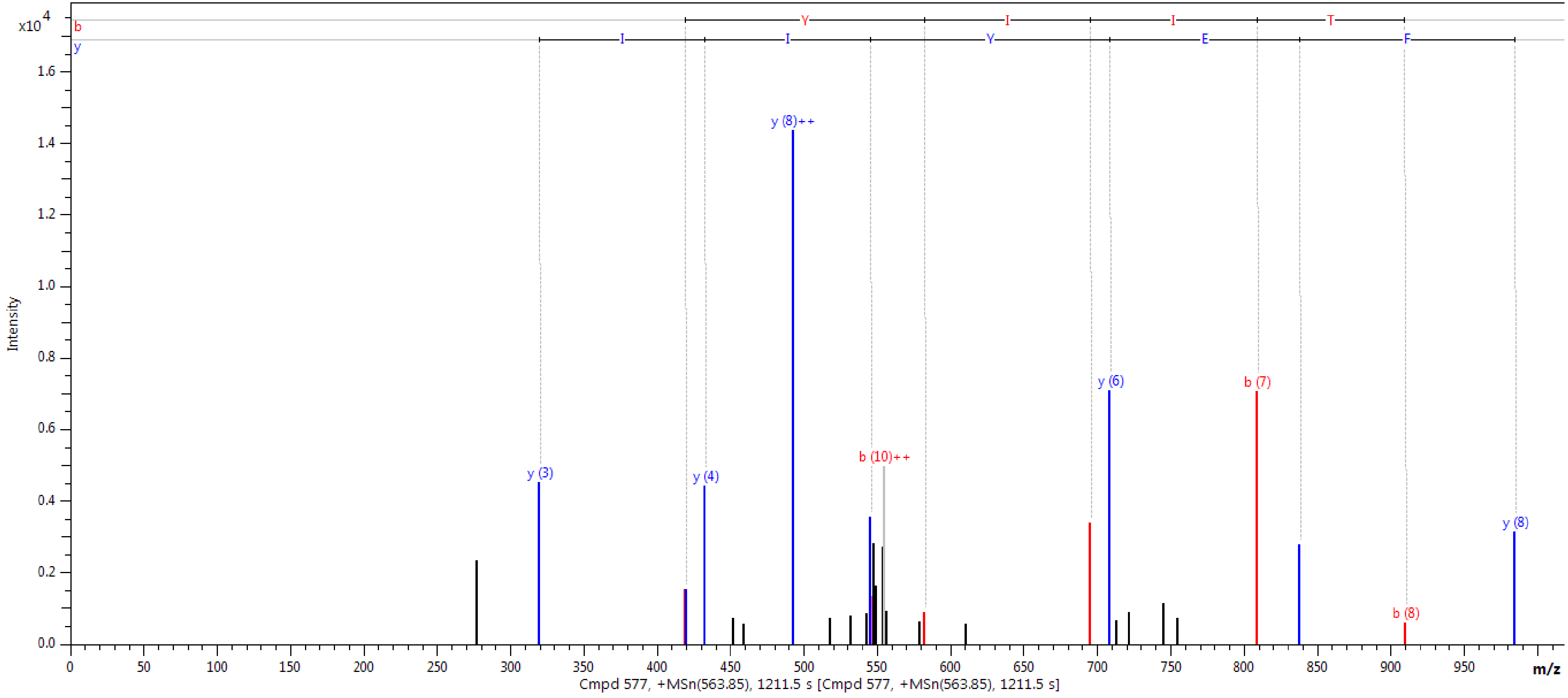

Spectrum Report

Source: M:/Documents/Lamb meat protein project/1. Characterisation of lamb skeletal proteome/Real run - 5 lambs from LCF/mgf_Obj_1/Myo_4-20pc_myo_15B-17B_concat_all_the_line_delet.mgf
Protein: PREDICTED: carbonyl reductase family member 4-like [Ovis aries]
Accession: gi|426258168|ref|XP_004022690.1| **Parent m/z:** 644.422, 2+
Sequence: R.VNFLVNAAGINR.D **Score:** 44.067239041407866

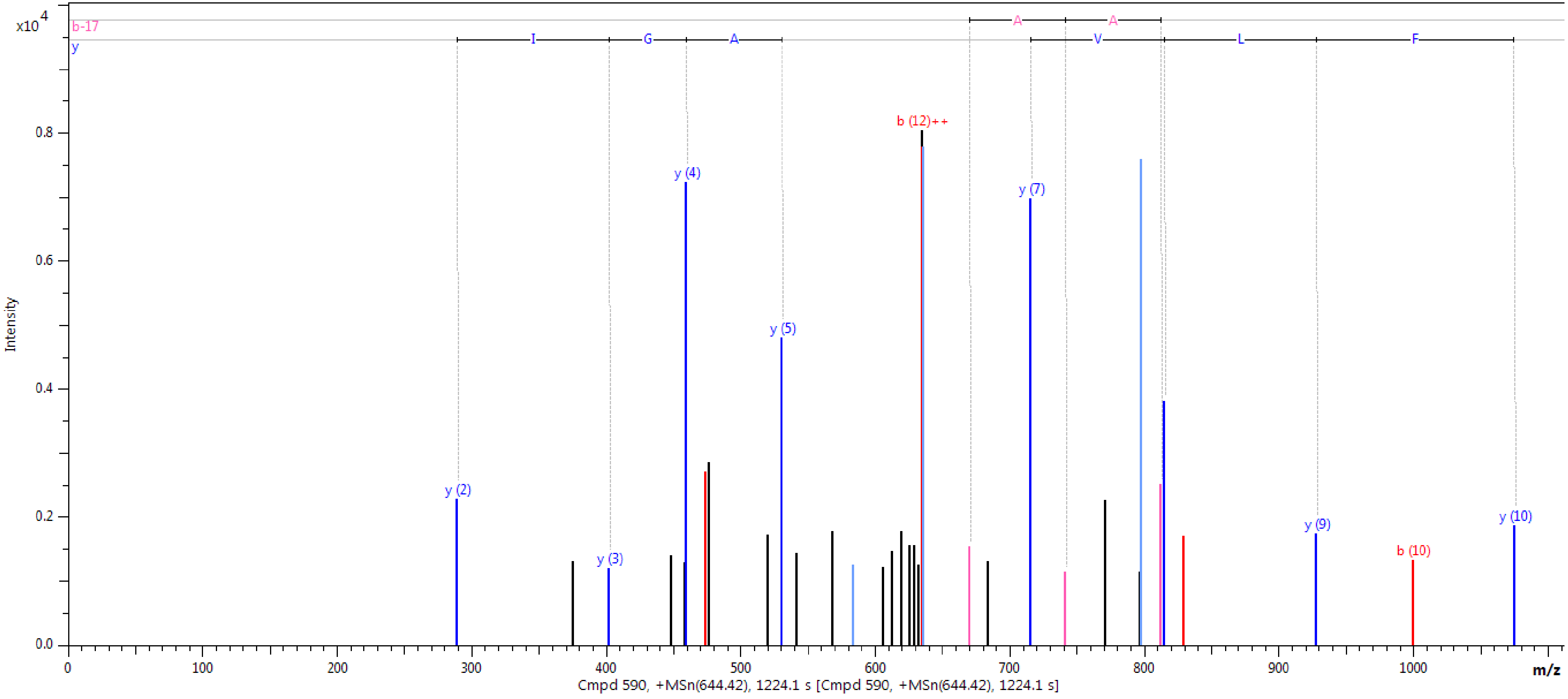

Spectrum Report

Source: M:/Documents/Lamb meat protein project/1. Characterisation of lamb skeletal proteome/Real run - 5 lambs from LCF/ mgf_Obj_1/Myo_4-20pc_myo_15B-17B_concat_all_the_line_delet.mgf
Protein: PREDICTED: vacuolar protein sorting-associated protein 35 [Ovis aries]
Accession: gi|426242306|ref|XP_004015015.1|
Parent m/z: 801.995, 2+
Sequence: K.IPVDTYNNILTVLK.L
Score: 35.72017559860578

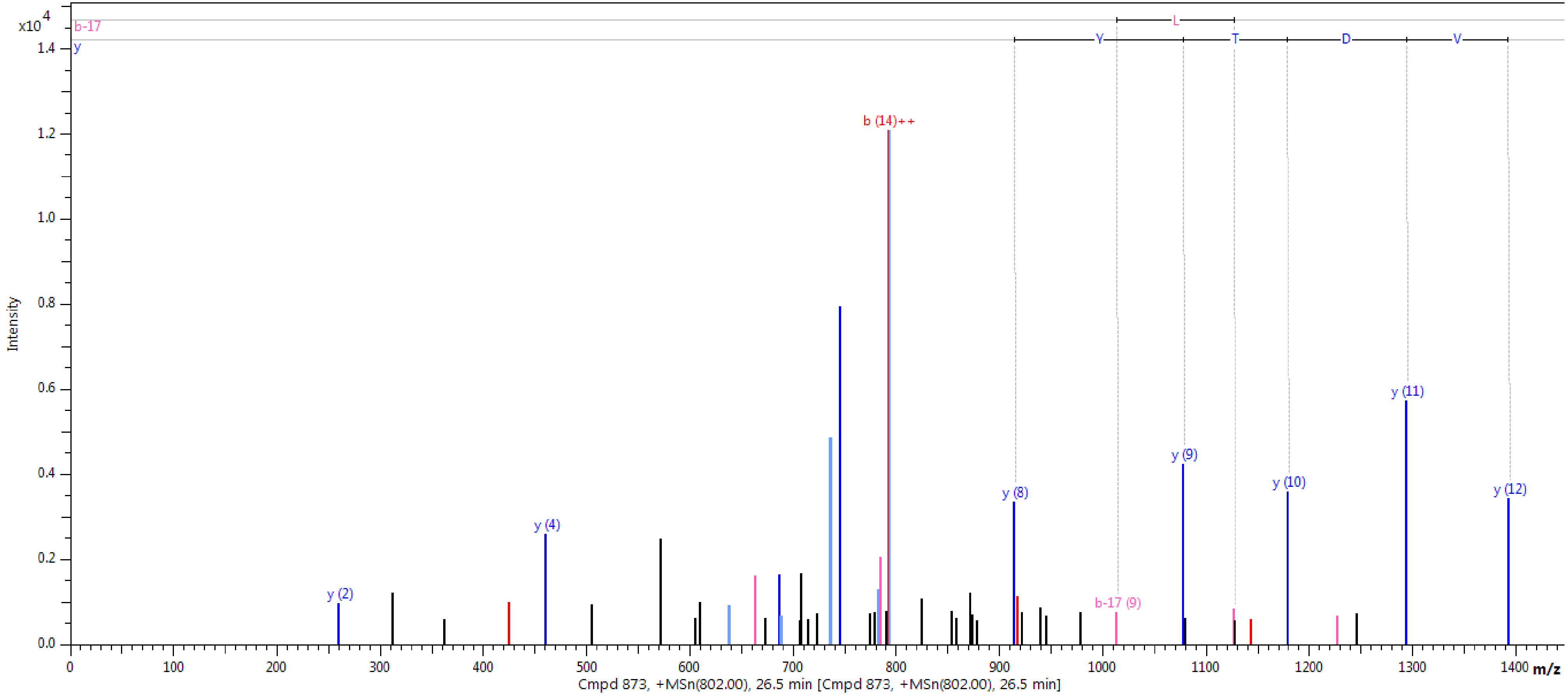

Spectrum Report

Source: M:/Documents/Lamb meat protein project/1. Characterisation of lamb skeletal proteome/Real run - 5 lambs from LCF/mgf_Obj_1/Myo_4-20pc_myo_15B-17B_concat_all_the_line_delet.mgf
Protein: carnitine palmitoyltransferase I [Ovis aries]
Accession: gi|18958223|emb|CAC81315.1|
Sequence: R.QALLDIANLFQVSK.A
Parent m/z: 780.515, 2+
Score: 43.16071752027764

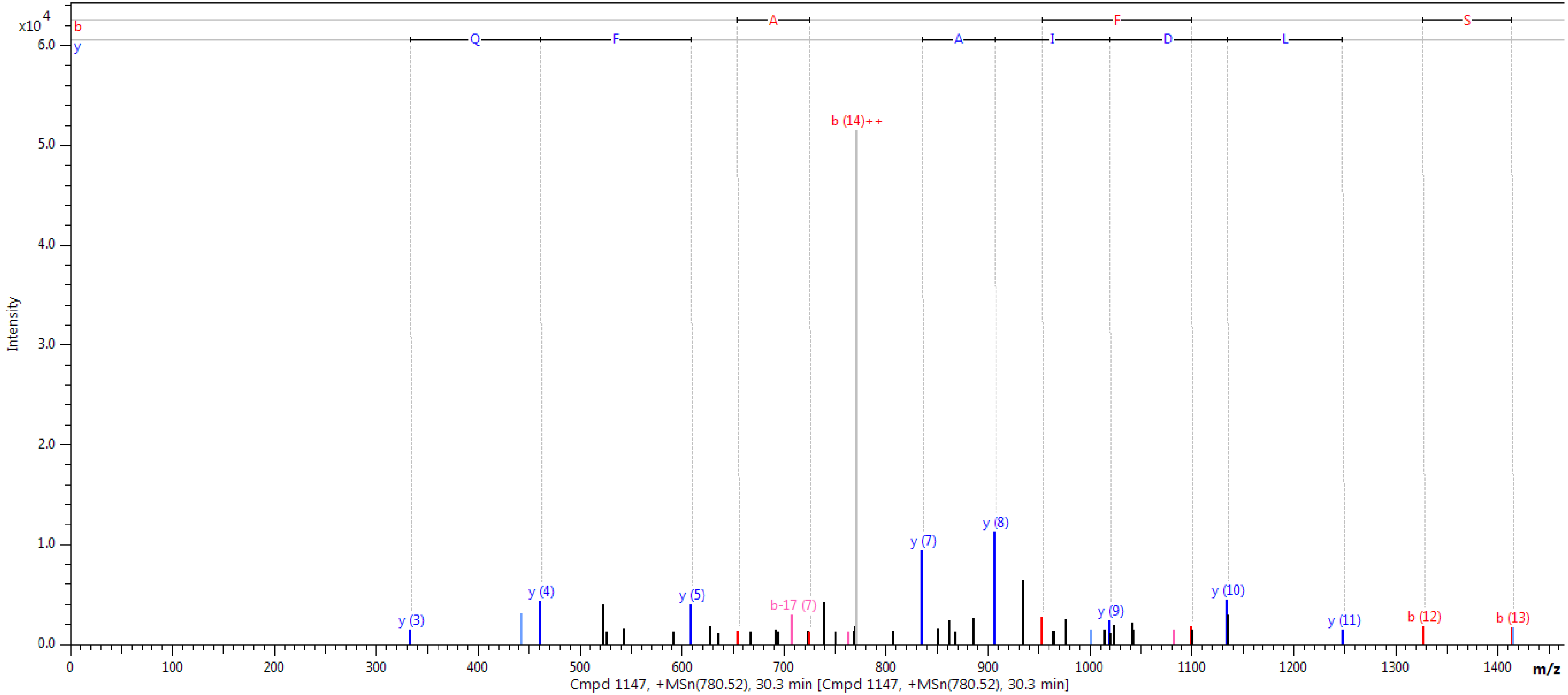

agresearch

Spectrum Report

Source: M:/Documents/Lamb meat protein project/1. Characterisation of lamb skeletal proteome/Real run - 5 lambs from LCF/mgf_Obj_1/Myo_4-20pc_myo_15B-17B_concat_all_the_line_delet.mgf
Protein: PREDICTED: low-density lipoprotein receptor-related protein 1B [Ovis aries]
Accession: gi|426221145|ref|XP_004004771.1| **Parent m/z:** 595.86, 2+
Sequence: R.LTDEWTISIL.Q **Score:** 27.47372666688641

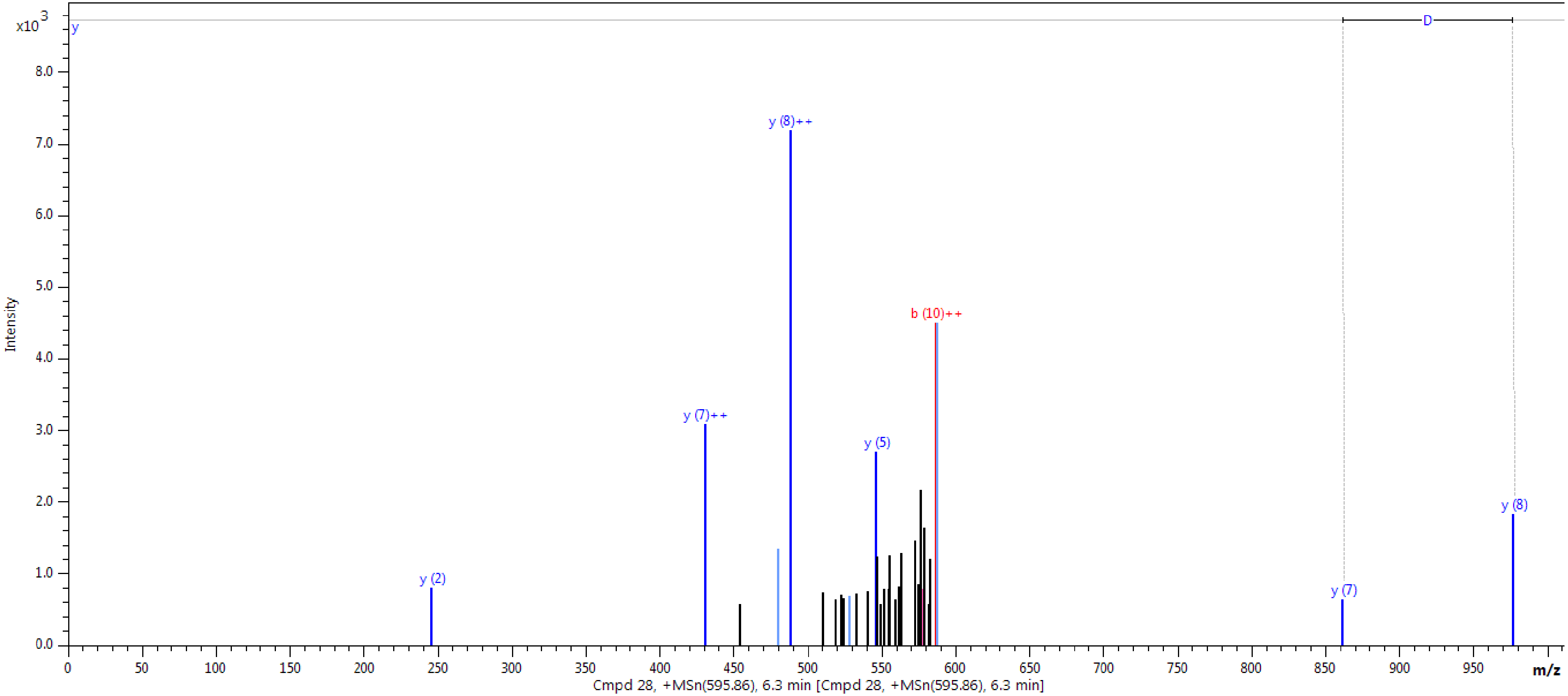

Spectrum Report

Source: M:/Documents/Lamb meat protein project/1. Characterisation of lamb skeletal proteome/Real run - 5 lambs from LCF/mgf_Obj_1/Myo_4-20pc_myo_15B-17B_concat_all_the_line_delet.mgf
Protein: PREDICTED: liver carboxylesterase-like isoform 2 [Ovis aries]
Accession: gi|426242363|ref|XP_004015042.1|
Parent m/z: 717.378, 2+
Sequence: G.LPPSPPVVDTAQGR.V
Score: 21.123241976123474

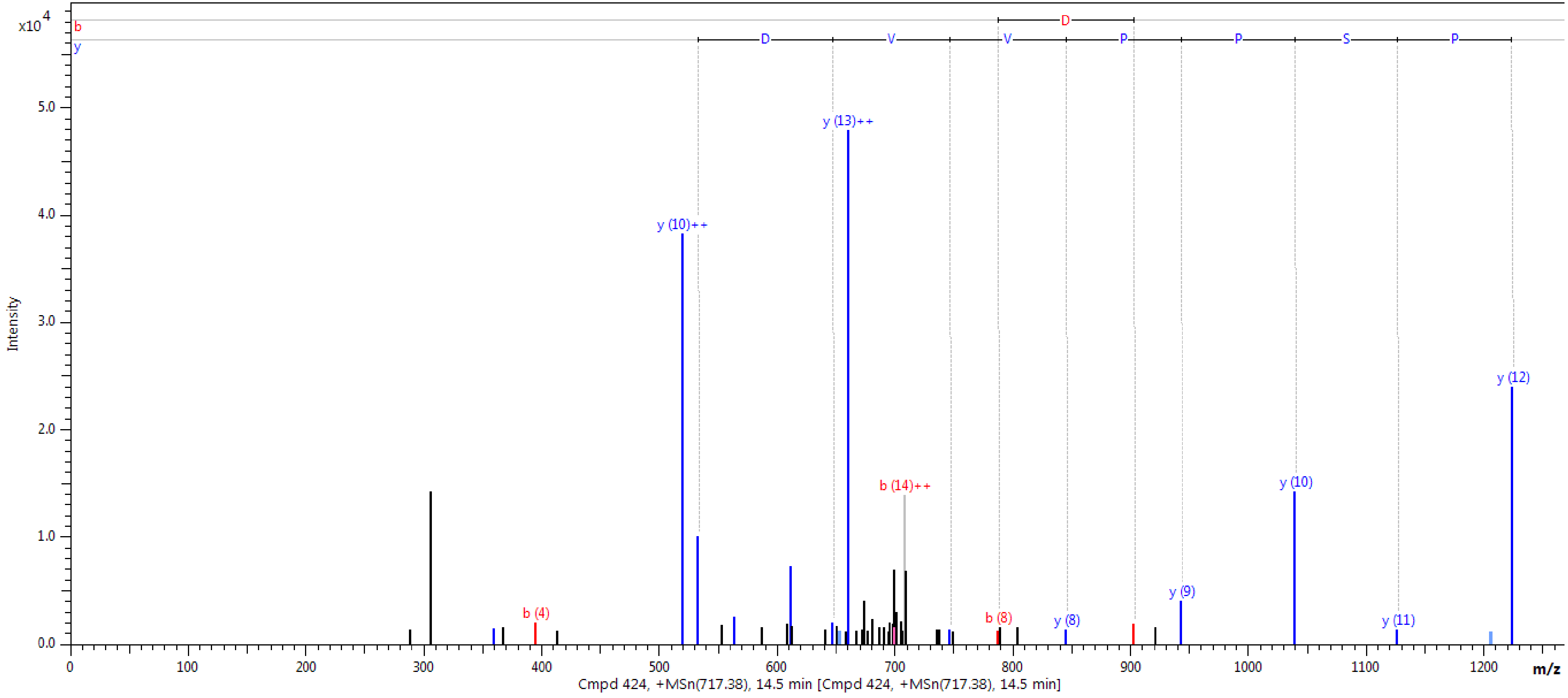

agresearch

Spectrum Report

Source: M:/Documents/Lamb meat protein project/1. Characterisation of lamb skeletal proteome/Real run - 5 lambs from LCF/mgf_Obj_1/Myo_4-20pc_myo_15B-17B_concat_all_the_line_delet.mgf
Protein: similar to Obscurin isoform IC, partial [Ovis aries: Oar v3]
Accession: gi|1999022426|gb|1999022426.1|
Parent m/z: 652.883, 2+
Sequence: R.LDVTEPSVVFAK.E
Score: 42.97462626466918

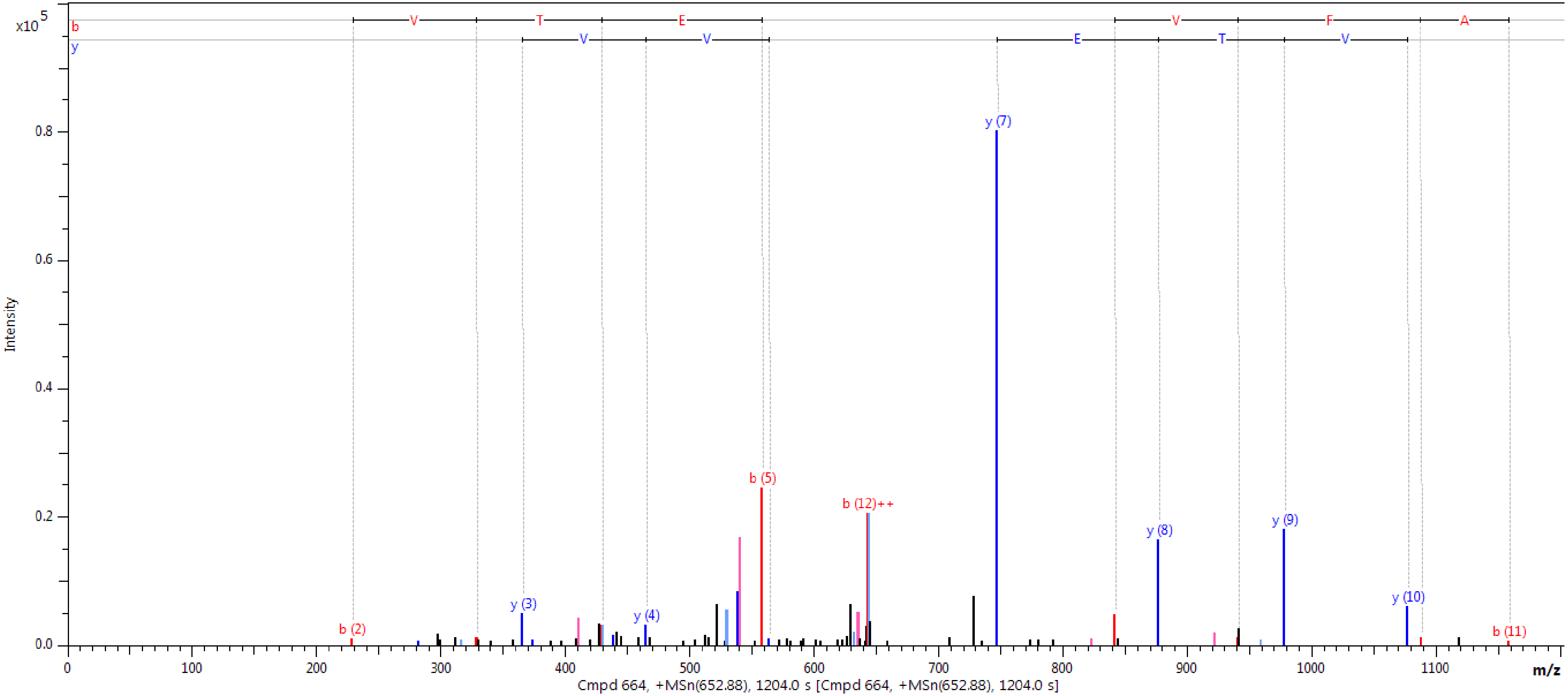

Spectrum Report

Source: M:/Documents/Lamb meat protein project/1. Characterisation of lamb skeletal proteome/Real run - 5 lambs from LCF/mgf_Obj_1/Myo_4-20pc_myo_15B-17B_concat_all_the_line_delet.mgf
Protein: PREDICTED: myc box-dependent-interacting protein 1-like, partial [Ovis aries]
Accession: gi|426258586|ref|XP_004022890.1|
Parent m/z: 806.997, 2+
Sequence: K.LNQNLNDVLVSLEK.Q
Score: 39.434267104591314

Modification: Methyl: 13

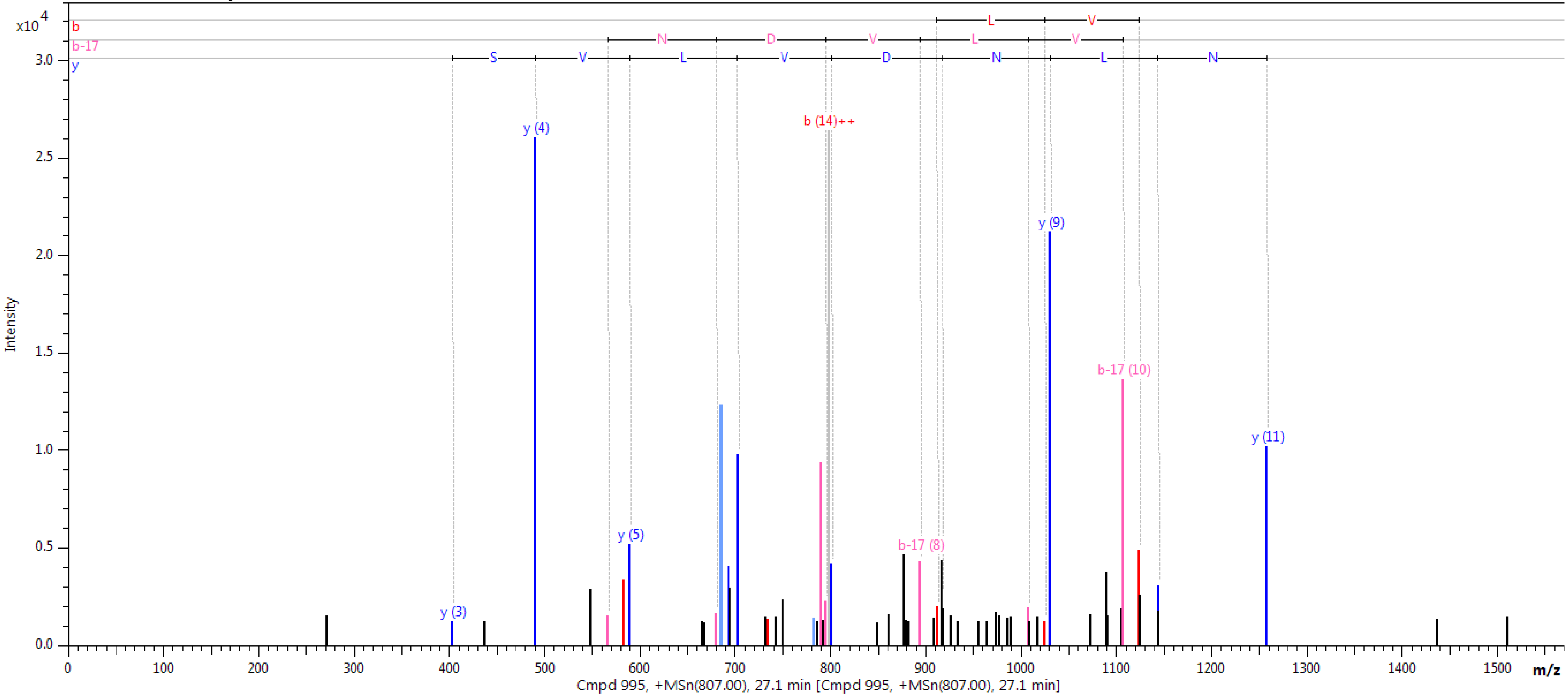

Supplement: Supplementary file 1 — Supplementary data [file mmc1.zip › Supple_data_3b_lamb_LL_prot_YMCD.pdf]
