# Supplementary material for: Data for in-depth characterisation of the lamb meat proteome from longissimus lumborum
Source: Data Brief. 2015 Feb 20;3:143–8. doi: 10.1016/j.dib.2015.02.006 (PMC4510072; doi:10.1016/j.dib.2015.02.006)

Spectrum Report

Source: M:/Documents/Lamb meat protein project/1. Characterisation of lamb skeletal proteome/Real run - 5 lambs from LCF/mgf_Obj_1/SDS-insoluble_pellet_mgf/u-3_undil_both_all_all_use_all_the_line_removed.mgf
Protein: PREDICTED: LOW QUALITY PROTEIN: myosin-3 [Ovis aries]
Accession: gi|426237623|ref|XP_004012757.1| **Parent m/z:** 632.314, 3+
Sequence: K.HADSVAELGEQIDNLQR.V **Score:** 31.932198930179982

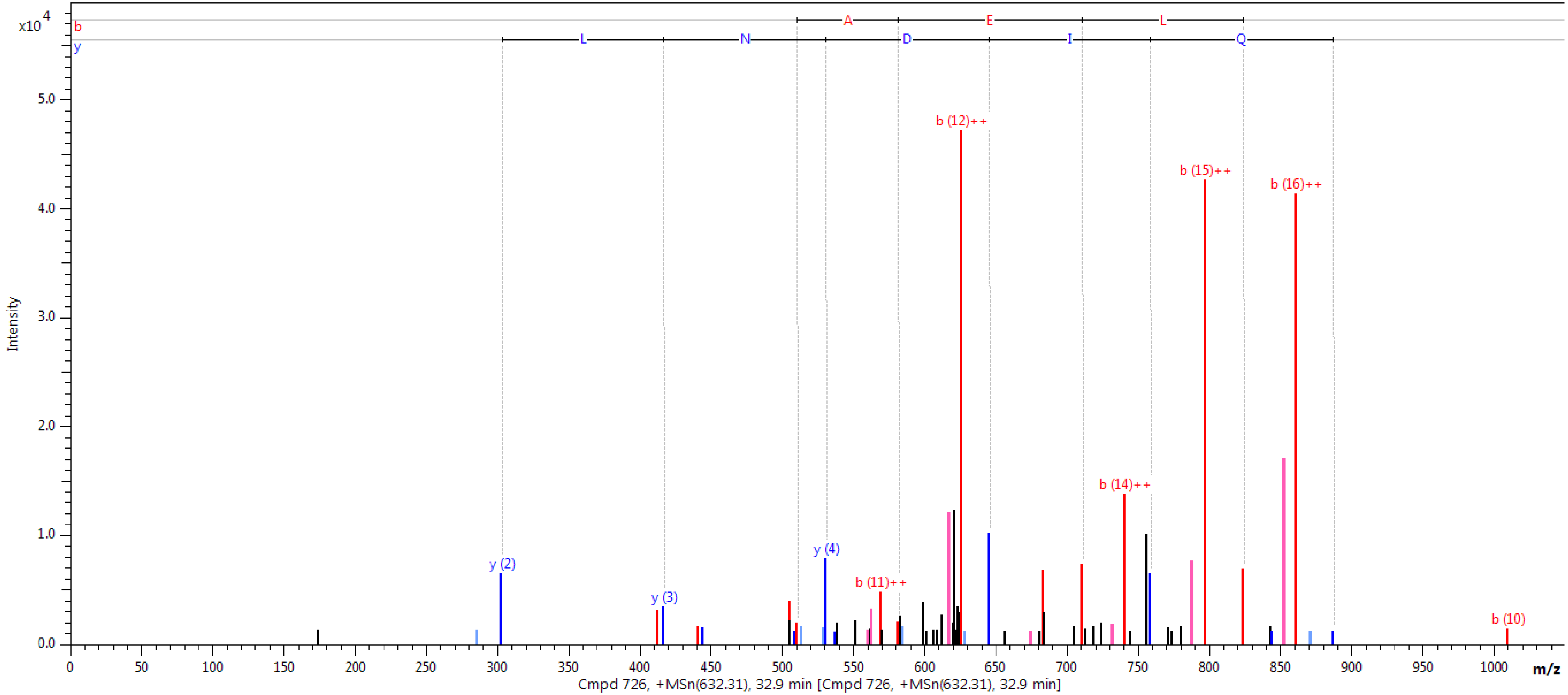

Spectrum Report

Source: M:/Documents/Lamb meat protein project/1. Characterisation of lamb skeletal proteome/Real run - 5 lambs from LCF/mgf_Obj_1/SDS-insoluble_pellet_mgf/u-3_undil_both_all_all_use_all_the_line_removed.mgf
Protein: PREDICTED: myosin-7 [Ovis aries]
Accession: gi|426232730|ref|XP_004010374.1|
Sequence: R.VIQYFAVIAAIGDR.S
Parent m/z: 768.341, 2+
Score: 29.989286152458412

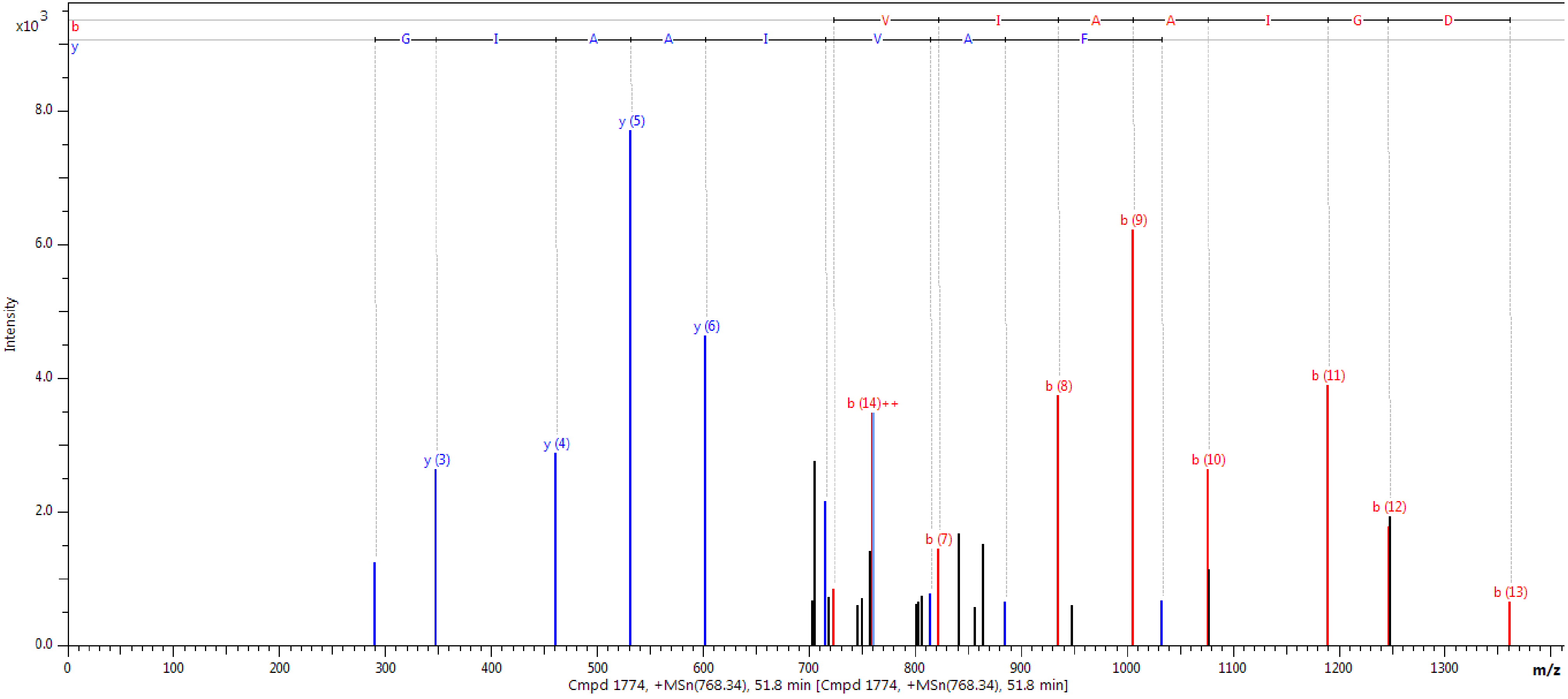

Spectrum Report

Source: M:/Documents/Lamb meat protein project/1. Characterisation of lamb skeletal proteome/Real run - 5 lambs from LCF/mgf_Obj_1/SDS-insoluble_pellet_mgf/u-3_undil_both_all_all_use_all_the_line_removed.mgf
Protein: PREDICTED: alpha-actinin-2 isoform 1 [Ovis aries]
Accession: gi|426255566|ref|XP_004021419.1|
Sequence: R.VGWELLLTTIAR.T
Parent m/z: 686.39, 2+
Score: 25.618723670269187

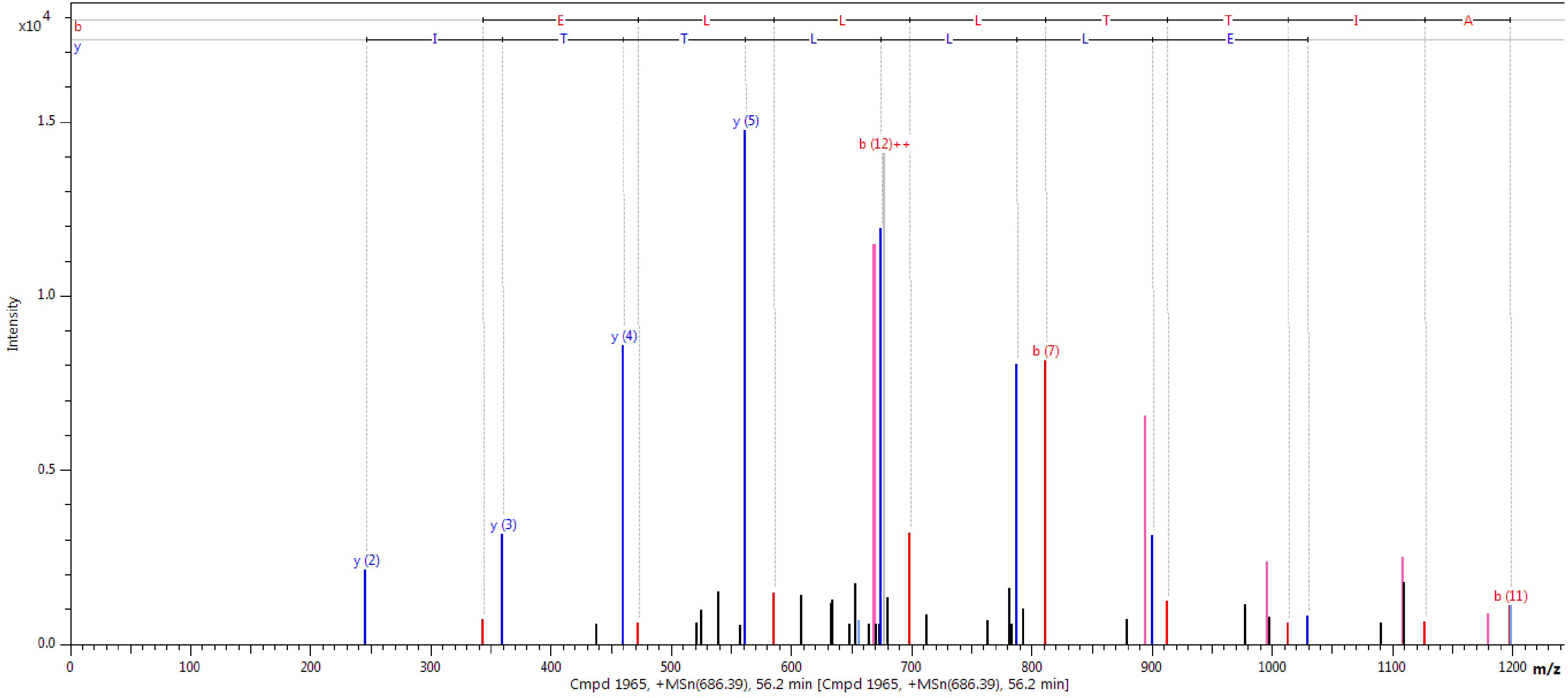

agresearch

Spectrum Report

Source: M:/Documents/Lamb meat protein project/1. Characterisation of lamb skeletal proteome/Real run - 5 lambs from LCF/mgf_Obj_1/SDS-insoluble_pellet_mgf/u-3_undil_both_all_all_use_all_the_line_removed.mgf
Protein: RecName: Full=Myoglobin
Accession: gi|124078333|sp|P02190.2|MYG_SHEEP
Sequence: K.HGNTVLTALGGILK.K
Parent m/z: 697.381, 2+
Score: 54.11074262065319

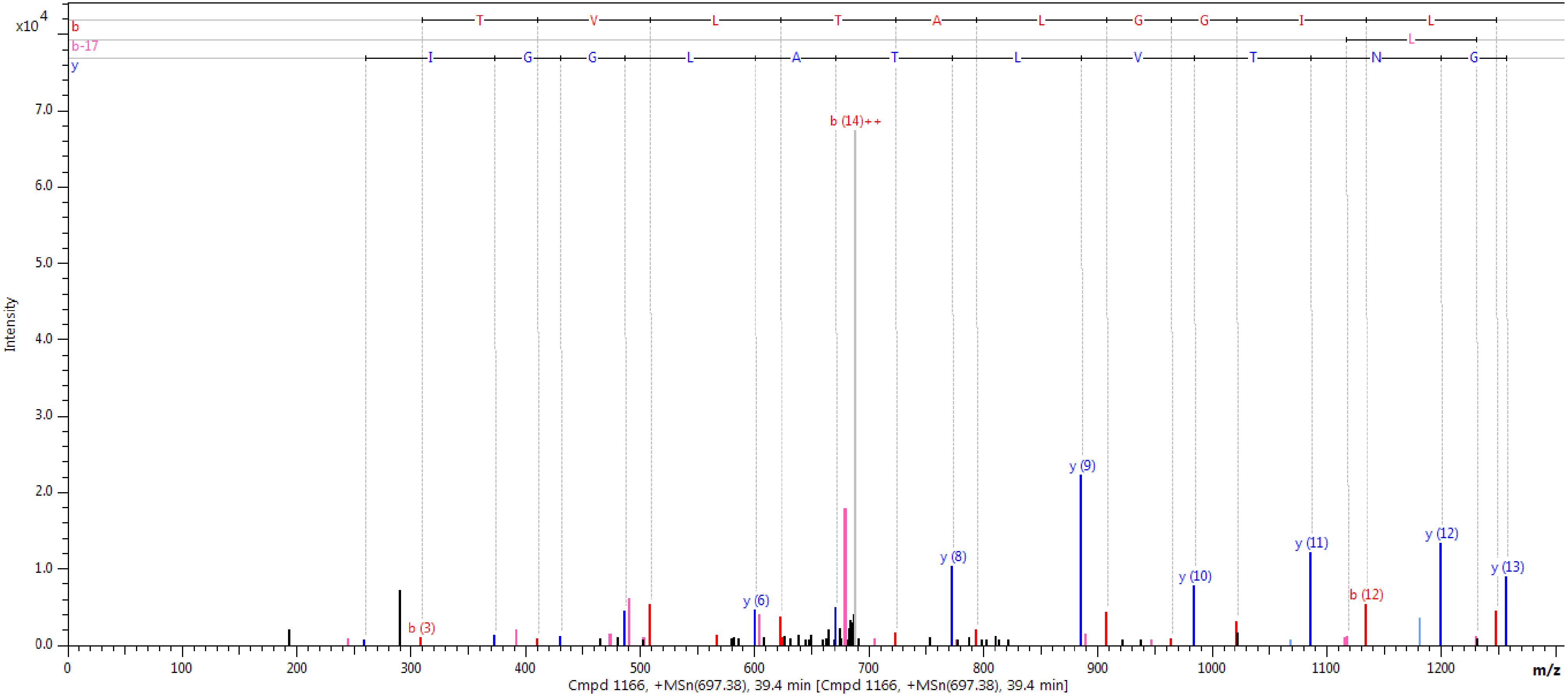

Spectrum Report

Source: M:/Documents/Lamb meat protein project/1. Characterisation of lamb skeletal proteome/Real run - 5 lambs from LCF/mgf_Obj_1/SDS-insoluble_pellet_mgf/u-3_undil_both_all_all_use_all_the_line_removed.mgf
Protein: PREDICTED: tropomyosin beta chain isoform 1 [Ovis aries]
Accession: gi|426220240|ref|XP_004004324.1|
Parent m/z: 769.803, 2+
Sequence: K.TIDDLEDEVYAQK.M
Score: 31.97076520228711

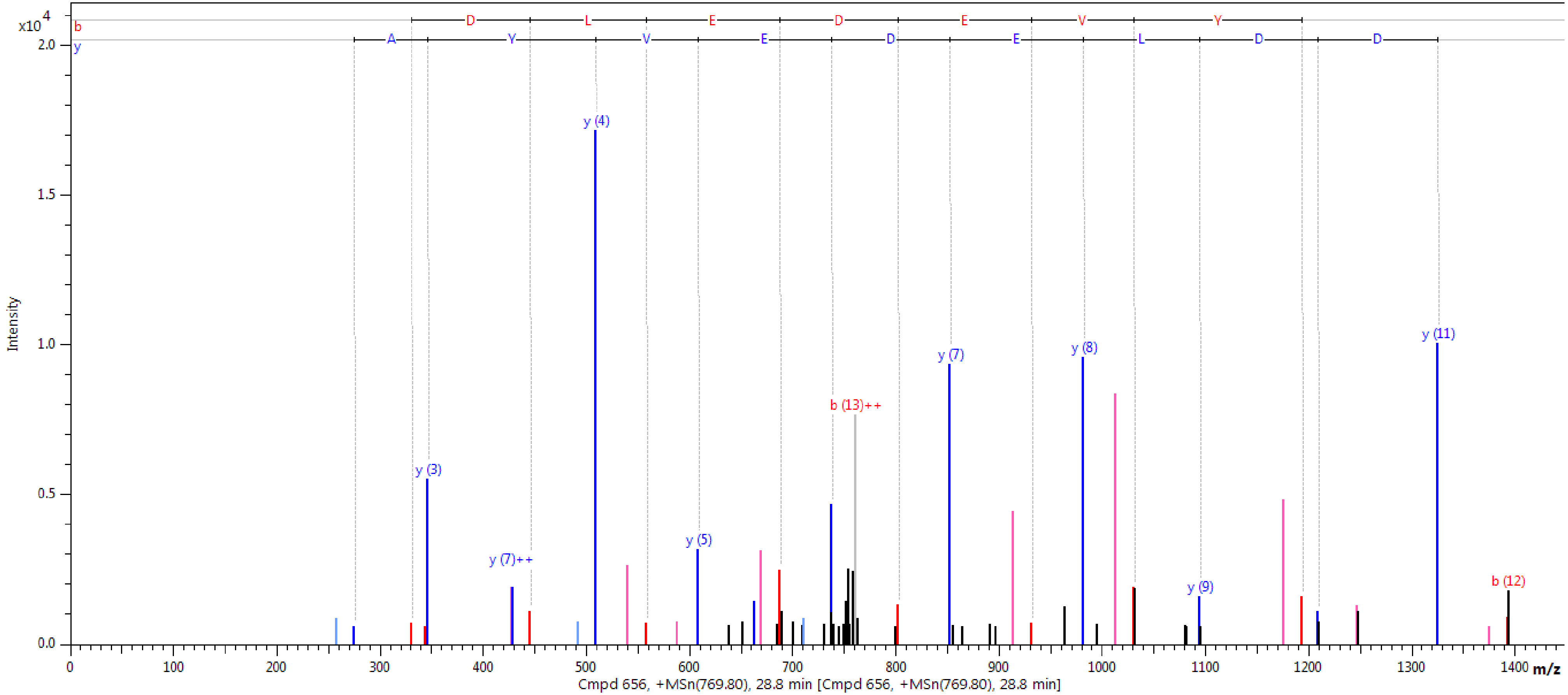

Spectrum Report

Source: M:/Documents/Lamb meat protein project/1. Characterisation of lamb skeletal proteome/Real run - 5 lambs from LCF/mgf_Obj_1/SDS-insoluble_pellet_mgf/u-3_undil_both_all_all_use_all_the_line_removed.mgf
Protein: PREDICTED: myozenin-1 [Ovis aries]
Accession: gi|426255772|ref|XP_004021522.1|
Parent m/z: 766.852, 2+
Sequence: R.DVMLEELSLLTNR.G
Score: 45.176882195146106

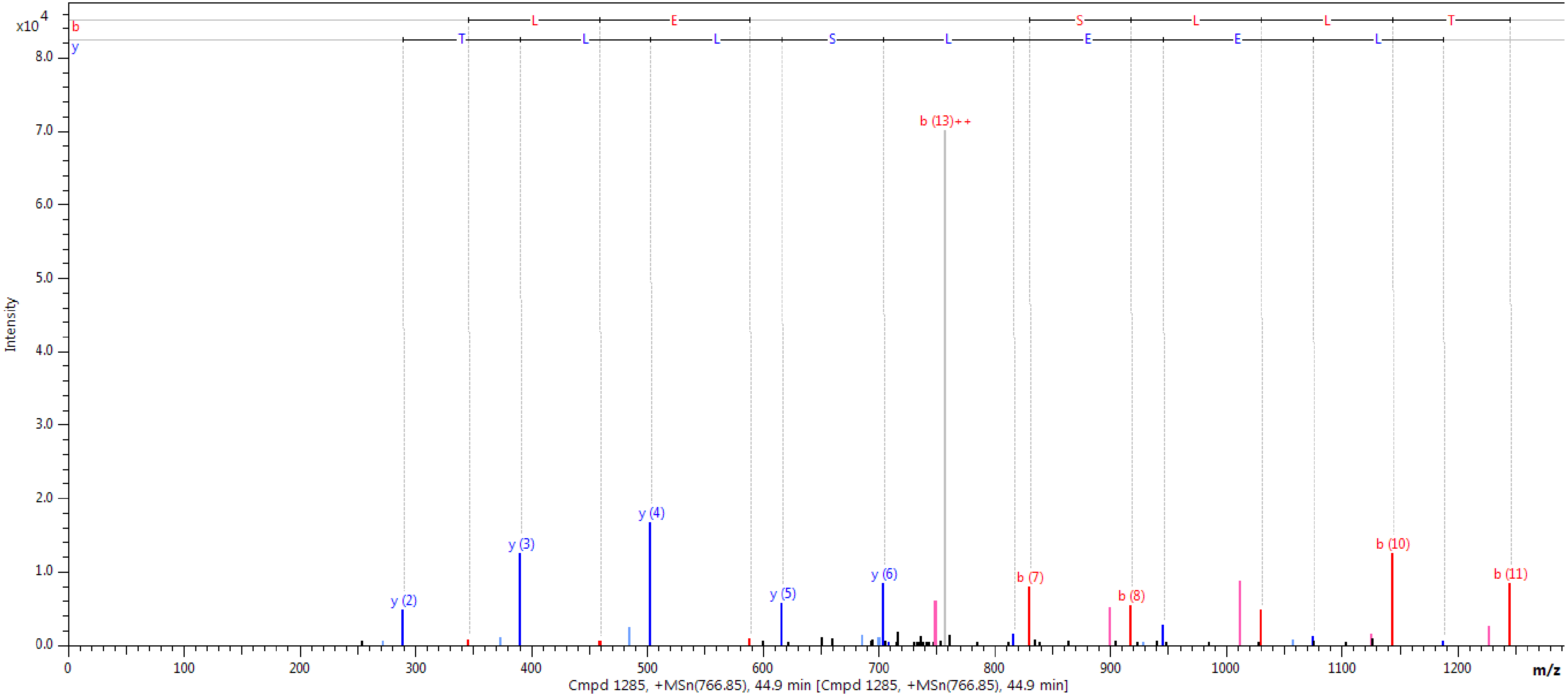

Spectrum Report

Source: M:/Documents/Lamb meat protein project/1. Characterisation of lamb skeletal proteome/Real run - 5 lambs from LCF/mgf_Obj_1/SDS-insoluble_pellet_mgf/u-3_undil_both_all_all_use_all_the_line_removed.mgf

Protein: PREDICTED: troponin C, skeletal muscle [Ovis aries]

Accession: gi|426242099|ref|XP_004014914.1|

Parent m/z: 723.809, 2+

Sequence: R.SYLSEEMIAEFK.A

Score: 43.664780183991795

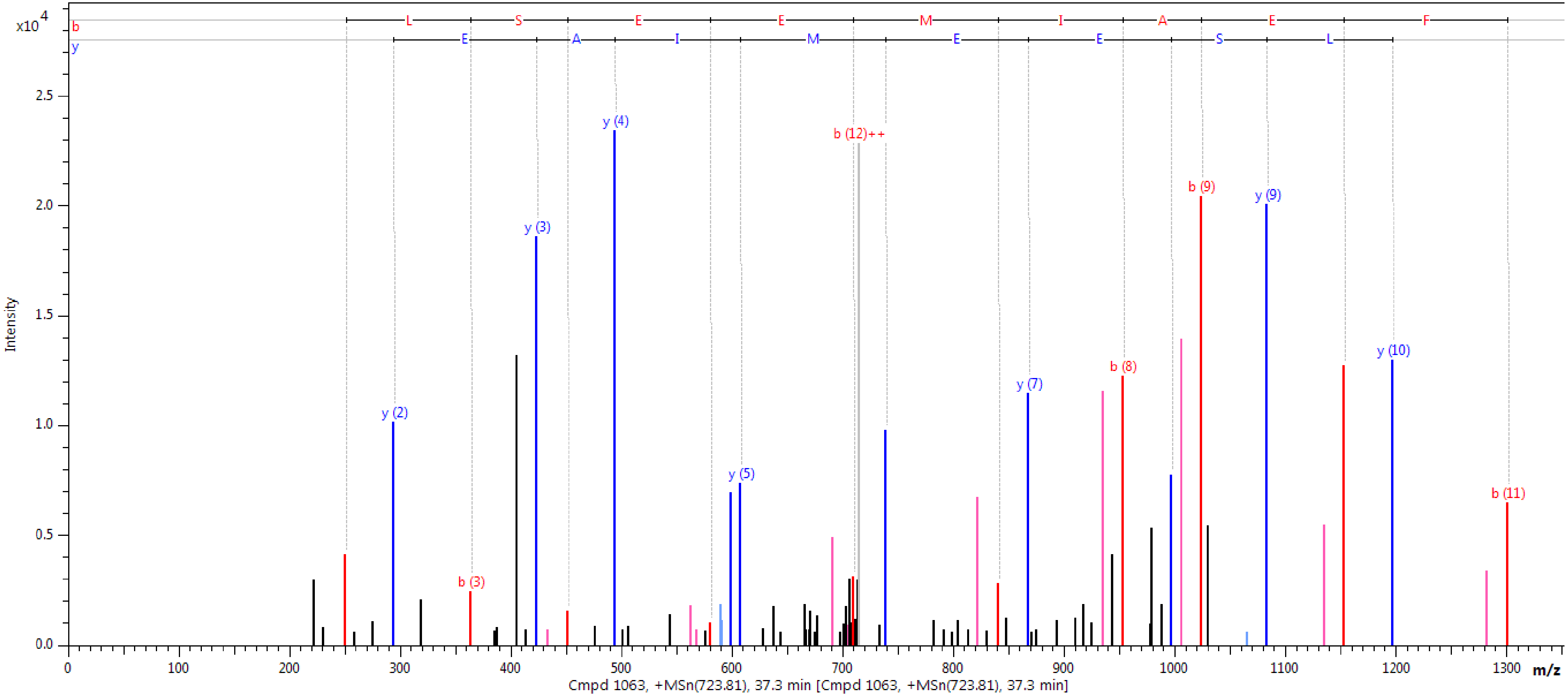

agresearch

Spectrum Report

Source: M:/Documents/Lamb meat protein project/1. Characterisation of lamb skeletal proteome/Real run - 5 lambs from LCF/mgf_Obj_1/SDS-insoluble_pellet_mgf/u-3_undil_both_all_all_use_all_the_line_removed.mgf

Protein: A-beta-globin, partial [Ovis aries]

Accession: gi|165900|gb|AAA31532.1|

Parent m/z: 633.39, 2+

Sequence: -.LLGNVLVVVLAR.H

Score: 36.75137500740678

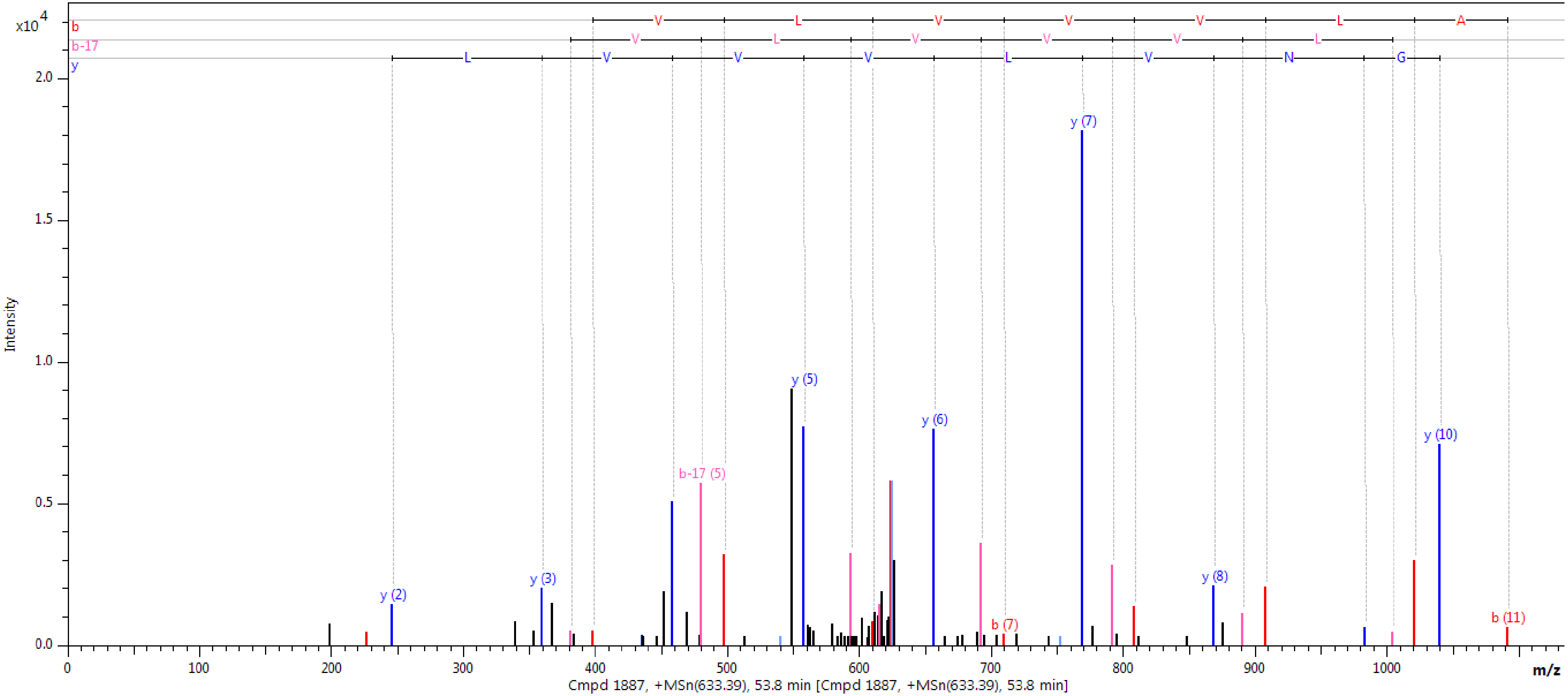

Spectrum Report

Source: M:/Documents/Lamb meat protein project/1. Characterisation of lamb skeletal proteome/Real run - 5 lambs from LCF/mgf_Obj_1/SDS-insoluble_pellet_mgf/u-3_undil_both_all_all_use_all_the_line_removed.mgf
Protein: myosin light chain 2 [Ovis aries]
Accession: gi|210148517|gb|ACJ09174.1|
Sequence: K.GADPEETILNAFK.V
Parent m/z: 702.778, 2+
Score: 31.05044599306012

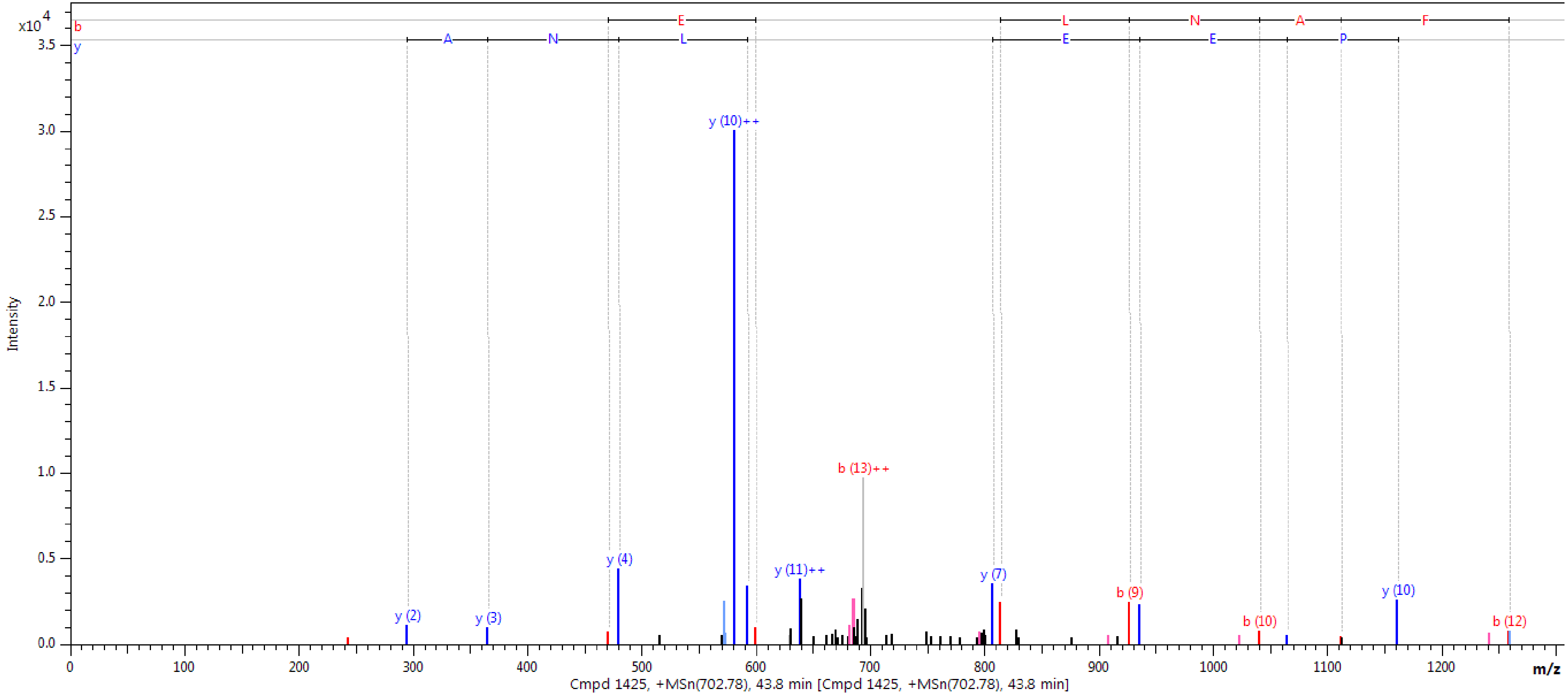

Spectrum Report

Source: M:/Documents/Lamb meat protein project/1. Characterisation of lamb skeletal proteome/Real run - 5 lambs from LCF/mgf_Obj_1/SDS-insoluble_pellet_mgf/u-3_undil_both_all_all_use_all_the_line_removed.mgf
Protein: PREDICTED: nebulin [Ovis aries]
Accession: gi|426221104|ref|XP_004004751.1|
Sequence: K.FSSPVDMLGVVLAK.K
Parent m/z: 731.845, 2+
Score: 29.380501767013634

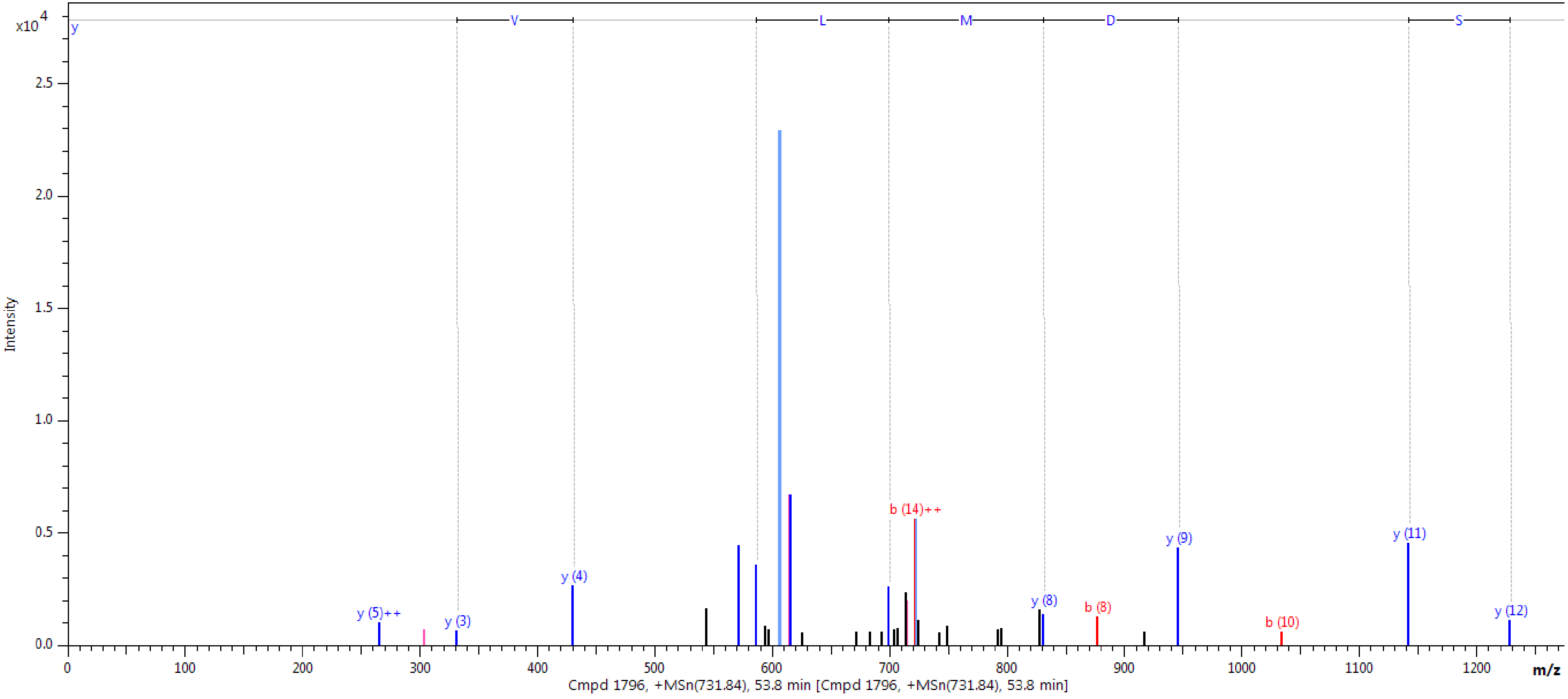

Spectrum Report

Source: M:/Documents/Lamb meat protein project/1. Characterisation of lamb skeletal proteome/Real run - 5 lambs from LCF/mgf_Obj_1/SDS-insoluble_pellet_mgf/u-3_undil_both_all_all_use_all_the_line_removed.mgf
Protein: PREDICTED: LOW QUALITY PROTEIN: heat shock protein beta-1 [Ovis aries]
Accession: gi|426255344|ref|XP_004021310.1|
Parent m/z: 582.302, 2+
Sequence: R.LFDQAFGLPR.L
Score: 28.226568835193994

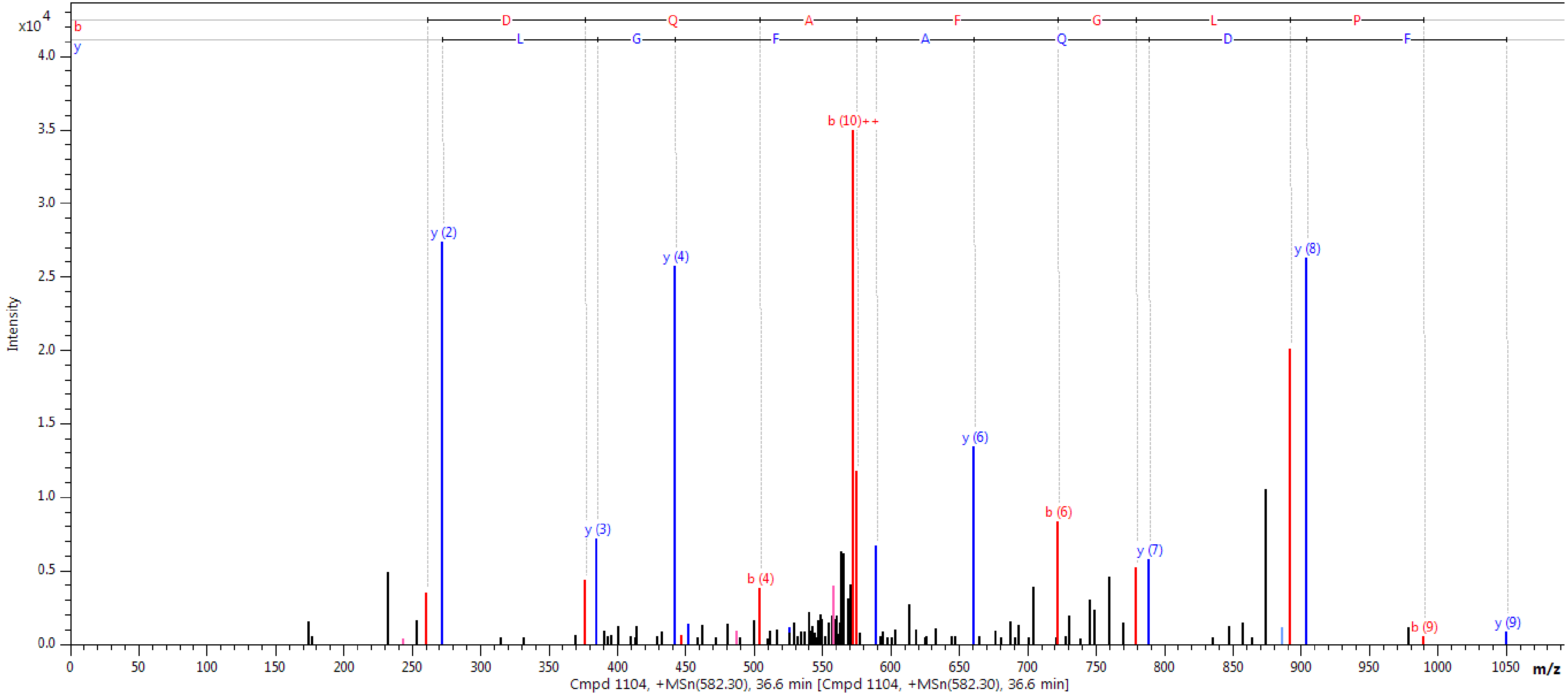

Spectrum Report

Source: M:/Documents/Lamb meat protein project/1. Characterisation of lamb skeletal proteome/Real run - 5 lambs from LCF/mgf_Obj_1/SDS-insoluble_pellet_mgf/u-3_undil_both_all_all_use_all_the_line_removed.mgf
Protein: PREDICTED: histone H2B type 1-K-like [Ovis aries]
Accession: gi|426250773|ref|XP_004019108.1|
Sequence: K.AMGIMNSFVNDIFER.I
Parent m/z: 872.308, 2+
Score: 27.330536937129878

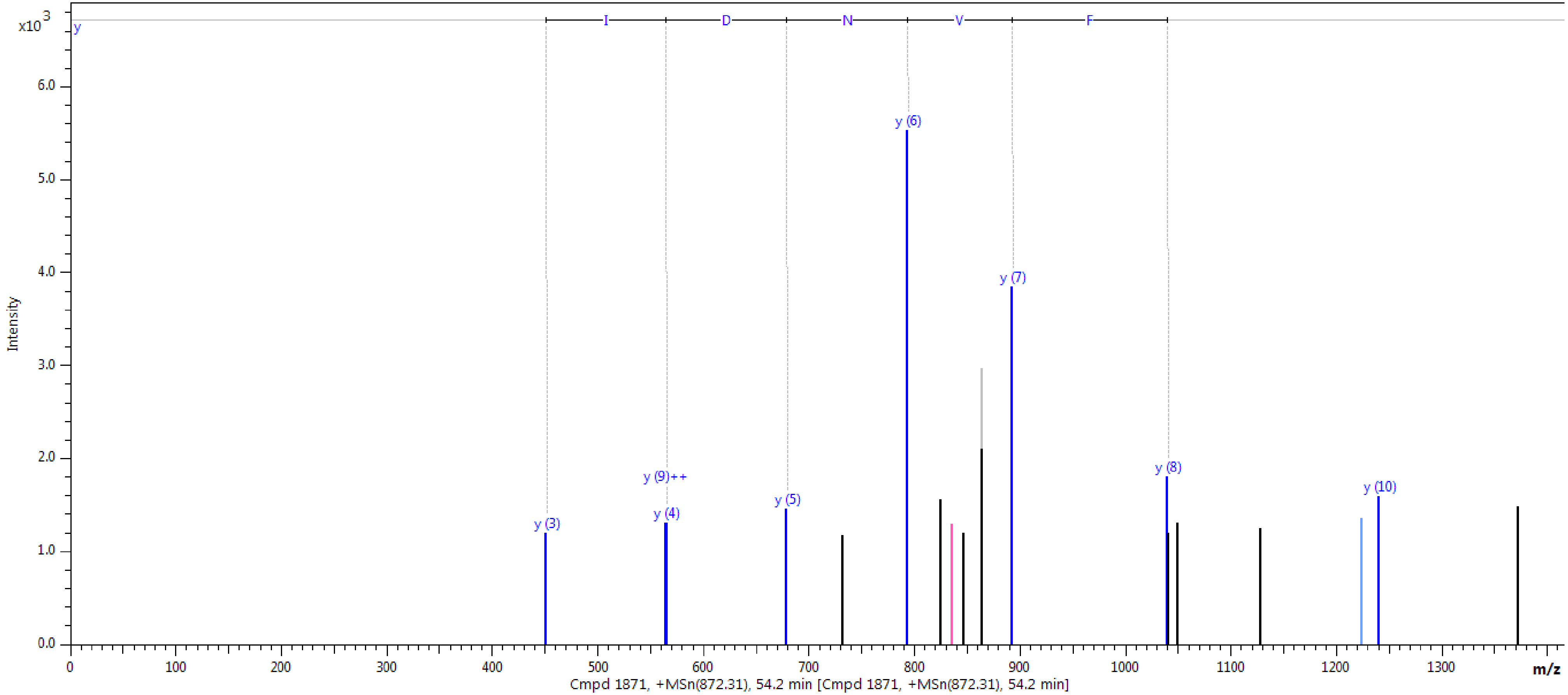

Spectrum Report

Source: M:/Documents/Lamb meat protein project/1. Characterisation of lamb skeletal proteome/Real run - 5 lambs from LCF/ mgf_Obj_1/SDS-insoluble_pellet_mgf/u-3_undil_both_all_all_use_all_the_line_removed.mgf
Protein: cytochrome oxidase subunit II [Ovis aries]
Accession: gi|66735537|gb|AAY53948.1|
Sequence: R.MLISSEDVLHSWAVPSLGLK.T
Parent m/z: 728.006, 3+
Score: 23.26719147233698

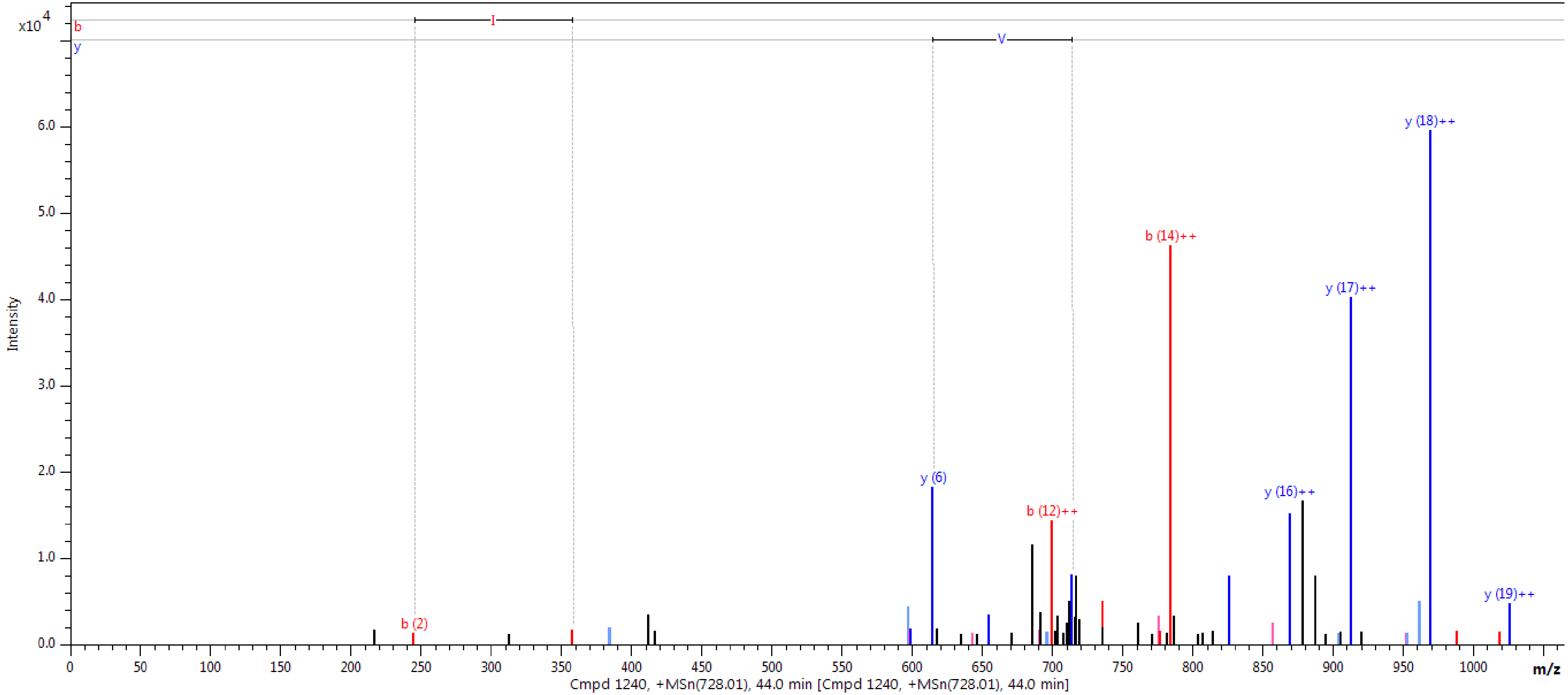

agresearch

Spectrum Report

Source: M:/Documents/Lamb meat protein project/1. Characterisation of lamb skeletal proteome/Real run - 5 lambs from LCF/ mgf_Obj_1/SDS-insoluble_pellet_mgf/u-3_undil_both_all_all_use_all_the_line_removed.mgf
Protein: PREDICTED: collagen alpha-1(IV) chain [Ovis aries]
Accession: gi|426236903|ref|XP_004012404.1|
Sequence: K.ILYHGYSLLYVQGNER.A
Parent m/z: 642.325, 3+
Score: 22.57899665312513

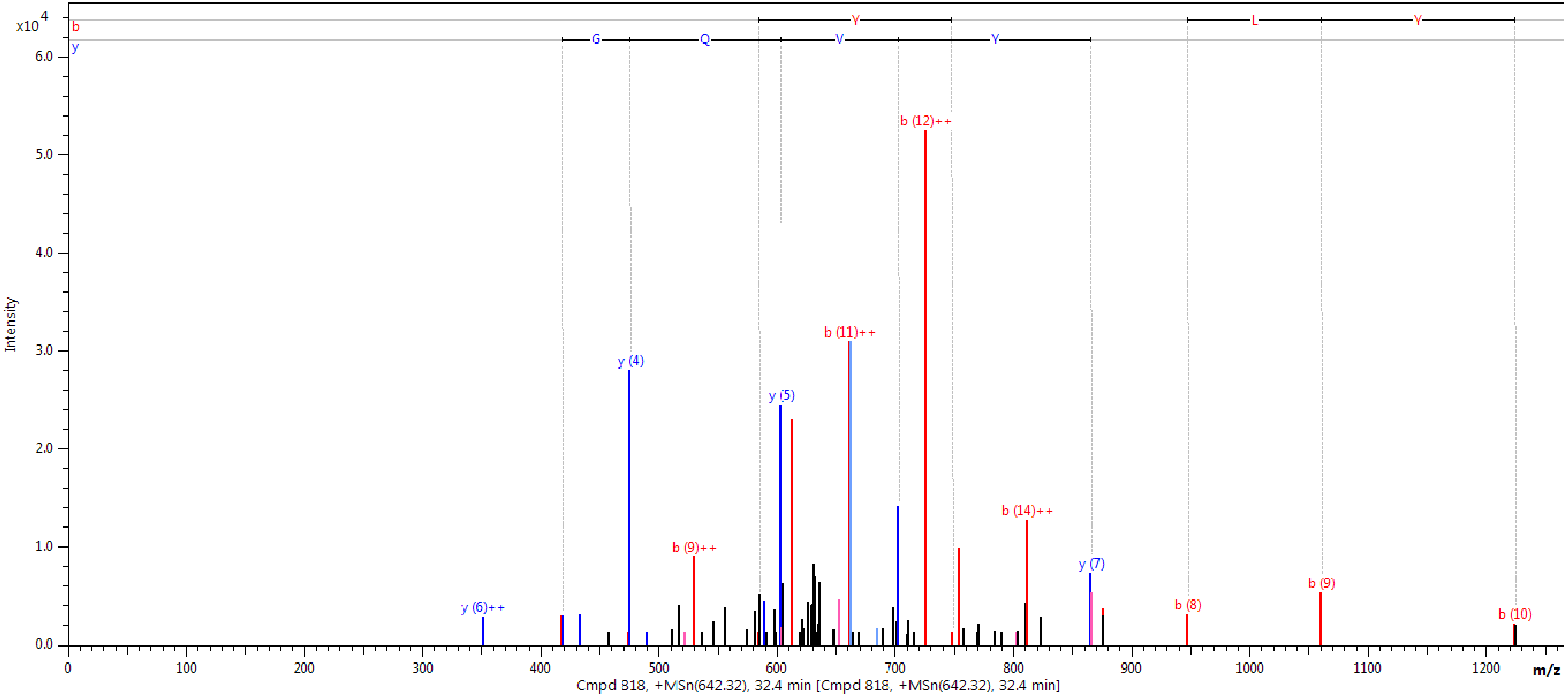

Spectrum Report

Source: M:/Documents/Lamb meat protein project/1. Characterisation of lamb skeletal proteome/Real run - 5 lambs from LCF/mgf_Obj_1/SDS-insoluble_pellet_mgf/u-3_undil_both_all_all_use_all_the_line_removed.mgf
Protein: four and a half LIM domains 1 protein [Ovis aries]
Accession: gi|256665363|gb|ACV04827.1|
Parent m/z: 590.814, 2+
Sequence: K.QVIGTGSFFPK.G
Score: 22.341057899336136

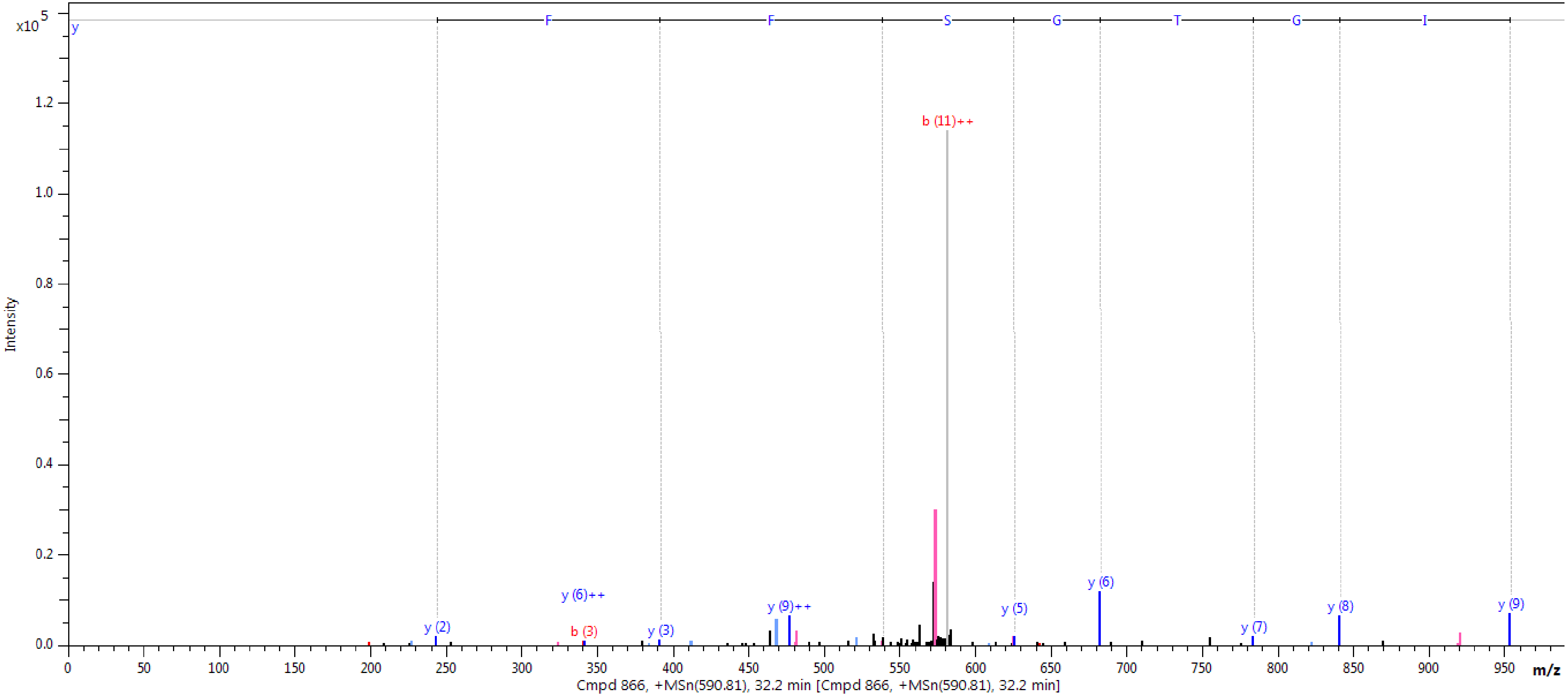

Spectrum Report

Source: M:/Documents/Lamb meat protein project/1. Characterisation of lamb skeletal proteome/Real run - 5 lambs from LCF/mgf_Obj_1/SDS-insoluble_pellet_mgf/u-3_undil_both_all_all_use_all_the_line_removed.mgf
Protein: PREDICTED: LIM domain-binding protein 3 isoform 4 [Ovis aries]
Accession: gi|426255902|ref|XP_004021587.1|
Parent m/z: 662.807, 2+
Sequence: K.SASYNLSLTLQK.S
Score: 21.64482546793378

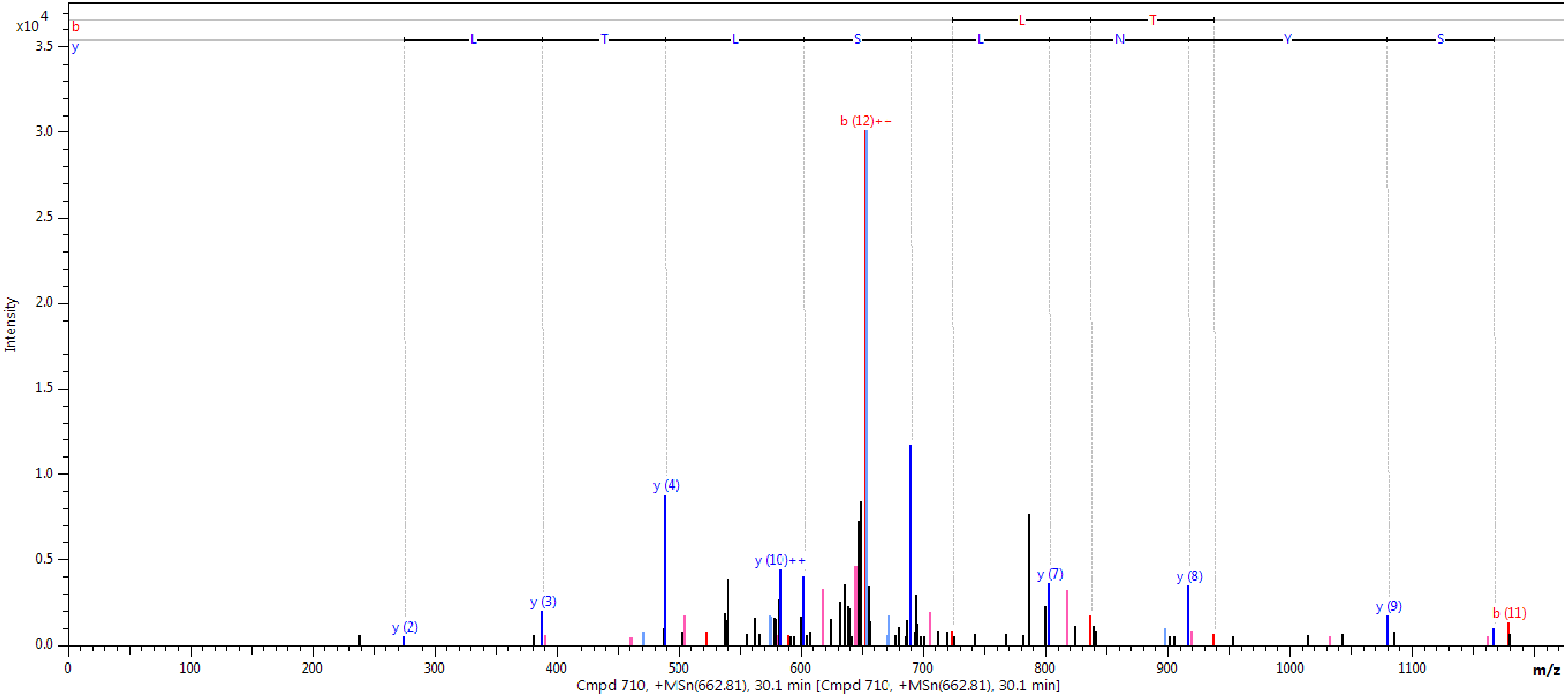

Supplement: Supplementary file 1 — Supplementary data [file mmc1.zip › Supple_data_3c_lamb_LL_prot_YMCD.pdf]
